# Supplementary material for: Extracting representations of cognition across neuroimaging studies improves brain decoding
Source: PLoS Comput Biol. 2021 May 3;17(5):e1008795. doi: 10.1371/journal.pcbi.1008795 (PMC8118532; doi:10.1371/journal.pcbi.1008795)
Supplement: S1 Appendix — This appendix discusses technical details of the multi-study decoding approach: the specific architecture, a 3-layer linear model, and the deep-learning technique used to regularize and train it. Discussion on the model design. In this appendix, we perform supportive experiments to explain the observed results, An ablation study of the various model components is provided to further support modelling choices. Reproduction details and tables. In this appendix, we provide implementation details for reproducibility, along with tables with quantitative results per contrast. (PDF) [file pcbi.1008795.s001.pdf]

S1 Appendix

The appendix is structured as follow: in the first section, we formalize the learning setting and method, after describing decoding baselines. In the second section, we perform supportive experiments to explain the observed results, and discuss various alternatives for the model, to further support modelling choices. Finally, we provide reproduction details, along with data and software notes. A visualization of all MSTONs components (that reproduces `https://cogspaces.github.io/assets/MSTON/components.html`) is provided for completeness in S1 Components.

Notations. We denote scalars, vectors and matrices using lower-case, bold lower-case and bold upper-case letters, e.g., x, $\boldsymbol{x}$ and $\boldsymbol{X}$. We denote the elements of $\boldsymbol{X}$ by $x_{i,j}$ and its rows by $\boldsymbol{x}_i$. We write x^j a value that is specific to study number j. We denote $\bar{x}$ a value built from an ensemble of value $(x_s)_s$. Finally, we write $[l]$ the set of integers ranging from 1 to l.

A Methods

We describe in mathematical terms the multi-layer decoder at the center of our method and provide supporting experiments. We start by formalizing the joint objective loss and the model training process.

A.1 Inter-subject decoding setting

We consider N task functional MRI studies (detailed in Table 1), on which we perform inter-subject decoding. In study number j, n^j subjects are made to perform one (or sometimes several) tasks. Acquired BOLD time-series are registered to a common template using non-linear spatial registration, after motion and slice-timing corrections. BOLD time-series are then fed to a standard analysis pipeline, which fits a linear model relating the design matrix of each experiment to the signal in every voxel. We use the *nistats* library for this purpose. From the obtained beta maps, we compute z-statistics maps, either associated with each of the base conditions (stimulus or task) of the experiments, or with contrasts defined by the study's authors. In both cases, z-maps are labeled with a number $1 \leq y \leq c^j$ that corresponds to k-th contrast/base condition (called contrast in the following). Overall, this produces a set of z-maps $(\boldsymbol{x}_i^j)_{i \in [c^j n^j]}$ living in $\mathbb{R}^p$, where p is the number of voxels, associated with a sequence of contrast $(k_i^j)_{i \in [c^j n^j]}$. The transformation from 3D brain images to 1D vectors is done using a grey-matter mask after alignment with the MNI template. We compare using a grey-matter mask with using a full brain mask in Section B.4.1. Inter-subject decoding proposes a model $f_\theta^j : \mathbb{R}^p \to [1, c^j]$ that predicts contrast identity from z-maps, i.e., $\hat{y}_i^j \triangleq f_\theta^j(\boldsymbol{x}_i^j)$, where θ is learned from training data, and the performance of the model is assessed on left-out subjects.

A.2 Baseline voxel-space decoder

Baseline decoders are linear classifier models defined separately for each study j, which take full brain images as input. For every input map $\boldsymbol{x}_i$ in $\mathbb{R}^p$, we compute the logits $\boldsymbol{l}_i$ in $\mathbb{R}^c$ as

$$\boldsymbol{l}_i(\boldsymbol{W}, \boldsymbol{b}) \triangleq \boldsymbol{W}\boldsymbol{x}_i + \boldsymbol{b},$$

where $\boldsymbol{W} \in \mathbb{R}^{c \times p}$ and $\boldsymbol{b} \in \mathbb{R}^c$ are the parameters of the linear model to be learned for study j—we drop the superscript j in this paragraph and the next for simplicity. Logits

are transformed into a classification probability vector using the softmax operator. At test time, we predict the label corresponding to the maximal logit, i.e., $\hat{y}_i = \mathrm{argmax}_{1 \leq y \leq c}\, l_{i,y}$. The model is trained on the data $(\boldsymbol{x}_i, y_i)_{i \in [n]}$ by minimizing the ℓ_2^2 regularized multinomial classification problem

$$\min_{\substack{\boldsymbol{W} \in \mathbb{R}^{c \times p} \\ \boldsymbol{b} \in \mathbb{R}^c}} -\frac{1}{n} \sum_{i=1}^{n} \Big(l_{i,y_i}(\boldsymbol{W}, \boldsymbol{b}) + \log(\sum_{k=1}^{c} \exp l_{i,k}(\boldsymbol{W}, \boldsymbol{b})) \Big) + \lambda \|\boldsymbol{W}\|_F^2, \tag{1}$$

where $\|\cdot\|_F^2$ is the Frobenius norm, that computes $\|\boldsymbol{W}\|_F^2 \triangleq \sum_{i,j=1}^{c,p} w_{i,j}^2$.

A.3 Baseline dimension reduced decoder

A variant of the voxel-based decoders is obtained by introducing a first-layer dimension reduction learned from resting-state data. This amounts to computing

$$\boldsymbol{l}_i(\boldsymbol{V}, \boldsymbol{b}, \boldsymbol{D}) \triangleq \boldsymbol{V}\boldsymbol{D}\boldsymbol{x}_i + \boldsymbol{b},$$

where $\boldsymbol{V}$ in $\mathbb{R}^{c \times k}$ forms the classifying weights of the model, and the matrix $\boldsymbol{D}$ in $\mathbb{R}^{k \times p}$ is *assigned* during training to functional networks learned on resting-state data, as detailed in A.5. Multiplying input data by $\boldsymbol{D}$ projects statistical images onto meaningful resting-state components, in an attempt to improve classification performance and reduce computation cost, akin to the methods proposed by [1, 2]. The model is trained by solving the convex objective (1) separately for each study, replacing $\boldsymbol{W}$ by $\boldsymbol{V}$ in $\mathbb{R}^{c \times k}$:

$$\min_{\substack{\boldsymbol{V} \in \mathbb{R}^{c \times k} \\ \boldsymbol{b} \in \mathbb{R}^c}} -\frac{1}{n} \sum_{i=1}^{n} \Big(l_{i,y_i}(\boldsymbol{V}, \boldsymbol{b}, \boldsymbol{D}) + \log(\sum_{k=1}^{c} \exp l_{i,k}(\boldsymbol{V}, \boldsymbol{b}, \boldsymbol{D})) \Big) + \lambda \|\boldsymbol{V}\|_F^2. \tag{2}$$

Our results (Fig 2C) show that decoding from functional networks is not significantly better than decoding from voxels directly. For both baselines, the parameter λ is found by half-split cross-validation. Training is performed using a L-BFGS solver [3]. We use non standardized maps $(\boldsymbol{x}_i)_i$ as input as we observed that standardization hinders performance.

A.4 Three-layer model description

Our three-layer model adds a second shared linear layer in between the projection on functional networks and the classification models. We still have

$$\boldsymbol{l}_i^j(\boldsymbol{W}^j, \boldsymbol{b}^j) \triangleq \boldsymbol{W}^j \boldsymbol{x}_i^j + \boldsymbol{b}^j,$$

for every z-map i and study j. However, we introduce a coupling between the various parameters $(\boldsymbol{W}^j)_{j \in [N]}$ of each study: they should decompose on common basis $\boldsymbol{L}\boldsymbol{D}$, where $\boldsymbol{L}$ is estimated from the whole corpus of data, and $\boldsymbol{D}$ is the resting-state dictionary presented above. Formally, we assume that there exist a matrix $\boldsymbol{L}$ in $\mathbb{R}^{l \times k}$ with $l < k < p$, and a set of matrices $(\boldsymbol{U}^j)_{j \in [N]}$ so that for all $j \in [N]$, the classification weights of (1) writes

$$\boldsymbol{W}^j \triangleq \boldsymbol{U}^j \boldsymbol{L}\boldsymbol{D}, \quad \text{where} \quad \boldsymbol{U}^j \in \mathbb{R}^{c^j \times l}. \tag{3}$$

The matrix $\boldsymbol{D}$ corresponds to the first-layer weights pictured in Fig 1, $\boldsymbol{L}$ to the second-layer weights, and $(\boldsymbol{U}^j, \boldsymbol{b}^j)_j$ to the various classification heads of the third layer. In this work, we choose $k = 465$ and $l = 128$. While $\boldsymbol{D}$ remains fixed, the second-layer matrix $\boldsymbol{L}$ and the N classification heads $(\boldsymbol{U}^j)_{j\in[N]}$ are jointly learned during training, a necessary step toward improving decoding accuracy. The "shared-layer" parameterization (3) is a common approach in multi-task learning [4, 5], and should allow *transfer learning* between decoding tasks, under certain conditions. In our setting, both the data distribution from the different studies and the classification task associated with each study differ—this is a particular case of *inductive transfer learning*[1], described by [6].

Modeling. Without refinement nor regularization, we seek a local minimizer of the following non-convex objective function, which combines the classification objectives (1) from all studies, with parameter sharing:

$$\min_{\substack{\boldsymbol{L}\in\mathbb{R}^{l\times k}\\(\boldsymbol{U}^j,\boldsymbol{b}^j)_j}} -\sum_{j=1}^{N} \frac{(n^j)^\beta}{n^j} \sum_{i=1}^{n^j} \Big(l^j_{i,y_i}(\boldsymbol{U}^j, \boldsymbol{b}^j, \boldsymbol{L}) \qquad (4)$$

$$- \log(\sum_{k=1}^{c^j} \exp l^j_{i,k}(\boldsymbol{U}^j, \boldsymbol{b}^j, \boldsymbol{L}))\Big),$$

where the dependence on $\boldsymbol{D}$ is left implicit. The scalar β in $[0, 1]$ is a parameter that regulates the importance of each study in the joint objective, that we further discuss in B.8. We note that the importance of the study j to find the latent parameter $\boldsymbol{L}$ depends on the amplitude of the gradient $\frac{\partial \ell_j}{\partial \boldsymbol{L}}$ that does not depend on the number of tasks c^j: in particular, for each study j, contrast $1 \leq k \leq c^j$ and subject $1 \leq i \leq n^j$, the susceptibility of the loss to the logits $l^j_{i,k}$ is such that $\frac{\partial \ell_j}{\partial l^j_{i,k}} \in [-1, 1]$, independent from c^j.

Regularization. We observe that minimizing (4) leads to strong overfitting and low performance on left-out data, with performance similar to fitting (1) without regularization, separately for each study. Adding ℓ_2 regularization to the second and third layer weights gives little benefit, as we discuss in Section B.2.3. On the other hand, introducing *dropout* [7] during training alleviates the overfitting issue and fosters transfer learning. Dropout is a stochastic regularization method that prevents the weights from each layer to co-adapt by perturbating them with multiplicative noise during training. It ensures that the information is well spread across coefficients rows and columns [8]. In our case, this favors transfer learning, as it ensures that no single row of $\boldsymbol{L}$, or in plain words no task-optimized network, becomes dedicated to a *single* study. We further compare the different methods that we can use to foster transfer of information between studies in Section B.2.

We use the variational flavor of dropout [9] to make the dropout rate for every study adaptive. This slightly improves performance compared to binary dropout: every decoding task requires a different level of regularization, depending on the size of the study and the hardness of the task, and it is beneficial to estimate it from data. In details, during training, at every iteration, for every input sample i of a mini-batch from study j, we randomly draw two multiplicative noise matrices

$$\boldsymbol{M}_D = \mathrm{Diag}([m_{D,t}]_{t\in[k]}), \quad \boldsymbol{M}^j_L = \mathrm{Diag}([m_{L,t}]_{t\in[l]}),$$

[1]This case is less studied than the classical multi-task setting where input data are single-source but learning tasks are multiple.

where $m_{D,t} \sim \mathcal{N}(1, \alpha)$ and $m_{L,t} \sim \mathcal{N}(1, \alpha^j)$, with α fixed and α^j estimated from data.[2] We then compute the noisy logits

$$\boldsymbol{l}_i^j \triangleq \boldsymbol{U}^j \boldsymbol{M}_L^j \boldsymbol{L} \boldsymbol{M}_D \boldsymbol{D} \boldsymbol{x}_i^j + \boldsymbol{b}^j,$$

and use these to compute the loss (5), to which we add a regularization term that regulates the learning of α^j, introduced by [10]. We compute the gradient with respect to $\boldsymbol{L}$, $\boldsymbol{U}^j$, $\boldsymbol{b}^j$ using the local reparametrization trick [9]. We refer to [10] for more details on variational dropout and a Bayesian grounding of this approach.

Optimization. We solve the problem (4) using stochastic optimization. Namely, at each iteration, we compute an unbiased estimate of the objective (4) and its gradient with respect to the model parameters, in order to perform a stochastic gradient step. For this, we randomly choose the study j with a probability proportional to $(n^j)^\beta$, and consider a mini-batch of z-maps $(\boldsymbol{x}_i^j)_{j \in B}$ that we use to compute the unbiased objective estimate

$$-\frac{1}{B} \sum_{i=1}^{n} -\Big(l_{i,k_i}^j \log(\sum_{k=1}^{c} \exp l_{i,k}^j) \Big), \tag{5}$$

from which we compute gradients with respect to $\boldsymbol{L}$, $\boldsymbol{U}^j$ and $\boldsymbol{b}^j$.

Optimization is performed using *Adam* [11], a flavor of stochastic gradient descent that depends less on the step-size. We use batch normalization [12] between the second and third layer, as it slightly improves performance—it reduces potential negative transfer learning—and training speed.

A.5 Resting-state data

As mentioned above, we use resting-state data to compute the first-layer weights $\boldsymbol{D}$ in $\mathbb{R}^{k \times p}$, where $k = 512$. Such high-order dictionaries are known to perform well for decoding [13]. We consider data from the HCP900 release, and stack all records to obtain a data matrix $\boldsymbol{X}$ in $\mathbb{R}^{n \times p}$. We then use an online solver [14] to solve the sparse non-negative matrix factorization problem

$$\boldsymbol{A}, \boldsymbol{D} \triangleq \underset{\boldsymbol{D} \in \mathcal{C}, \boldsymbol{A} \in \mathbb{R}^{k \times n}}{\operatorname{argmin}} \|\boldsymbol{X} - \boldsymbol{A}\boldsymbol{D}\|_F^2 + \lambda \|\boldsymbol{A}\|_F^2, \tag{6}$$

where the constraint $\mathcal{C} = \big\{ \boldsymbol{D} \in \mathbb{R}^{k \times p}, \boldsymbol{D} \geq 0, \|\boldsymbol{d}_j\|_1 \leq 1 \,\forall\, j \in [k] \big\}$ enforces every dictionary component to live in the simplex of $\mathbb{R}^p$, ensuring sparsity and non-negativity of the functional networks. The sparsity level is chosen so that the functional networks $\boldsymbol{D}$ cover the whole brain with as little overlap as possible. Larger overlap leads to more correlated activations input to the second layer, yielding a harder learning problem. With lower coverage, we would miss important information to decode some of the predicted psychological conditions. We refer to [13] for further discussion on selecting sparsity level when using dictionary learning in fMRI analysis.

Second-layer initialization. To initialize the weights of the second layer, we learn a smaller dictionary $\boldsymbol{D}_l$ in $\mathbb{R}^{l \times p}$ as in (6), where $l = 128$. We then compute the initial weights $\boldsymbol{L}_l$ so that $\boldsymbol{D}_l \approx \boldsymbol{L}_l \boldsymbol{D}$ using least-square regression. This way, applying the first two layers initially amount to projecting data onto $l = 128$ larger functional networks $\boldsymbol{D}_l$, which is a reasonable prior for reducing the dimension of brain statistical maps. Using this resting-state based initialization slightly improves performance, as we discuss in Section B.4.

[2] This *Gaussian* dropout has a similar behavior to the more commonly used binary dropout with parameter $p = \frac{\alpha}{\alpha+1}$.

Grey matter restriction. To help interpreting the obtained model, we found it helpful to remove from $\boldsymbol{D}$ the fraction (9%) of the functional networks components located in the white matter and the cerebrospinal fluid areas, turning $k = 512$ into $k = 465$. We discuss the effect of this restriction in Section B.4.1.

A.6 Model introspection with ensembling

Given any invertible matrix $\boldsymbol{M}$ in $\mathbb{R}^{l \times l}$, the non regularized version of the objective (4) is left invariant when transforming $\boldsymbol{L}$ into $\boldsymbol{M}\boldsymbol{L}$ and each $\boldsymbol{U}^j$ into $\boldsymbol{U}^j \boldsymbol{M}^{-1}$. This prevents us from interpreting the coefficients of $\boldsymbol{L}$ at the end of the training procedure, and to retrieve relevant networks by reading the weights of the second weight. The only aspect of $\boldsymbol{L}$ that remains unchanged after a linear parameter transformation is its span. Dropout regularization, which favors the canonical directions in matrix space [7], should break this symmetry, but does not help to uncover meaningful directions in the span of $\boldsymbol{L}$ in practice.

On the other hand, we found that this span was remarkably stable across runs on the same data, whether when varying initialization or simply the order in which data are streamed during stochastic gradient descent. More precisely, we trained our model 100 times with different seeds, and concatenated the weights $(\boldsymbol{L}_r)_r$ of the second-layer into a big matrix $\bar{\boldsymbol{L}}$. We performed a SVD on this matrix, and observed that the first $l = 128$ components captured 98% of the variance of $\bar{\boldsymbol{L}}$ when using the same initialization but different streaming order, and 96% when also using a different random initialization. Despite the many local minima that objective (4) admits, the span of $\boldsymbol{L}$ thus remains close to some reference span that we can extract with a matrix factorization method.

The above remark suggested the following ensemble method. We run the learning algorithm $R = 100$ times, and store the weights $(\boldsymbol{L}_r)_r$ of the second layer for each run, along with the average matrices and biases

$$\bar{\boldsymbol{W}}^j = \frac{1}{R} \sum_{r=1}^{R} \boldsymbol{U}_r^j \boldsymbol{L}_r \qquad \bar{\boldsymbol{b}}^j = \frac{1}{R} \sum_{r=1}^{R} \boldsymbol{b}_r^j, \quad \forall\, j \in [N],$$

that combine the second and third-layer weights and biases for each study j and run N, and average them across runs. We then stack the second-layer weights $(\boldsymbol{L}_r)_r$ into a tall matrix $\tilde{\boldsymbol{L}} \in \mathbb{R}^{lR \times k}$ on which we perform sparse non-negative matrix factorization. Namely, we compute $\bar{\boldsymbol{L}} \in \mathbb{R}^{l \times k}$, the new weight matrix for the second layer, solving

$$\bar{\boldsymbol{L}} \triangleq \operatorname*{argmin}_{\boldsymbol{L} \in \mathcal{C}} \min_{\boldsymbol{K} \in \mathbb{R}^{lR \times l}} \frac{1}{2} \|\tilde{\boldsymbol{L}} - \boldsymbol{K}\boldsymbol{L}\|_F^2 + \lambda \|\boldsymbol{K}\|_F^2,$$

where $\mathcal{C} = \left\{\boldsymbol{L} \in \mathbb{R}^{l \times k}, \boldsymbol{L} \geq 0, \|\boldsymbol{l}_j\|_1 \leq 1\, \forall\, j \in [l]\right\}$ and λ regulates the sparsity of $\bar{\boldsymbol{L}}$—performance little depends on λ provided it leads to finding $\bar{\boldsymbol{L}}\boldsymbol{D}$ with more than 50% non-zero voxels (see Section C.1). Higher λ leads to sparser maps with lower performance as brain coverage is reduced, while lower λ gives good performances but lower interpretability of the extracted networks. Finally, we compute new weights $\bar{\boldsymbol{U}}^j$ for all the classification heads of the third layer, so that $\bar{\boldsymbol{W}}^j \approx \bar{\boldsymbol{U}}^j \bar{\boldsymbol{L}}$, from a least-square point of view, for each study j. The new model is then formed of parameters $\boldsymbol{D}, \bar{\boldsymbol{L}}, (\bar{\boldsymbol{U}}^j, \bar{\boldsymbol{b}}^j)_{j \in [N]}$. In plain words, we obtain sparse non-negative second-layer weights $\bar{\boldsymbol{L}}$, and define from these weights a new model that is as close as possible to the ensemble of all learned models $\left\{\boldsymbol{D}, \boldsymbol{L}_r, (\boldsymbol{U}_r^j, \boldsymbol{b}_r^j)_j\right\}_{r \in [R]}$.

The rows of $\bar{\boldsymbol{L}}$ are now interpretable separately, as the non-negative and sparse constraints have broken the inherent parameter invariance of the original model. The rows of $\bar{\boldsymbol{L}}$ hold the coefficients for combining resting-state networks held in $\boldsymbol{D}$ into l multi-study task-optimized networks $\bar{\boldsymbol{L}}\boldsymbol{D}$ in $\mathbb{R}^{l \times p}$. We initialize the sparse NMF

algorithm with the weights $\boldsymbol{L}_l$ computed in Section A.5, to inject a small prior regarding final MSTON distribution: before running NMF, those are set to $\boldsymbol{L}_l \boldsymbol{D} \approx \boldsymbol{D}_l$, i.e., are close to large resting-state functional networks.

We observed that directly enforcing negativity/sparsity over $\boldsymbol{L}$ during the training of the model led to a strong loss in accuracy. Finding a consensus model through a post-hoc ensembling transformation thus proves to be the right solution for obtaining both performance improvement *and* interpretability.

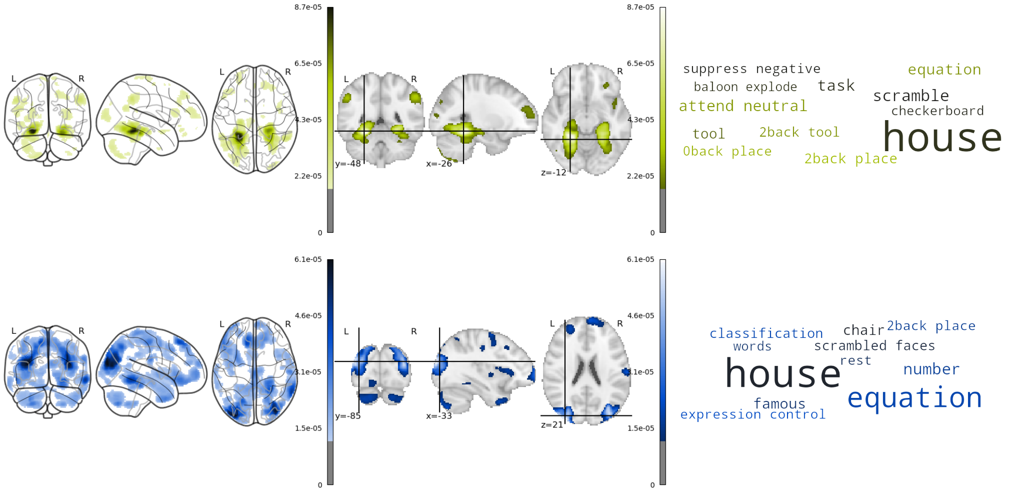

A. MSTONs recruited by the "house" base condition

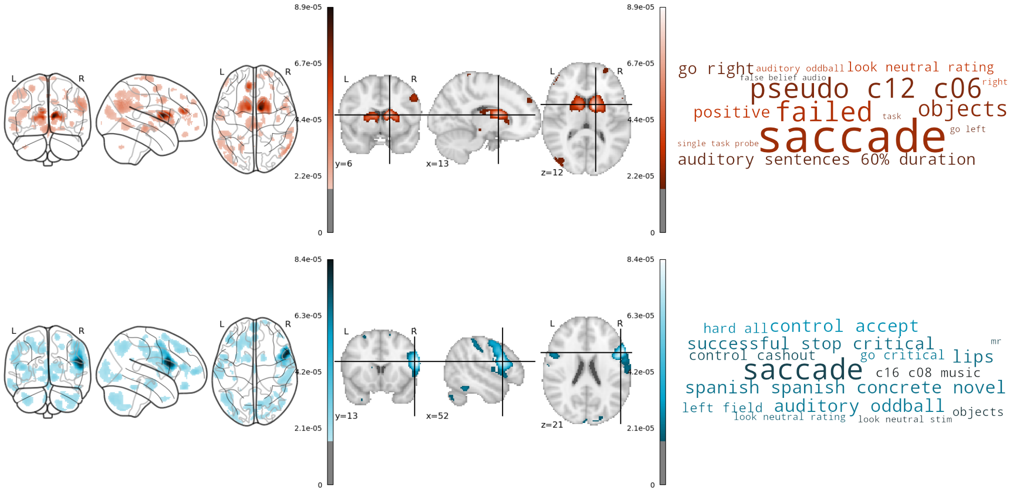

A. MSTONs recruited by the "saccade" base condition

Fig A. Examples of MSTONs that are activated in many different tasks.

B Discussion on the model design

In this section, we discuss various choices made for designing our model and training procedures. To this end, we perform diverse quantitative and qualitative comparisons of model variants.

B.1 Understanding the role of task-optimized networks

We first provide new examples of MSTONs to enlight their properties. Then, we propose several measurements and experiments that allow to better understand how the dimension reduction performed by projecting on multi-study task-optimized networks brings quantitative improvements in decoding.

B.1.1 Other examples of MSTONs

Fig 4 shows a selection of MSTONs that are well associated with relevant clusters of base psychological conditions. Other MSTONs are of interest to discuss the multi-study decoding approach, as we now discuss.

Some base conditions recruit many MSTONs. We observe that some base psychological conditions are strongly correlated with many different MSTONs, as exemplified in Fig A. The "saccade" condition [15] triggers a very distributed response of the brain, which is the reason why it appears often in the word-clouds. The base condition "house" is in particular part of the HCP Working Memory [16] task. Decoding it versus the other HCP conditions gives a classification map for which much of the lateral visual cortex is positively activated, hence the appearance of the word "house" in the MSTONs that includes a fraction of these regions.

B.1.2 Performance of MSTONs on new studies

We argue that using the joint objective (4) improves decoding performance because the data from every study influence the model weights in both the second layer *and* all components of the third layer. This can be measured as follows. We compare the performance of learning task-optimized networks on all studies but a target one, before using the second layer as a fixed dimension reduction for fitting a decoder from the target (unobserved) study. Using this technique, information transfer from the corpus to the new study can only be imputed to the fact that the second layer has captured a dimension reduction for brain images that is efficient for decoding in general. In other words, the task optimized networks learned on N - 1 studies form a universal prior of cognition that generalizes to new paradigms.

We observe in Fig B that decoding cognitive processes from externally learned MSTON indeed performs better than decoding from voxels (3.7% mean accuracy gain, 67% experiments with net increase[3]). On the other hand, leveraging a low-dimensional representation of brain images using all studies, including the target one, during training (1.9% mean accuracy gain, 75% experiments with net increase) performs even better. This can only be explained by the fact that joint objective also fosters transfer between the classification heads of the third layer during training.

B.1.3 Effect of brain-map dimension reduction

In a dual perspective, we study the effect of reducing the dimension of the input data with the first two linear layers. We set $\boldsymbol{M} = \bar{\boldsymbol{L}}\boldsymbol{D}$ in $\mathbb{R}^{l \times p}$ to hold the task-optimized networks on each row, and compute, for all input statistical map $\boldsymbol{x}$ in $\mathbb{R}^p$, the projection of $\boldsymbol{x}$ onto span$(\boldsymbol{M})$, namely

$$\boldsymbol{x}_{\text{proj}} = \boldsymbol{M}^{\boldsymbol{T}}(\boldsymbol{M}\boldsymbol{M}^{\boldsymbol{T}})^{-1}\boldsymbol{M}\boldsymbol{x} \in \mathbb{R}^p.$$

$\boldsymbol{x}_{\text{proj}}$ is thus a denoised, low-dimensional representation of the brain map $\boldsymbol{x}$, held in the span of the l multi-study task-optimized networks contained in matrix $\boldsymbol{M}$. We compare different maps $\boldsymbol{x}$ to their projection $\boldsymbol{x}_{\text{proj}}$ in Fig C.

B.2 Fostering transfer learning

We now discuss the various way in which we can foster information sharing across studies in training our multi-layer model.

[3]Due the fact that half-split folds are overlapping and performance betweens studies are interacting, model comparison experiments are not independent. This suggests to report the amount of advantageous model comparisons instead of classical null hypothesis testing, that assumes independence of trials.

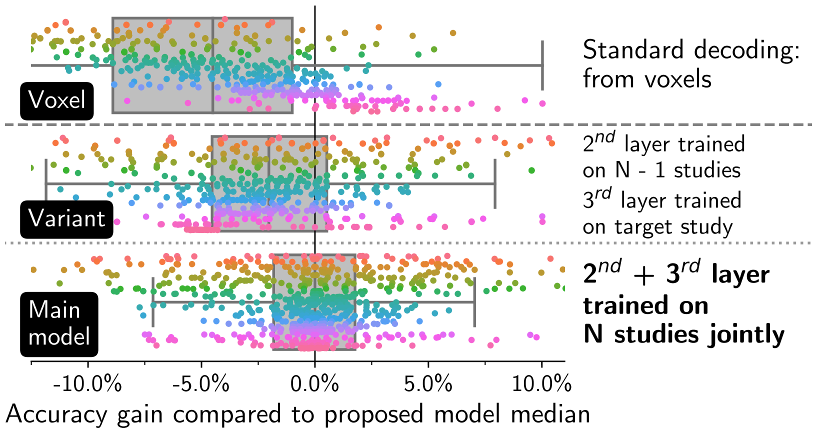

Fig B. Quantitative improvement linked to training the model on the joint objective (4), versus improvement linked to transfer in the second-layer only. Box plots calculated over 20 random data half-split and all studies.

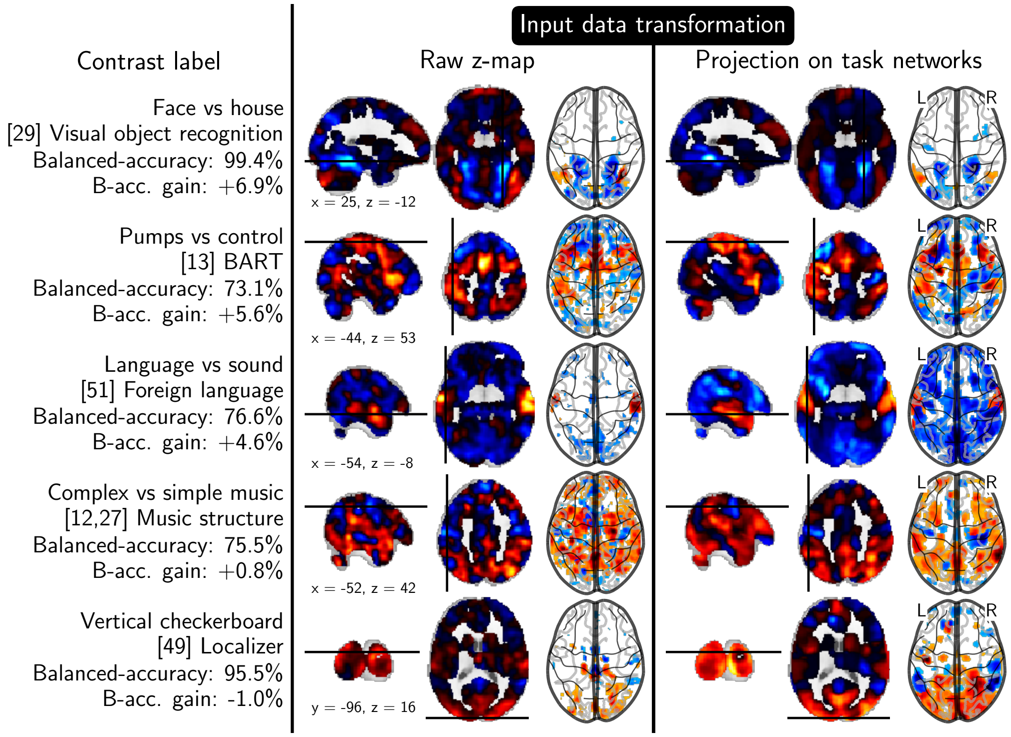

Fig C. Effect of projecting z-maps onto MSTONs. In a dual perspective to Fig 6, input data are simplified by the projection onto task-optimized networks, and become easier to classify.

B.2.1 The need for objective coupling

Without modification nor constraint on the second layer output size l, we cannot expect to observe any transfer learning by solving the joint objective (4). Indeed, in the general case where we allow $l \geq c \triangleq \sum_{j=1}^{N} c^j$, we let $(\tilde{\boldsymbol{V}}^j, \boldsymbol{b}^j)_j$ be the unique solutions of the N non-regularized convex problems (2). We let $\tilde{\boldsymbol{V}} \in \mathbb{R}^{c \times k}$ be the vertical concatenation of $(\boldsymbol{V}^j)_j$. We then form the matrices

$$\boldsymbol{L} = \begin{bmatrix} \tilde{\boldsymbol{V}} \\ \mathbf{o} \in \mathbb{R}^{l-c \times k} \end{bmatrix} \in \mathbb{R}^{l \times k} \qquad \text{and} \tag{8}$$

$$\begin{bmatrix} \boldsymbol{U}^1 \\ \vdots \\ \boldsymbol{U}^N \end{bmatrix} \triangleq \left[\boldsymbol{I}_c \in \mathbb{R}^{c \times c}, \mathbf{o} \in \mathbb{R}^{l-c \times l} \right],$$

where $\boldsymbol{I}_c$ is the identity matrix of $\mathbb{R}^{c \times c}$. $\boldsymbol{L}$ is thus split into row-blocks $(\tilde{\boldsymbol{V}}^j)_j$, dedicated to and learned on *single studies*. It follows from elementary considerations that the matrices $(\boldsymbol{L}, (\boldsymbol{U}^j, \boldsymbol{b}^j)_j)$ form a global minimizer of (4), that is formed from the solutions of the *separated* problems (2). It is therefore possible to find solutions of (4) for which no transfer occurs. Two possible modifications of the objective (4) allow to enforce transfer: Dropout regularization and low-rank constraints, that we present and compare.

B.2.2 Dropout as a transfer incentive

First, as presented in Section A, we can use dropout between the second layer weight L and the third layer head weights $\boldsymbol{U}^j$. Dropout prevents constructions of block-separated solution of objective (4) similar to the one proposed in (8). Indeed, every reduced sample $\boldsymbol{L}\boldsymbol{D}\boldsymbol{x}_i^j$ fed to the third layer classification head j can see any of his features corrupted by multiplicative noise $\boldsymbol{M}_L$ during training. This pushes the model to capture information relevant for all studies in every activation of the second layer. In other word, the projection performed on any task-optimized network $\boldsymbol{l}_h\boldsymbol{D}$, for $h \in [l]$ should be relevant for decoding every study. This fosters transfer learning as $\boldsymbol{L}$ carries multi-study aggregated information at the end of training, unlike in (8).

B.2.3 Transfer through low-rank constraints/penalty

A second approach to transfer is to force the matrices

$$\boldsymbol{V} \triangleq \begin{bmatrix} \boldsymbol{V}^1 \\ \vdots \\ \boldsymbol{V}^N \end{bmatrix} \triangleq \begin{bmatrix} \boldsymbol{U}^1 \\ \vdots \\ \boldsymbol{U}^N \end{bmatrix} \boldsymbol{L},$$

formed of the parameters of the joint objective (4) to be *low-rank*. In this case, the subspace of $\mathbb{R}^{c \times k}$ in which $\boldsymbol{V}$ evolves is strictly smaller than $\mathbb{R}^{c \times k}$, and we cannot always find a global minimum of the joint objective (4) formed with the solutions $\tilde{\boldsymbol{V}}$ of the separate objectives (2), as we did in the construction (8). As a consequence, the data from studies truly influence the solutions $(\boldsymbol{L}, (\boldsymbol{U}^j, \boldsymbol{b}_j)_j)$ of (4), and transfer is theoretically possible.

The low-rank property may be enforced in two ways. First, we may set it as a hard constraint, setting $l < c$ in the joint objective (4). This is in practice what we do when selecting $l = 128$, as $c = 545$ in our experiments.

Alternatively, following [17], we may resort to a convex objective function parameterized by $\boldsymbol{V}$ in $\mathbb{R}^{c \times k}$, that penalizes the rank of $\boldsymbol{V}$. We learn $\boldsymbol{V}^j$ in $\mathbb{R}^{c^j \times k}$ for

all study j in $[N]$ solving the joint objective

$$\min_{(\boldsymbol{V}^j, \boldsymbol{b}^j)_j} -\sum_{j=1}^{N} \frac{(n^j)^\beta}{n^j} \sum_{i=1}^{n^j} \Big(l^j_{i,y_i}(\boldsymbol{V}^j, \boldsymbol{b}^j) - \log(\sum_{k=1}^{c^j} \exp l^j_{i,k}(\boldsymbol{V}^j, \boldsymbol{b}^j)) \Big) + \lambda \Big\| \Big[\boldsymbol{V}^{1\top} \dots \boldsymbol{V}^{N\top} \Big] \Big\|_\star, \tag{9}$$

where $\|\boldsymbol{V}\|_\star$ is the nuclear norm of $\boldsymbol{V}$, defined as $\sum_{i=1}^{\min(c,k)} \sigma_i(\boldsymbol{V})$, where $(\sigma_i(\boldsymbol{V}))_i$ are the singular values of $\boldsymbol{V}$. The nuclear norm is a convex proxy for the rank of matrix $\boldsymbol{V}$. As a consequence, the rank of the solution decreases from $\min(c, k)$ to 0 as λ increases. The objective (9) is solvable using proximal methods, e.g., FISTA [18]. However, these methods become unpractical when c becomes large—it requires to perform a $c \times c$ singular value decomposition at each iteration. Fortunately, there exists a non-convex objective [19], amenable to stochastic gradient descent [20], that includes the solution of (9) as a minimizer. It is obtained by setting $l = \max(x, k)$ and adding ℓ_2^2 penalties to the objective (4):

$$\min_{\substack{\boldsymbol{L} \in \mathbb{R}^{l \times k} \\ (\boldsymbol{U}^j, \boldsymbol{b}^j)_j}} -\sum_{j=1}^{N} \frac{(n^j)^\beta}{n^j} \sum_{i=1}^{n^j} \Big(l^j_{i,y_i}(\boldsymbol{U}^j, \boldsymbol{b}^j, \boldsymbol{L}) - \log(\sum_{k=1}^{c^j} \exp l^j_{i,k}(\boldsymbol{U}^j, \boldsymbol{b}^j, \boldsymbol{L})) \Big) + \frac{\lambda}{2} \Big(\|\boldsymbol{L}\|_F^2 + \sum_{j=1}^{N} \|\boldsymbol{U}^j\|_F^2 \Big).$$

We solve this objective using *Adam*, similarly to the main method. It is possible to continue using dropout in between the first and second layer while enforcing $\boldsymbol{V}$ to be low-rank—this can then be understood as a regularization technique through feature noising [21].

B.2.4 Empirical comparison of transfer penalties

Dropout versus ℓ_2. Both the dropout and low-rank approaches are a priori competitive to foster transfer learning. Our final method uses a combination of both, as it enforces a hard low-rank constraint and uses dropout. This choice was motivated by a first experiment, summarized in Fig D. We compare three regularization variants by measuring the improvement due to hard low-rank constraints and the difference between dropout and ℓ_2. The three estimators use input dropout ($p = 0.25$). The first two estimators use ℓ_2 regularization. Dropout between layer 2 and 3 is initialized to $p = 0.75$ in the third estimators. The first estimator does not use a hard-rank constraint ($l = c = 545$), while others use $l = 128$.[4] We observe that forcing $\boldsymbol{V}$ to be low-rank is beneficial (0.7% mean accuracy gain, 72% experiments with net increase) in the absence of dropout, and that dropout regularization performs significantly better than low-rank inducing ℓ_2 penalties (2.7% mean accuracy gain, 79% experiments with net increase). This justifies using dropout regularization.

[4]The reported ℓ_2 accuracy gain is larger than its actual performance when λ is set with cross-validation, as we take the highest performing λ on the *test* sets. Symmetrically, we may slightly improve results by setting dropout rates using cross-validation—we choose not to, to avoid the fragility of cross-validation in neuro-imaging [22].

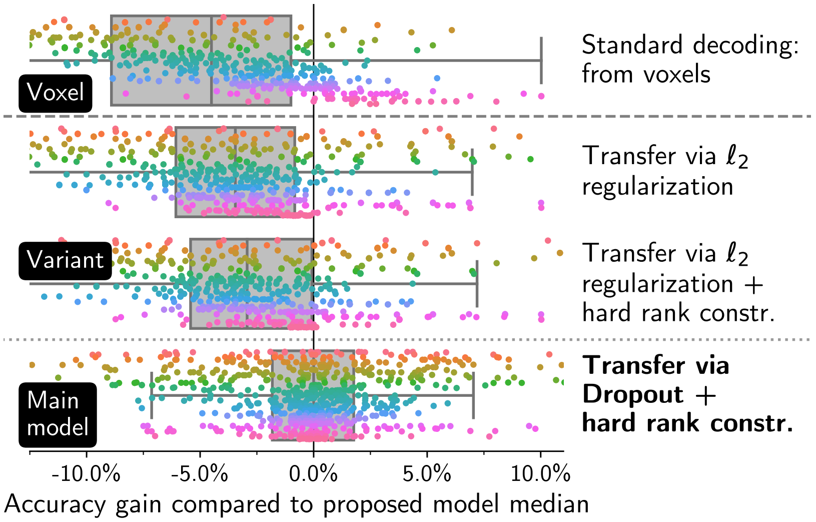

Fig D. Quantitative comparison of transfer inducing regularizations. Dropout with hard-rank constraints outperforms ℓ_2 regularization with and without hard-rank constraints. Box plots calculated over 20 random data half-split and all studies.

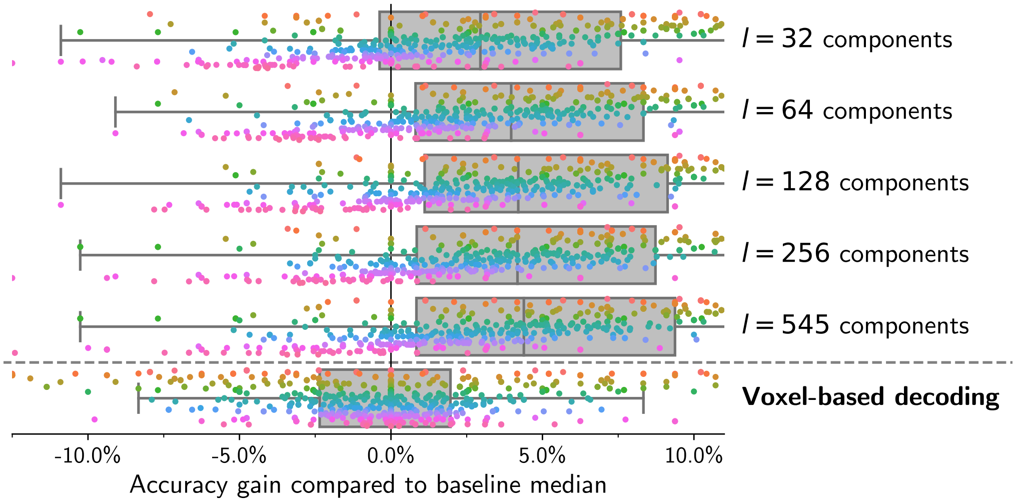

Fig E. Performance of multi-study decoding for varying second layer width l.

Low-rank constraints and second-layer width. With dropout, the performance of multi-study decoding varies with the size of the latent space l, as displayed in Fig E. The performance reaches a plateau at $l \approx 128$. Setting a high l results in more scattered networks, so that different but similar MSTONs may be recruited to decode the same psychological condition (see examples in Fig A). Choosing a low l leads to slightly worse performances but more interpretable components. We therefore use $l = 128$, as if offers the best performance/interpretability trade-off.

First-layer width. Some previous work [13] studies the impact of projecting brain signal onto k functional units, for varying k and different fMRI analysis tasks. The conclusion of this work applies here: setting a high k ensures the best performances. We use $k = 465$ grey-matter components extracted from 512 full-brain components due to constraints in training—higher k may be used in future work.

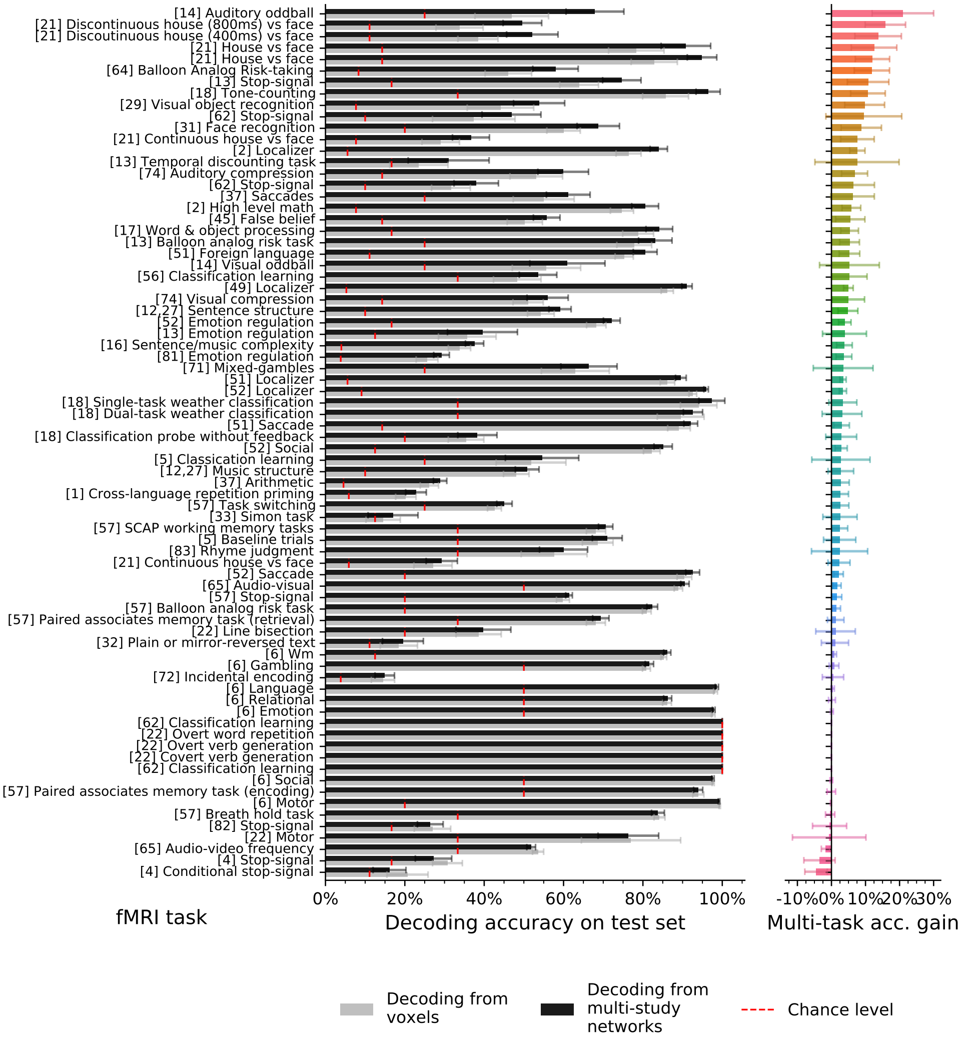

Fig F. Performance of multi-study multi-task decoding, versus single-study single-task decoding from resting-state functional units. Numbers are reported in Table B.

B.3 Multi-study multi-task decoding

We have validated the multi-study decoding approach in a *per-site* setting, in which each study defines a single decoding task. Some studies include different fMRI tasks: we can also use each of these tasks to define a single decoding problem, and perform *multi-study multi-task decoding.* To evaluate this approach, we use the task annotations from the 35 studies of our corpus and obtain 76 classification tasks to be solved simultaneously. We compare the performance of the three-layer model, versus single-task decoding from the resting-state functional units. We use the exact same architecture as for multi-study training.

Results are displayed in Fig F. Multi-task training brings an improvement for 62/76 tasks. Quantitatively, the mean improvement is lower than the one obtained for within-study decoding (+3.9% vs +5.8%). This was expected, as the average chance-level in within-task decoding is higher than in within-study decoding. Using multi-task or multi-site modelling should depend on the purpose of the study.

B.4 Interpretability incentives

A core feature of our approach is model interpretability. Three aspects allow to find cognitive meaningful task-optimized networks. First, the initial first layer, learned on resting-state data, coarsens the resolution of networks in a way adapted to typical brain signals. Second, we compute a consensus model, so that the task-optimized network loadings held in $\boldsymbol{L}$ are non-negative and interpretable. Third, we initialize the second-layer weights so that $\boldsymbol{L}_{\text{init}}\boldsymbol{D}$ corresponds to resting-state functional networks $\boldsymbol{D}_l$, coarser than $\boldsymbol{D}$. This initialization is used both during the training phase and the consensus phase.

Consensus model and resting-state initialization. In Fig H, we measure the quantitative effects of the two later factors on decoder accuracy. Learning a consensus model using sparse NMF is crucial for finding interpretable direction in the span of $\boldsymbol{L}$. Without this refinement, the directions we obtain are similar to the one displayed in Fig GA, and are less interpretable. Both the consensus phase and the resting-state initialization contributes positively to the model decoding performance (0.6% mean accuracy gain, 66% experiments with net increase). We attribute this improvement to an ensembling effect similar to the benefits of bagging [23], as the final model summarizes 100 training runs on the same data, with different random seeds, and to the fact that resting-state networks form a good prior for task-optimized network.

Qualitatively, we show examples of three components found without resting-state initialization in Fig GB. Two of those are scattered networks, that capture various connected components whose co-occurrence is not interpretable: those components are likely artifacts due to random initialization. Using resting-state initialization finds such networks much less frequently. It remains interesting to note that most of the components found without resting-state based prior bear cognitive meaning, similar to the third components displayed in Fig GB.

B.4.1 Effect of selecting grey-matter components

We project data onto a subset of 465 out of 512 functional networks learned on HCP resting-state data, selecting the networks that intersect with an anatomical grey-matter mask. This avoids finding MSTONs that are distributed or formed with non grey-matter regions. In Fig GC, we show that without those precautions, our model finds networks located in the white matter and the cerebro-spinal fluid zones. Quantitatively (Fig I),

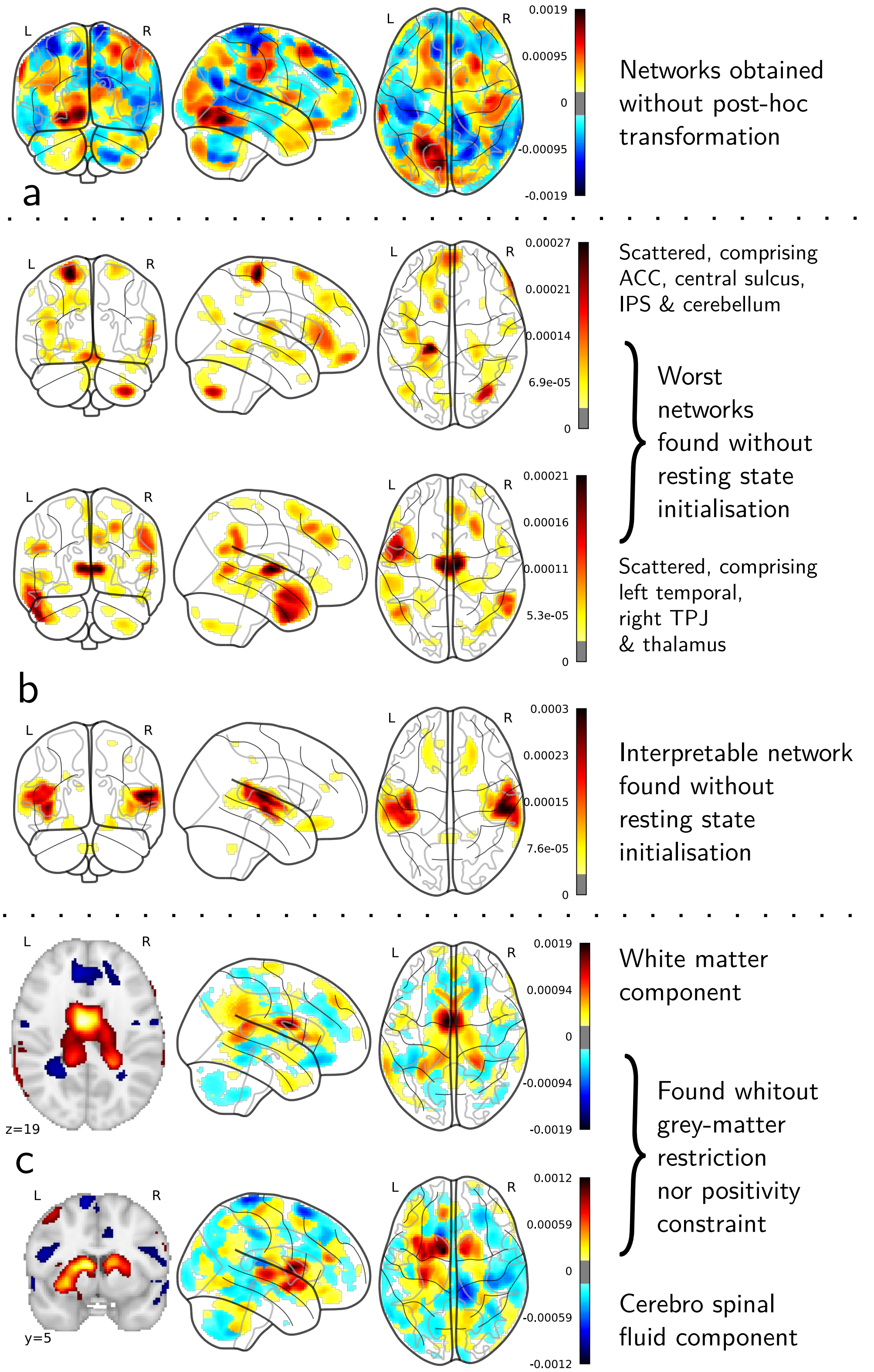

Fig G. Effects of components selection. Without post-hoc transformation **(A)** resting-state based initialization **(B)** and grey matter components selection **(C)**, some task-optimized networks may be hard to interpret or not relevant from a cognitive perspective.

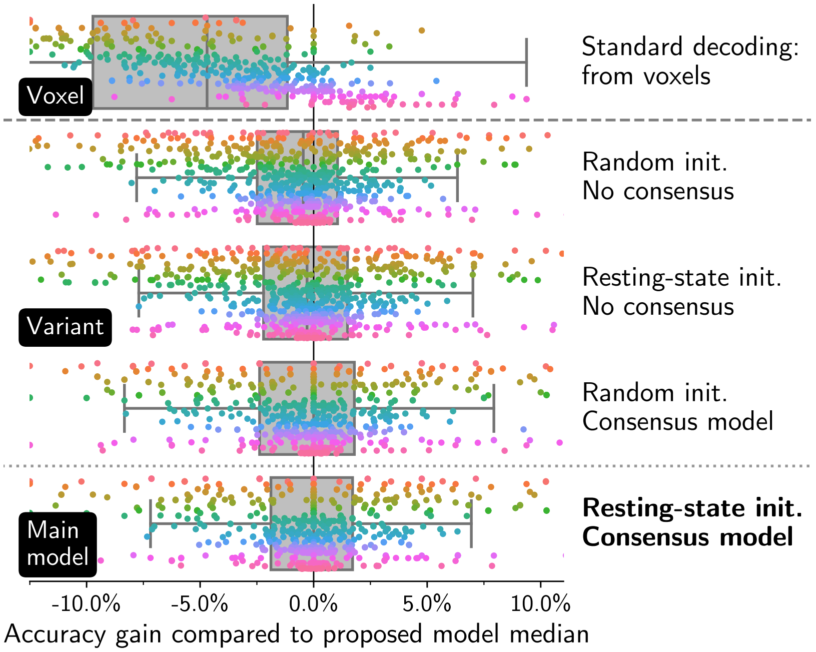

Fig H. Quantitative improvement linked to ensembling and resting-state initialization. Box plots calculated over 20 random data half-split and all studies.

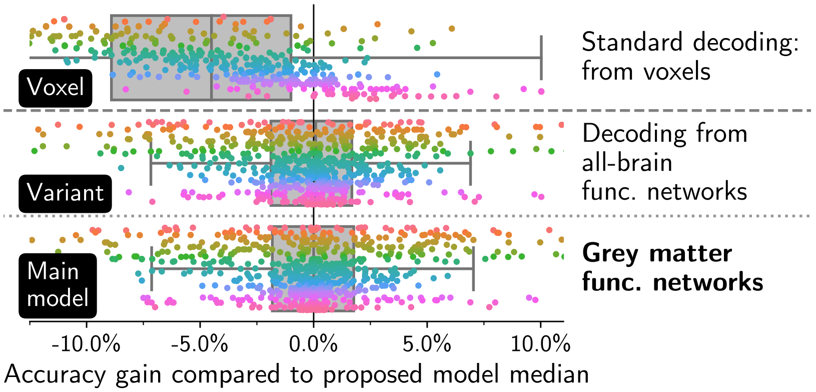

Fig I. Quantitative improvement linked to working with a grey-matter mask. Working with functional networks located in the grey matter only do not have a significant impact on performance. Box plots calculated over 20 random data half-split and all studies.

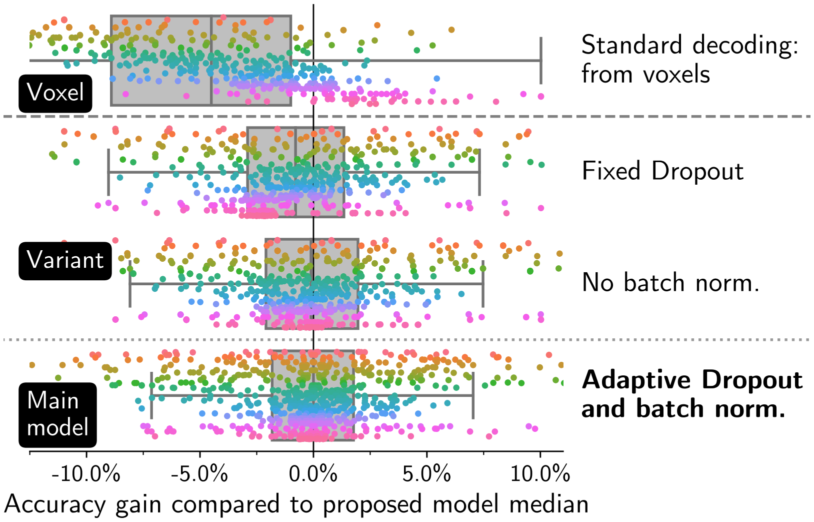

Fig J. Batch normalization and adaptive variational dropout both have a beneficial impact on classification accuracy of the final learned decoder. Box plots calculated over 20 random data half-split and all studies.

as expected, performing classification from grey-matter components only brings a non-significant performance loss (0.03% median accuracy gain).

B.5 Effect of variational dropout and batch normalization

We introduced variational dropout and batch normalization in the training procedure of our algorithm. Fig J shows that it is indeed beneficial. Variational dropout brings a mean accuracy gain of 0.7% (64% experiments with net increase) compared to binary dropout; batch normalization benefit is smaller but positive (0.1% mean accuracy gain, 55% experiments with net increase), and allows faster training—in line with its original purpose [12].

B.6 Stronger improvement for smaller studies

To verify the finding of Fig 3 and evaluate the impact of training-size on multi-study decoding, we perform the following experiment. We restrict the study corpus to studies with more than 30 subjects, train the three-layer model on 15 subjects from each study, and evaluate its performance on the remaining population. We repeat this experiment 20 times.

We report results in Fig K. Transfer learning is positive for all studies (mean accuracy gain +4.8%.). This includes studies with a large complete cohort, for which transfer learning is uneffective when considering all available subjects (e.g. HCP, Fig 2 and data from the UCLA consortium). The multi-study approach is therefore particularly efficient for studies with less than 30 subjects, that are still the most common in the literature.

B.7 Effect of decoding difficulty

We investigate how the difficulty of a given decoding task (provided by a single study) influences the performance improvement due to multi-study decoding. For this, we report in Fig L the same numbers as in Fig 2, sorting studies by their chance level: lower chance level means “harder” decoding tasks, as contrasts must be selected in

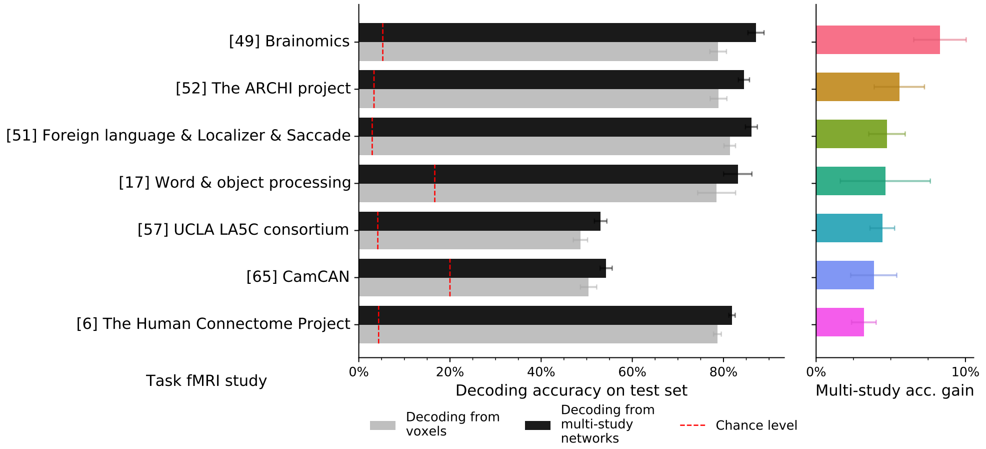

Fig K. Performance of multi-study decoding with 15 training subjects per study.

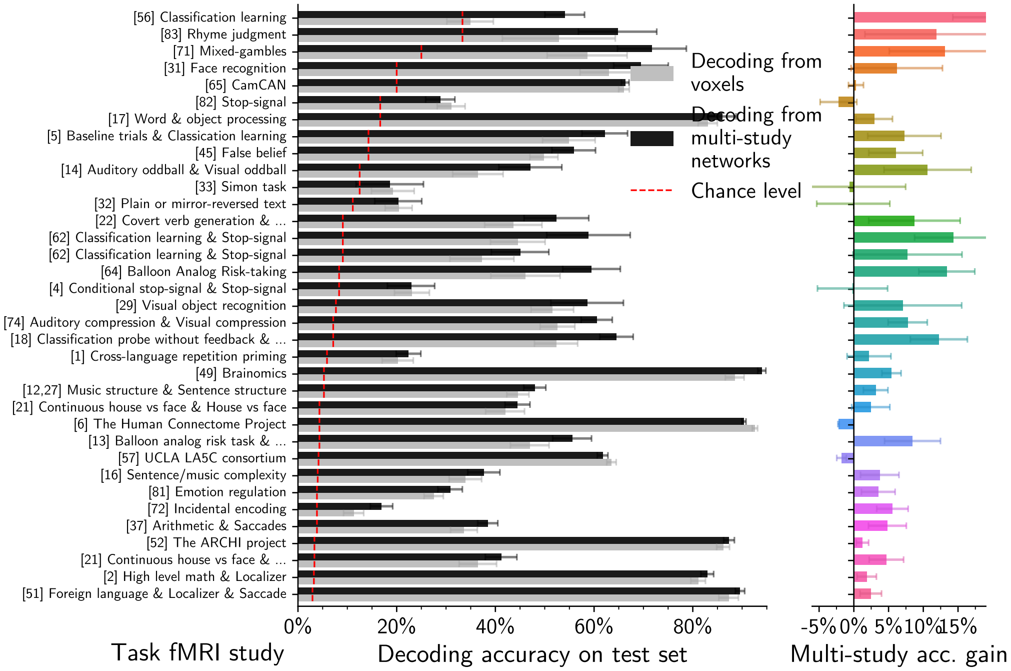

Fig L. Performance improvement of multi-study decoding vs voxel-level decoding, **sorted by the chance level** of the decoding task of each study.

larger sets. We observe a slight tendency of higher improvement for easier tasks, although no strong conclusion may be drawn.

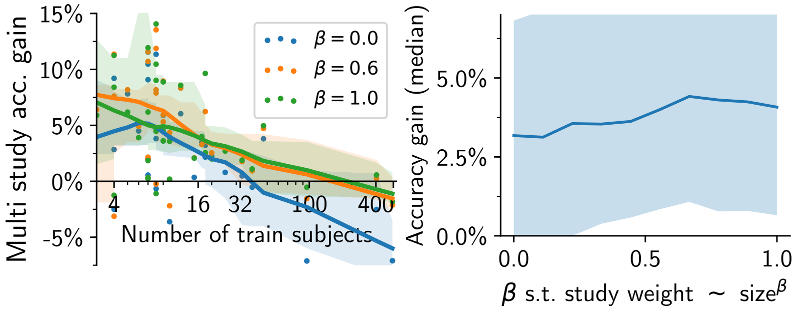

Fig M. Impact of changing the study weight in the joint objective. Giving more weight ($\beta \to 1$) to large studies prevents negative transfer learning but may reduce overall performance. Small studies should not be given too much weight ($\beta \to 0$), as this voids the benefits of jointly training over bigger studies. An intermediary $\beta = 0.6$ gives the best performances. Error bars calculated over 20 random data half-split and all studies.

B.8 Effect of study weights

Our model learns the second and third layer weights by solving

$$\min_{\substack{\boldsymbol{L}\in\mathbb{R}^{l\times k}\\ (\boldsymbol{U}^j,\boldsymbol{b}^j)_j}} - \sum_{j=1}^{N} \frac{(n^j)^\beta}{n^j} \sum_{i=1}^{n^j} \Big(l^j_{i,y_i}(\boldsymbol{U}^j, \boldsymbol{b}^j, \boldsymbol{L}) - \log(\sum_{k=1}^{c^j} \exp l^j_{i,k}(\boldsymbol{U}^j, \boldsymbol{b}^j, \boldsymbol{L}))\Big),$$

in which the many studies can be given various weights. At one extreme, we may consider that all studies of the corpus should be weighted the same, which amounts to setting $\beta = 0$ in (4). At the opposite, we can consider that each brain map from each study should have the same importance, which amounts to setting $\beta = 1$. As Fig MB shows, it is beneficial to set an intermediary β, typically $\beta = 0.6$. On the one hand, we want to give the smallest study of our corpus a non negligible importance; on the other hand, we want the large studies to remain more weighted than the smaller ones, as they should provide more accurate information. Our reweighting amounts to giving every study j an "effective sample size"

$$n^j_{\text{eff}} = \sum_{i=1}^{N} n^i \frac{{n^j}^\beta}{\sum_{i=1}^{N} {n^i}^\beta},$$

that is larger than the true sample size for smaller studies and smaller for larger studies. We observe on Fig MA that the negative transfer learning endured by large-study decoders such as HCP and LA5C reduces as these studies are given more weight ($\beta \to 1$). On the other hand, the performance on small datasets slightly reduces for $\beta > 0.6$. It also reduces for low β, hinting at the importance of using large studies for improving small studies decoding.

We thus have provided justifications for all the technical design choices made in training our decoding model: regularization, joint training, training refinements, choice of study weights.

B.9 Comparison with earlier work

We proposed a proof-of-concept, smaller-scale and harder to interpret multi-study decoding approach in [24]. This earlier work already relies on a three-layer linear model, with joint training of the second and third layer. Beyond its extended cognitive neuroscience point-of-view, the present work strongly improves the multi-study decoding methods and results.

Model interpretability. From a methodological point of view, [24] fail short of providing a principled way for interpreting results and extracting meaningful task-optimized networks, as those outlined in Fig 4. Their approach yields networks akin to Fig GA, which are not relevant from a cognitive perspective. A template-extracting approach that clusters the low-dimensional brain map representations is proposed; yet it remains exogenous to the model and does not perform convincingly. The consensus post-hoc transformation method we propose in this work addresses the issue of interpretability and finds cognitive directions that efficiently capture mental state information. As Fig B shows, these meaningful networks can be used as a cognitive atlas for improving decoding on newly acquired datasets, without joint training. Consensus through matrix factorization of the model weights also increases model performance (Fig B).

Architecture, constraints, training. The functional atlases used as a first-layer by [24] are smaller (up to 256 components) and not constrained to be non-negative. As we discovered, enforcing non-negativity of the first layer $\boldsymbol{D}$ and the second layer $\boldsymbol{L}$ (after ensembling) is crucial to interpret the prediction of the model. Using a larger functional atlas extracted from resting-state data ensures that no information is lost when reducing the dimension of brain maps. Initialization of the second-layer with resting-state information increases the model performance (Fig H), as well as the use of variational dropout [9] and batch normalization [12] (Fig J).

Data and validation. [24] pool only the results of 5 studies, which prevents the observation heavy transfer effects, and the extraction of broadly-valid cognitive directions. The present work validates the approach on 7 times more studies, proving that our multi-study approach is valid beyond proof-of-concept, and truly promising for the neuroscience community. To better explain the transfer of information across studies, we compare several transfer approaches (convex models, low-rank constraints, stochastic regularization: see Section B.2), and assess how classification maps are affected by the use of task-optimized network (Figs 6, 7 and C); this endeavor is missing in earlier work.

C Reproduction details and tables

In this last section, we detail our experiment pipeline, the numerical parameters needed for reproducing this study, and the sources from which we obtained our corpus of studies.

C.1 Software and parameters

We used *nilearn* [25] and *scikit-learn* [26] in our experiment pipelines, the stochastic solver from [14] to learn resting state dictionaries and *pytorch* [27] for model design and training. The *cogspaces* package that we have published provides the multi-scale resting-state dictionaries extracted from HCP, as those are costly to learn. It also provides the reduced representations of the data from the 35 studies we consider.

General cross-validation scheme. For every validation experiment and comparison, we perform 20 half-split of all data. Namely, we consider half of the subjects of every study for training, and test the decoder on the other half. As two studies [28] share subjects, we also ensure that no single subject appears in both the training and the test sets across studies.

Baseline parameter selection. We cross validate the λ parameter for the baseline multinomial regression classifiers, on a grid

$$\{10^i, i = \{-3, -2, -1, 0, 1, 2, 3\}\}.$$

Dropout rate. We use a dropout rate of $p = 0.25$ in between the first and second layer and initialize study-specific dropout rates with $p = 0.75$ in between the second-layer and third-layer classification heads (i.e., we set $\alpha = \frac{p}{1-p}$ in variational dropout).

Resting-state dictionaries. We obtain the 512-components and 128 components resting-state dictionaries by choosing λ on a grid

$$\{10^i, i = \{-5, -4, -3, -2, -1, 0, 1\}\},$$

so to obtain components that cover the whole brain with minimal overlap.

Consensus phase. We run the training procedure 100 times with different random seeds. We set $\lambda = 10^{-4}$, so as to obtain 80% sparsity. We tried $\lambda \in \{10^{-5}, 10^{-4}, 10^{-3}, 10^{-2}\}$. Higher sparsity leads to a slight decrease in performance, lower sparsity is softer on symmetry breaking, which may reduce interpretability. This parameter has little influence as long as the sparsity remains higher than 50%.

Word-clouds. In Fig 4, we form word-clouds associated with the k-th MSTON network $\boldsymbol{Dl}_k$ as follows. We compute the correlations between each classification map $\boldsymbol{w}_c$, associated with a condition c, and the network $\boldsymbol{Dl}_k$ as

$$d_{k,c} = \frac{\langle \boldsymbol{Dl}_k, \boldsymbol{w}_c \rangle}{\|\boldsymbol{Dl}_k\|_2 \|\boldsymbol{w}_c\|_2}.$$

We then show the 20 contrast names with highest correlation values—this corresponds to the contrasts whose likelihood increases the most when the input data is pushed in the direction of $\boldsymbol{Dl}_k$. The height of the contrast name c in the word-cloud reflects the rank of the contrast in the sorted values $(d_{k,c})_c$ and the value $d_{k,c}$, using heuristics from the Python *word_cloud* package (`https://github.com/amueller/word_cloud`).

C.2 Validation metrics

We used two metrics to measure the performance of our models. To compare per-study decoding accuracy, we use the multi-class accuracy, defined as

$$a^j = \frac{\#\{i \in [c^j n^j], \hat{y}_i^j = y_i^j\}}{c^j n^j},$$

for study j, where $(\hat{y}_i^j)_{i\in[c^j n^j]}$ and $(y_i^j)_{i\in[c^j n^j]}$ encodes the predicted and ground-truth contrasts, respectively. Box plots presented in Fig 2 and Figs D–I reports the median and 25%, 75% quantiles of

$$\{a_r^j - \bar{a}_0^j, j \in [1, \dots, N], r \in [1, 2, \cdots, 20]\},$$

where r is the half-split run index and $\bar{a}_0^j$ is the median accuracy obtained for study j over 20 half-split.

We use balanced accuracy to measure the performance relative to a single contrast $y \in [1, \dots, c^j]$. It corresponds to the average of 1) the proportion of z-maps being correctly classified into y and 2) the proportion of z-maps being correctly classified into other classes. This metric has the advantage of being comparable across studies, as its chance level is always 50% no matter the number of contrasts in the study. We recall that the balanced accuracy b_y^i for study j and contrast y in $[1, \dots, c^j]$ is defined as

$$b_y^j \triangleq \frac{1}{2}\Big(\frac{n^j}{\#\{i \in [1,2,\dots,c^j n^j], \hat{y}_i^j = y\}} + \frac{n^j(c^j-1)}{\#\{i \in [1,2,\dots,c^j n^j], \hat{y}_i^j \neq y\}}\Big).$$

C.3 Quantitative results per study, task and contrast

We report the accuracies displayed in Fig 2 in Table A (multi-study decoding), and the ones displayed in Fig F in Table B (multi-task decoding). We report the list of all contrasts used in this paper in Table C, as provided by the authors of each study. We report the associated balanced-accuracy when performing multi-study decoding, i.e. when we predict each contrasts among the set of all contrasts of a given study.

Table B. Accuracies per task in multi-site multi task decoding.

Study	Task	Chance level	Multi-task accuracy	Single-task accuracy	Accuracy gain
[29]	High level math	8%	$81 \pm 3\%$	$75 \pm 3\%$	$6 \pm 3\%$
	Localizer	6%	$84 \pm 2\%$	$76 \pm 3\%$	$8 \pm 2\%$
[30]	Emotion regulation	17%	$72 \pm 2\%$	$68 \pm 2\%$	$4 \pm 2\%$
	Localizer	9%	$96 \pm 1\%$	$93 \pm 1\%$	$3 \pm 1\%$
	Saccade	20%	$93 \pm 2\%$	$90 \pm 2\%$	$2 \pm 1\%$
	Social	12%	$85 \pm 2\%$	$82 \pm 2\%$	$3 \pm 2\%$
[15]	Localizer	5%	$91 \pm 1\%$	$86 \pm 2\%$	$5 \pm 1\%$
[31]	Audio-video frequency	33%	$52 \pm 1\%$	$54 \pm 1\%$	$-2 \pm 1\%$
	Audio-visual	50%	$91 \pm 1\%$	$89 \pm 1\%$	$2 \pm 1\%$
[32, 33]	Music structure	10%	$51 \pm 3\%$	$48 \pm 3\%$	$3 \pm 4\%$
	Sentence structure	10%	$59 \pm 3\%$	$54 \pm 3\%$	$5 \pm 3\%$
[34]	Sentence/music complexity	4%	$38 \pm 2\%$	$34 \pm 3\%$	$4 \pm 2\%$
[35]	Balloon Analog Risk-taking	8%	$58 \pm 6\%$	$46 \pm 6\%$	$12 \pm 5\%$
[36]	Baseline trials	33%	$71 \pm 4\%$	$69 \pm 4\%$	$2 \pm 5\%$

Continued on next page

Study	Task	Chance level	Multi-task accuracy	Single-task accuracy	Accuracy gain
	Classication learning	25%	$55 \pm 9\%$	$52 \pm 9\%$	$3 \pm 9\%$
[37]	Rhyme judgment	33%	$60 \pm 6\%$	$58 \pm 8\%$	$2 \pm 8\%$
[38]	Mixed-gambles	25%	$66 \pm 7\%$	$63 \pm 9\%$	$3 \pm 9\%$
[39]	Plain or mirror-reversed text	11%	$20 \pm 5\%$	$18 \pm 5\%$	$1 \pm 4\%$
[40]	Stop-signal	17%	$26 \pm 3\%$	$27 \pm 5\%$	$-1 \pm 5\%$
[41]	Conditional stop-signal	11%	$16 \pm 4\%$	$21 \pm 5\%$	$-5 \pm 3\%$
	Stop-signal	17%	$27 \pm 5\%$	$31 \pm 4\%$	$-4 \pm 5\%$
[42]	Balloon analog risk task	25%	$83 \pm 4\%$	$78 \pm 4\%$	$5 \pm 3\%$
	Emotion regulation	12%	$40 \pm 9\%$	$36 \pm 7\%$	$4 \pm 6\%$
	Stop-signal	17%	$75 \pm 5\%$	$64 \pm 5\%$	$11 \pm 6\%$
	Temporal discounting task	17%	$31 \pm 10\%$	$24 \pm 7\%$	$8 \pm 12\%$
[43]	Classification probe without feedback	20%	$38 \pm 5\%$	$35 \pm 4\%$	$3 \pm 5\%$
	Dual-task weather classification	33%	$93 \pm 2\%$	$90 \pm 6\%$	$3 \pm 6\%$
	Single-task weather classification	33%	$97 \pm 3\%$	$94 \pm 5\%$	$3 \pm 4\%$
	Tone-counting	33%	$96 \pm 3\%$	$86 \pm 6\%$	$11 \pm 5\%$
[28]	Classification learning	100%	$100 \pm 0\%$	$100 \pm 0\%$	$0 \pm 0\%$
	Stop-signal	10%	$38 \pm 6\%$	$32 \pm 5\%$	$6 \pm 6\%$
	Classification learning	100%	$100 \pm 0\%$	$100 \pm 0\%$	$0 \pm 0\%$
	Stop-signal	10%	$47 \pm 7\%$	$37 \pm 10\%$	$9 \pm 11\%$
[44]	Cross-language repetition priming	6%	$23 \pm 3\%$	$20 \pm 3\%$	$3 \pm 2\%$
[45]	Classification learning	33%	$54 \pm 5\%$	$48 \pm 6\%$	$5 \pm 5\%$
[46]	Simon task	12%	$17 \pm 6\%$	$15 \pm 4\%$	$2 \pm 5\%$
[47]	Visual object recognition	8%	$54 \pm 6\%$	$44 \pm 8\%$	$10 \pm 6\%$
[48]	Word & object processing	17%	$84 \pm 3\%$	$79 \pm 4\%$	$5 \pm 3\%$
[49]	Emotion regulation	4%	$29 \pm 2\%$	$26 \pm 3\%$	$4 \pm 2\%$
[50]	False belief	14%	$56 \pm 3\%$	$50 \pm 4\%$	$6 \pm 4\%$
[51]	Incidental encoding	4%	$15 \pm 2\%$	$14 \pm 3\%$	$0 \pm 3\%$
[52]	Covert verb generation	100%	$100 \pm 0\%$	$100 \pm 0\%$	$0 \pm 0\%$
	Line bisection	20%	$40 \pm 7\%$	$39 \pm 6\%$	$1 \pm 6\%$
	Motor	33%	$76 \pm 8\%$	$77 \pm 13\%$	$-1 \pm 11\%$
	Overt verb generation	100%	$100 \pm 0\%$	$100 \pm 0\%$	$0 \pm 0\%$
	Overt word repetition	100%	$100 \pm 0\%$	$100 \pm 0\%$	$0 \pm 0\%$
[53]	Auditory oddball	25%	$68 \pm 7\%$	$47 \pm 9\%$	$21 \pm 9\%$
	Visual oddball	25%	$61 \pm 9\%$	$56 \pm 9\%$	$5 \pm 9\%$
[54]	Continuous house vs face	8%	$37 \pm 5\%$	$29 \pm 5\%$	$8 \pm 5\%$
	Discontinuous house (800ms) vs face	11%	$50 \pm 5\%$	$34 \pm 6\%$	$16 \pm 6\%$
	Discoutinuous house (400ms) vs face	11%	$52 \pm 6\%$	$38 \pm 5\%$	$14 \pm 7\%$
	House vs face	14%	$91 \pm 6\%$	$78 \pm 7\%$	$13 \pm 7\%$
	Continuous house vs face	6%	$29 \pm 4\%$	$27 \pm 5\%$	$2 \pm 3\%$
	House vs face	14%	$95 \pm 4\%$	$83 \pm 6\%$	$12 \pm 5\%$
[55]	Emotion	50%	$98 \pm 0\%$	$98 \pm 0\%$	$0 \pm 0\%$
	Gambling	50%	$82 \pm 1\%$	$81 \pm 1\%$	$1 \pm 1\%$
	Language	50%	$99 \pm 0\%$	$98 \pm 0\%$	$0 \pm 1\%$
	Motor	20%	$99 \pm 0\%$	$99 \pm 0\%$	$-0 \pm 0\%$
	Relational	50%	$86 \pm 1\%$	$86 \pm 1\%$	$0 \pm 1\%$
	Social	50%	$98 \pm 0\%$	$98 \pm 0\%$	$-0 \pm 0\%$
	Wm	12%	$86 \pm 1\%$	$85 \pm 1\%$	$1 \pm 1\%$
[56]	Face recognition	20%	$69 \pm 5\%$	$60 \pm 4\%$	$9 \pm 6\%$
[57]	Arithmetic	5%	$29 \pm 2\%$	$26 \pm 2\%$	$3 \pm 3\%$
	Saccades	25%	$61 \pm 6\%$	$55 \pm 8\%$	$6 \pm 6\%$
[58]	Balloon analog risk task	20%	$82 \pm 1\%$	$81 \pm 1\%$	$1 \pm 1\%$
	Breath hold task	33%	$84 \pm 2\%$	$84 \pm 1\%$	$-0 \pm 1\%$
	Paired associates memory task (encoding)	50%	$94 \pm 1\%$	$94 \pm 1\%$	$-0 \pm 1\%$

Continued on next page

Study	Task	Chance level	Multi-task accuracy	Single-task accuracy	Accuracy gain
	Paired associates memory task (retrieval)	33%	$69 \pm 2\%$	$68 \pm 2\%$	$1 \pm 2\%$
	SCAP working memory tasks	33%	$71 \pm 2\%$	$68 \pm 2\%$	$2 \pm 2\%$
	Stop-signal	20%	$61 \pm 1\%$	$60 \pm 2\%$	$2 \pm 1\%$
	Task switching	25%	$45 \pm 2\%$	$43 \pm 2\%$	$3 \pm 3\%$
[59]	Foreign language	11%	$81 \pm 3\%$	$75 \pm 2\%$	$5 \pm 3\%$
	Localizer	6%	$90 \pm 1\%$	$86 \pm 2\%$	$3 \pm 1\%$
	Saccade	14%	$92 \pm 2\%$	$89 \pm 3\%$	$3 \pm 2\%$
[60]	Auditory compression	14%	$60 \pm 6\%$	$53 \pm 7\%$	$7 \pm 4\%$
	Visual compression	14%	$56 \pm 5\%$	$51 \pm 4\%$	$5 \pm 5\%$

Table C. List of all contrasts used in this paper, per study of origin and task.

Study	Task	Contrast	Multi-study B-acc	Voxel-level B-acc	B-acc gain
[29]	High level math	body vs baseline	$90 \pm 5\%$	$90 \pm 5\%$	$1 \pm 5\%$
		body vs checkerboard	$94 \pm 5\%$	$97 \pm 3\%$	$-3 \pm 4\%$
		checkerboard vs baseline	$88 \pm 4\%$	$89 \pm 5\%$	$-1 \pm 5\%$
		equation vs baseline	$83 \pm 4\%$	$84 \pm 5\%$	$-1 \pm 4\%$
		equation vs number	$97 \pm 2\%$	$96 \pm 3\%$	$2 \pm 3\%$
		face vs baseline	$90 \pm 5\%$	$92 \pm 3\%$	$-2 \pm 4\%$
		face vs house	$100 \pm 0\%$	$98 \pm 3\%$	$2 \pm 3\%$
		house vs baseline	$88 \pm 6\%$	$88 \pm 8\%$	$0 \pm 7\%$
		number vs baseline	$86 \pm 5\%$	$89 \pm 6\%$	$-3 \pm 6\%$
		number vs word	$95 \pm 4\%$	$88 \pm 6\%$	$7 \pm 5\%$
		tool vs baseline	$83 \pm 6\%$	$84 \pm 8\%$	$-1 \pm 8\%$
		tool vs checkerboard	$90 \pm 5\%$	$86 \pm 5\%$	$3 \pm 5\%$
		word vs baseline	$85 \pm 8\%$	$81 \pm 6\%$	$3 \pm 6\%$
	Localizer	auditory calculation vs baseline	$95 \pm 3\%$	$93 \pm 2\%$	$2 \pm 2\%$
		auditory calculation vs sentences	$72 \pm 8\%$	$71 \pm 12\%$	$2 \pm 16\%$
		auditory left motor vs baseline	$98 \pm 2\%$	$96 \pm 2\%$	$2 \pm 2\%$
		auditory motor vs sentences	$94 \pm 3\%$	$91 \pm 5\%$	$3 \pm 4\%$
		auditory right motor vs baseline	$98 \pm 3\%$	$93 \pm 3\%$	$4 \pm 5\%$
		auditory right vs left motor	$80 \pm 5\%$	$80 \pm 6\%$	$-1 \pm 6\%$
		auditory sentences vs baseline	$100 \pm 0\%$	$99 \pm 2\%$	$1 \pm 2\%$
		horizontal checkerboard vs baseline	$95 \pm 3\%$	$98 \pm 2\%$	$-2 \pm 3\%$
		horizontal vs vertical checkerboard	$92 \pm 3\%$	$95 \pm 3\%$	$-3 \pm 5\%$
		vertical checkerboard vs baseline	$92 \pm 4\%$	$98 \pm 2\%$	$-6 \pm 5\%$
		visual calculation vs baseline	$96 \pm 3\%$	$93 \pm 3\%$	$3 \pm 3\%$
		visual calculation vs sentences	$70 \pm 9\%$	$67 \pm 10\%$	$4 \pm 14\%$
		visual left motor vs baseline	$99 \pm 2\%$	$99 \pm 2\%$	$0 \pm 1\%$
		visual motor vs sentences	$99 \pm 2\%$	$96 \pm 3\%$	$3 \pm 3\%$
		visual right motor vs baseline	$97 \pm 3\%$	$95 \pm 3\%$	$1 \pm 3\%$
		visual right vs left motor	$82 \pm 7\%$	$77 \pm 7\%$	$5 \pm 6\%$
		visual sentences vs baseline	$98 \pm 2\%$	$96 \pm 3\%$	$2 \pm 3\%$

Continued on next page

Study	Task	Contrast	Multi-study B-acc	Voxel-level B-acc	B-acc gain
		visual sentences vs checker-board	100 ± 0%	97 ± 2%	2 ± 2%
[30]	Emotion regulation	expression control	81 ± 4%	83 ± 4%	−1 ± 4%
		expression intention	96 ± 1%	94 ± 2%	2 ± 1%
		expression sex	84 ± 4%	85 ± 4%	−1 ± 3%
		face control	79 ± 4%	78 ± 4%	1 ± 5%
		face sex	81 ± 2%	81 ± 4%	0 ± 4%
		face trusty	90 ± 2%	89 ± 3%	2 ± 2%
	Localizer	audio	97 ± 2%	93 ± 4%	5 ± 3%
		calculaudio	97 ± 2%	96 ± 2%	2 ± 1%
		calculvideo	97 ± 2%	94 ± 2%	2 ± 3%
		clicdaudio	98 ± 1%	98 ± 1%	0 ± 1%
		clicdvideo	98 ± 1%	98 ± 1%	1 ± 1%
		clicgaudio	98 ± 1%	98 ± 2%	1 ± 2%
		clicgvideo	98 ± 1%	96 ± 2%	2 ± 1%
		computation	97 ± 2%	96 ± 2%	1 ± 2%
		damier h	97 ± 2%	98 ± 1%	−1 ± 2%
		damier v	98 ± 1%	99 ± 1%	−1 ± 1%
		motor-cognitive	100 ± 0%	100 ± 0%	0 ± 0%
	Saccade	object grasp	97 ± 2%	95 ± 2%	1 ± 2%
		object orientation	94 ± 2%	92 ± 3%	2 ± 3%
		rotation hand	97 ± 1%	96 ± 2%	0 ± 1%
		rotation side	95 ± 1%	94 ± 2%	1 ± 2%
		saccade	98 ± 1%	96 ± 2%	2 ± 2%
	Social	false belief audio	98 ± 1%	97 ± 1%	1 ± 1%
		false belief video	92 ± 3%	91 ± 3%	1 ± 3%
		mecanistic audio	96 ± 3%	94 ± 3%	2 ± 2%
		mecanistic video	88 ± 2%	90 ± 3%	−2 ± 2%
		non speech	92 ± 3%	93 ± 3%	−1 ± 3%
		speech	90 ± 4%	92 ± 3%	−2 ± 2%
		triangle intention	90 ± 3%	89 ± 3%	0 ± 2%
		triangle random	91 ± 3%	92 ± 2%	−1 ± 2%
[15]	Localizer	auditory calculation	99 ± 1%	99 ± 0%	0 ± 1%
		auditory processing	100 ± 0%	93 ± 5%	7 ± 5%
		auditory sentences	98 ± 1%	97 ± 2%	1 ± 1%
		auditory&visual calculation	97 ± 2%	93 ± 3%	5 ± 3%
		auditory&visual sentences	95 ± 2%	90 ± 3%	5 ± 3%
		checkerboard	90 ± 3%	86 ± 3%	4 ± 2%
		effects of interest	100 ± 0%	85 ± 4%	14 ± 4%
		horizontal checkerboard	89 ± 3%	93 ± 2%	−4 ± 2%
		left auditory click	99 ± 1%	98 ± 1%	0 ± 1%
		left auditory&visual click	97 ± 2%	93 ± 3%	4 ± 3%
		left visual click	99 ± 1%	98 ± 2%	2 ± 2%
		motor	97 ± 1%	91 ± 3%	5 ± 3%
		right auditory click	98 ± 1%	96 ± 2%	2 ± 2%
		right auditory&visual click	95 ± 3%	93 ± 4%	2 ± 3%
		right visual click	98 ± 1%	96 ± 2%	2 ± 1%
		vertical checkerboard	95 ± 2%	98 ± 1%	−3 ± 1%
		visual calculation	97 ± 1%	97 ± 2%	0 ± 1%
		visual processing	99 ± 1%	93 ± 3%	6 ± 3%
		visual sentences	97 ± 2%	97 ± 2%	1 ± 3%
[31]	Audio-video frequency	audvid1200	75 ± 1%	75 ± 1%	0 ± 1%
		audvid300	70 ± 1%	71 ± 1%	−0 ± 2%
		audvid600	66 ± 1%	67 ± 1%	−1 ± 1%
	Audio-visual	audonly	91 ± 1%	89 ± 1%	1 ± 1%
		vidonly	93 ± 1%	92 ± 1%	1 ± 1%

Continued on next page

Study	Task	Contrast	Multi-study B-acc	Voxel-level B-acc	B-acc gain
[32, 33]	Music structure	c01 c02 vs c16 c08 music	$75 \pm 5\%$	$72 \pm 6\%$	$3 \pm 7\%$
		c01 music vs baseline	$57 \pm 4\%$	$56 \pm 4\%$	$1 \pm 3\%$
		c02 music vs baseline	$58 \pm 3\%$	$57 \pm 4\%$	$1 \pm 5\%$
		c04 music vs baseline	$54 \pm 3\%$	$54 \pm 4\%$	$0 \pm 4\%$
		c08 music vs baseline	$55 \pm 4\%$	$57 \pm 4\%$	$-2 \pm 4\%$
		c16 c08 music vs motor	$89 \pm 6\%$	$85 \pm 6\%$	$4 \pm 4\%$
		c16 c08 vs c01 c02 music	$75 \pm 5\%$	$71 \pm 5\%$	$4 \pm 6\%$
		c16 music vs baseline	$55 \pm 4\%$	$57 \pm 6\%$	$-3 \pm 3\%$
		motor vs baseline	$91 \pm 2\%$	$86 \pm 3\%$	$5 \pm 2\%$
		motor vs c16 c08 music	$86 \pm 6\%$	$86 \pm 6\%$	$-0 \pm 3\%$
	Sentence structure	c01 c02 vs c16 c08 language	$87 \pm 5\%$	$85 \pm 5\%$	$1 \pm 3\%$
		c01 language vs baseline	$61 \pm 6\%$	$62 \pm 5\%$	$-1 \pm 3\%$
		c02 language vs baseline	$63 \pm 6\%$	$60 \pm 5\%$	$3 \pm 6\%$
		c04 language vs baseline	$59 \pm 5\%$	$60 \pm 6\%$	$-1 \pm 6\%$
		c08 language vs baseline	$60 \pm 4\%$	$57 \pm 5\%$	$3 \pm 6\%$
		c16 c08 language vs motor	$86 \pm 5\%$	$84 \pm 4\%$	$2 \pm 4\%$
		c16 c08 vs c01 c02 language	$88 \pm 4\%$	$85 \pm 5\%$	$3 \pm 4\%$
		c16 language vs baseline	$74 \pm 4\%$	$72 \pm 5\%$	$3 \pm 4\%$
		motor vs baseline	$91 \pm 2\%$	$86 \pm 3\%$	$5 \pm 2\%$
		motor vs c16 c08 language	$85 \pm 6\%$	$84 \pm 6\%$	$1 \pm 5\%$
[34]	Sentence/music complexity	c01 c02 vs c12 c06	$90 \pm 9\%$	$81 \pm 11\%$	$8 \pm 7\%$
		c01 vs baseline	$57 \pm 6\%$	$55 \pm 5\%$	$2 \pm 6\%$
		c02 vs baseline	$56 \pm 6\%$	$54 \pm 7\%$	$2 \pm 6\%$
		c03 vs baseline	$53 \pm 4\%$	$50 \pm 3\%$	$3 \pm 4\%$
		c04 vs baseline	$54 \pm 5\%$	$54 \pm 4\%$	$0 \pm 6\%$
		c06 vs baseline	$62 \pm 6\%$	$58 \pm 6\%$	$4 \pm 6\%$
		c12 c06 vs c01 c02	$90 \pm 8\%$	$80 \pm 10\%$	$10 \pm 7\%$
		c12 c06 vs motor	$91 \pm 7\%$	$88 \pm 9\%$	$3 \pm 6\%$
		c12 vs baseline	$74 \pm 9\%$	$67 \pm 8\%$	$7 \pm 8\%$
		motor vs baseline	$98 \pm 2\%$	$99 \pm 1\%$	$-0 \pm 2\%$
		motor vs c12 c06	$89 \pm 9\%$	$88 \pm 8\%$	$1 \pm 6\%$
		nc3 vs baseline	$53 \pm 4\%$	$51 \pm 4\%$	$1 \pm 5\%$
		nc4 vs baseline	$56 \pm 6\%$	$57 \pm 9\%$	$-1 \pm 6\%$
		pseudo c01 c02 vs c12 c06	$71 \pm 10\%$	$73 \pm 10\%$	$-2 \pm 9\%$
		pseudo c01 vs baseline	$55 \pm 5\%$	$55 \pm 4\%$	$0 \pm 5\%$
		pseudo c02 vs baseline	$52 \pm 3\%$	$52 \pm 2\%$	$0 \pm 4\%$
		pseudo c03 vs baseline	$50 \pm 3\%$	$50 \pm 1\%$	$1 \pm 3\%$
		pseudo c04 vs baseline	$53 \pm 4\%$	$52 \pm 3\%$	$1 \pm 5\%$
		pseudo c06 vs baseline	$52 \pm 3\%$	$53 \pm 3\%$	$-1 \pm 4\%$
		pseudo c12 c06 vs c01 c02	$72 \pm 11\%$	$73 \pm 10\%$	$-1 \pm 9\%$
		pseudo c12 c06 vs motor	$90 \pm 9\%$	$84 \pm 9\%$	$6 \pm 7\%$
		pseudo c12 vs baseline	$62 \pm 7\%$	$60 \pm 5\%$	$2 \pm 5\%$
		pseudo motor vs c12 c06	$92 \pm 8\%$	$87 \pm 8\%$	$5 \pm 7\%$
		pseudo nc3 vs baseline	$54 \pm 6\%$	$54 \pm 4\%$	$1 \pm 7\%$
		pseudo nc4 vs baseline	$52 \pm 2\%$	$52 \pm 4\%$	$-0 \pm 3\%$
[35]	Balloon Analog Risk-taking	cash fixed vs baseline	$72 \pm 7\%$	$66 \pm 6\%$	$6 \pm 8\%$
		control fixed vs baseline	$77 \pm 7\%$	$60 \pm 6\%$	$17 \pm 6\%$
		control pumps demean vs baseline	$80 \pm 8\%$	$74 \pm 9\%$	$6 \pm 9\%$
		ctrl demean vs pumps demean	$88 \pm 7\%$	$86 \pm 7\%$	$2 \pm 7\%$
		ctrl fixed vs pumps fixed	$86 \pm 5\%$	$76 \pm 7\%$	$10 \pm 7\%$
		ctrl realrt vs pumps realrt	$73 \pm 9\%$	$66 \pm 9\%$	$7 \pm 8\%$
		explode fixed vs baseline	$83 \pm 6\%$	$81 \pm 7\%$	$2 \pm 7\%$
		pumps demean vs baseline	$82 \pm 8\%$	$71 \pm 9\%$	$11 \pm 8\%$
		pumps demean vs ctrl demean	$73 \pm 8\%$	$71 \pm 12\%$	$2 \pm 8\%$

Continued on next page

Study	Task	Contrast	Multi-study B-acc	Voxel-level B-acc	B-acc gain
		pumps fixed vs baseline	$75 \pm 8\%$	$72 \pm 8\%$	$3 \pm 8\%$
		pumps fixed vs ctrl fixed	$78 \pm 6\%$	$65 \pm 8\%$	$13 \pm 9\%$
		pumps realrt vs ctrl realrt	$68 \pm 8\%$	$60 \pm 7\%$	$8 \pm 10\%$
[36]	Baseline trials	feedback vs baseline	$89 \pm 3\%$	$84 \pm 6\%$	$5 \pm 5\%$
		task vs baseline	$78 \pm 6\%$	$76 \pm 5\%$	$2 \pm 7\%$
		task vs feedback	$76 \pm 6\%$	$70 \pm 8\%$	$5 \pm 6\%$
	Classication learning	deterministic classification vs baseline	$69 \pm 8\%$	$70 \pm 9\%$	$-1 \pm 9\%$
		deterministic vs probabilistic classification	$76 \pm 8\%$	$71 \pm 11\%$	$5 \pm 13\%$
		probabilistic classification vs baseline	$73 \pm 11\%$	$63 \pm 6\%$	$10 \pm 10\%$
		probabilistic vs deterministic classification	$75 \pm 6\%$	$72 \pm 7\%$	$3 \pm 7\%$
[37]	Rhyme judgment	pseudoword vs baseline	$68 \pm 7\%$	$60 \pm 10\%$	$8 \pm 9\%$
		word vs baseline	$64 \pm 8\%$	$58 \pm 10\%$	$6 \pm 10\%$
		word vs pseudoword	$89 \pm 9\%$	$76 \pm 11\%$	$13 \pm 11\%$
[38]	Mixed-gambles	distance from indifference vs baseline	$77 \pm 6\%$	$67 \pm 9\%$	$10 \pm 10\%$
		parametric gain vs baseline	$80 \pm 9\%$	$71 \pm 10\%$	$9 \pm 9\%$
		parametric loss vs baseline	$75 \pm 10\%$	$64 \pm 8\%$	$11 \pm 10\%$
		task vs baseline	$93 \pm 4\%$	$88 \pm 6\%$	$5 \pm 7\%$
[39]	Plain or mirror-reversed text	junk	$58 \pm 6\%$	$55 \pm 5\%$	$3 \pm 8\%$
		mr non-switch vs baseline	$53 \pm 6\%$	$51 \pm 5\%$	$2 \pm 6\%$
		mr switch vs baseline	$50 \pm 3\%$	$50 \pm 2\%$	$0 \pm 3\%$
		mr vs plain	$52 \pm 7\%$	$55 \pm 9\%$	$-4 \pm 11\%$
		pl non-switch vs baseline	$55 \pm 6\%$	$60 \pm 8\%$	$-5 \pm 8\%$
		pl switch vs baseline	$50 \pm 5\%$	$50 \pm 5\%$	$-1 \pm 5\%$
		switch vs nonswitch	$51 \pm 4\%$	$51 \pm 6\%$	$-1 \pm 7\%$
		switch vs nonswitch mronly	$68 \pm 11\%$	$65 \pm 7\%$	$3 \pm 13\%$
		switch vs nonswitch plainonly	$61 \pm 8\%$	$59 \pm 7\%$	$2 \pm 12\%$
[40]	Stop-signal	failed stop vs baseline	$57 \pm 4\%$	$53 \pm 3\%$	$4 \pm 3\%$
		failed vs successful stop	$60 \pm 5\%$	$66 \pm 5\%$	$-6 \pm 5\%$
		go vs baseline	$61 \pm 3\%$	$65 \pm 4\%$	$-3 \pm 4\%$
		junk vs baseline	$52 \pm 3\%$	$51 \pm 2\%$	$1 \pm 4\%$
		successful stop vs baseline	$52 \pm 2\%$	$51 \pm 1\%$	$1 \pm 2\%$
		successful stop vs go	$62 \pm 3\%$	$66 \pm 3\%$	$-5 \pm 5\%$
[41]	Conditional stop-signal	failed stop critical vs baseline	$50 \pm 1\%$	$50 \pm 3\%$	$-1 \pm 3\%$
		failed stop non-critical vs baseline	$51 \pm 3\%$	$51 \pm 3\%$	$1 \pm 5\%$
		failed vs successful stop	$63 \pm 8\%$	$65 \pm 7\%$	$-2 \pm 11\%$
		go crital vs go non-crital	$50 \pm 4\%$	$49 \pm 5\%$	$0 \pm 6\%$
		go critical vs baseline	$52 \pm 5\%$	$50 \pm 1\%$	$3 \pm 5\%$
		go non-critical vs baseline	$53 \pm 6\%$	$55 \pm 6\%$	$-2 \pm 8\%$
		junk vs baseline	$57 \pm 6\%$	$54 \pm 3\%$	$3 \pm 7\%$
		successful stop critical vs baseline	$52 \pm 3\%$	$50 \pm 2\%$	$2 \pm 3\%$
		successful stop vs go	$69 \pm 5\%$	$74 \pm 6\%$	$-5 \pm 8\%$
	Stop-signal	failed stop vs baseline	$51 \pm 3\%$	$52 \pm 3\%$	$-1 \pm 4\%$
		failed vs successful stop	$63 \pm 8\%$	$65 \pm 7\%$	$-2 \pm 11\%$
		go vs baseline	$55 \pm 7\%$	$56 \pm 7\%$	$-0 \pm 7\%$
		junk vs baseline	$57 \pm 6\%$	$54 \pm 3\%$	$3 \pm 7\%$
		successful stop vs baseline	$53 \pm 4\%$	$50 \pm 3\%$	$3 \pm 5\%$
		successful stop vs go	$69 \pm 5\%$	$74 \pm 6\%$	$-5 \pm 8\%$
[42]	Balloon analog risk task	accept vs baseline	$75 \pm 7\%$	$74 \pm 6\%$	$1 \pm 7\%$

Continued on next page

Study	Task	Contrast	Multi-study B-acc	Voxel-level B-acc	B-acc gain
		accept vs reject	$98 \pm 3\%$	$90 \pm 6\%$	$8 \pm 6\%$
		explode vs baseline	$85 \pm 4\%$	$78 \pm 6\%$	$6 \pm 5\%$
		reject vs baseline	$88 \pm 6\%$	$85 \pm 7\%$	$3 \pm 7\%$
	Emotion regulation	attend negative vs attend neutral	$81 \pm 11\%$	$69 \pm 12\%$	$12 \pm 12\%$
		attend negative vs baseline	$78 \pm 7\%$	$68 \pm 11\%$	$9 \pm 9\%$
		attend neutral vs baseline	$64 \pm 10\%$	$63 \pm 9\%$	$1 \pm 10\%$
		junk rating vs baseline	$57 \pm 6\%$	$52 \pm 4\%$	$4 \pm 5\%$
		rating all vs baseline	$71 \pm 12\%$	$67 \pm 10\%$	$4 \pm 9\%$
		rating par vs baseline	$52 \pm 4\%$	$51 \pm 4\%$	$1 \pm 6\%$
		suppress negative vs attend negative	$56 \pm 6\%$	$53 \pm 5\%$	$3 \pm 7\%$
		suppress negative vs baseline	$61 \pm 8\%$	$55 \pm 7\%$	$6 \pm 7\%$
	Stop-signal	go vs baseline	$85 \pm 6\%$	$83 \pm 5\%$	$1 \pm 7\%$
		junk vs baseline	$72 \pm 7\%$	$68 \pm 6\%$	$4 \pm 6\%$
		successful stop vs baseline	$83 \pm 7\%$	$74 \pm 8\%$	$9 \pm 6\%$
		successful stop vs go	$97 \pm 2\%$	$94 \pm 5\%$	$3 \pm 4\%$
		unsuccessful stop vs baseline	$80 \pm 7\%$	$74 \pm 9\%$	$6 \pm 8\%$
		unsuccessul vs successful stop	$83 \pm 7\%$	$76 \pm 7\%$	$7 \pm 7\%$
	Temporal discounting task	easy all vs baseline	$54 \pm 8\%$	$51 \pm 7\%$	$2 \pm 5\%$
		easy par vs baseline	$50 \pm 0\%$	$49 \pm 1\%$	$1 \pm 1\%$
		hard all vs baseline	$50 \pm 0\%$	$54 \pm 7\%$	$-4 \pm 7\%$
		hard all vs easy all	$50 \pm 4\%$	$58 \pm 13\%$	$-7 \pm 14\%$
		hard par vs baseline	$50 \pm 0\%$	$49 \pm 6\%$	$1 \pm 6\%$
		junk vs baseline	$72 \pm 7\%$	$68 \pm 6\%$	$4 \pm 6\%$
[43]	Classification probe without feedback	correct dual task classification vs baseline	$60 \pm 8\%$	$56 \pm 7\%$	$4 \pm 7\%$
		correct single task classification vs baseline	$59 \pm 6\%$	$61 \pm 8\%$	$-2 \pm 8\%$
		correct single vs dual task classification	$90 \pm 3\%$	$76 \pm 9\%$	$14 \pm 9\%$
		junk dual task items vs baseline	$58 \pm 6\%$	$53 \pm 5\%$	$5 \pm 8\%$
		junk single task items vs baseline	$63 \pm 8\%$	$57 \pm 7\%$	$6 \pm 10\%$
	Dual-task weather classification	dual task classification vs baseline	$84 \pm 9\%$	$70 \pm 7\%$	$14 \pm 8\%$
		dual task classification vs probe	$90 \pm 7\%$	$89 \pm 7\%$	$1 \pm 9\%$
		dual task probe vs baseline	$88 \pm 7\%$	$83 \pm 7\%$	$5 \pm 8\%$
	Single-task weather classification	single task classification vs baseline	$84 \pm 9\%$	$80 \pm 12\%$	$4 \pm 9\%$
		single task classification vs probe	$91 \pm 6\%$	$84 \pm 8\%$	$7 \pm 9\%$
		single task probe vs baseline	$86 \pm 6\%$	$73 \pm 7\%$	$12 \pm 11\%$
	Tone-counting	tone counting probe vs baseline	$88 \pm 7\%$	$83 \pm 8\%$	$5 \pm 8\%$
		tone counting vs baseline	$97 \pm 4\%$	$87 \pm 8\%$	$10 \pm 7\%$
		tone counting vs probe	$96 \pm 5\%$	$87 \pm 9\%$	$9 \pm 7\%$
[28]	Classification learning	classification vs baseline	$85 \pm 7\%$	$82 \pm 7\%$	$3 \pm 7\%$

Continued on next page

Study	Task	Contrast	Multi-study B-acc	Voxel-level B-acc	B-acc gain
	Stop-signal	failed stop critical vs baseline	$68 \pm 7\%$	$72 \pm 8\%$	$-4 \pm 8\%$
		failed stop critical vs non-critical	$58 \pm 9\%$	$55 \pm 8\%$	$3 \pm 13\%$
		failed stop critical vs successful	$78 \pm 8\%$	$63 \pm 10\%$	$15 \pm 13\%$
		failed stop non-critical vs baseline	$66 \pm 10\%$	$59 \pm 7\%$	$7 \pm 11\%$
		go critical vs baseline	$63 \pm 13\%$	$65 \pm 11\%$	$-2 \pm 12\%$
		go critical vs non-critical	$62 \pm 10\%$	$56 \pm 6\%$	$5 \pm 10\%$
		go non-critical vs baseline	$84 \pm 9\%$	$79 \pm 17\%$	$5 \pm 16\%$
		junk vs baseline	$62 \pm 11\%$	$52 \pm 6\%$	$10 \pm 12\%$
		successful stop vs baseline	$65 \pm 8\%$	$56 \pm 8\%$	$9 \pm 8\%$
		successful stop vs go non-critical	$75 \pm 10\%$	$81 \pm 8\%$	$-6 \pm 12\%$
	Classification learning	classification vs baseline	$94 \pm 6\%$	$92 \pm 12\%$	$2 \pm 12\%$
	Stop-signal	failed stop critical vs baseline	$82 \pm 13\%$	$85 \pm 9\%$	$-3 \pm 12\%$
		failed stop critical vs non-critical	$84 \pm 6\%$	$71 \pm 11\%$	$13 \pm 10\%$
		failed stop critical vs successful	$91 \pm 10\%$	$64 \pm 9\%$	$27 \pm 11\%$
		failed stop non-critical vs baseline	$62 \pm 12\%$	$56 \pm 8\%$	$6 \pm 10\%$
		go critical vs baseline	$64 \pm 13\%$	$62 \pm 12\%$	$2 \pm 9\%$
		go critical vs non-critical	$78 \pm 14\%$	$64 \pm 10\%$	$14 \pm 14\%$
		go non-critical vs baseline	$80 \pm 8\%$	$77 \pm 10\%$	$3 \pm 7\%$
		junk vs baseline	$62 \pm 11\%$	$53 \pm 11\%$	$9 \pm 14\%$
		successful stop vs baseline	$64 \pm 13\%$	$57 \pm 9\%$	$7 \pm 20\%$
		successful stop vs go non-critical	$89 \pm 7\%$	$83 \pm 6\%$	$6 \pm 11\%$
[44]	Cross-language repetition priming	abstract vs concrete	$94 \pm 5\%$	$84 \pm 8\%$	$10 \pm 10\%$
		english english abstract novel vs baseline	$48 \pm 2\%$	$53 \pm 5\%$	$-4 \pm 5\%$
		english english abstract repeat vs baseline	$51 \pm 3\%$	$50 \pm 3\%$	$0 \pm 4\%$
		english english concrete novel vs baseline	$55 \pm 6\%$	$53 \pm 5\%$	$2 \pm 7\%$
		english english concrete repeat vs baseline	$71 \pm 11\%$	$67 \pm 9\%$	$4 \pm 6\%$
		english spanish abstract novel vs baseline	$61 \pm 9\%$	$59 \pm 8\%$	$2 \pm 10\%$
		english spanish abstract repeat vs baseline	$56 \pm 6\%$	$56 \pm 7\%$	$-0 \pm 5\%$
		english spanish concrete novel vs baseline	$52 \pm 4\%$	$57 \pm 7\%$	$-5 \pm 7\%$
		english spanish concrete repeat vs baseline	$54 \pm 4\%$	$55 \pm 7\%$	$-2 \pm 8\%$
		spanish english abstract novel vs baseline	$56 \pm 7\%$	$55 \pm 6\%$	$2 \pm 8\%$
		spanish english abstract repeat vs baseline	$53 \pm 5\%$	$52 \pm 5\%$	$0 \pm 5\%$
		spanish english concrete novel vs baseline	$52 \pm 5\%$	$53 \pm 6\%$	$-1 \pm 7\%$
		spanish english concrete repeat vs baseline	$54 \pm 6\%$	$52 \pm 3\%$	$2 \pm 6\%$

Continued on next page

Study	Task	Contrast	Multi-study B-acc	Voxel-level B-acc	B-acc gain
		spanish spanish abstract novel vs baseline	$58 \pm 6\%$	$56 \pm 8\%$	$2 \pm 6\%$
		spanish spanish abstract repeat vs baseline	$58 \pm 7\%$	$56 \pm 6\%$	$1 \pm 8\%$
		spanish spanish concrete novel vs baseline	$63 \pm 8\%$	$58 \pm 4\%$	$5 \pm 7\%$
		spanish spanish concrete repeat vs baseline	$64 \pm 9\%$	$63 \pm 7\%$	$1 \pm 9\%$
[45]	Classification learning	negative feedback vs baseline	$54 \pm 5\%$	$40 \pm 5\%$	$15 \pm 5\%$
		positive feedback vs baseline	$61 \pm 6\%$	$56 \pm 5\%$	$4 \pm 7\%$
		positive vs negative feedback	$81 \pm 5\%$	$57 \pm 4\%$	$24 \pm 6\%$
[46]	Simon task	congruent correct vs baseline	$59 \pm 8\%$	$59 \pm 7\%$	$0 \pm 7\%$
		congruent incorrect vs baseline	$49 \pm 10\%$	$46 \pm 5\%$	$3 \pm 7\%$
		incongruent correct vs baseline	$53 \pm 6\%$	$62 \pm 9\%$	$-10 \pm 12\%$
		incongruent incorrect vs baseline	$51 \pm 5\%$	$55 \pm 11\%$	$-4 \pm 12\%$
		incongruent vs congruent	$56 \pm 14\%$	$48 \pm 2\%$	$8 \pm 14\%$
		incongruent vs congruent correct	$49 \pm 7\%$	$47 \pm 2\%$	$2 \pm 8\%$
		incorrect vs correct	$56 \pm 8\%$	$52 \pm 6\%$	$4 \pm 9\%$
		incorrect vs correct incongruent	$55 \pm 8\%$	$62 \pm 8\%$	$-7 \pm 7\%$
[47]	Visual object recognition	bottle vs baseline	$60 \pm 13\%$	$55 \pm 9\%$	$5 \pm 11\%$
		cat vs baseline	$63 \pm 10\%$	$67 \pm 11\%$	$-3 \pm 10\%$
		chair vs baseline	$71 \pm 15\%$	$73 \pm 16\%$	$-2 \pm 17\%$
		chair vs scramble	$94 \pm 6\%$	$80 \pm 15\%$	$14 \pm 12\%$
		face vs baseline	$66 \pm 14\%$	$62 \pm 14\%$	$4 \pm 8\%$
		face vs house	$99 \pm 1\%$	$91 \pm 9\%$	$8 \pm 9\%$
		face vs scramble	$76 \pm 13\%$	$69 \pm 13\%$	$7 \pm 19\%$
		house vs baseline	$83 \pm 14\%$	$73 \pm 10\%$	$10 \pm 18\%$
		house vs face	$100 \pm 1\%$	$91 \pm 14\%$	$8 \pm 14\%$
		house vs scramble	$80 \pm 18\%$	$83 \pm 9\%$	$-3 \pm 19\%$
		scissors vs baseline	$66 \pm 12\%$	$71 \pm 8\%$	$-5 \pm 13\%$
		scramble vs baseline	$88 \pm 10\%$	$78 \pm 12\%$	$11 \pm 17\%$
		shoe vs baseline	$61 \pm 13\%$	$64 \pm 11\%$	$-3 \pm 8\%$
[48]	Word & object processing	consonant vs baseline	$94 \pm 3\%$	$90 \pm 2\%$	$3 \pm 3\%$
		objects vs baseline	$86 \pm 4\%$	$86 \pm 3\%$	$0 \pm 4\%$
		objects vs scrambled	$97 \pm 1\%$	$96 \pm 2\%$	$2 \pm 2\%$
		scramble vs baseline	$94 \pm 3\%$	$93 \pm 3\%$	$0 \pm 2\%$
		words vs baseline	$84 \pm 4\%$	$81 \pm 5\%$	$4 \pm 5\%$
		words vs consonants	$95 \pm 2\%$	$93 \pm 2\%$	$2 \pm 2\%$
[49]	Emotion regulation	look neg ant vs look neu ant	$52 \pm 3\%$	$51 \pm 3\%$	$1 \pm 3\%$
		look neg cue vs look neu cue	$56 \pm 3\%$	$61 \pm 5\%$	$-5 \pm 6\%$
		look neg rating vs look neu rating	$59 \pm 4\%$	$57 \pm 5\%$	$2 \pm 5\%$
		look neg stim vs look neu stim	$80 \pm 4\%$	$77 \pm 5\%$	$3 \pm 5\%$
		look negative ant vs baseline	$59 \pm 6\%$	$55 \pm 3\%$	$5 \pm 6\%$
		look negative cue vs baseline	$65 \pm 5\%$	$63 \pm 3\%$	$3 \pm 5\%$

Continued on next page

Study	Task	Contrast	Multi-study B-acc	Voxel-level B-acc	B-acc gain
		look negative rating vs baseline	$65 \pm 7\%$	$61 \pm 5\%$	$4 \pm 7\%$
		look negative stim vs baseline	$74 \pm 5\%$	$70 \pm 5\%$	$4 \pm 6\%$
		look neu ant vs look neg ant	$53 \pm 3\%$	$54 \pm 4\%$	$-0 \pm 4\%$
		look neu ant vs reapp neg ant	$54 \pm 4\%$	$53 \pm 4\%$	$1 \pm 3\%$
		look neu cue vs look neg cue	$57 \pm 5\%$	$60 \pm 6\%$	$-3 \pm 6\%$
		look neu rating vs look neg rating	$55 \pm 4\%$	$55 \pm 5\%$	$0 \pm 5\%$
		look neu rating vs reapp neg rating	$61 \pm 6\%$	$57 \pm 5\%$	$4 \pm 5\%$
		look neu stim vs look neg stim	$70 \pm 5\%$	$65 \pm 5\%$	$5 \pm 4\%$
		look neu stim vs reapp neg stim	$80 \pm 5\%$	$78 \pm 6\%$	$2 \pm 4\%$
		look neutral ant vs baseline	$57 \pm 5\%$	$56 \pm 4\%$	$1 \pm 5\%$
		look neutral cue vs baseline	$61 \pm 5\%$	$59 \pm 4\%$	$2 \pm 4\%$
		look neutral rating vs baseline	$70 \pm 5\%$	$65 \pm 5\%$	$6 \pm 6\%$
		look neutral stim vs baseline	$90 \pm 4\%$	$85 \pm 7\%$	$5 \pm 6\%$
		reapp neg ant vs look neu ant	$55 \pm 4\%$	$52 \pm 4\%$	$3 \pm 5\%$
		reapp neg cue vs look neg cue	$52 \pm 3\%$	$52 \pm 4\%$	$0 \pm 3\%$
		reapp neg stim vs look neg stim	$64 \pm 6\%$	$67 \pm 5\%$	$-3 \pm 5\%$
		reapp negative ant vs baseline	$66 \pm 5\%$	$65 \pm 3\%$	$0 \pm 5\%$
		reapp negative cue vs baseline	$60 \pm 6\%$	$59 \pm 5\%$	$2 \pm 5\%$
		reapp negative rating vs baseline	$71 \pm 6\%$	$68 \pm 6\%$	$2 \pm 7\%$
		reapp negative stim vs baseline	$80 \pm 7\%$	$76 \pm 7\%$	$4 \pm 8\%$
[50]	False belief	false belief question vs baseline	$76 \pm 5\%$	$71 \pm 6\%$	$5 \pm 5\%$
		false belief story vs baseline	$78 \pm 5\%$	$77 \pm 4\%$	$1 \pm 6\%$
		false photo question vs baseline	$82 \pm 6\%$	$72 \pm 5\%$	$9 \pm 5\%$
		false photo story vs baseline	$84 \pm 6\%$	$79 \pm 5\%$	$4 \pm 5\%$
		falsebelief vs falsepicture	$59 \pm 6\%$	$54 \pm 3\%$	$5 \pm 6\%$
		falsebeliefquestion vs falsepicturequestion	$71 \pm 6\%$	$68 \pm 6\%$	$4 \pm 5\%$
		falsebeliefstory vs falsepicturestory	$69 \pm 5\%$	$73 \pm 4\%$	$-4 \pm 4\%$
[51]	Incidental encoding	cue vs fixation	$51 \pm 4\%$	$50 \pm 0\%$	$2 \pm 4\%$
		high confidence hit object vs miss object	$50 \pm 2\%$	$50 \pm 3\%$	$0 \pm 4\%$
		invalid high confidence hit cue vs baseline	$50 \pm 2\%$	$51 \pm 5\%$	$-1 \pm 5\%$
		invalid high confidence hit object vs baseline	$49 \pm 2\%$	$50 \pm 4\%$	$-1 \pm 5\%$
		invalid high confidence hit object vs invalid miss object	$54 \pm 6\%$	$50 \pm 3\%$	$4 \pm 6\%$
		invalid low confidence hit cue vs baseline	$56 \pm 7\%$	$51 \pm 2\%$	$5 \pm 6\%$

Continued on next page

Study	Task	Contrast	Multi-study B-acc	Voxel-level B-acc	B-acc gain
		invalid low confidence hit object vs baseline	$57 \pm 5\%$	$53 \pm 3\%$	$3 \pm 4\%$
		invalid miss cue vs baseline	$49 \pm 2\%$	$54 \pm 4\%$	$-5 \pm 5\%$
		invalid miss object vs baseline	$58 \pm 6\%$	$57 \pm 7\%$	$1 \pm 11\%$
		invalid miss object vs invalid high confidence hit object	$54 \pm 6\%$	$53 \pm 7\%$	$1 \pm 6\%$
		invalid other greeble cue vs baseline	$53 \pm 6\%$	$51 \pm 3\%$	$2 \pm 5\%$
		invalid other greeble vs baseline	$57 \pm 8\%$	$52 \pm 5\%$	$5 \pm 9\%$
		invalidly vs validly cued objects	$52 \pm 4\%$	$50 \pm 2\%$	$2 \pm 5\%$
		miss object vs high confidence hit object	$50 \pm 2\%$	$50 \pm 1\%$	$-0 \pm 2\%$
		valid high confidence hit cue vs baseline	$60 \pm 5\%$	$57 \pm 6\%$	$3 \pm 7\%$
		valid high confidence hit object vs baseline	$73 \pm 7\%$	$62 \pm 8\%$	$11 \pm 9\%$
		valid high confidence hit object vs valid miss object	$64 \pm 6\%$	$65 \pm 9\%$	$-1 \pm 11\%$
		valid low confidence hit cue vs baseline	$52 \pm 3\%$	$49 \pm 1\%$	$2 \pm 3\%$
		valid low confidence hit object vs baseline	$52 \pm 4\%$	$52 \pm 5\%$	$-1 \pm 5\%$
		valid miss cue vs baseline	$55 \pm 5\%$	$51 \pm 3\%$	$4 \pm 6\%$
		valid miss object vs baseline	$59 \pm 6\%$	$55 \pm 5\%$	$4 \pm 7\%$
		valid other greeble cue vs baseline	$70 \pm 8\%$	$56 \pm 5\%$	$13 \pm 11\%$
		valid other greeble vs baseline	$76 \pm 10\%$	$65 \pm 5\%$	$11 \pm 8\%$
		valid other object cue vs baseline	$53 \pm 4\%$	$50 \pm 2\%$	$2 \pm 4\%$
		valid other object vs baseline	$56 \pm 5\%$	$52 \pm 5\%$	$3 \pm 6\%$
		valid valid miss object vs high confidence hit object	$67 \pm 9\%$	$63 \pm 9\%$	$4 \pm 9\%$
[52]	Covert verb generation	covert verb generation vs baseline	$86 \pm 8\%$	$84 \pm 8\%$	$2 \pm 6\%$
	Line bisection	correct bisection vs baseline	$73 \pm 12\%$	$71 \pm 12\%$	$2 \pm 16\%$
		incorrect bisection vs baseline	$57 \pm 9\%$	$50 \pm 5\%$	$7 \pm 9\%$
		no response control vs baseline	$68 \pm 13\%$	$62 \pm 10\%$	$6 \pm 15\%$
		no response task vs baseline	$56 \pm 9\%$	$51 \pm 5\%$	$6 \pm 11\%$
		response control vs baseline	$71 \pm 8\%$	$68 \pm 10\%$	$4 \pm 10\%$
	Motor	finger vs baseline	$85 \pm 8\%$	$84 \pm 8\%$	$1 \pm 6\%$
		foot vs baseline	$84 \pm 8\%$	$82 \pm 8\%$	$2 \pm 11\%$
		lips vs baseline	$90 \pm 6\%$	$87 \pm 9\%$	$3 \pm 9\%$
	Overt verb generation	overt verb generation vs baseline	$69 \pm 10\%$	$62 \pm 8\%$	$6 \pm 12\%$
	Overt word repetition	overt word repetition vs baseline	$73 \pm 14\%$	$58 \pm 7\%$	$15 \pm 15\%$
[53]	Auditory oddball	auditory oddball vs baseline	$58 \pm 7\%$	$55 \pm 5\%$	$4 \pm 9\%$
		auditory oddball vs standard	$63 \pm 7\%$	$68 \pm 10\%$	$-5 \pm 10\%$

Continued on next page

Study	Task	Contrast	Multi-study B-acc	Voxel-level B-acc	B-acc gain
		auditory rt vs baseline	$71 \pm 6\%$	$55 \pm 4\%$	$15 \pm 7\%$
		auditory standard vs baseline	$87 \pm 8\%$	$80 \pm 6\%$	$7 \pm 7\%$
	Visual oddball	visual oddball vs baseline	$60 \pm 9\%$	$50 \pm 4\%$	$10 \pm 7\%$
		visual oddball vs standard	$68 \pm 9\%$	$69 \pm 8\%$	$-1 \pm 11\%$
		visual rt vs baseline	$70 \pm 9\%$	$60 \pm 7\%$	$10 \pm 11\%$
		visual standard vs baseline	$82 \pm 7\%$	$74 \pm 7\%$	$8 \pm 9\%$
[54]	Continuous house vs face	baseline	$92 \pm 5\%$	$88 \pm 6\%$	$4 \pm 5\%$
		continuous house face 100ms frequency vs baseline	$58 \pm 7\%$	$64 \pm 8\%$	$-6 \pm 8\%$
		continuous house face 1600ms frequency vs baseline	$52 \pm 5\%$	$52 \pm 5\%$	$-0 \pm 6\%$
		continuous house face 17ms frequency vs baseline	$58 \pm 6\%$	$58 \pm 7\%$	$0 \pm 5\%$
		continuous house face 200ms frequency vs baseline	$67 \pm 8\%$	$58 \pm 7\%$	$9 \pm 9\%$
		continuous house face 3200ms frequency vs baseline	$53 \pm 7\%$	$52 \pm 4\%$	$1 \pm 6\%$
		continuous house face 33ms frequency vs baseline	$55 \pm 6\%$	$53 \pm 6\%$	$2 \pm 8\%$
		continuous house face 400ms frequency vs baseline	$51 \pm 4\%$	$51 \pm 4\%$	$0 \pm 4\%$
		continuous house face 4800ms frequency vs baseline	$60 \pm 8\%$	$60 \pm 9\%$	$1 \pm 9\%$
		continuous house face 50ms frequency vs baseline	$57 \pm 8\%$	$54 \pm 5\%$	$3 \pm 6\%$
		continuous house face 800ms frequency vs baseline	$54 \pm 6\%$	$51 \pm 4\%$	$3 \pm 6\%$
		high vs low frequency	$76 \pm 6\%$	$73 \pm 7\%$	$3 \pm 5\%$
		low vs high frequency	$85 \pm 5\%$	$78 \pm 6\%$	$7 \pm 5\%$
	Discontinuous house (800ms) vs face	baseline	$92 \pm 5\%$	$88 \pm 6\%$	$4 \pm 5\%$
		discontinuous house face 800ms frequency 100ms duration vs baseline	$52 \pm 5\%$	$50 \pm 2\%$	$2 \pm 4\%$
		discontinuous house face 800ms frequency 33ms duration vs baseline	$51 \pm 3\%$	$52 \pm 4\%$	$-1 \pm 4\%$
		discontinuous house face 800ms frequency 400ms duration vs baseline	$53 \pm 7\%$	$52 \pm 4\%$	$1 \pm 7\%$
		discontinuous house face 800ms frequency 50ms duration vs baseline	$54 \pm 5\%$	$54 \pm 5\%$	$-0 \pm 6\%$
		discontinuous house face 800ms frequency 800ms duration vs baseline	$53 \pm 6\%$	$53 \pm 6\%$	$1 \pm 5\%$
		high vs low frequency	$76 \pm 6\%$	$73 \pm 7\%$	$3 \pm 5\%$
		low vs high frequency	$85 \pm 5\%$	$78 \pm 6\%$	$7 \pm 5\%$
		medium vs other frequency	$65 \pm 6\%$	$65 \pm 9\%$	$-0 \pm 8\%$

Continued on next page

Study	Task	Contrast	Multi-study B-acc	Voxel-level B-acc	B-acc gain
	Discoutinuous house (400ms) vs face	baseline	$92 \pm 5\%$	$88 \pm 6\%$	$4 \pm 5\%$
		discontinuous house face 400ms frequency 100ms duration vs baseline	$55 \pm 7\%$	$51 \pm 3\%$	$4 \pm 7\%$
		discontinuous house face 400ms frequency 200ms duration vs baseline	$51 \pm 5\%$	$56 \pm 7\%$	$-5 \pm 7\%$
		discontinuous house face 400ms frequency 33ms duration vs baseline	$50 \pm 2\%$	$53 \pm 4\%$	$-2 \pm 5\%$
		discontinuous house face 400ms frequency 400ms duration vs baseline	$58 \pm 5\%$	$66 \pm 9\%$	$-7 \pm 9\%$
		discontinuous house face 400ms frequency 50ms duration vs baseline	$57 \pm 7\%$	$55 \pm 6\%$	$2 \pm 7\%$
		high vs low frequency	$76 \pm 6\%$	$73 \pm 7\%$	$3 \pm 5\%$
		low vs high frequency	$85 \pm 5\%$	$78 \pm 6\%$	$7 \pm 5\%$
		medium vs other frequency	$65 \pm 6\%$	$65 \pm 9\%$	$-0 \pm 8\%$
	House vs face	baseline	$92 \pm 5\%$	$88 \pm 6\%$	$4 \pm 5\%$
		face vs baseline	$91 \pm 6\%$	$77 \pm 11\%$	$13 \pm 10\%$
		face vs house	$100 \pm 0\%$	$97 \pm 5\%$	$3 \pm 5\%$
		house vs baseline	$93 \pm 6\%$	$85 \pm 10\%$	$8 \pm 7\%$
		object vs baseline	$88 \pm 6\%$	$87 \pm 12\%$	$2 \pm 10\%$
		object vs scramble	$95 \pm 6\%$	$84 \pm 15\%$	$11 \pm 11\%$
		scramble vs baseline	$81 \pm 12\%$	$81 \pm 10\%$	$0 \pm 5\%$
	Continuous house vs face	baseline	$96 \pm 2\%$	$90 \pm 5\%$	$5 \pm 5\%$
		continuous house face 100ms frequency vs baseline	$57 \pm 4\%$	$58 \pm 5\%$	$-1 \pm 3\%$
		continuous house face 125ms frequency vs baseline	$58 \pm 5\%$	$56 \pm 4\%$	$2 \pm 4\%$
		continuous house face 150ms frequency vs baseline	$53 \pm 3\%$	$57 \pm 7\%$	$-4 \pm 6\%$
		continuous house face 175ms frequency vs baseline	$55 \pm 4\%$	$55 \pm 5\%$	$-0 \pm 3\%$
		continuous house face 200ms frequency vs baseline	$62 \pm 5\%$	$61 \pm 4\%$	$1 \pm 6\%$
		continuous house face 250ms frequency vs baseline	$54 \pm 4\%$	$56 \pm 6\%$	$-1 \pm 6\%$
		continuous house face 400ms frequency vs baseline	$54 \pm 4\%$	$58 \pm 6\%$	$-5 \pm 4\%$
		continuous house face 50ms frequency vs baseline	$55 \pm 4\%$	$56 \pm 5\%$	$-1 \pm 5\%$
		continuous house face 75ms frequency vs baseline	$56 \pm 5\%$	$57 \pm 5\%$	$-1 \pm 6\%$
		continuous house face 800ms frequency vs baseline	$60 \pm 5\%$	$62 \pm 6\%$	$-2 \pm 7\%$
		continuous house face high vs low frequency	$77 \pm 5\%$	$75 \pm 5\%$	$2 \pm 5\%$

Continued on next page

Study	Task	Contrast	Multi-study B-acc	Voxel-level B-acc	B-acc gain
		continuous house face low vs high frequency	$77 \pm 5\%$	$79 \pm 4\%$	$-2 \pm 7\%$
		hits vs baseline	$85 \pm 7\%$	$76 \pm 7\%$	$9 \pm 9\%$
		hits vs misses	$68 \pm 7\%$	$64 \pm 7\%$	$3 \pm 7\%$
		misses vs baseline	$69 \pm 6\%$	$64 \pm 8\%$	$5 \pm 7\%$
		misses vs hits	$72 \pm 9\%$	$70 \pm 7\%$	$2 \pm 9\%$
	House vs face	baseline	$96 \pm 2\%$	$90 \pm 5\%$	$5 \pm 5\%$
		face vs baseline	$98 \pm 3\%$	$88 \pm 9\%$	$10 \pm 8\%$
		face vs house	$100 \pm 0\%$	$100 \pm 0\%$	$0 \pm 0\%$
		house vs baseline	$100 \pm 0\%$	$96 \pm 4\%$	$4 \pm 4\%$
		object vs baseline	$98 \pm 3\%$	$89 \pm 7\%$	$10 \pm 6\%$
		object vs scramble	$100 \pm 0\%$	$97 \pm 5\%$	$3 \pm 5\%$
		scramble vs baseline	$100 \pm 0\%$	$93 \pm 7\%$	$7 \pm 7\%$
[55]	Emotion	faces	$98 \pm 1\%$	$99 \pm 0\%$	$-1 \pm 1\%$
		shapes	$98 \pm 0\%$	$99 \pm 0\%$	$-1 \pm 0\%$
	Gambling	punish	$88 \pm 2\%$	$92 \pm 1\%$	$-4 \pm 1\%$
		reward	$88 \pm 1\%$	$92 \pm 1\%$	$-4 \pm 1\%$
	Language	math	$99 \pm 0\%$	$99 \pm 0\%$	$0 \pm 0\%$
		story	$99 \pm 0\%$	$99 \pm 0\%$	$-0 \pm 0\%$
	Motor	cue	$100 \pm 0\%$	$100 \pm 0\%$	$0 \pm 0\%$
		lf	$100 \pm 0\%$	$100 \pm 0\%$	$-0 \pm 0\%$
		lh	$100 \pm 0\%$	$100 \pm 0\%$	$-0 \pm 0\%$
		rf	$99 \pm 0\%$	$100 \pm 0\%$	$-1 \pm 0\%$
		rh	$100 \pm 0\%$	$100 \pm 0\%$	$-0 \pm 0\%$
	Relational	match	$91 \pm 1\%$	$94 \pm 1\%$	$-3 \pm 1\%$
		rel	$92 \pm 1\%$	$94 \pm 1\%$	$-3 \pm 1\%$
	Social	random	$98 \pm 0\%$	$99 \pm 0\%$	$-1 \pm 0\%$
		tom	$99 \pm 0\%$	$99 \pm 0\%$	$-0 \pm 0\%$
	Wm	0bk body	$89 \pm 1\%$	$91 \pm 2\%$	$-2 \pm 1\%$
		0bk face	$94 \pm 1\%$	$94 \pm 1\%$	$-0 \pm 0\%$
		0bk place	$92 \pm 0\%$	$92 \pm 1\%$	$0 \pm 1\%$
		0bk tool	$90 \pm 0\%$	$93 \pm 1\%$	$-2 \pm 1\%$
		2bk body	$92 \pm 2\%$	$93 \pm 1\%$	$-1 \pm 1\%$
		2bk face	$95 \pm 1\%$	$96 \pm 1\%$	$-1 \pm 0\%$
		2bk place	$94 \pm 1\%$	$94 \pm 0\%$	$-0 \pm 0\%$
		2bk tool	$91 \pm 1\%$	$93 \pm 1\%$	$-2 \pm 1\%$
[56]	Face recognition	faces vs scrambled faces	$91 \pm 8\%$	$92 \pm 5\%$	$-1 \pm 8\%$
		famous faces vs baseline	$70 \pm 7\%$	$68 \pm 7\%$	$3 \pm 5\%$
		famous vs unfamiliar faces	$95 \pm 3\%$	$82 \pm 9\%$	$12 \pm 9\%$
		scrambled faces vs baseline	$83 \pm 9\%$	$78 \pm 10\%$	$5 \pm 8\%$
		unfamiliar faces vs baseline	$65 \pm 6\%$	$65 \pm 7\%$	$0 \pm 7\%$
[57]	Arithmetic	first operand non-symbolic addition vs baseline	$61 \pm 8\%$	$59 \pm 5\%$	$2 \pm 7\%$
		first operand non-symbolic color vs baseline	$65 \pm 8\%$	$58 \pm 7\%$	$6 \pm 7\%$
		first operand non-symbolic subtraction vs baseline	$64 \pm 8\%$	$57 \pm 6\%$	$8 \pm 9\%$
		first operand symbolic addition vs baseline	$64 \pm 7\%$	$60 \pm 5\%$	$4 \pm 6\%$
		first operand symbolic color vs baseline	$72 \pm 6\%$	$69 \pm 8\%$	$3 \pm 5\%$
		first operand symbolic subtraction vs baseline	$68 \pm 8\%$	$63 \pm 5\%$	$5 \pm 8\%$
		operator addition vs baseline	$60 \pm 5\%$	$59 \pm 5\%$	$1 \pm 7\%$
		operator color vs baseline	$71 \pm 8\%$	$72 \pm 8\%$	$-1 \pm 7\%$
		operator subtraction vs baseline	$66 \pm 7\%$	$57 \pm 6\%$	$9 \pm 8\%$

Continued on next page

Study	Task	Contrast	Multi-study B-acc	Voxel-level B-acc	B-acc gain
		response vs baseline	$100 \pm 0\%$	$93 \pm 6\%$	$7 \pm 6\%$
		second operand non-symbolic addition larger vs baseline	$56 \pm 4\%$	$55 \pm 3\%$	$1 \pm 5\%$
		second operand non-symbolic addition smaller vs baseline	$61 \pm 6\%$	$62 \pm 5\%$	$-1 \pm 7\%$
		second operand non-symbolic color larger vs baseline	$55 \pm 6\%$	$54 \pm 5\%$	$1 \pm 4\%$
		second operand non-symbolic color smaller vs baseline	$56 \pm 7\%$	$55 \pm 6\%$	$1 \pm 5\%$
		second operand non-symbolic subtraction larger vs baseline	$63 \pm 6\%$	$62 \pm 6\%$	$0 \pm 5\%$
		second operand non-symbolic subtraction smaller vs baseline	$56 \pm 6\%$	$56 \pm 3\%$	$-0 \pm 7\%$
		second operand symbolic addition larger vs baseline	$72 \pm 9\%$	$69 \pm 7\%$	$3 \pm 8\%$
		second operand symbolic addition smaller vs baseline	$60 \pm 8\%$	$55 \pm 6\%$	$5 \pm 7\%$
		second operand symbolic color larger vs baseline	$56 \pm 5\%$	$53 \pm 3\%$	$3 \pm 5\%$
		second operand symbolic color smaller vs baseline	$55 \pm 5\%$	$56 \pm 5\%$	$-0 \pm 6\%$
		second operand symbolic subtraction larger vs baseline	$71 \pm 11\%$	$67 \pm 10\%$	$5 \pm 7\%$
		second operand symbolic subtraction smaller vs baseline	$60 \pm 6\%$	$54 \pm 3\%$	$6 \pm 7\%$
	Saccades	left field vs baseline	$74 \pm 7\%$	$75 \pm 5\%$	$-1 \pm 5\%$
		left vs right field	$80 \pm 6\%$	$78 \pm 7\%$	$2 \pm 7\%$
		right field vs baseline	$77 \pm 6\%$	$72 \pm 9\%$	$5 \pm 8\%$
		right vs left field	$81 \pm 5\%$	$80 \pm 5\%$	$0 \pm 3\%$
[58]	Balloon analog risk task	baloon accept	$86 \pm 2\%$	$86 \pm 1\%$	$-0 \pm 1\%$
		baloon cashout	$90 \pm 1\%$	$92 \pm 1\%$	$-2 \pm 1\%$
		baloon explode	$91 \pm 1\%$	$94 \pm 2\%$	$-3 \pm 0\%$
		control accept	$86 \pm 1\%$	$86 \pm 2\%$	$0 \pm 2\%$
		control cashout	$61 \pm 3\%$	$63 \pm 3\%$	$-3 \pm 3\%$
	Breath hold task	hold ons	$80 \pm 2\%$	$82 \pm 2\%$	$-2 \pm 2\%$
		prep ons	$91 \pm 2\%$	$92 \pm 1\%$	$-1 \pm 1\%$
		rest ons	$86 \pm 2\%$	$85 \pm 2\%$	$1 \pm 1\%$
	Paired associates memory task (encoding)	control	$90 \pm 1\%$	$91 \pm 1\%$	$-1 \pm 1\%$
		task	$94 \pm 1\%$	$93 \pm 1\%$	$1 \pm 1\%$
	Paired associates memory task (retrieval)	control	$90 \pm 1\%$	$91 \pm 1\%$	$-1 \pm 1\%$
		correctly	$75 \pm 3\%$	$77 \pm 2\%$	$-3 \pm 3\%$
		incorrectly	$78 \pm 4\%$	$79 \pm 3\%$	$-1 \pm 3\%$
	SCAP working memory tasks	correct	$85 \pm 2\%$	$86 \pm 2\%$	$-1 \pm 2\%$
		incorrect	$81 \pm 2\%$	$81 \pm 3\%$	$0 \pm 3\%$

Continued on next page

Study	Task	Contrast	Multi-study B-acc	Voxel-level B-acc	B-acc gain
		no response	$71 \pm 4\%$	$70 \pm 3\%$	$1 \pm 2\%$
	Stop-signal	blankscreen	$95 \pm 1\%$	$93 \pm 1\%$	$1 \pm 1\%$
		go left	$68 \pm 2\%$	$70 \pm 3\%$	$-2 \pm 1\%$
		go right	$73 \pm 2\%$	$74 \pm 2\%$	$-1 \pm 2\%$
		stop left	$71 \pm 2\%$	$74 \pm 2\%$	$-3 \pm 2\%$
		stop right	$75 \pm 3\%$	$75 \pm 3\%$	$-1 \pm 3\%$
	Task switching	noswitch color	$67 \pm 2\%$	$69 \pm 2\%$	$-2 \pm 2\%$
		noswitch shape	$67 \pm 2\%$	$71 \pm 2\%$	$-4 \pm 3\%$
		switch color	$68 \pm 3\%$	$68 \pm 2\%$	$-1 \pm 3\%$
		switch shape	$70 \pm 2\%$	$68 \pm 2\%$	$2 \pm 3\%$
[59]	Foreign language	french vs baseline	$94 \pm 3\%$	$92 \pm 3\%$	$3 \pm 2\%$
		french vs korean	$97 \pm 2\%$	$93 \pm 2\%$	$3 \pm 3\%$
		french vs sound	$87 \pm 4\%$	$79 \pm 4\%$	$8 \pm 3\%$
		korean vs baseline	$91 \pm 3\%$	$92 \pm 2\%$	$-0 \pm 3\%$
		korean vs sound	$93 \pm 3\%$	$91 \pm 4\%$	$2 \pm 4\%$
		language vs sound	$76 \pm 5\%$	$74 \pm 5\%$	$2 \pm 4\%$
		sound vs baseline	$94 \pm 5\%$	$96 \pm 4\%$	$-2 \pm 3\%$
		sound vs french	$97 \pm 1\%$	$95 \pm 3\%$	$2 \pm 3\%$
		sound vs korean	$98 \pm 2\%$	$98 \pm 1\%$	$-1 \pm 2\%$
	Localizer	action vs baseline	$96 \pm 2\%$	$97 \pm 2\%$	$-1 \pm 1\%$
		digit vs baseline	$91 \pm 4\%$	$91 \pm 4\%$	$-0 \pm 3\%$
		digit vs house	$93 \pm 3\%$	$92 \pm 3\%$	$1 \pm 5\%$
		digit vs scramble	$90 \pm 5\%$	$90 \pm 4\%$	$-0 \pm 3\%$
		digit vs words	$99 \pm 1\%$	$96 \pm 3\%$	$3 \pm 3\%$
		face vs baseline	$97 \pm 2\%$	$98 \pm 1\%$	$-1 \pm 2\%$
		face vs house	$99 \pm 1\%$	$98 \pm 2\%$	$1 \pm 2\%$
		face vs scramble	$98 \pm 2\%$	$97 \pm 3\%$	$1 \pm 3\%$
		house vs baseline	$97 \pm 2\%$	$97 \pm 3\%$	$1 \pm 2\%$
		house vs scramble	$100 \pm 0\%$	$99 \pm 2\%$	$1 \pm 2\%$
		scramble vs baseline	$98 \pm 1\%$	$97 \pm 2\%$	$2 \pm 2\%$
		tool vs baseline	$91 \pm 3\%$	$93 \pm 2\%$	$-2 \pm 3\%$
		tool vs house	$99 \pm 1\%$	$99 \pm 1\%$	$-0 \pm 1\%$
		tool vs scramble	$98 \pm 3\%$	$96 \pm 4\%$	$2 \pm 3\%$
		words vs baseline	$91 \pm 4\%$	$88 \pm 4\%$	$3 \pm 4\%$
		words vs digit	$97 \pm 2\%$	$97 \pm 3\%$	$0 \pm 3\%$
		words vs house	$91 \pm 3\%$	$91 \pm 3\%$	$-0 \pm 4\%$
		words vs scramble	$92 \pm 3\%$	$91 \pm 3\%$	$2 \pm 4\%$
	Saccade	calculation vs baseline	$95 \pm 2\%$	$96 \pm 1\%$	$-1 \pm 2\%$
		calculation vs next number	$97 \pm 2\%$	$96 \pm 3\%$	$1 \pm 3\%$
		calculation vs saccade	$96 \pm 3\%$	$94 \pm 4\%$	$2 \pm 2\%$
		next number vs baseline	$99 \pm 2\%$	$98 \pm 2\%$	$1 \pm 1\%$
		saccade vs baseline	$97 \pm 2\%$	$93 \pm 3\%$	$4 \pm 2\%$
		saccade vs calculation	$97 \pm 3\%$	$92 \pm 2\%$	$5 \pm 2\%$
		saccade vs next number	$95 \pm 2\%$	$91 \pm 3\%$	$3 \pm 3\%$
[60]	Auditory compression	auditory bottleneck vs language	$96 \pm 3\%$	$97 \pm 3\%$	$-0 \pm 3\%$
		auditory language vs bottleneck	$90 \pm 6\%$	$78 \pm 9\%$	$12 \pm 9\%$
		auditory sentences 100% duration vs baseline	$74 \pm 10\%$	$69 \pm 10\%$	$5 \pm 9\%$
		auditory sentences 20% duration vs baseline	$84 \pm 5\%$	$81 \pm 7\%$	$3 \pm 8\%$
		auditory sentences 40% duration vs baseline	$84 \pm 7\%$	$83 \pm 8\%$	$0 \pm 5\%$
		auditory sentences 60% duration vs baseline	$67 \pm 7\%$	$61 \pm 9\%$	$6 \pm 6\%$
		auditory sentences 80% duration vs baseline	$61 \pm 7\%$	$67 \pm 8\%$	$-6 \pm 6\%$

Continued on next page

Study	Task	Contrast	Multi-study B-acc	Voxel-level B-acc	B-acc gain
	Visual compression	visual bottleneck vs language	$99 \pm 2\%$	$98 \pm 3\%$	$1 \pm 4\%$
		visual language vs bottleneck	$93 \pm 5\%$	$78 \pm 6\%$	$15 \pm 6\%$
		visual sentences 100% duration vs baseline	$76 \pm 7\%$	$74 \pm 10\%$	$2 \pm 8\%$
		visual sentences 20% duration vs baseline	$76 \pm 8\%$	$64 \pm 7\%$	$12 \pm 10\%$
		visual sentences 40% duration vs baseline	$70 \pm 10\%$	$77 \pm 9\%$	$-7 \pm 10\%$
		visual sentences 60% duration vs baseline	$61 \pm 5\%$	$53 \pm 5\%$	$9 \pm 6\%$
		visual sentences 80% duration vs baseline	$69 \pm 8\%$	$62 \pm 8\%$	$7 \pm 7\%$

Table A. Accuracies per study in multi-study decoding.

Study	Chance level	Multi-task accuracy	Single-task accuracy	Accuracy gain
[29] High level math & Localizer	3%	$83 \pm 1\%$	$81 \pm 1\%$	$2 \pm 1\%$
[30] The ARCHI project	3%	$87 \pm 1\%$	$86 \pm 1\%$	$1 \pm 1\%$
[15] Brainomics	5%	$94 \pm 1\%$	$88 \pm 2\%$	$5 \pm 1\%$
[31] CamCAN	20%	$66 \pm 1\%$	$66 \pm 1\%$	$0 \pm 1\%$
[32, 33] Music structure & Sentence structure	5%	$48 \pm 2\%$	$45 \pm 2\%$	$3 \pm 2\%$
[34] Sentence/music complexity	4%	$38 \pm 3\%$	$34 \pm 3\%$	$4 \pm 3\%$
[35] Balloon Analog Risk-taking	8%	$59 \pm 6\%$	$46 \pm 7\%$	$13 \pm 4\%$
[36] Baseline trials & Classication learning	14%	$62 \pm 5\%$	$55 \pm 5\%$	$7 \pm 5\%$
[37] Rhyme judgment	33%	$65 \pm 8\%$	$53 \pm 11\%$	$12 \pm 10\%$
[38] Mixed-gambles	25%	$72 \pm 7\%$	$59 \pm 8\%$	$13 \pm 8\%$
[39] Plain or mirror-reversed text	11%	$20 \pm 5\%$	$20 \pm 3\%$	$-0 \pm 5\%$
[40] Stop-signal	17%	$29 \pm 3\%$	$31 \pm 3\%$	$-2 \pm 3\%$
[41] Conditional stop-signal & Stop-signal	8%	$23 \pm 5\%$	$23 \pm 4\%$	$-0 \pm 5\%$
[42] Balloon analog risk task & Emotion regulation & Stop-signal & Temporal discounting task	4%	$56 \pm 4\%$	$47 \pm 4\%$	$8 \pm 4\%$
[43] Classification probe without feedback & Dual-task weather classification & Single-task weather classification & Tone-counting	7%	$65 \pm 3\%$	$52 \pm 4\%$	$12 \pm 4\%$
[28] Classification learning & Stop-signal	9%	$45 \pm 6\%$	$37 \pm 6\%$	$8 \pm 8\%$
[28] Classification learning & Stop-signal	9%	$59 \pm 8\%$	$45 \pm 6\%$	$14 \pm 6\%$
[44] Cross-language repetition priming	6%	$22 \pm 3\%$	$20 \pm 3\%$	$2 \pm 3\%$
[45] Classification learning	33%	$54 \pm 4\%$	$35 \pm 5\%$	$19 \pm 5\%$
[46] Simon task	12%	$19 \pm 7\%$	$19 \pm 4\%$	$-1 \pm 8\%$
[47] Visual object recognition	8%	$59 \pm 7\%$	$52 \pm 4\%$	$7 \pm 8\%$
[48] Word & object processing	17%	$86 \pm 3\%$	$83 \pm 2\%$	$3 \pm 3\%$
[49] Emotion regulation	4%	$31 \pm 2\%$	$28 \pm 2\%$	$4 \pm 2\%$
[50] False belief	14%	$56 \pm 4\%$	$50 \pm 3\%$	$6 \pm 4\%$
[51] Incidental encoding	4%	$17 \pm 2\%$	$11 \pm 2\%$	$6 \pm 2\%$
[52] Covert verb generation & Line bisection & Motor & Overt verb generation & Overt word repetition	9%	$52 \pm 7\%$	$44 \pm 6\%$	$9 \pm 7\%$
[53] Auditory oddball & Visual oddball	12%	$47 \pm 6\%$	$36 \pm 5\%$	$11 \pm 6\%$
[54] Continuous house vs face & Discontinuous house (800ms) vs face & Discoutinuous house (400ms) vs face & House vs face	3%	$41 \pm 3\%$	$36 \pm 4\%$	$5 \pm 2\%$
[54] Continuous house vs face & House vs face	4%	$44 \pm 3\%$	$42 \pm 4\%$	$2 \pm 3\%$
[55] The Human Connectome Project	4%	$90 \pm 0\%$	$93 \pm 1\%$	$-2 \pm 0\%$
[56] Face recognition	20%	$69 \pm 6\%$	$63 \pm 6\%$	$6 \pm 7\%$
[57] Arithmetic & Saccades	4%	$38 \pm 2\%$	$34 \pm 3\%$	$5 \pm 3\%$
[58] UCLA LA5C consortium	4%	$62 \pm 1\%$	$64 \pm 1\%$	$-2 \pm 1\%$
[59] Foreign language & Localizer & Saccade	3%	$90 \pm 1\%$	$87 \pm 2\%$	$2 \pm 2\%$
[60] Auditory compression & Visual compression	7%	$61 \pm 3\%$	$53 \pm 4\%$	$8 \pm 3\%$

References

1. Smith SM, Fox PT, Miller KL, Glahn DC, Fox PM, Mackay CE, et al. Correspondence of the Brain's Functional Architecture During Activation and Rest. Proceedings of the National Academy of Sciences. 2009;106(31):13040–13045.
2. Yeo TBT, Krienen FM, Sepulcre J, Sabuncu MR, Lashkari D, Hollinshead M, et al. The Organization of the Human Cerebral Cortex Estimated by Intrinsic Functional Connectivity. Journal of Neurophysiology. 2011;106(3):1125–1165.
3. Nocedal J. Updating Quasi-Newton Matrices with Limited Storage. Mathematics of Computation. 1980;35(151):773–782.
4. Ando RK, Zhang T. A Framework for Learning Predictive Structures from Multiple Tasks and Unlabeled Data. Journal of Machine Learning Research. 2005;6:1817–1853.
5. Xue Y, Liao X, Carin L, Krishnapuram B. Multi-Task Learning for Classification with Dirichlet Process Priors. Journal of Machine Learning Research. 2007;8(Jan):35–63.
6. Pan SJ, Yang Q. A Survey on Transfer Learning. IEEE Transactions on Knowledge and Data Engineering. 2010;22(10):1345–1359.
7. Srivastava N, Hinton GE, Krizhevsky A, Sutskever I, Salakhutdinov R. Dropout: A Simple Way to Prevent Neural Networks from Overfitting. Journal of Machine Learning Research. 2014;15(1):1929–1958.
8. Neyshabur B. Implicit Regularization in Deep Learning [PhD thesis]. Toyota Technological Institute at Chicago; 2017.
9. Kingma DP, Salimans T, Welling M. Variational Dropout and the Local Reparameterization Trick. In: Advances in Neural Information Processing Systems; 2015. p. 2575–2583.
10. Molchanov D, Ashukha A, Vetrov D. Variational Dropout Sparsifies Deep Neural Networks. In: Proceedings of the International Conference on Machine Learning; 2017. p. 2498–2507.
11. Kingma DP, Ba J. Adam: A Method for Stochastic Optimization. In: International Conference for Learning Representations; 2015.
12. Ioffe S, Szegedy C. Batch Normalization: Accelerating Deep Network Training by Reducing Internal Covariate Shift. In: Proceedings of the International Conference on Machine Learning; 2015. p. 448–456.
13. Dadi K, Varoquaux G, Machlouzarides-Shalit A, Gorgolewski KJ, Wassermann D, Thirion B, et al. Fine-grain atlases of functional modes for fMRI analysis. To appear in NeuroImage. 2020;.
14. Mensch A, Mairal J, Thirion B, Varoquaux G. Stochastic Subsampling for Factorizing Huge Matrices. IEEE Transactions on Signal Processing. 2018;66(1):113–128.
15. Papadopoulos Orfanos D, Michel V, Schwartz Y, Pinel P, Moreno A, Le Bihan D, et al. The Brainomics/Localizer Database. NeuroImage. 2017;144:309–314.
16. Van Essen DC, Ugurbil K, Auerbach E, Barch D, Behrens TEJ, Bucholz R, et al. The Human Connectome Project: A Data Acquisition Perspective. NeuroImage. 2012;62(4):2222–2231.
17. Srebro N, Rennie J, Jaakkola TS. Maximum-Margin Matrix Factorization. In: Advances in Neural Information Processing Systems; 2004. p. 1329–1336.
18. Beck A, Teboulle M. A Fast Iterative Shrinkage-Thresholding Algorithm for Linear Inverse Problems. SIAM Journal on Imaging Sciences. 2009;2(1):183–202.
19. Rennie JDM, Srebro N. Fast Maximum Margin Matrix Factorization for Collaborative Prediction. In: Proceedings of the International Conference on Machine Learning; 2005. p. 713–719.
20. Bell RM, Koren Y. Lessons from the Netflix Prize Challenge. ACM SIGKDD Explorations Newsletter. 2007;9(2):75–79.
21. Wager S, Wang S, Liang PS. Dropout Training as Adaptive Regularization. In: Advances in Neural Information Processing Systems; 2013. p. 351–359.
22. Varoquaux G. Cross-Validation Failure: Small Sample Sizes Lead to Large Error Bars. NeuroImage. 2018;180:68–77.
23. Breiman L. Bagging Predictors. Machine Learning. 1996;24(2):123–140.
24. Mensch A, Mairal J, Bzdok D, Thirion B, Varoquaux G. Learning Neural Representations of Human Cognition Across Many fMRI Studies. In: Advances in Neural Information Processing Systems; 2017. p. 5883–5893.
25. Abraham A, Pedregosa F, Eickenberg M, Gervais P, Mueller A, Kossaifi J, et al. Machine Learning for Neuroimaging with Scikit-Learn. Frontiers in Neuroinformatics. 2014;8:14.

26. Pedregosa F, Varoquaux G, Gramfort A, Michel V, Thirion B, Grisel O, et al. Scikit-Learn: Machine Learning in Python. Journal of Machine Learning Research. 2011;12:2825–2830.
27. Paszke A, Gross S, Chintala S, Chanan G. PyTorch: Tensors and Dynamic Neural Networks in Python with Strong GPU Acceleration; 2017.
28. Rizk-Jackson A, Aron AR, Poldrack RA. Classification Learning and Stop-Signal (one Year Test-Retest); 2011. https://openfmri.org/dataset/ds000017.
29. Amalric M, Dehaene S. Origins of the Brain Networks for Advanced Mathematics in Expert Mathematicians. Proceedings of the National Academy of Sciences. 2016;113(18):4909–4917.
30. Pinel P, Thirion B, Meriaux S, Jobert A, Serres J, Bihan DL, et al. Fast Reproducible Identification and Large-Scale Databasing of Individual Functional Cognitive Networks. BMC neuroscience. 2007;8:91.
31. Shafto MA, Tyler LK, Dixon M, Taylor JR, Rowe JB, Cusack R, et al. The Cambridge Centre for Ageing and Neuroscience (Cam-CAN) Study Protocol: A Cross-Sectional, Lifespan, Multidisciplinary Examination of Healthy Cognitive Ageing. BMC Neurology. 2014;14:204.
32. Cauvet E. Traitement des structures syntaxiques dans le langage et dans la musique [PhD thesis]. Paris 6; 2012.
33. Hara N, Cauvet E, Devauchelle AD, Dehaene S, Pallier C, et al. Neural Correlates of Constituent Structure in Language and Music. NeuroImage. 2009;47:S143.
34. Devauchelle AD, Oppenheim C, Rizzi L, Dehaene S, Pallier C. Sentence Syntax and Content in the Human Temporal Lobe: An fMRI Adaptation Study in Auditory and Visual Modalities. Journal of Cognitive Neuroscience. 2009;21(5):1000–1012.
35. Schonberg T, Fox C, Mumford JA, Congdon C, Trepel C, Poldrack RA. Decreasing Ventromedial Prefrontal Cortex Activity During Sequential Risk-Taking: An fMRI Investigation of the Balloon Analog Risk Task. Frontiers in Neuroscience. 2012;6:80.
36. Aron AR, Gluck M, Poldrack RA. Long-Term Test–Retest Reliability of Functional MRI in a Classification Learning Task. NeuroImage. 2006;29:1000–1006.
37. Xue G, Poldrack RA. The Neural Substrates of Visual Perceptual Learning of Words: Implications for the Visual Word Form Area Hypothesis. Journal of Cognitive Neuroscience. 2007;19:1643–1655.
38. Tom SM, Fox CR, Trepel C, Poldrack RA. The Neural Basis of Loss Aversion in Decision-Making Under Risk. Science. 2007;315(5811):515–518.
39. Jimura K, Cazalis F, Stover ERS, Poldrack RA. The Neural Basis of Task Switching Changes with Skill Acquisition. Frontiers in Human Neuroscience. 2014;8.
40. Xue G, Aron AR, Poldrack RA. Common Neural Substrates for Inhibition of Spoken and Manual Responses. Cerebral Cortex. 2008;18:1923–1932.
41. Aron AR, Behrens TE, Smith S, Frank MJ, Poldrack RA. Triangulating a Cognitive Control Network Using Diffusion-Weighted Magnetic Resonance Imaging (MRI) and Functional MRI. The Journal of Neuroscience. 2007;27:3743–3752.
42. Cohen JR. The Development and Generality of Self-Control [PhD thesis]. University of the City of Los Angeles; 2009.
43. Foerde K, Knowlton B, Poldrack RA. Modulation of Competing Memory Systems by Distraction. Proceedings of the National Academy of Science. 2006;103:11778–11783.
44. Alvarez RP, Jasdzewski G, Poldrack RA. Building Memories in Two Languages: An fMRI Study of Episodic Encoding in Bilinguals. In: Society for Neuroscience Abstracts; 2002. p. 179.12.
45. Poldrack RA, Clark J, Pare-Blagoev E, Shohamy D, Creso Moyano J, Myers C, et al. Interactive Memory Systems in the Human Brain. Nature. 2001;414(6863):546–550.
46. Kelly A, Milham M. Simon Task; 2011. https://openfmri.org/dataset/ds000101.
47. Haxby JV, Gobbini IM, Furey ML, Ishai A, Schouten JL, Pietrini P. Distributed and Overlapping Representations of Faces and Objects in Ventral Temporal Cortex. Science. 2001;293(5539):2425–2430.
48. Duncan K, Pattamadilok C, Knierim I, Devlin J. Consistency and Variability in Functional Localisers. NeuroImage. 2009;46:1018–1026.
49. Wager TD, Davidson ML, Hughes BL, Lindquist MA, Ochsner KN. Prefrontal-Subcortical Pathways Mediating Successful Emotion Regulation. Neuron. 2008;59:1037–1050.
50. Moran JM, Jolly E, Mitchell JP. Social-Cognitive Deficits in Normal Aging. The Journal of Neuroscience. 2012;32:5553–5561.

51. Uncapher MR, Hutchinson JB, Wagner AD. Dissociable Effects of Top-Down and Bottom-Up Attention During Episodic Encoding. The Journal of Neuroscience: The Official Journal of the Society for Neuroscience. 2011;31(35):12613–12628.

52. Gorgolewski KJ, Storkey A, Bastin ME, Whittle IR, Wardlaw JM, Pernet CR. A Test-Retest fMRI Dataset for Motor, Language and Spatial Attention Functions. GigaScience. 2013;2(1):6.

53. Collier AK, Wolf DH, Valdez JN, Turetsky BI, Elliott MA, Gur RE, et al. Comparison of Auditory and Visual Oddball fMRI in Schizophrenia. Schizophrenia research. 2014;158:183–188.

54. Gauthier B, Eger E, Hesselmann G, Giraud AL, Kleinschmidt A. Temporal Tuning Properties Along the Human Ventral Visual Stream. The Journal of Neuroscience. 2012;32:14433–14441.

55. Barch DM, Burgess GC, Harms MP, Petersen SE, Schlaggar BL, Corbetta M, et al. Function in the Human Connectome: Task-fMRI and Individual Differences in Behavior. NeuroImage. 2013;80:169–189.

56. Henson RN, Wakeman DG, Litvak V, Friston KJ. A Parametric Empirical Bayesian Framework for the EEG/MEG Inverse Problem: Generative Models for Multi-Subject and Multi-Modal Integration. Frontiers in Human Neuroscience. 2011;5.

57. Knops A, Thirion B, Hubbard EM, Michel V, Dehaene S. Recruitment of an Area Involved in Eye Movements During Mental Arithmetic. Science. 2009;324:1583–1585.

58. Poldrack RA, Congdon E, Triplett W, Gorgolewski KJ, Karlsgodt K, Mumford JA, et al. A Phenome-Wide Examination of Neural and Cognitive Function. Scientific Data. 2016;3:160110.

59. Pinel P, Dehaene S. Genetic and Environmental Contributions to Brain Activation During Calculation. NeuroImage. 2013;81:306–316.

60. Vagharchakian L, Dehaene-Lambertz G, Pallier C, Dehaene S. A Temporal Bottleneck in the Language Comprehension Network. The Journal of Neuroscience. 2012;32:9089–9102.
