# Supplementary material for: Extracting representations of cognition across neuroimaging studies improves brain decoding
Source: PLoS Comput Biol. 2021 May 3;17(5):e1008795. doi: 10.1371/journal.pcbi.1008795 (PMC8118532; doi:10.1371/journal.pcbi.1008795)
Supplement: S1 Components — (ZIP) [file pcbi.1008795.s002.zip › components/components.html]

# MSTON Network-task associations

## components\_0


## components\_1


## components\_2


## components\_3


## components\_4


## components\_5


## components\_6


## components\_7


## components\_8


## components\_9


## components\_10


## components\_11


## components\_12


## components\_13


## components\_14


## components\_15


## components\_16


## components\_17


## components\_18


## components\_19


## components\_20


## components\_21


## components\_22


## components\_23


## components\_24


## components\_25


## components\_26


## components\_27


## components\_28


## components\_29


## components\_30


## components\_31


## components\_32


## components\_33


## components\_34


## components\_35


## components\_36


## components\_37


## components\_38


## components\_39


## components\_40


## components\_41


## components\_42


## components\_43


## components\_44


## components\_45


## components\_46


## components\_47


## components\_48


## components\_49


## components\_50


## components\_51


## components\_52


## components\_53


## components\_54


## components\_55


## components\_56


## components\_57


## components\_58


## components\_59


## components\_60


## components\_61


## components\_62


## components\_63


## components\_64


## components\_65


## components\_66


## components\_67


## components\_68


## components\_69


## components\_70


## components\_71


## components\_72


## components\_73


## components\_74


## components\_75


## components\_76


## components\_77


## components\_78


## components\_79


## components\_80


## components\_81


## components\_82


## components\_83


## components\_84


## components\_85


## components\_86


## components\_87


## components\_88


## components\_89


## components\_90


## components\_91


## components\_92


## components\_93


## components\_94


## components\_95


## components\_96


## components\_97


## components\_98


## components\_99


## components\_100


## components\_101


## components\_102


## components\_103


## components\_104


## components\_105


## components\_106


## components\_107


## components\_108


## components\_109


## components\_110


## components\_111


## components\_112


## components\_113


## components\_114


## components\_115


## components\_116


## components\_117


## components\_118


## components\_119


## components\_120


## components\_121


## components\_122


## components\_123


## components\_124


## components\_125


## components\_126


## components\_127
