# Supplementary figures and images for: Extracting representations of cognition across neuroimaging studies improves brain decoding (part 2 of 2)
Source: PLoS Comput Biol. 2021 May 3;17(5):e1008795. doi: 10.1371/journal.pcbi.1008795 (PMC8118532; doi:10.1371/journal.pcbi.1008795)

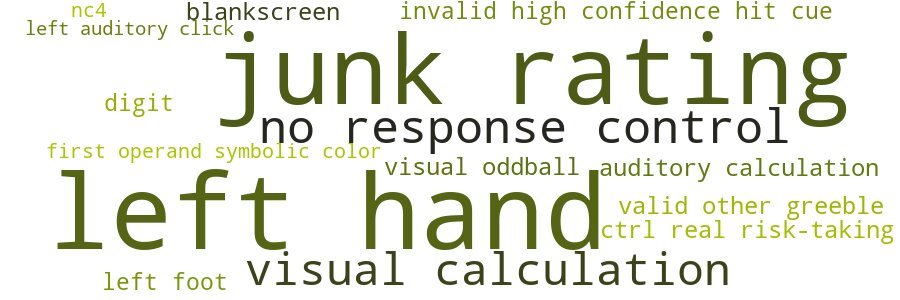

Supplement: S1 Components — (ZIP) [file pcbi.1008795.s002.zip › components/components_files/wc_cat_103.jpg]

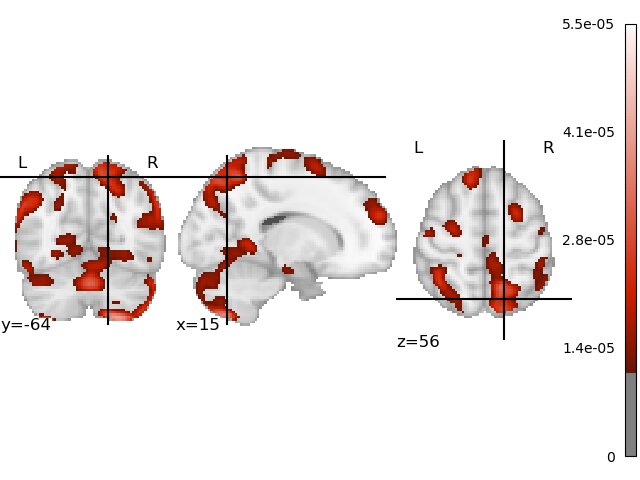

Supplement: S1 Components — (ZIP) [file pcbi.1008795.s002.zip › components/components_files/components_105_stat_map.jpg]

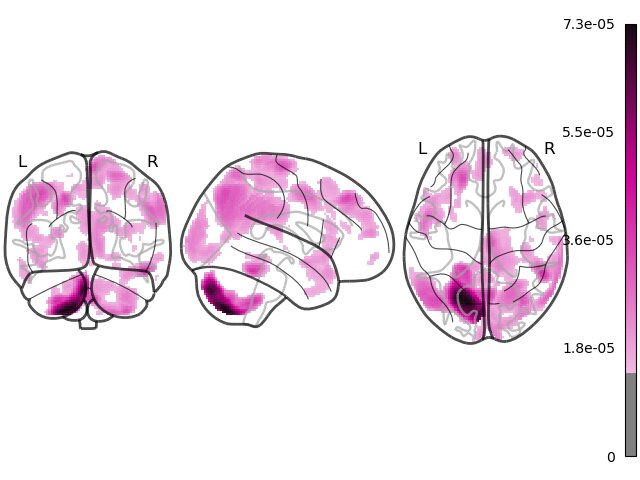

Supplement: S1 Components — (ZIP) [file pcbi.1008795.s002.zip › components/components_files/components_29_glass_brain.jpg]

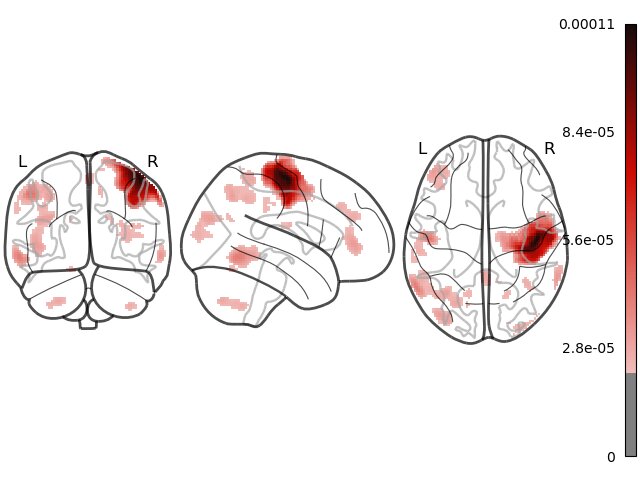

Supplement: S1 Components — (ZIP) [file pcbi.1008795.s002.zip › components/components_files/components_14_glass_brain.jpg]

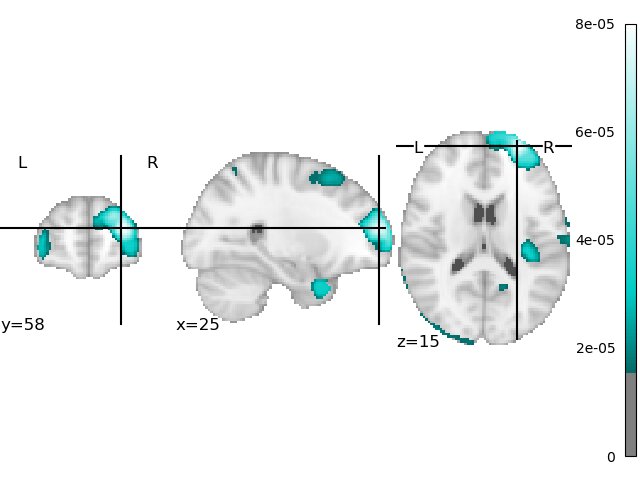

Supplement: S1 Components — (ZIP) [file pcbi.1008795.s002.zip › components/components_files/components_102_stat_map.jpg]

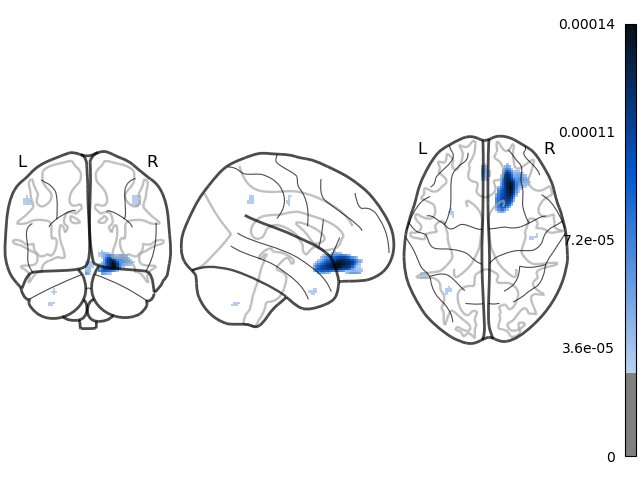

Supplement: S1 Components — (ZIP) [file pcbi.1008795.s002.zip › components/components_files/components_0_glass_brain.jpg]

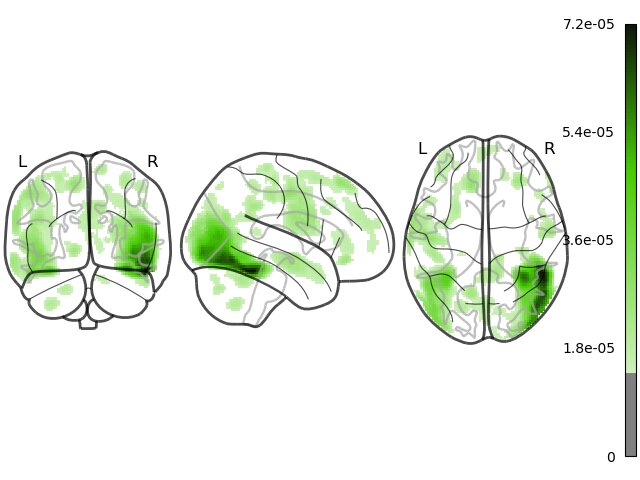

Supplement: S1 Components — (ZIP) [file pcbi.1008795.s002.zip › components/components_files/components_30_glass_brain.jpg]

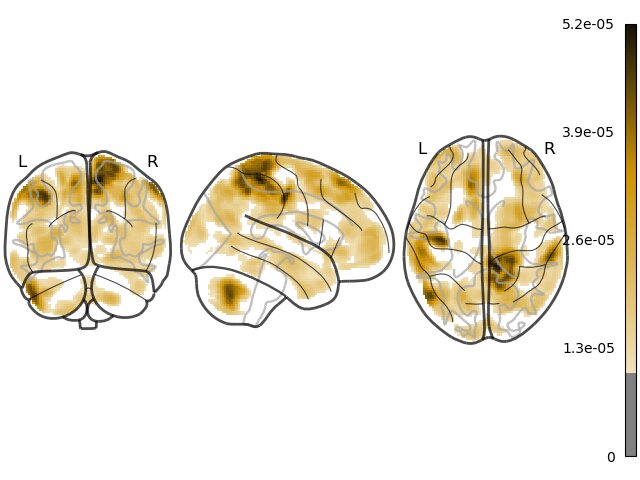

Supplement: S1 Components — (ZIP) [file pcbi.1008795.s002.zip › components/components_files/components_96_glass_brain.jpg]

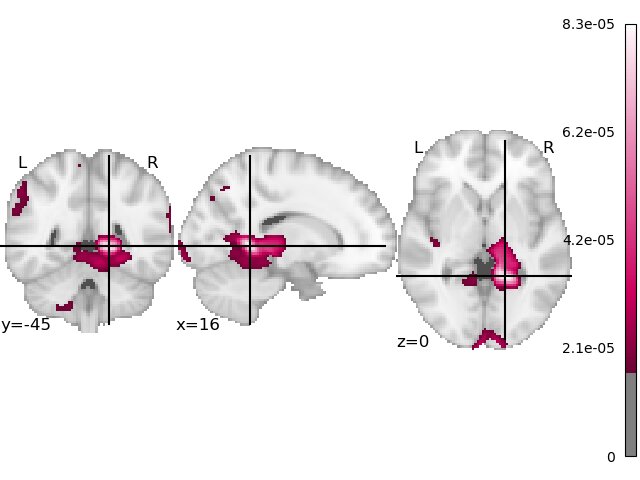

Supplement: S1 Components — (ZIP) [file pcbi.1008795.s002.zip › components/components_files/components_48_stat_map.jpg]

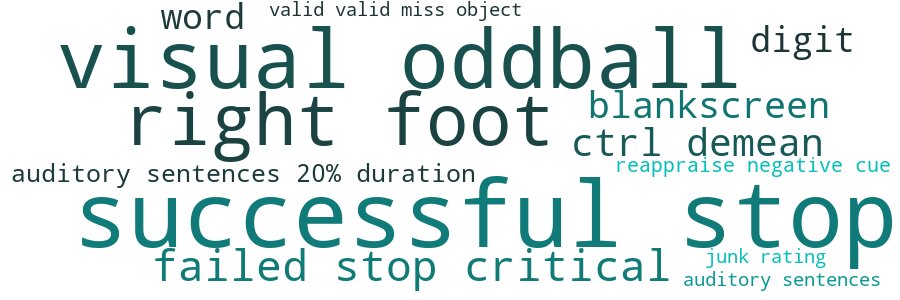

Supplement: S1 Components — (ZIP) [file pcbi.1008795.s002.zip › components/components_files/wc_cat_102.jpg]

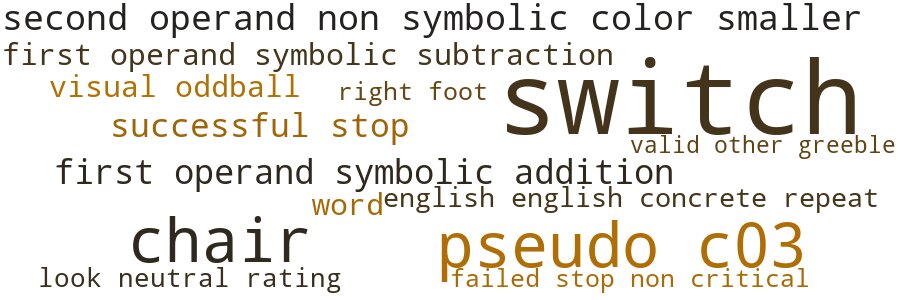

Supplement: S1 Components — (ZIP) [file pcbi.1008795.s002.zip › components/components_files/wc_cat_116.jpg]

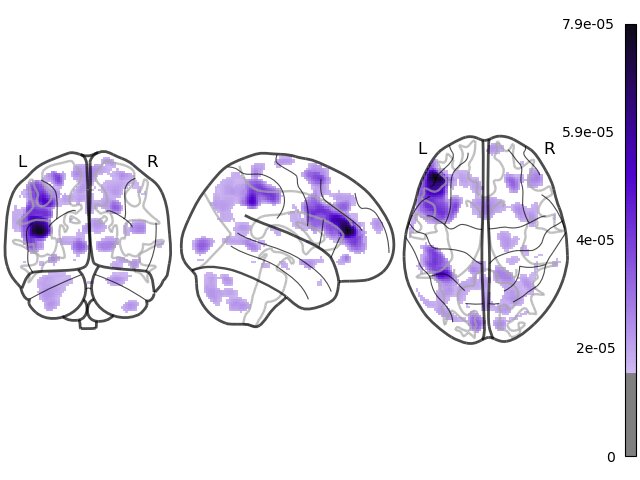

Supplement: S1 Components — (ZIP) [file pcbi.1008795.s002.zip › components/components_files/components_77_glass_brain.jpg]

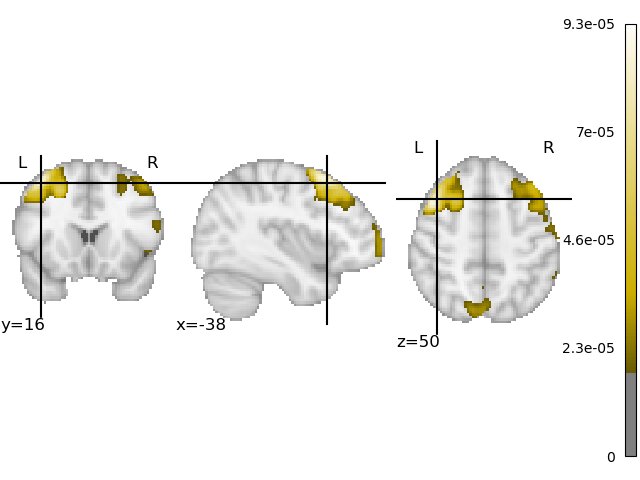

Supplement: S1 Components — (ZIP) [file pcbi.1008795.s002.zip › components/components_files/components_117_stat_map.jpg]

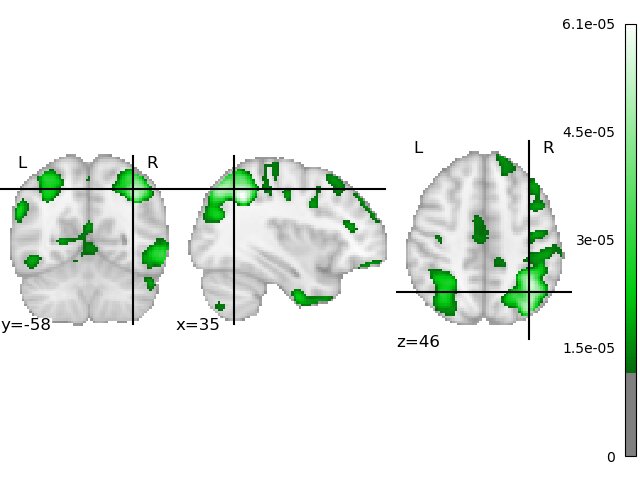

Supplement: S1 Components — (ZIP) [file pcbi.1008795.s002.zip › components/components_files/components_110_stat_map.jpg]

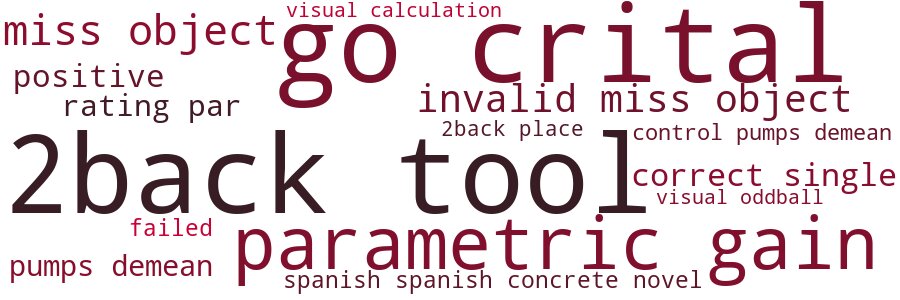

Supplement: S1 Components — (ZIP) [file pcbi.1008795.s002.zip › components/components_files/wc_cat_1.jpg]

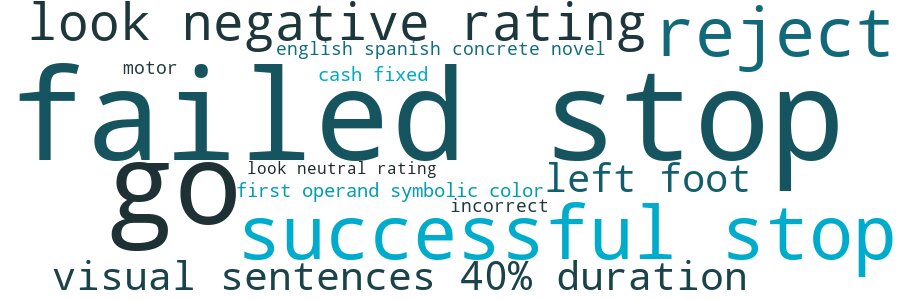

Supplement: S1 Components — (ZIP) [file pcbi.1008795.s002.zip › components/components_files/wc_cat_13.jpg]

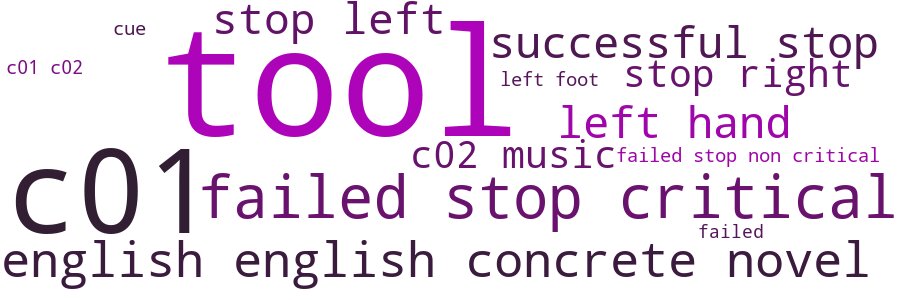

Supplement: S1 Components — (ZIP) [file pcbi.1008795.s002.zip › components/components_files/wc_cat_39.jpg]

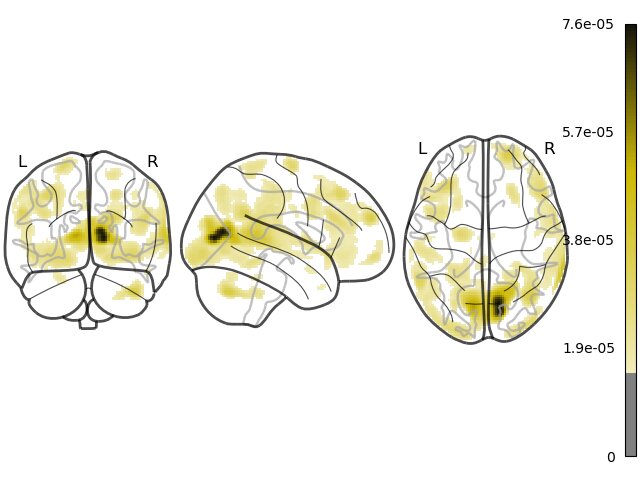

Supplement: S1 Components — (ZIP) [file pcbi.1008795.s002.zip › components/components_files/components_100_glass_brain.jpg]

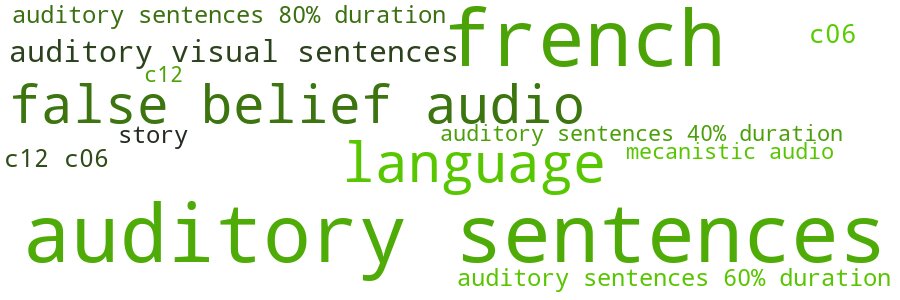

Supplement: S1 Components — (ZIP) [file pcbi.1008795.s002.zip › components/components_files/wc_cat_11.jpg]

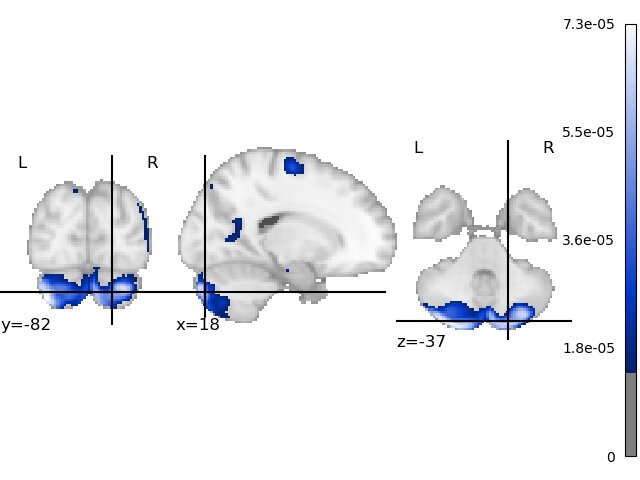

Supplement: S1 Components — (ZIP) [file pcbi.1008795.s002.zip › components/components_files/components_79_stat_map.jpg]

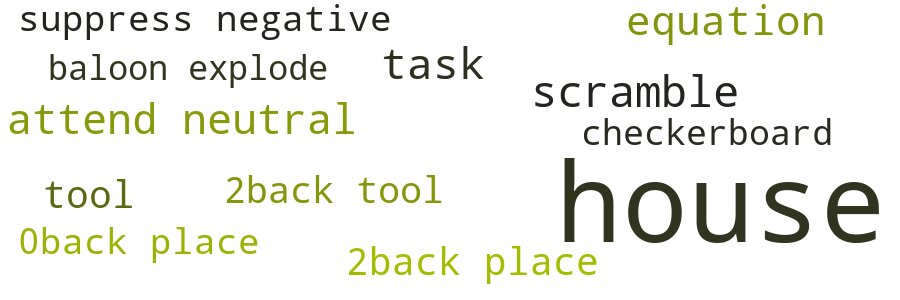

Supplement: S1 Components — (ZIP) [file pcbi.1008795.s002.zip › components/components_files/wc_cat_3.jpg]

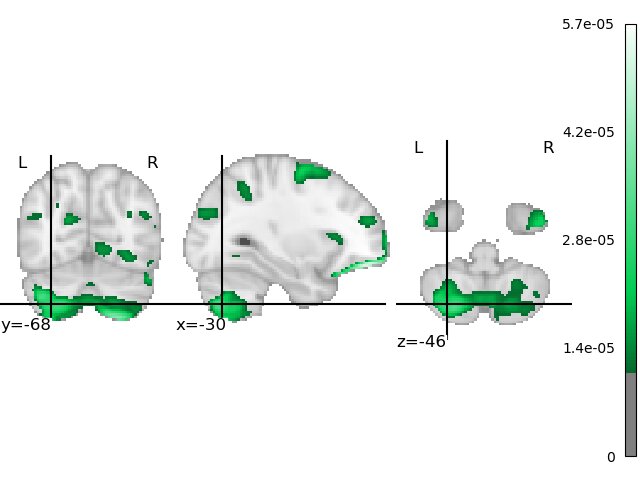

Supplement: S1 Components — (ZIP) [file pcbi.1008795.s002.zip › components/components_files/components_86_stat_map.jpg]

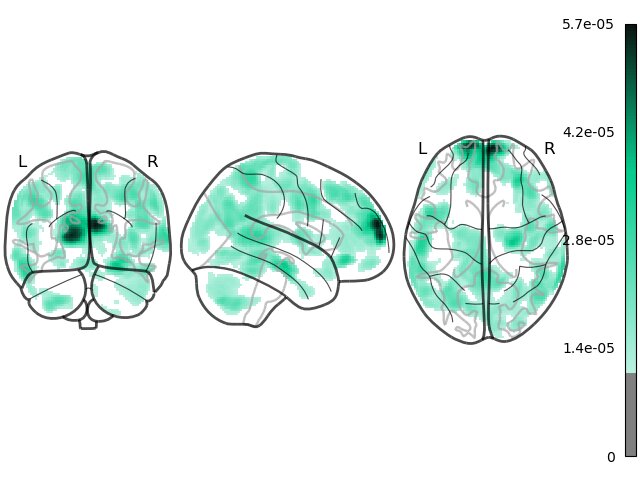

Supplement: S1 Components — (ZIP) [file pcbi.1008795.s002.zip › components/components_files/components_99_glass_brain.jpg]

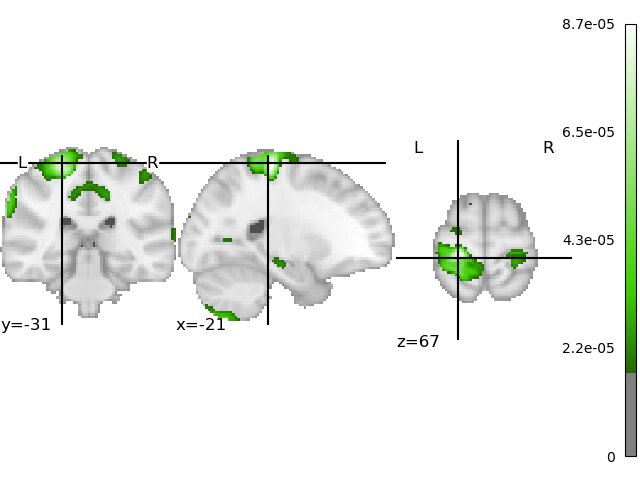

Supplement: S1 Components — (ZIP) [file pcbi.1008795.s002.zip › components/components_files/components_81_stat_map.jpg]

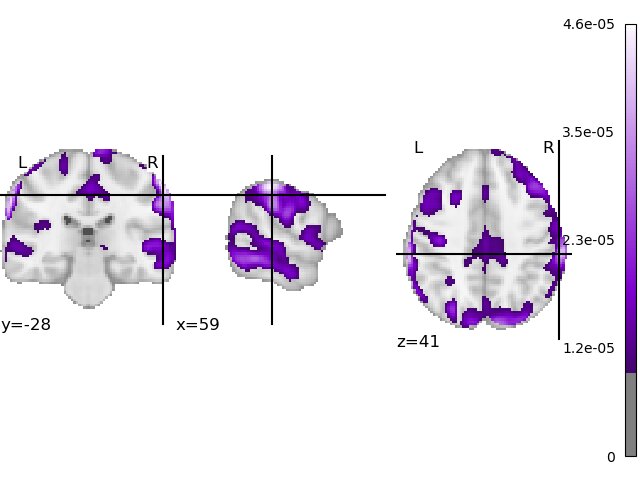

Supplement: S1 Components — (ZIP) [file pcbi.1008795.s002.zip › components/components_files/components_126_stat_map.jpg]

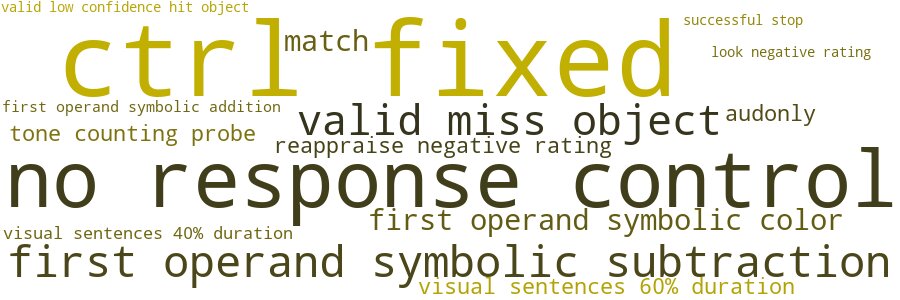

Supplement: S1 Components — (ZIP) [file pcbi.1008795.s002.zip › components/components_files/wc_cat_100.jpg]

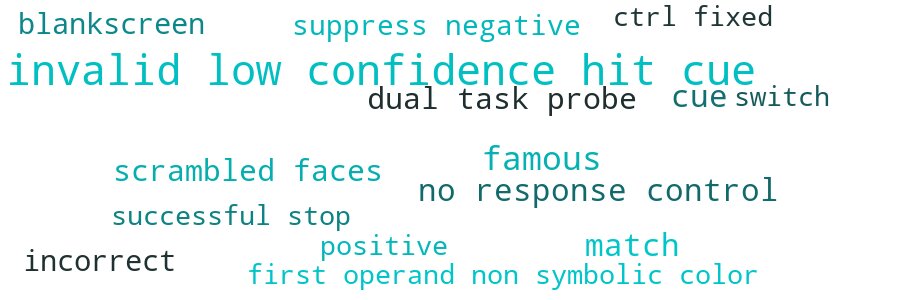

Supplement: S1 Components — (ZIP) [file pcbi.1008795.s002.zip › components/components_files/wc_cat_114.jpg]

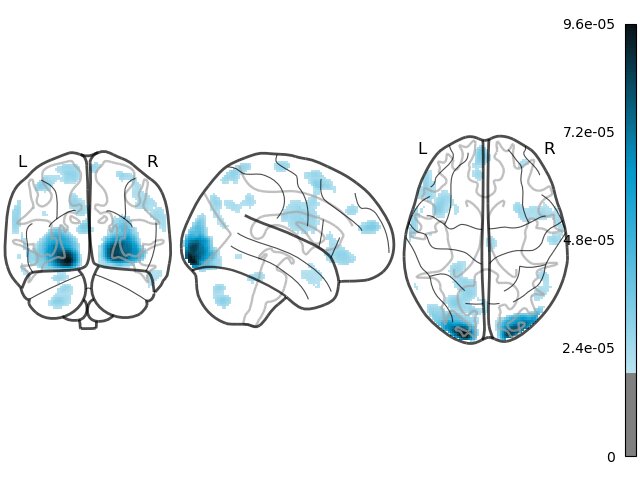

Supplement: S1 Components — (ZIP) [file pcbi.1008795.s002.zip › components/components_files/components_45_glass_brain.jpg]

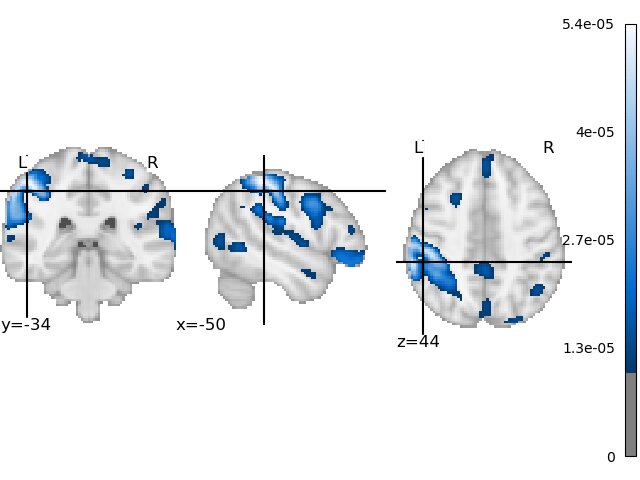

Supplement: S1 Components — (ZIP) [file pcbi.1008795.s002.zip › components/components_files/components_121_stat_map.jpg]

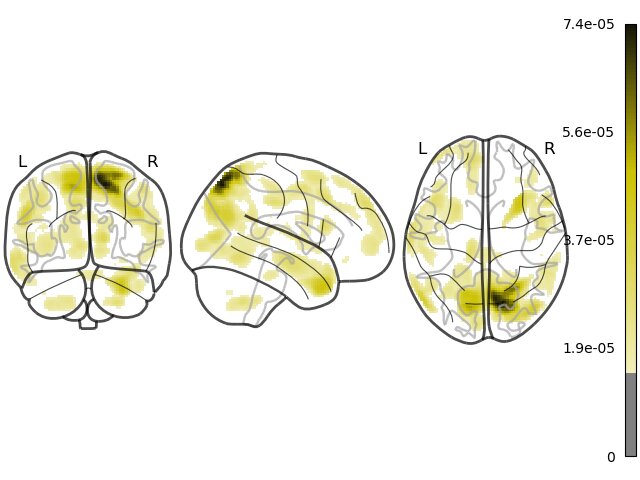

Supplement: S1 Components — (ZIP) [file pcbi.1008795.s002.zip › components/components_files/components_78_glass_brain.jpg]

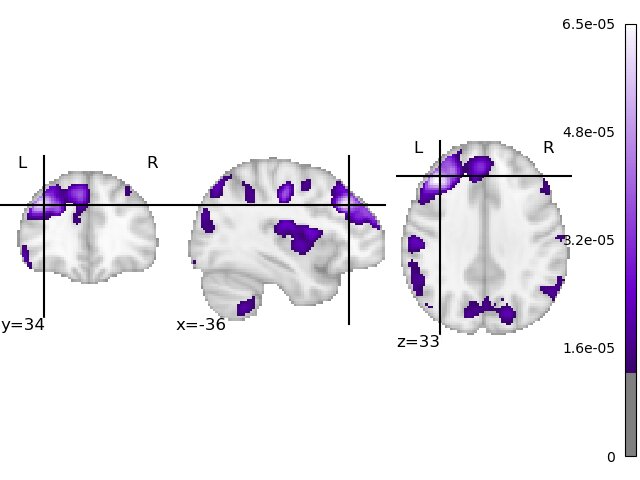

Supplement: S1 Components — (ZIP) [file pcbi.1008795.s002.zip › components/components_files/components_94_stat_map.jpg]

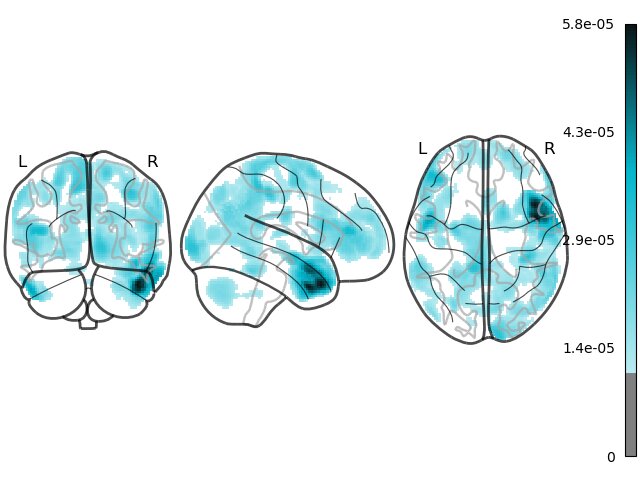

Supplement: S1 Components — (ZIP) [file pcbi.1008795.s002.zip › components/components_files/components_61_glass_brain.jpg]

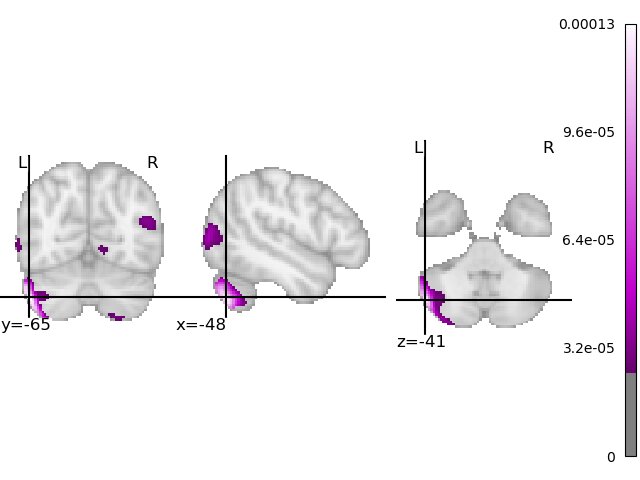

Supplement: S1 Components — (ZIP) [file pcbi.1008795.s002.zip › components/components_files/components_39_stat_map.jpg]

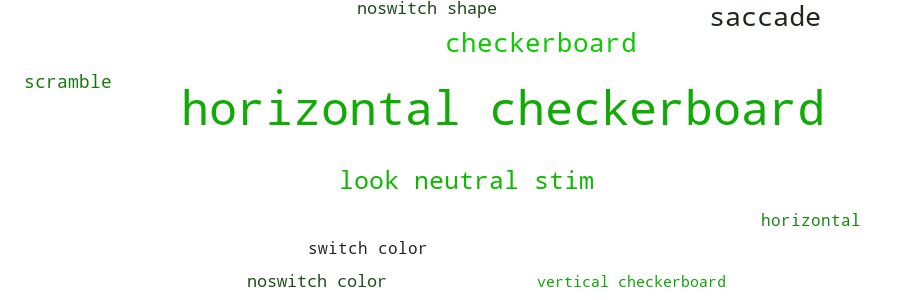

Supplement: S1 Components — (ZIP) [file pcbi.1008795.s002.zip › components/components_files/wc_cat_115.jpg]

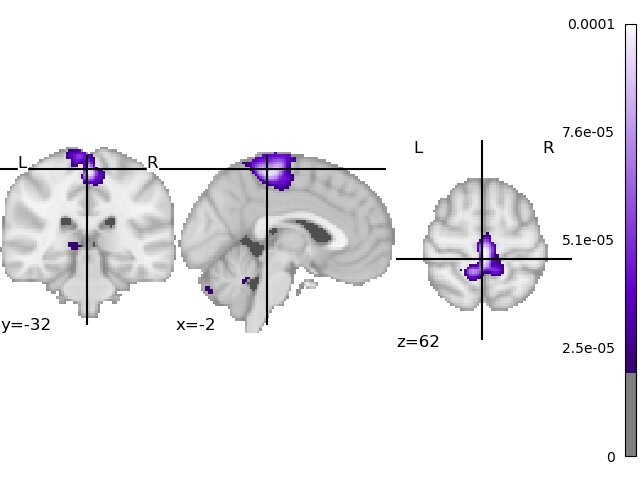

Supplement: S1 Components — (ZIP) [file pcbi.1008795.s002.zip › components/components_files/components_93_stat_map.jpg]

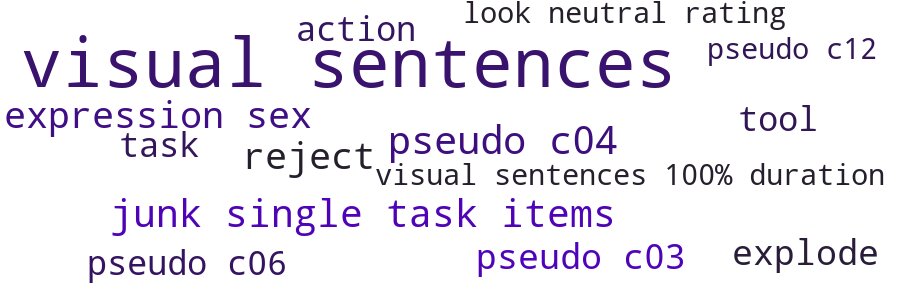

Supplement: S1 Components — (ZIP) [file pcbi.1008795.s002.zip › components/components_files/wc_cat_101.jpg]

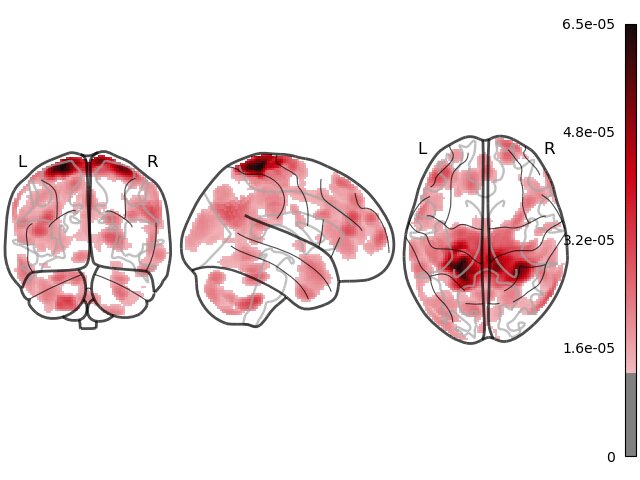

Supplement: S1 Components — (ZIP) [file pcbi.1008795.s002.zip › components/components_files/components_80_glass_brain.jpg]

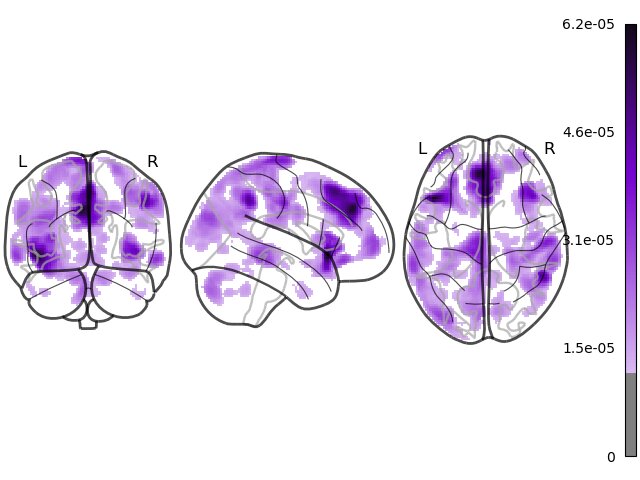

Supplement: S1 Components — (ZIP) [file pcbi.1008795.s002.zip › components/components_files/components_26_glass_brain.jpg]

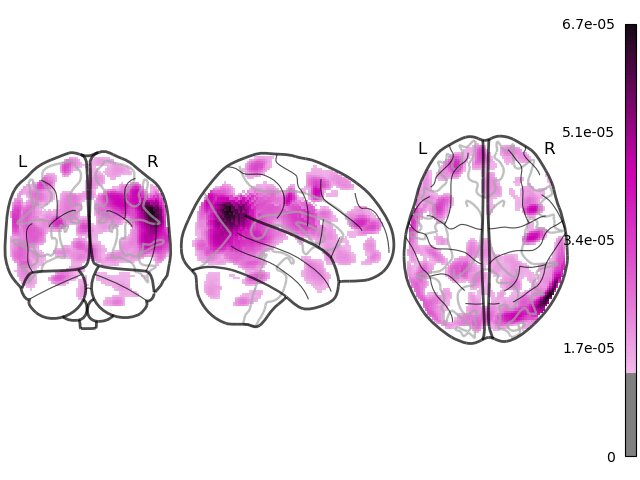

Supplement: S1 Components — (ZIP) [file pcbi.1008795.s002.zip › components/components_files/components_119_glass_brain.jpg]

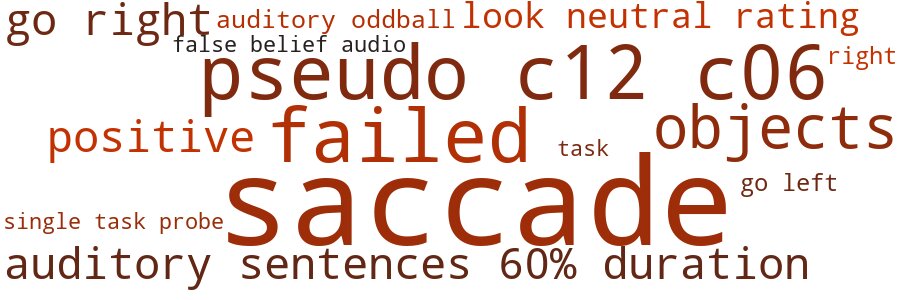

Supplement: S1 Components — (ZIP) [file pcbi.1008795.s002.zip › components/components_files/wc_cat_2.jpg]

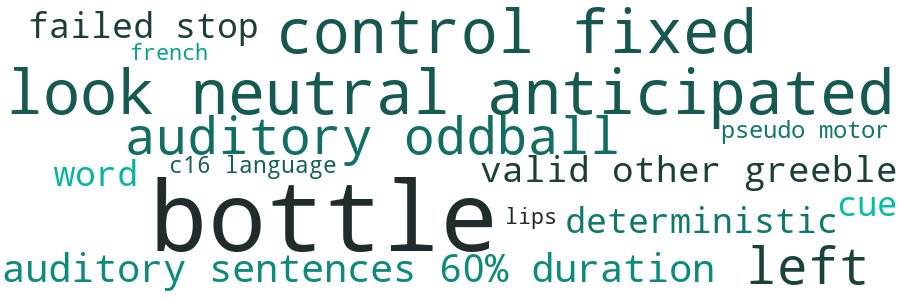

Supplement: S1 Components — (ZIP) [file pcbi.1008795.s002.zip › components/components_files/wc_cat_10.jpg]

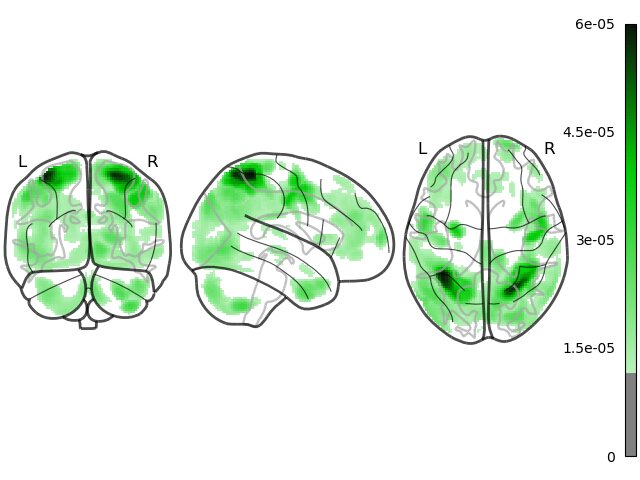

Supplement: S1 Components — (ZIP) [file pcbi.1008795.s002.zip › components/components_files/components_124_glass_brain.jpg]

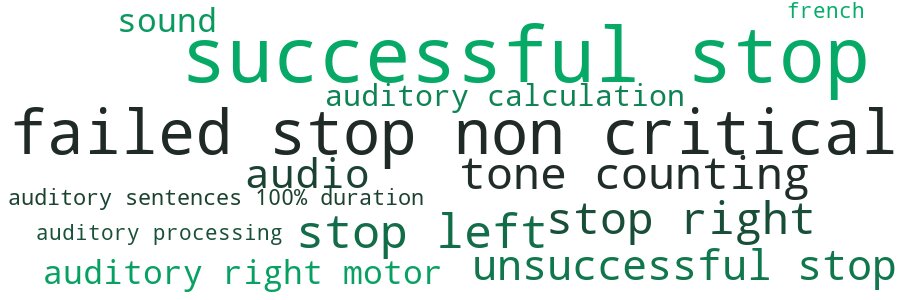

Supplement: S1 Components — (ZIP) [file pcbi.1008795.s002.zip › components/components_files/wc_cat_38.jpg]

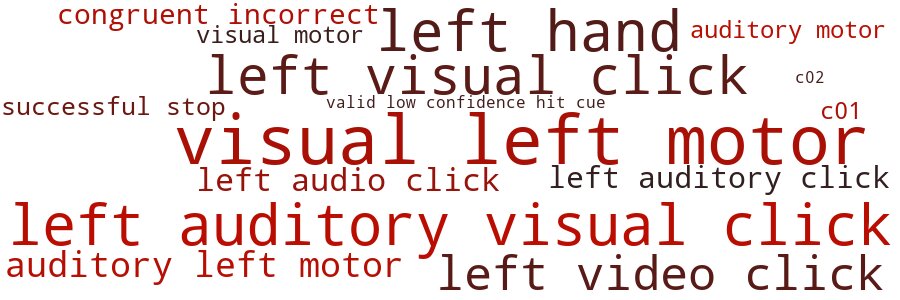

Supplement: S1 Components — (ZIP) [file pcbi.1008795.s002.zip › components/components_files/wc_cat_14.jpg]

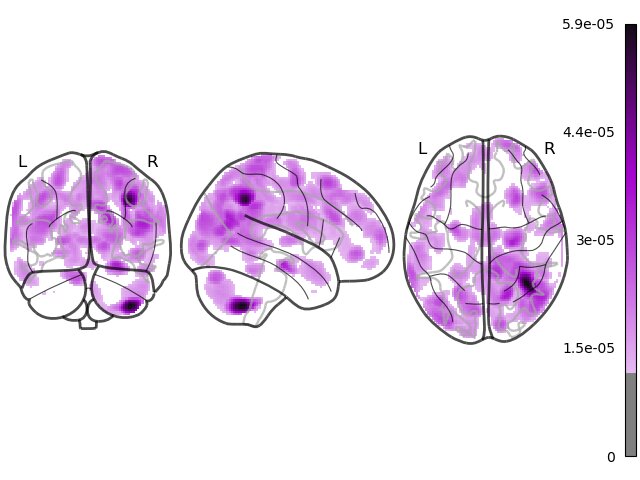

Supplement: S1 Components — (ZIP) [file pcbi.1008795.s002.zip › components/components_files/components_123_glass_brain.jpg]

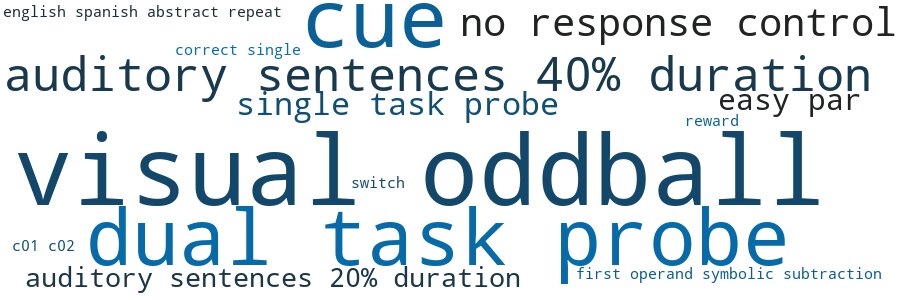

Supplement: S1 Components — (ZIP) [file pcbi.1008795.s002.zip › components/components_files/wc_cat_28.jpg]

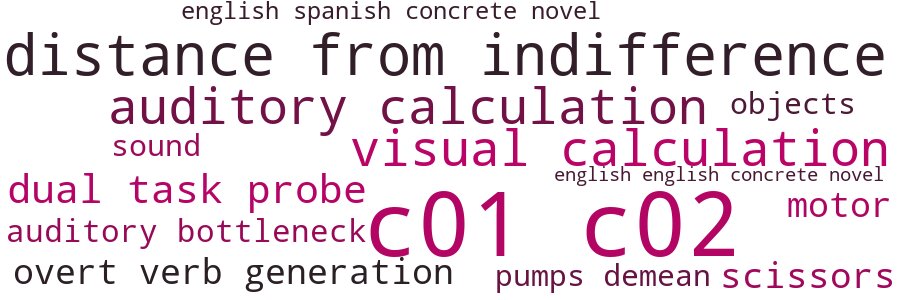

Supplement: S1 Components — (ZIP) [file pcbi.1008795.s002.zip › components/components_files/wc_cat_6.jpg]

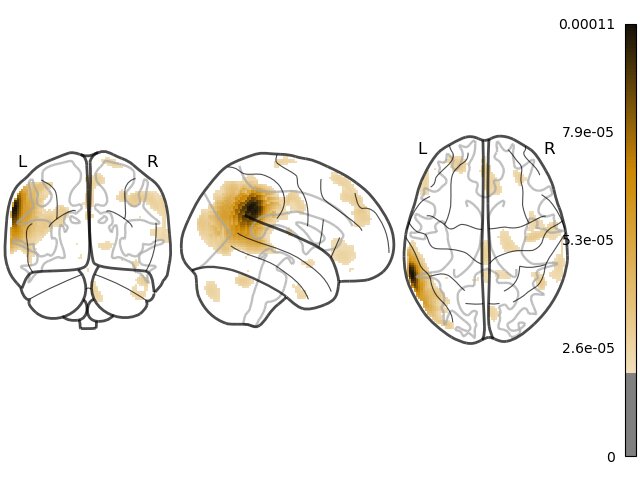

Supplement: S1 Components — (ZIP) [file pcbi.1008795.s002.zip › components/components_files/components_21_glass_brain.jpg]

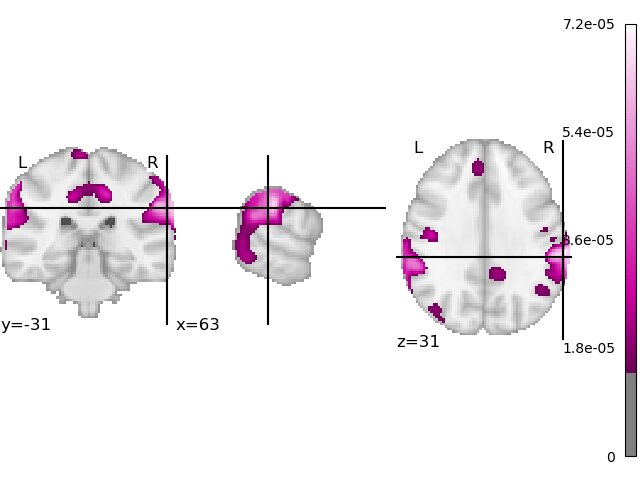

Supplement: S1 Components — (ZIP) [file pcbi.1008795.s002.zip › components/components_files/components_49_stat_map.jpg]

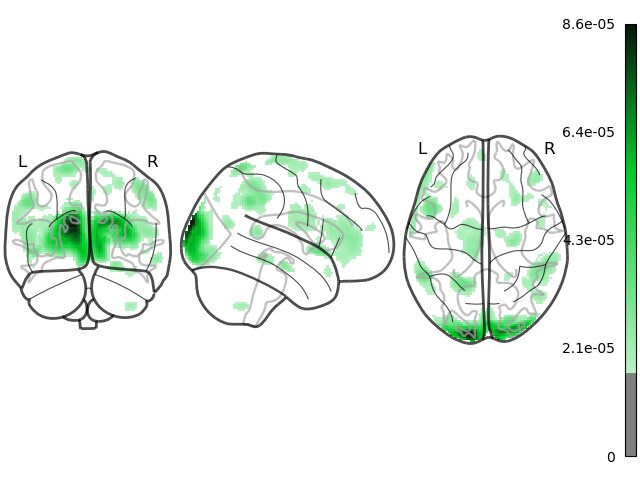

Supplement: S1 Components — (ZIP) [file pcbi.1008795.s002.zip › components/components_files/components_87_glass_brain.jpg]

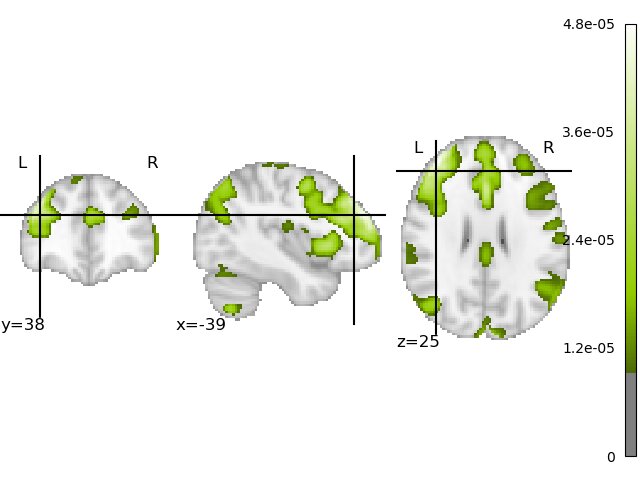

Supplement: S1 Components — (ZIP) [file pcbi.1008795.s002.zip › components/components_files/components_111_stat_map.jpg]

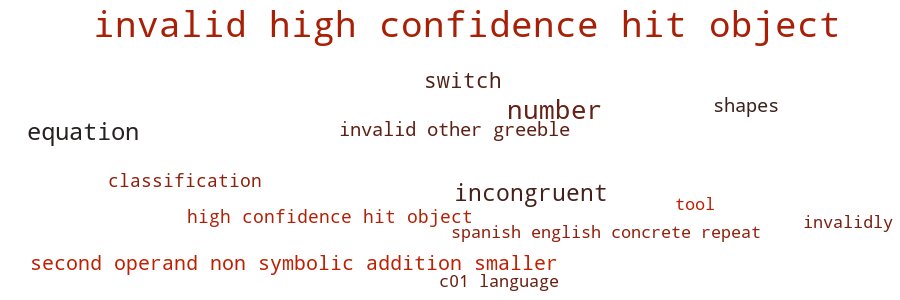

Supplement: S1 Components — (ZIP) [file pcbi.1008795.s002.zip › components/components_files/wc_cat_105.jpg]

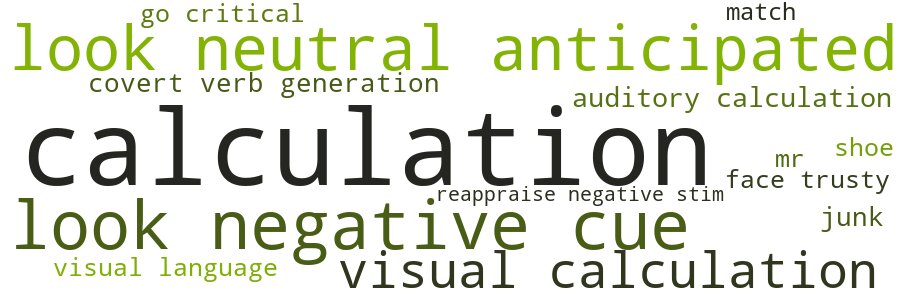

Supplement: S1 Components — (ZIP) [file pcbi.1008795.s002.zip › components/components_files/wc_cat_111.jpg]

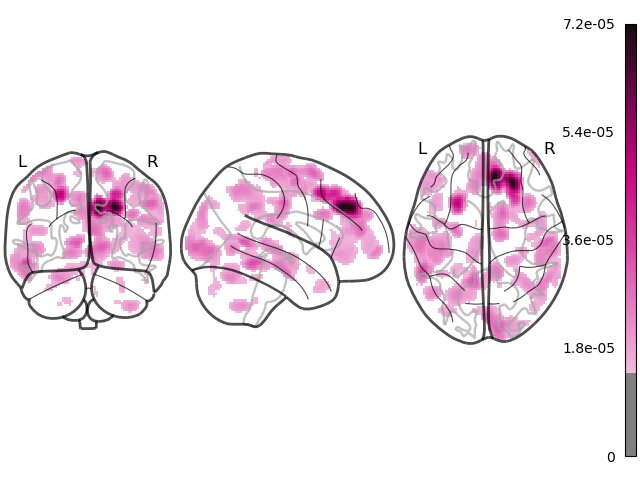

Supplement: S1 Components — (ZIP) [file pcbi.1008795.s002.zip › components/components_files/components_66_glass_brain.jpg]

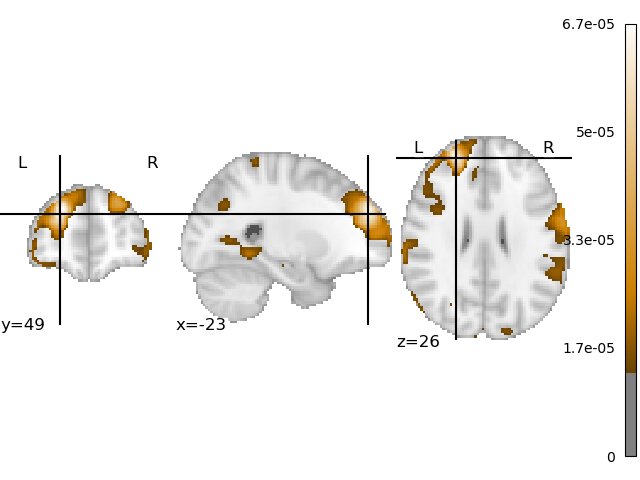

Supplement: S1 Components — (ZIP) [file pcbi.1008795.s002.zip › components/components_files/components_116_stat_map.jpg]

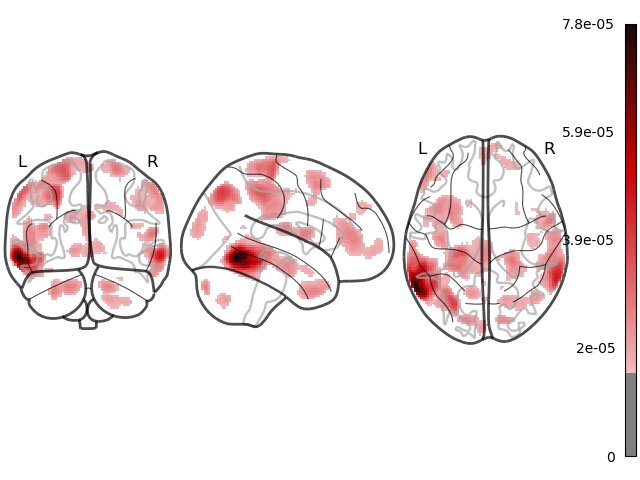

Supplement: S1 Components — (ZIP) [file pcbi.1008795.s002.zip › components/components_files/components_42_glass_brain.jpg]

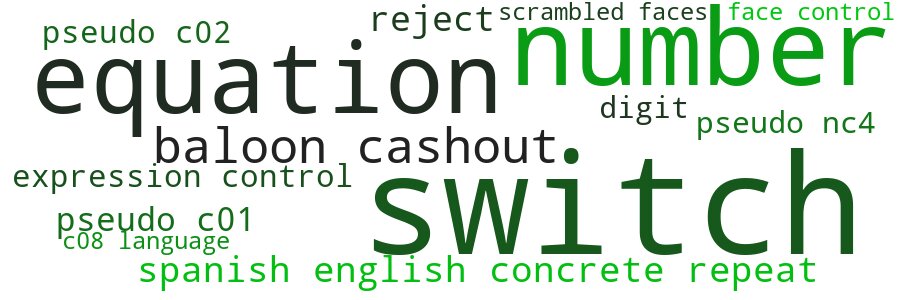

Supplement: S1 Components — (ZIP) [file pcbi.1008795.s002.zip › components/components_files/wc_cat_110.jpg]

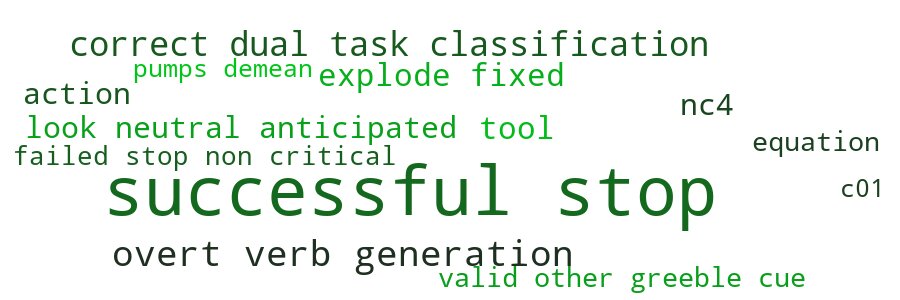

Supplement: S1 Components — (ZIP) [file pcbi.1008795.s002.zip › components/components_files/wc_cat_104.jpg]

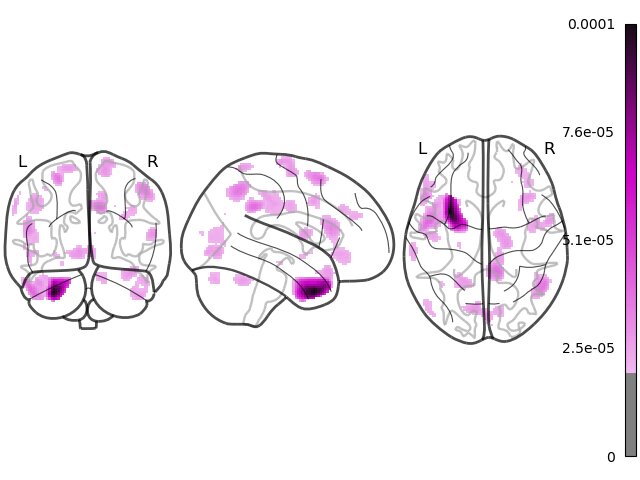

Supplement: S1 Components — (ZIP) [file pcbi.1008795.s002.zip › components/components_files/components_8_glass_brain.jpg]

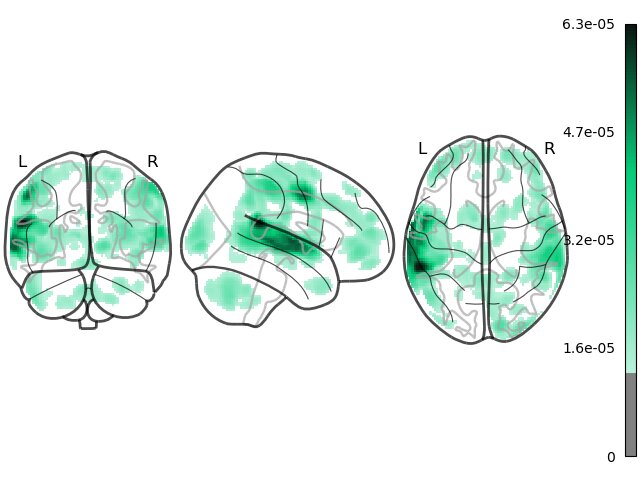

Supplement: S1 Components — (ZIP) [file pcbi.1008795.s002.zip › components/components_files/components_38_glass_brain.jpg]

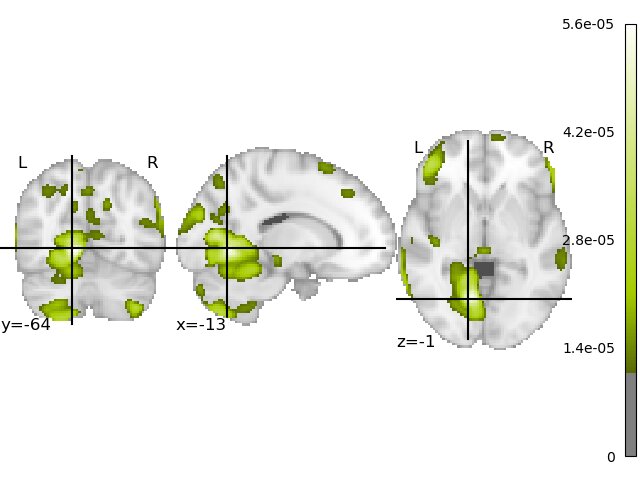

Supplement: S1 Components — (ZIP) [file pcbi.1008795.s002.zip › components/components_files/components_103_stat_map.jpg]

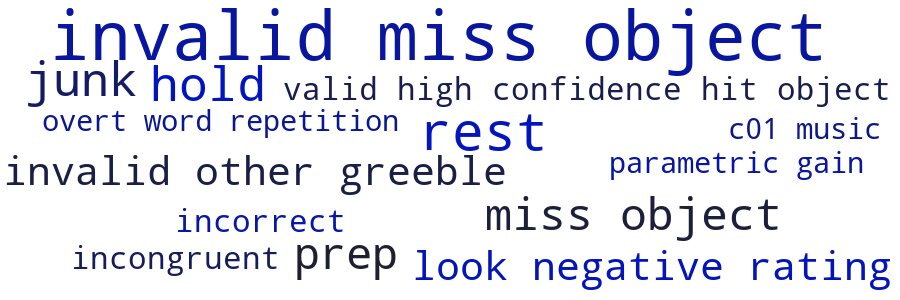

Supplement: S1 Components — (ZIP) [file pcbi.1008795.s002.zip › components/components_files/wc_cat_7.jpg]

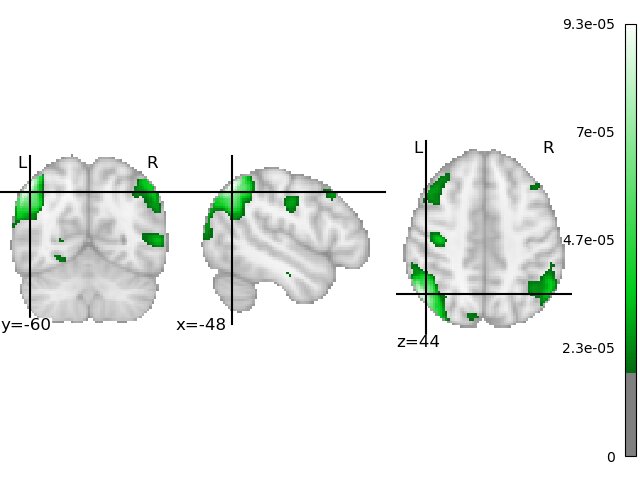

Supplement: S1 Components — (ZIP) [file pcbi.1008795.s002.zip › components/components_files/components_104_stat_map.jpg]

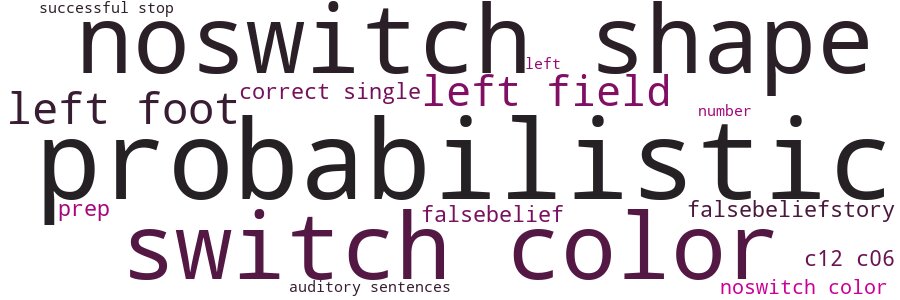

Supplement: S1 Components — (ZIP) [file pcbi.1008795.s002.zip › components/components_files/wc_cat_29.jpg]

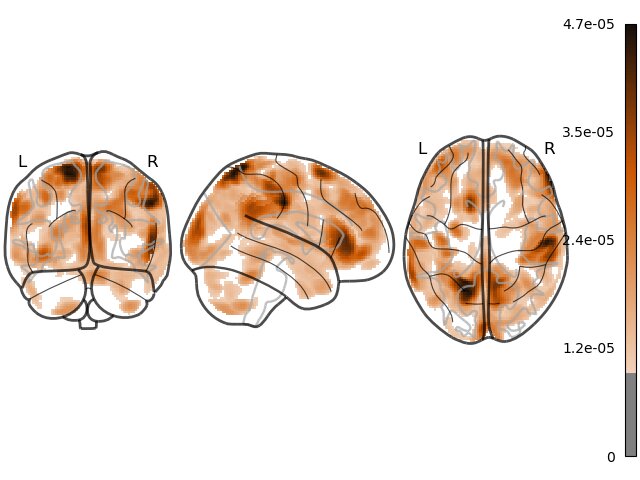

Supplement: S1 Components — (ZIP) [file pcbi.1008795.s002.zip › components/components_files/components_107_glass_brain.jpg]

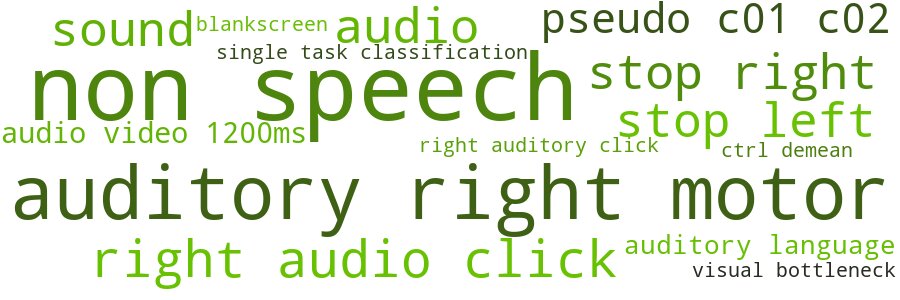

Supplement: S1 Components — (ZIP) [file pcbi.1008795.s002.zip › components/components_files/wc_cat_15.jpg]

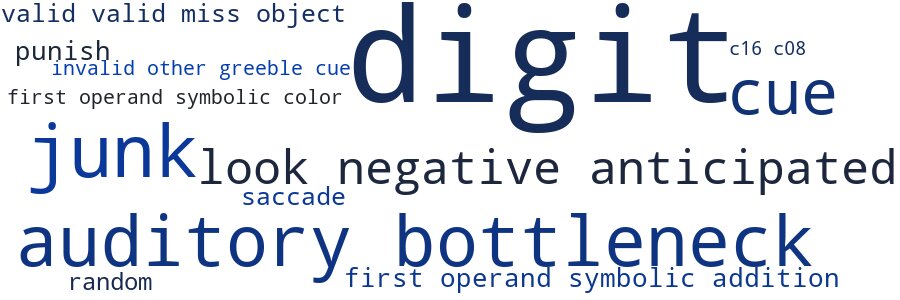

Supplement: S1 Components — (ZIP) [file pcbi.1008795.s002.zip › components/components_files/wc_cat_17.jpg]

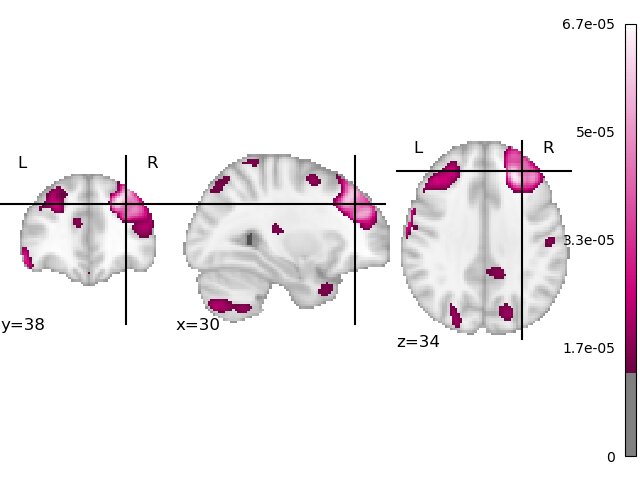

Supplement: S1 Components — (ZIP) [file pcbi.1008795.s002.zip › components/components_files/components_92_stat_map.jpg]

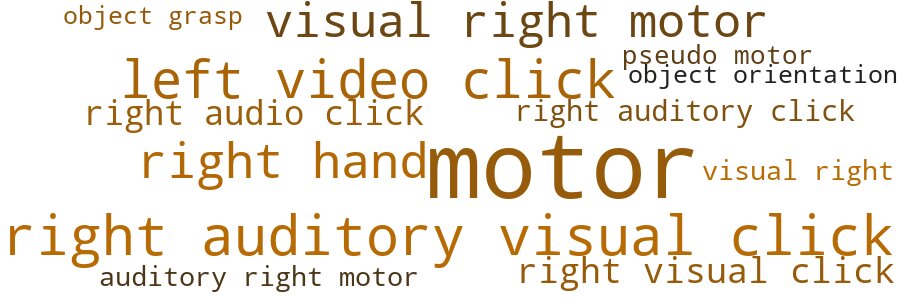

Supplement: S1 Components — (ZIP) [file pcbi.1008795.s002.zip › components/components_files/wc_cat_5.jpg]

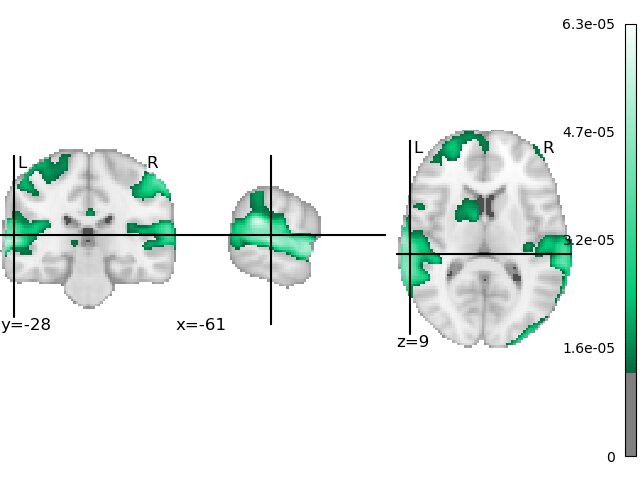

Supplement: S1 Components — (ZIP) [file pcbi.1008795.s002.zip › components/components_files/components_38_stat_map.jpg]

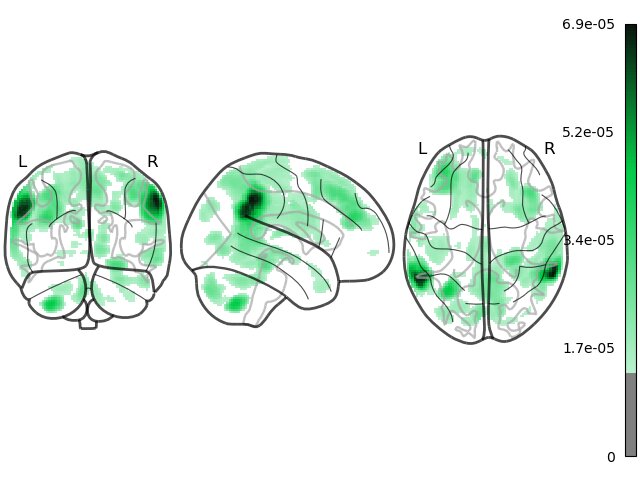

Supplement: S1 Components — (ZIP) [file pcbi.1008795.s002.zip › components/components_files/components_70_glass_brain.jpg]

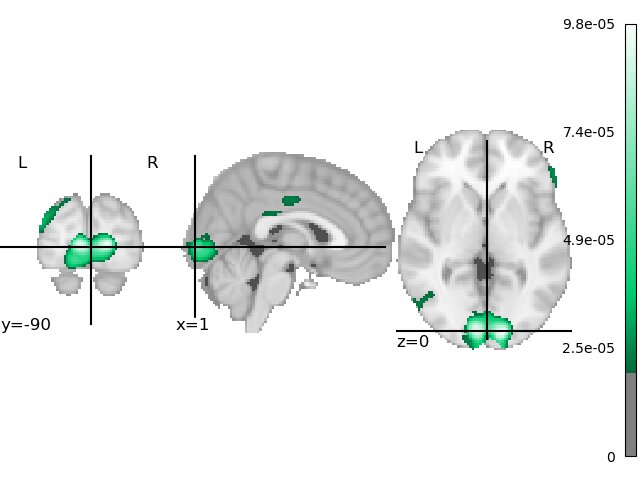

Supplement: S1 Components — (ZIP) [file pcbi.1008795.s002.zip › components/components_files/components_95_stat_map.jpg]

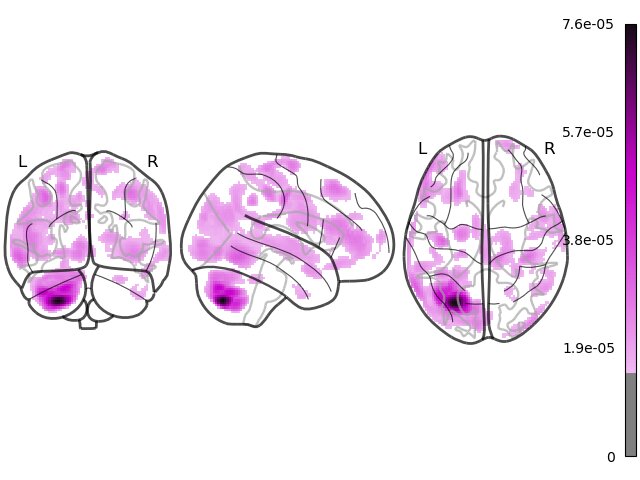

Supplement: S1 Components — (ZIP) [file pcbi.1008795.s002.zip › components/components_files/components_108_glass_brain.jpg]

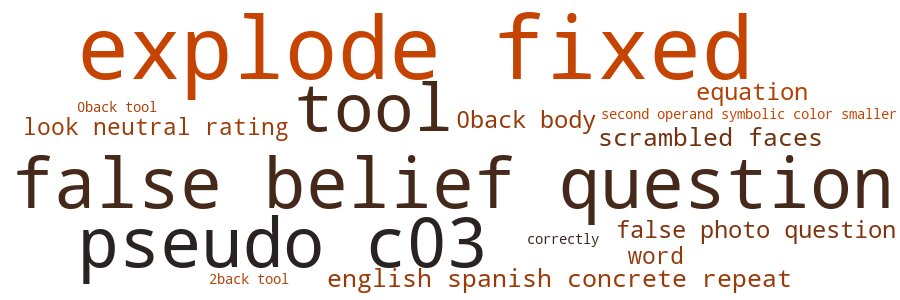

Supplement: S1 Components — (ZIP) [file pcbi.1008795.s002.zip › components/components_files/wc_cat_112.jpg]

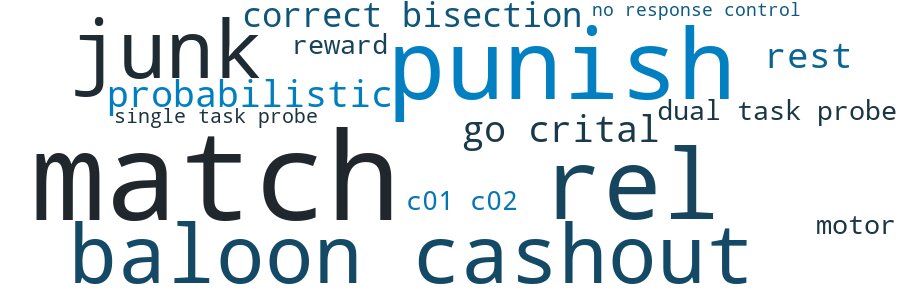

Supplement: S1 Components — (ZIP) [file pcbi.1008795.s002.zip › components/components_files/wc_cat_106.jpg]

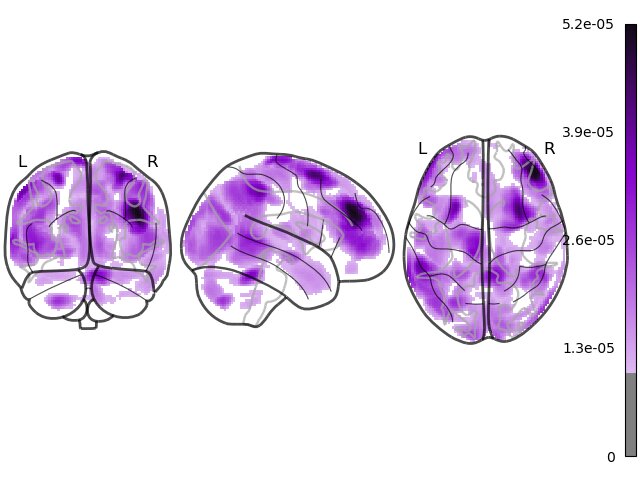

Supplement: S1 Components — (ZIP) [file pcbi.1008795.s002.zip › components/components_files/components_91_glass_brain.jpg]

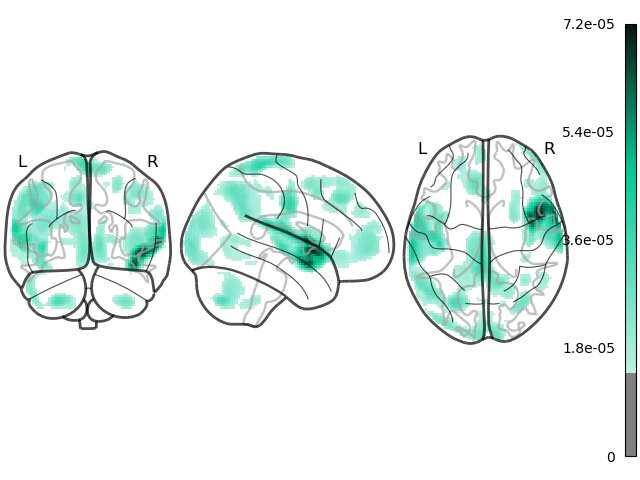

Supplement: S1 Components — (ZIP) [file pcbi.1008795.s002.zip › components/components_files/components_37_glass_brain.jpg]

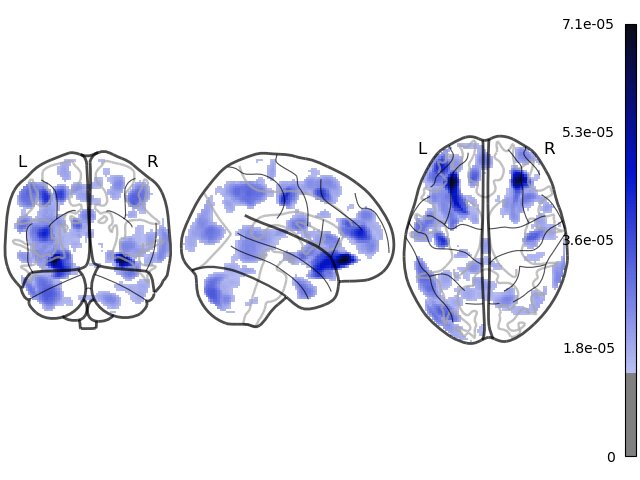

Supplement: S1 Components — (ZIP) [file pcbi.1008795.s002.zip › components/components_files/components_7_glass_brain.jpg]

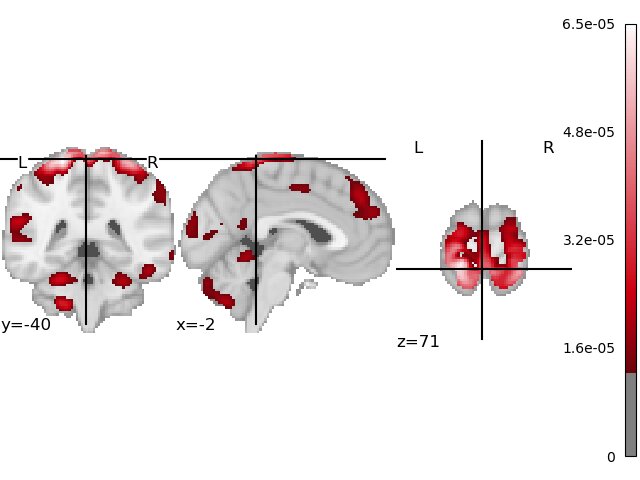

Supplement: S1 Components — (ZIP) [file pcbi.1008795.s002.zip › components/components_files/components_80_stat_map.jpg]

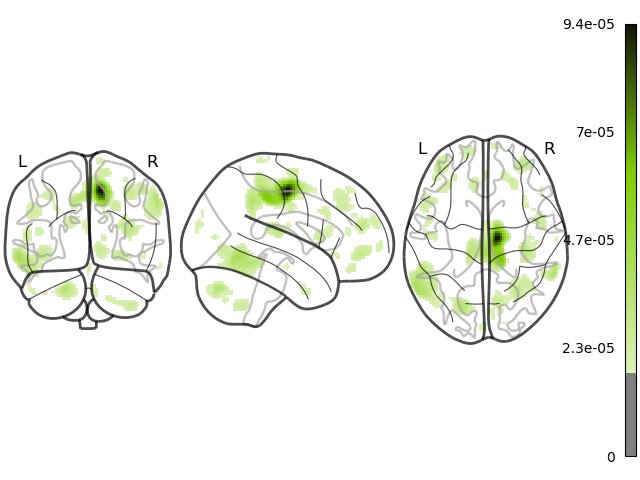

Supplement: S1 Components — (ZIP) [file pcbi.1008795.s002.zip › components/components_files/components_88_glass_brain.jpg]

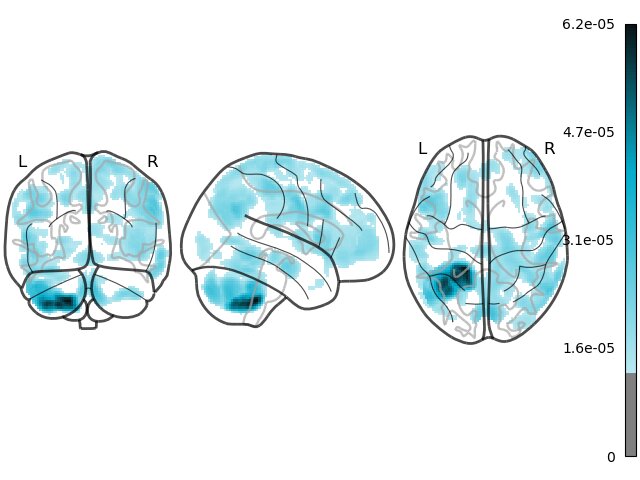

Supplement: S1 Components — (ZIP) [file pcbi.1008795.s002.zip › components/components_files/components_13_glass_brain.jpg]

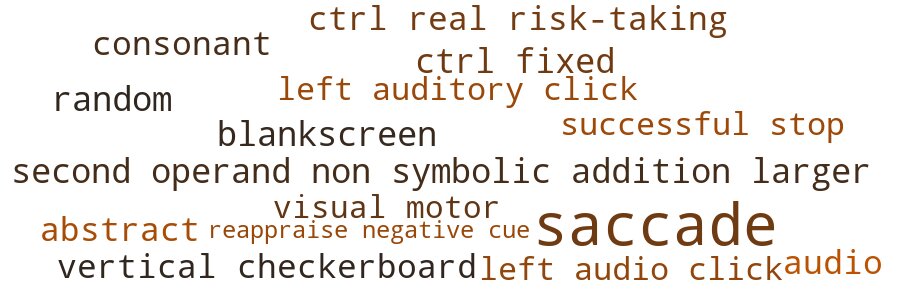

Supplement: S1 Components — (ZIP) [file pcbi.1008795.s002.zip › components/components_files/wc_cat_107.jpg]

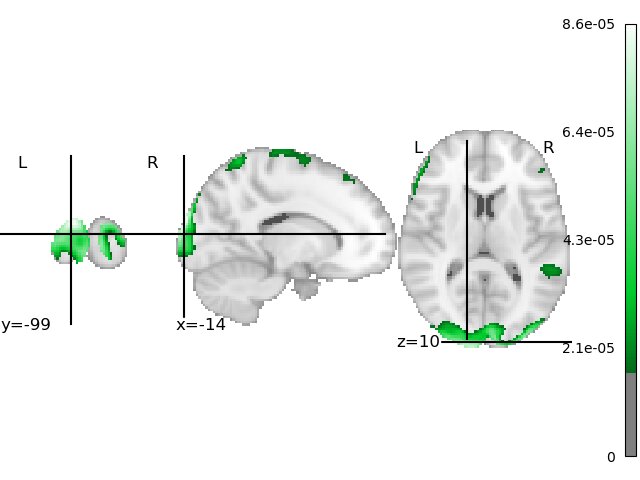

Supplement: S1 Components — (ZIP) [file pcbi.1008795.s002.zip › components/components_files/components_87_stat_map.jpg]

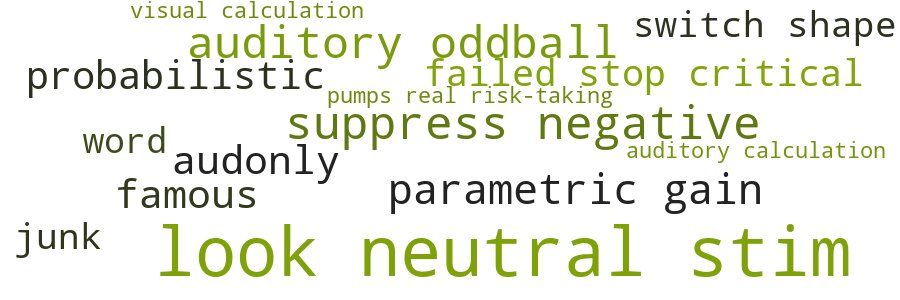

Supplement: S1 Components — (ZIP) [file pcbi.1008795.s002.zip › components/components_files/wc_cat_113.jpg]

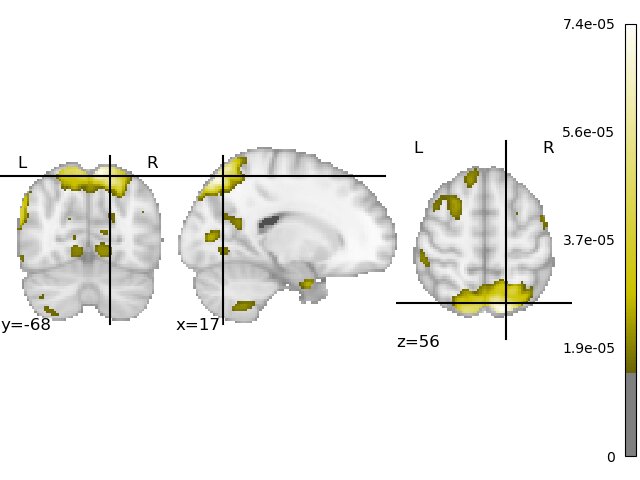

Supplement: S1 Components — (ZIP) [file pcbi.1008795.s002.zip › components/components_files/components_78_stat_map.jpg]

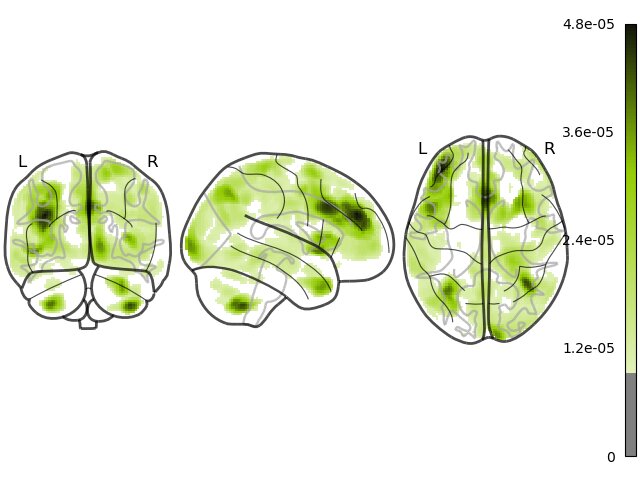

Supplement: S1 Components — (ZIP) [file pcbi.1008795.s002.zip › components/components_files/components_111_glass_brain.jpg]

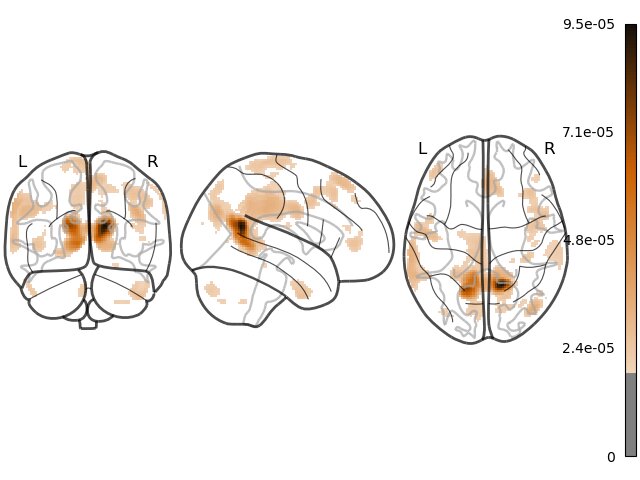

Supplement: S1 Components — (ZIP) [file pcbi.1008795.s002.zip › components/components_files/components_54_glass_brain.jpg]

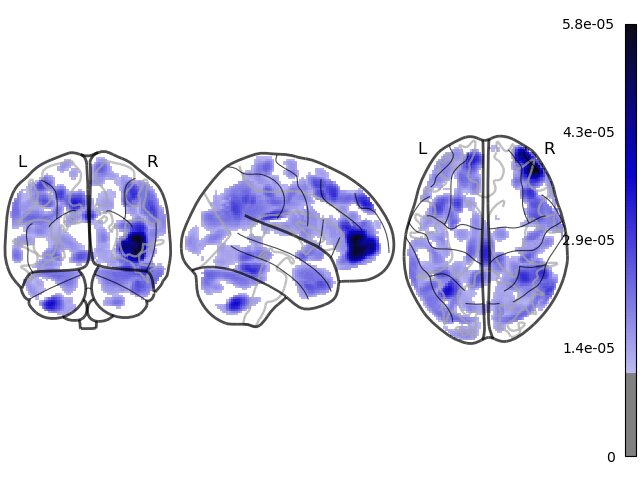

Supplement: S1 Components — (ZIP) [file pcbi.1008795.s002.zip › components/components_files/components_69_glass_brain.jpg]

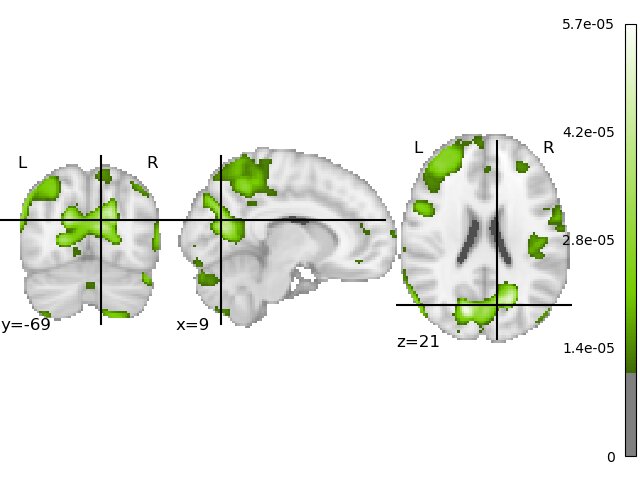

Supplement: S1 Components — (ZIP) [file pcbi.1008795.s002.zip › components/components_files/components_120_stat_map.jpg]

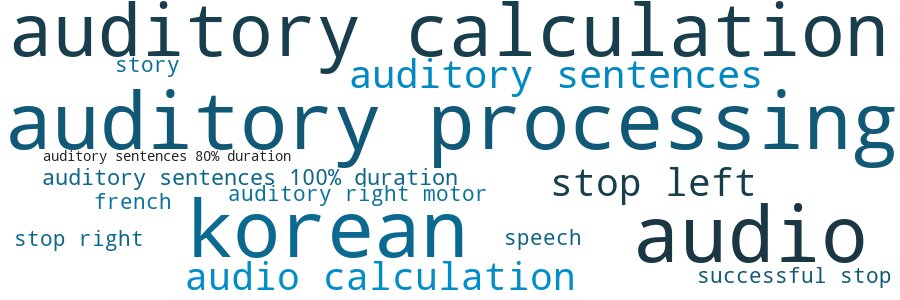

Supplement: S1 Components — (ZIP) [file pcbi.1008795.s002.zip › components/components_files/wc_cat_4.jpg]

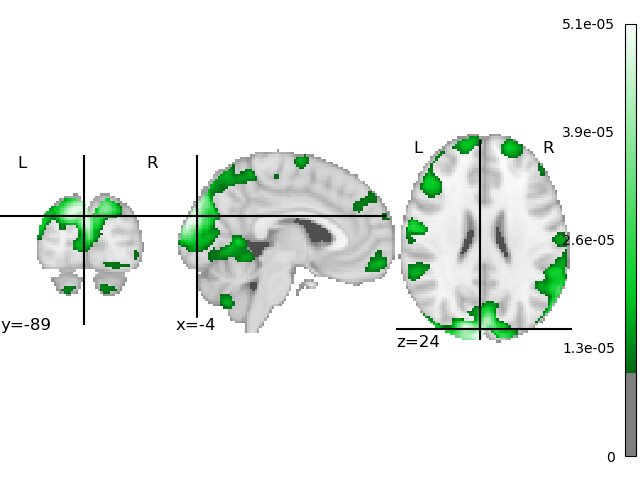

Supplement: S1 Components — (ZIP) [file pcbi.1008795.s002.zip › components/components_files/components_127_stat_map.jpg]

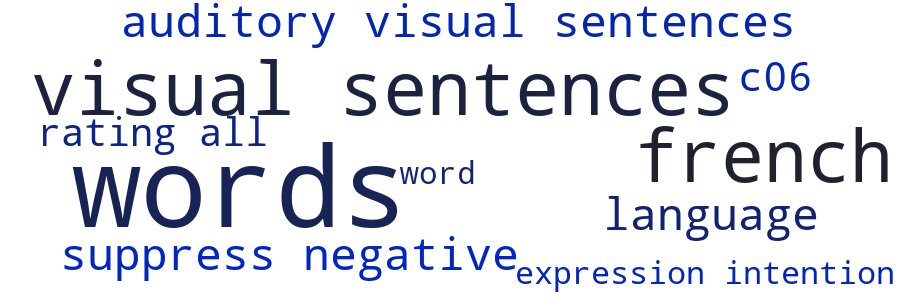

Supplement: S1 Components — (ZIP) [file pcbi.1008795.s002.zip › components/components_files/wc_cat_16.jpg]

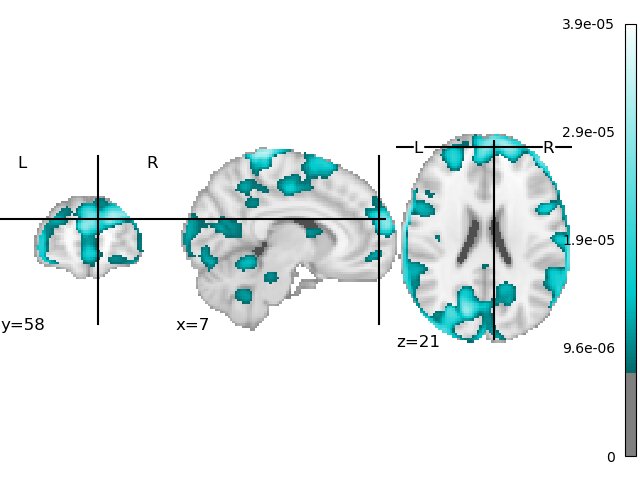

Supplement: S1 Components — (ZIP) [file pcbi.1008795.s002.zip › components/components_files/components_114_stat_map.jpg]

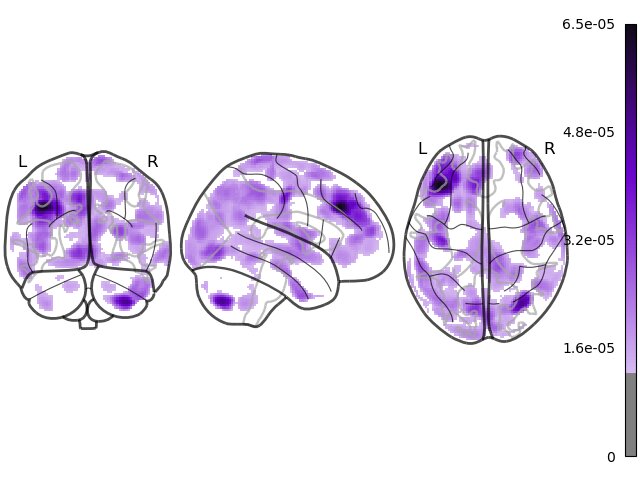

Supplement: S1 Components — (ZIP) [file pcbi.1008795.s002.zip › components/components_files/components_94_glass_brain.jpg]

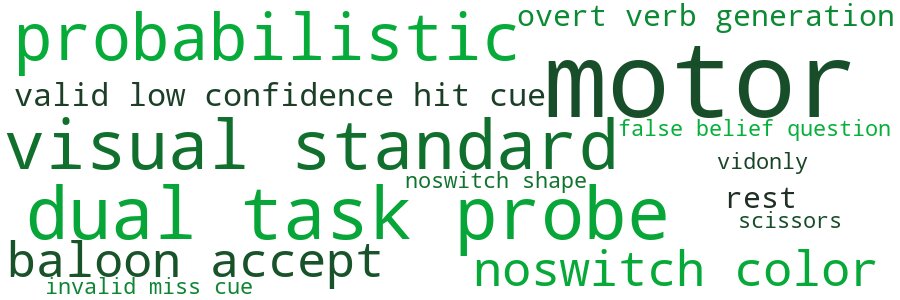

Supplement: S1 Components — (ZIP) [file pcbi.1008795.s002.zip › components/components_files/wc_cat_33.jpg]

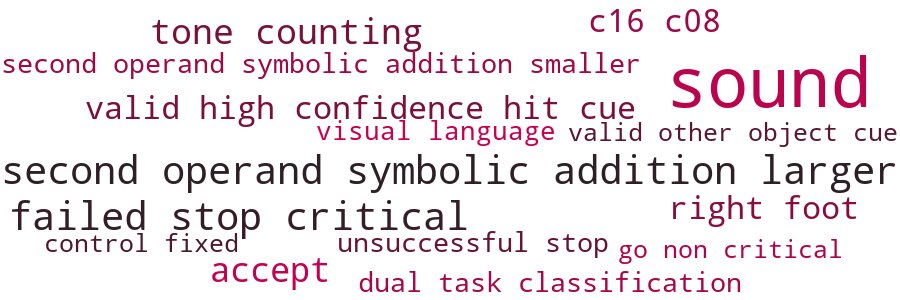

Supplement: S1 Components — (ZIP) [file pcbi.1008795.s002.zip › components/components_files/wc_cat_27.jpg]

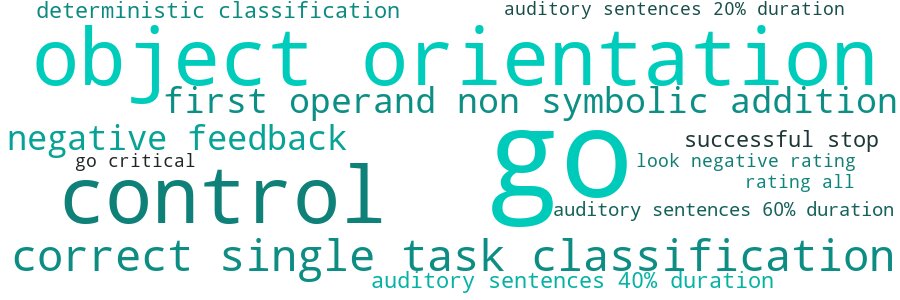

Supplement: S1 Components — (ZIP) [file pcbi.1008795.s002.zip › components/components_files/wc_cat_9.jpg]

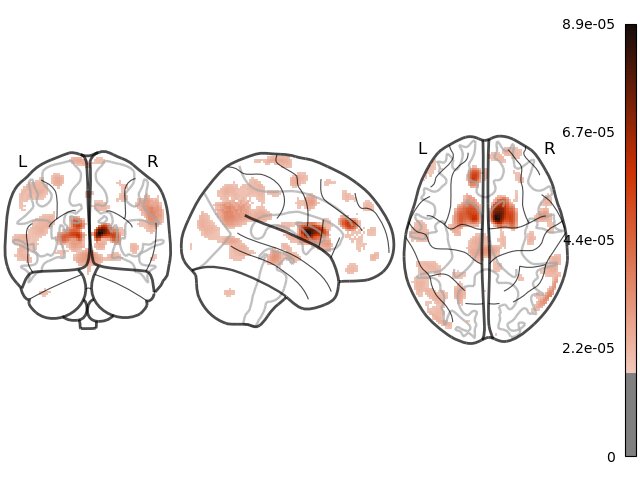

Supplement: S1 Components — (ZIP) [file pcbi.1008795.s002.zip › components/components_files/components_2_glass_brain.jpg]

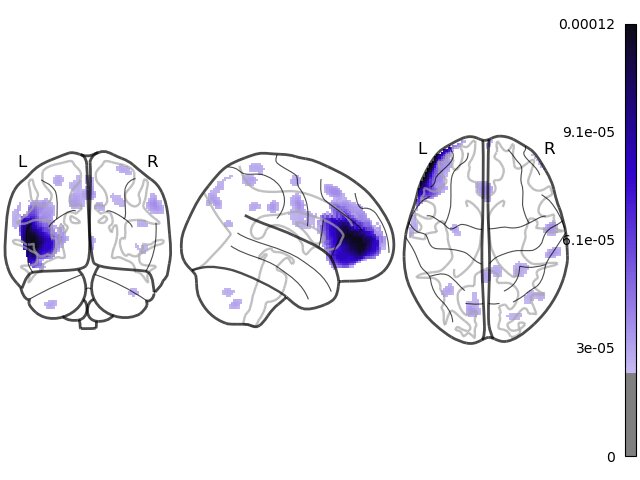

Supplement: S1 Components — (ZIP) [file pcbi.1008795.s002.zip › components/components_files/components_32_glass_brain.jpg]
