# Supplementary figures and images for: Extracting representations of cognition across neuroimaging studies improves brain decoding (part 1 of 2)
Source: PLoS Comput Biol. 2021 May 3;17(5):e1008795. doi: 10.1371/journal.pcbi.1008795 (PMC8118532; doi:10.1371/journal.pcbi.1008795)

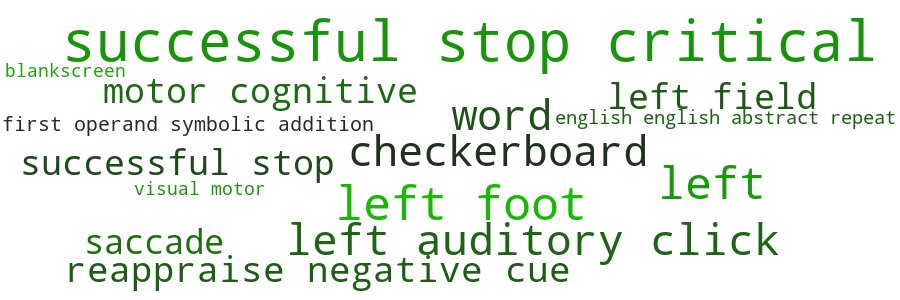

Supplement: S1 Components — (ZIP) [file pcbi.1008795.s002.zip › components/components_files/wc_cat_59.jpg]

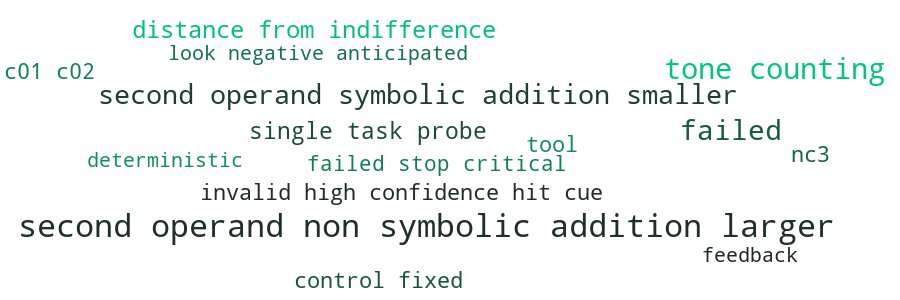

Supplement: S1 Components — (ZIP) [file pcbi.1008795.s002.zip › components/components_files/wc_cat_71.jpg]

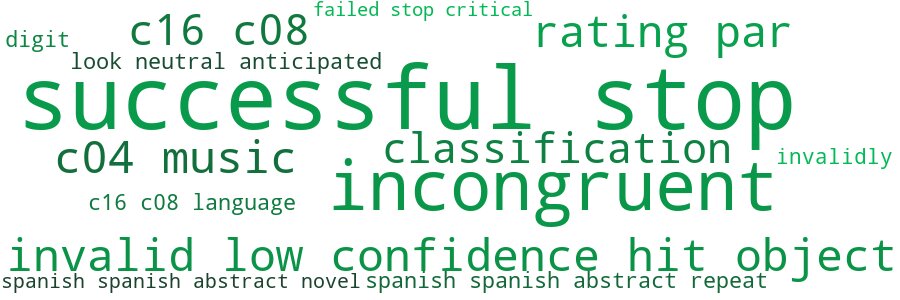

Supplement: S1 Components — (ZIP) [file pcbi.1008795.s002.zip › components/components_files/wc_cat_65.jpg]

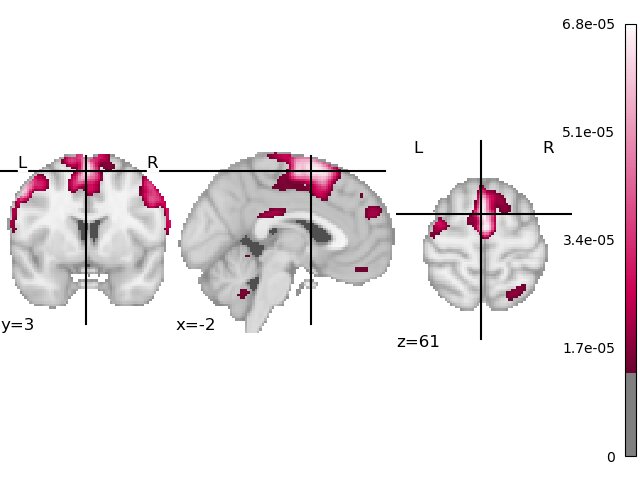

Supplement: S1 Components — (ZIP) [file pcbi.1008795.s002.zip › components/components_files/components_27_stat_map.jpg]

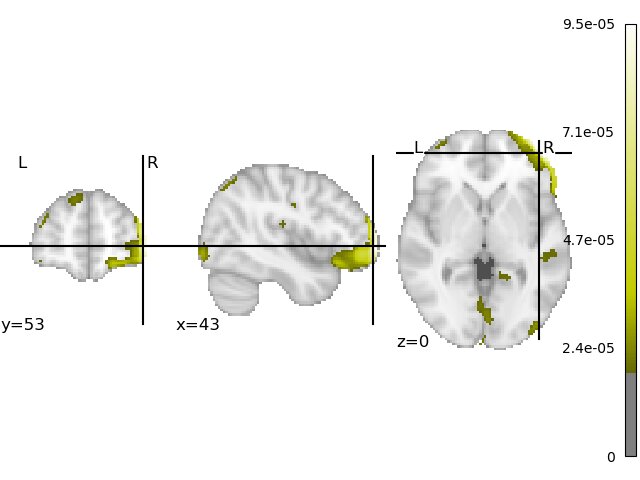

Supplement: S1 Components — (ZIP) [file pcbi.1008795.s002.zip › components/components_files/components_72_stat_map.jpg]

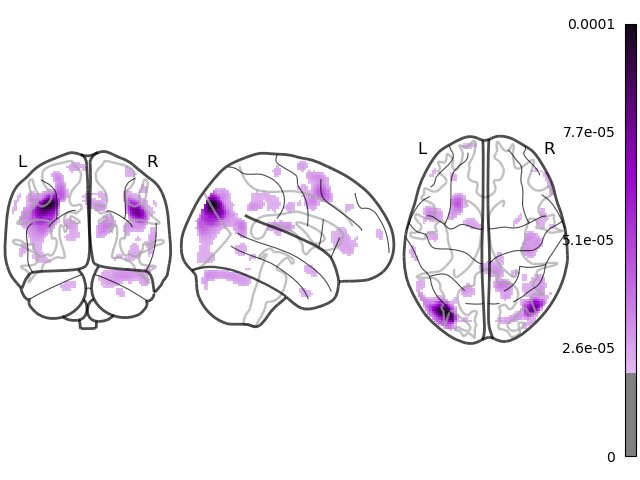

Supplement: S1 Components — (ZIP) [file pcbi.1008795.s002.zip › components/components_files/components_67_glass_brain.jpg]

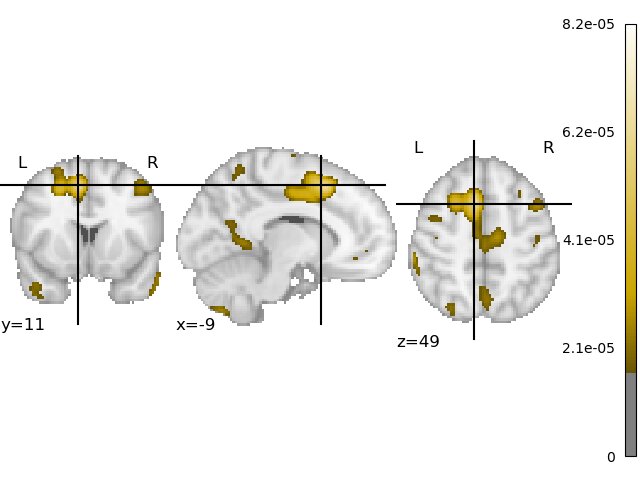

Supplement: S1 Components — (ZIP) [file pcbi.1008795.s002.zip › components/components_files/components_20_stat_map.jpg]

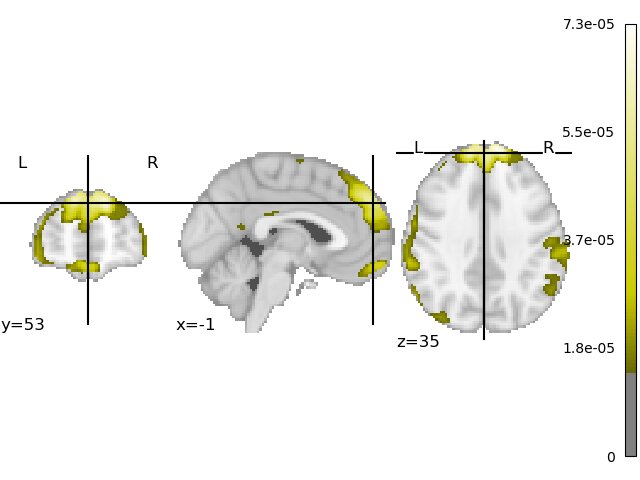

Supplement: S1 Components — (ZIP) [file pcbi.1008795.s002.zip › components/components_files/components_75_stat_map.jpg]

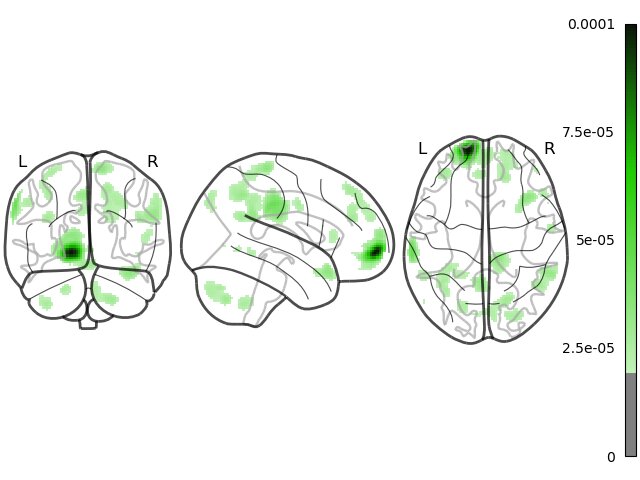

Supplement: S1 Components — (ZIP) [file pcbi.1008795.s002.zip › components/components_files/components_122_glass_brain.jpg]

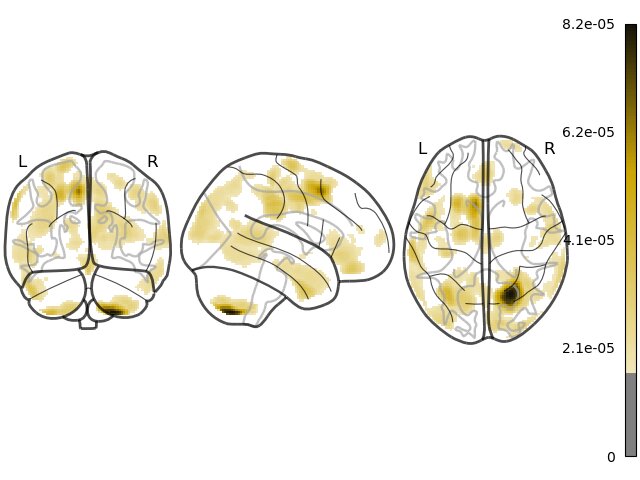

Supplement: S1 Components — (ZIP) [file pcbi.1008795.s002.zip › components/components_files/components_20_glass_brain.jpg]

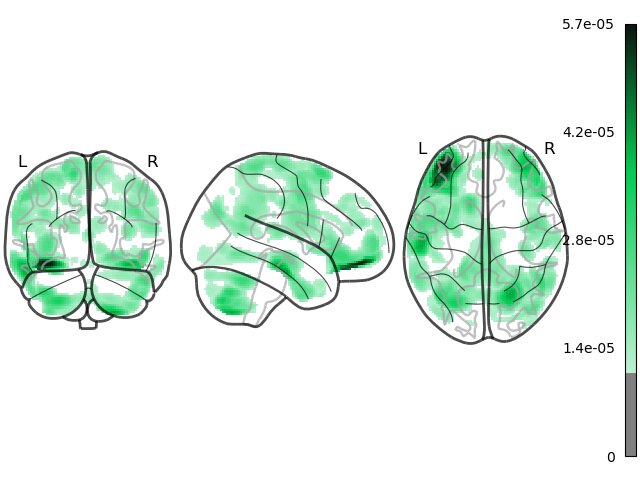

Supplement: S1 Components — (ZIP) [file pcbi.1008795.s002.zip › components/components_files/components_86_glass_brain.jpg]

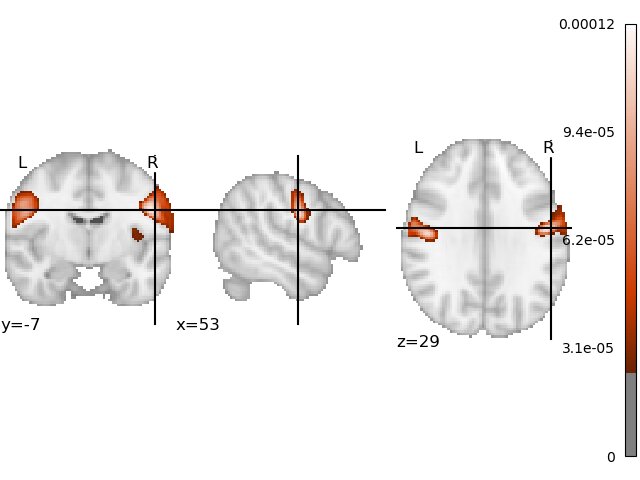

Supplement: S1 Components — (ZIP) [file pcbi.1008795.s002.zip › components/components_files/components_35_stat_map.jpg]

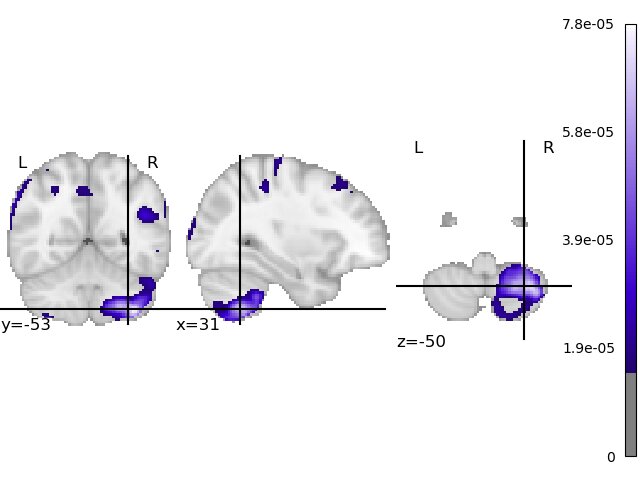

Supplement: S1 Components — (ZIP) [file pcbi.1008795.s002.zip › components/components_files/components_60_stat_map.jpg]

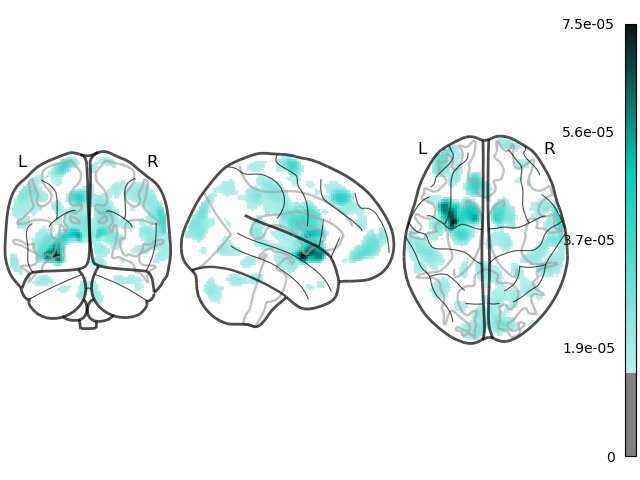

Supplement: S1 Components — (ZIP) [file pcbi.1008795.s002.zip › components/components_files/components_9_glass_brain.jpg]

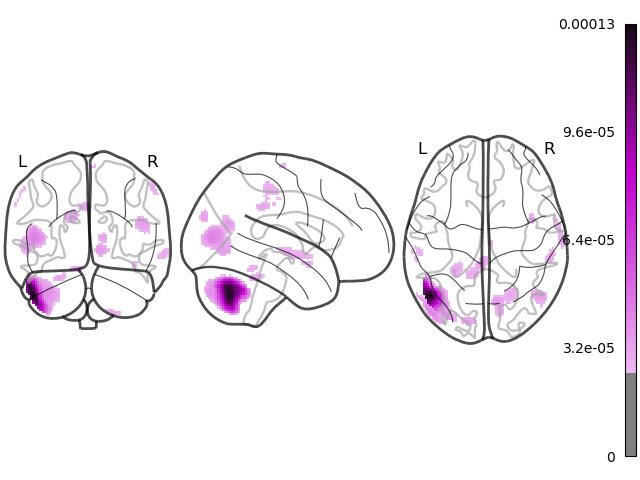

Supplement: S1 Components — (ZIP) [file pcbi.1008795.s002.zip › components/components_files/components_39_glass_brain.jpg]

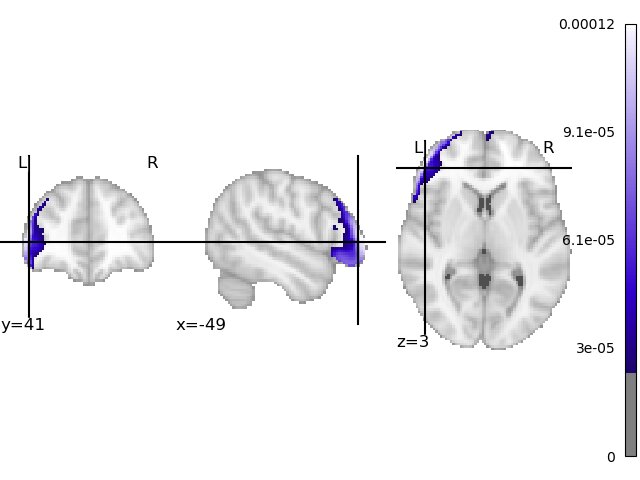

Supplement: S1 Components — (ZIP) [file pcbi.1008795.s002.zip › components/components_files/components_32_stat_map.jpg]

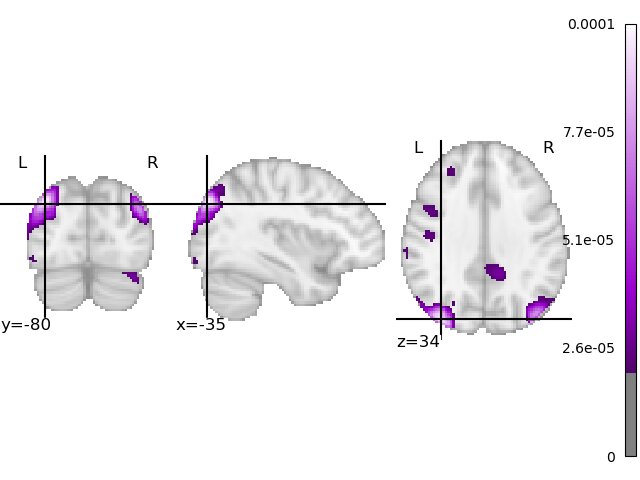

Supplement: S1 Components — (ZIP) [file pcbi.1008795.s002.zip › components/components_files/components_67_stat_map.jpg]

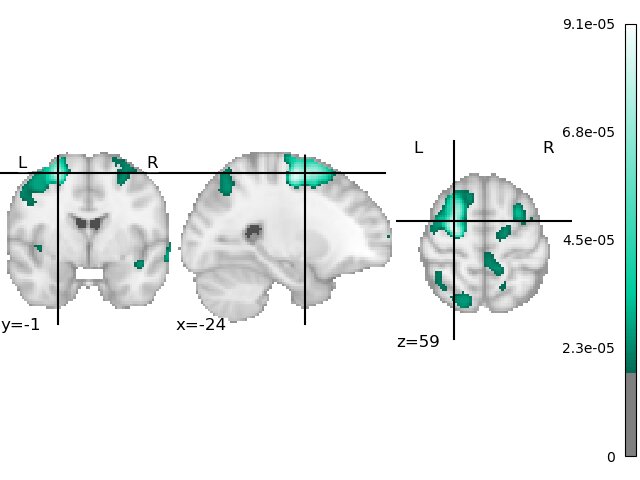

Supplement: S1 Components — (ZIP) [file pcbi.1008795.s002.zip › components/components_files/components_98_stat_map.jpg]

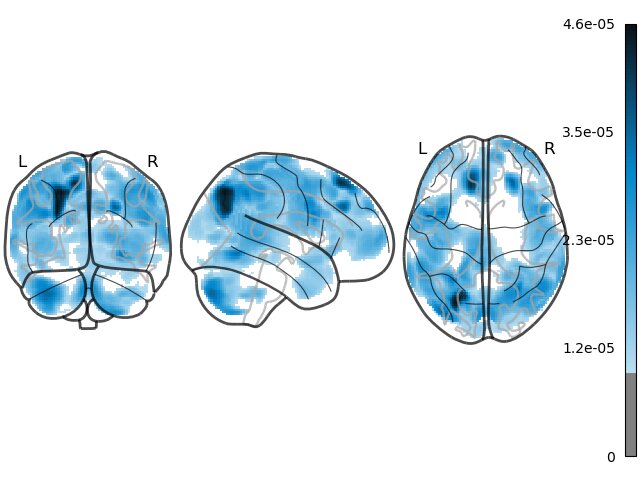

Supplement: S1 Components — (ZIP) [file pcbi.1008795.s002.zip › components/components_files/components_106_glass_brain.jpg]

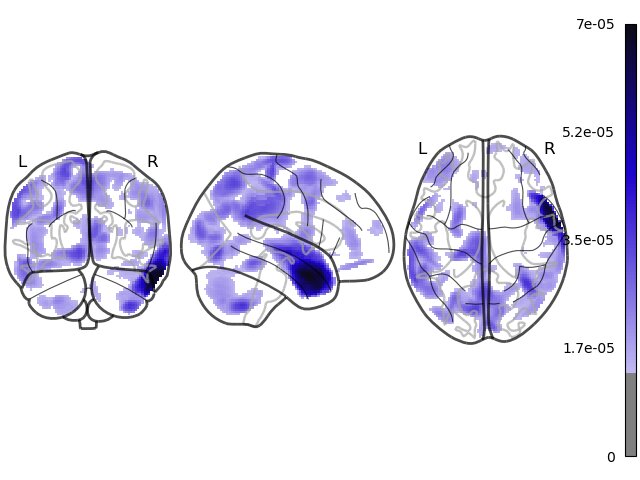

Supplement: S1 Components — (ZIP) [file pcbi.1008795.s002.zip › components/components_files/components_43_glass_brain.jpg]

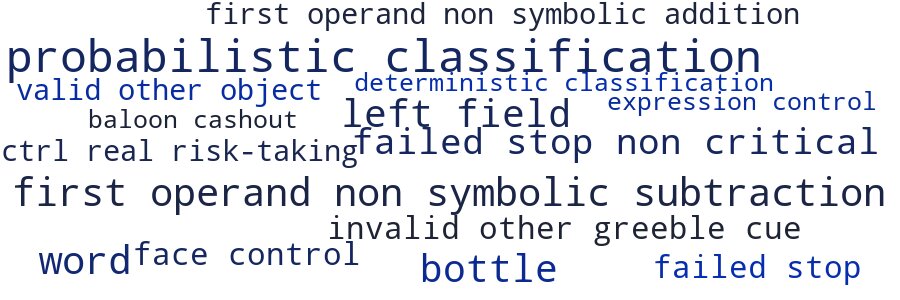

Supplement: S1 Components — (ZIP) [file pcbi.1008795.s002.zip › components/components_files/wc_cat_64.jpg]

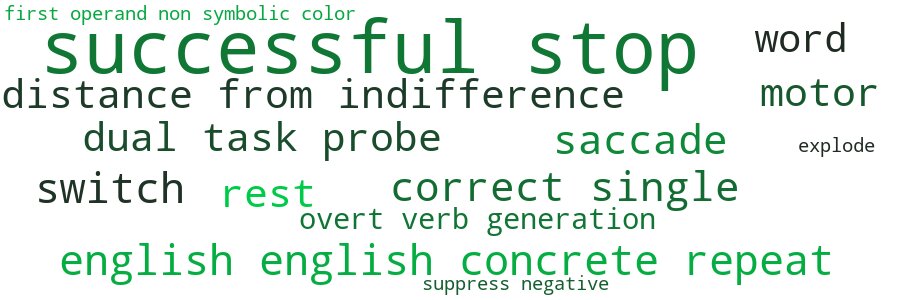

Supplement: S1 Components — (ZIP) [file pcbi.1008795.s002.zip › components/components_files/wc_cat_70.jpg]

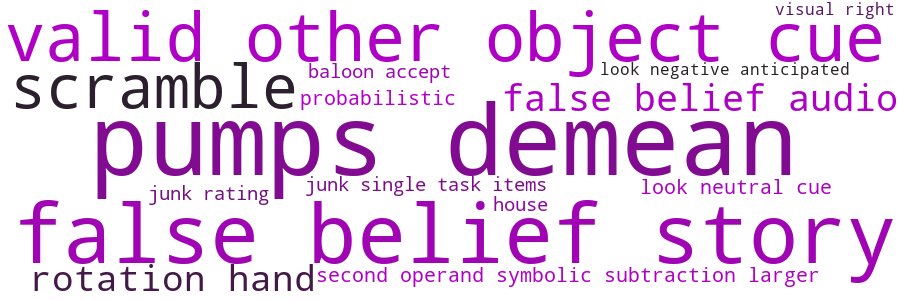

Supplement: S1 Components — (ZIP) [file pcbi.1008795.s002.zip › components/components_files/wc_cat_58.jpg]

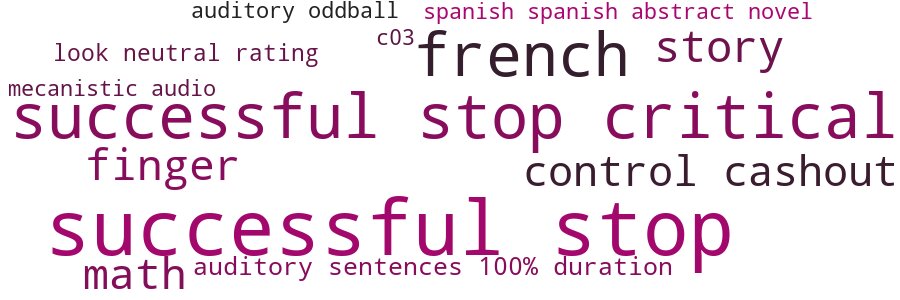

Supplement: S1 Components — (ZIP) [file pcbi.1008795.s002.zip › components/components_files/wc_cat_66.jpg]

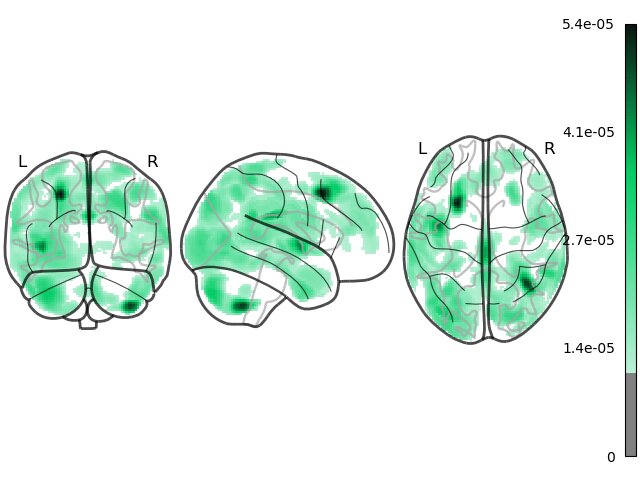

Supplement: S1 Components — (ZIP) [file pcbi.1008795.s002.zip › components/components_files/components_109_glass_brain.jpg]

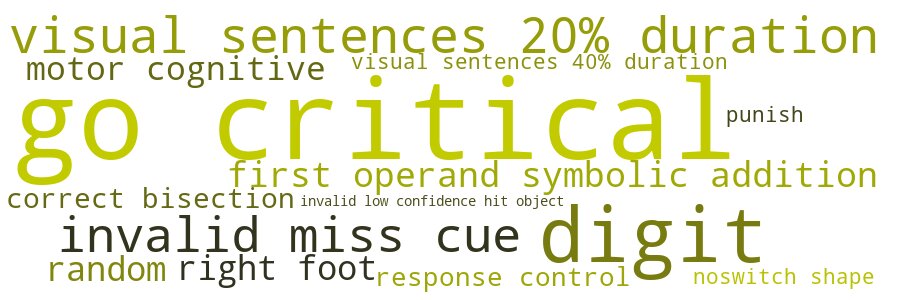

Supplement: S1 Components — (ZIP) [file pcbi.1008795.s002.zip › components/components_files/wc_cat_72.jpg]

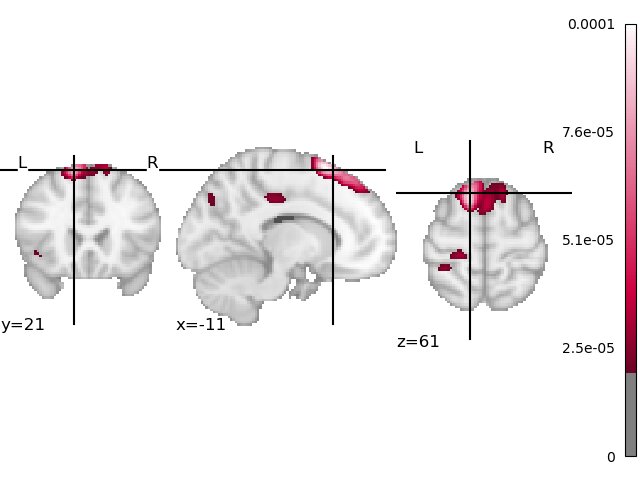

Supplement: S1 Components — (ZIP) [file pcbi.1008795.s002.zip › components/components_files/components_51_stat_map.jpg]

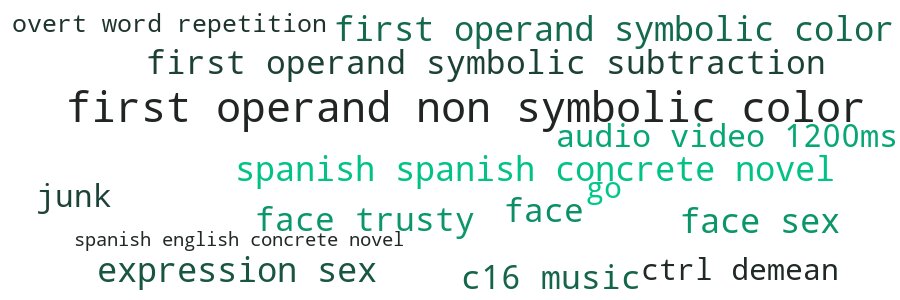

Supplement: S1 Components — (ZIP) [file pcbi.1008795.s002.zip › components/components_files/wc_cat_99.jpg]

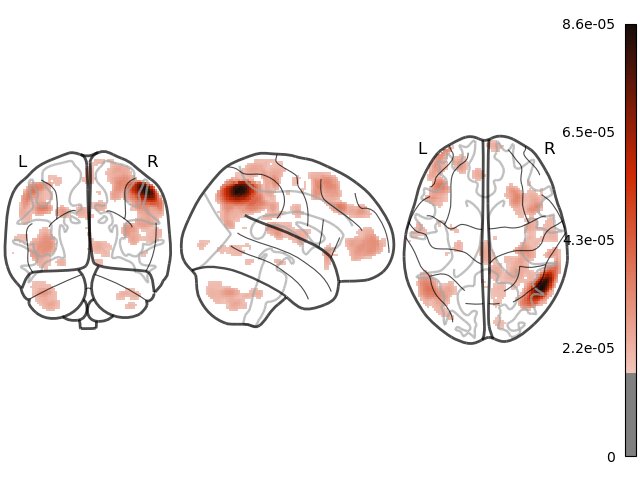

Supplement: S1 Components — (ZIP) [file pcbi.1008795.s002.zip › components/components_files/components_90_glass_brain.jpg]

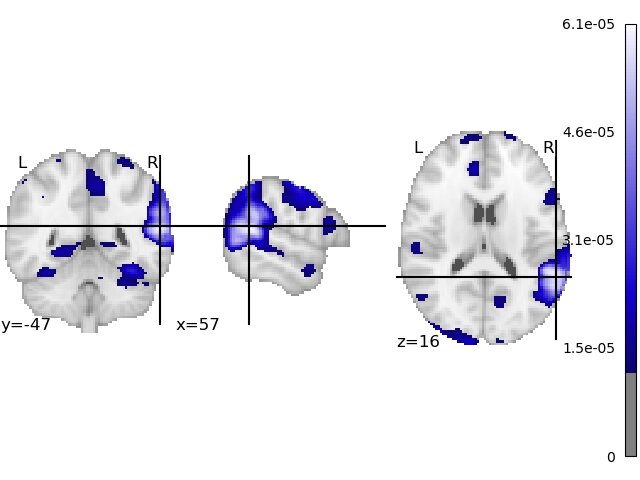

Supplement: S1 Components — (ZIP) [file pcbi.1008795.s002.zip › components/components_files/components_56_stat_map.jpg]

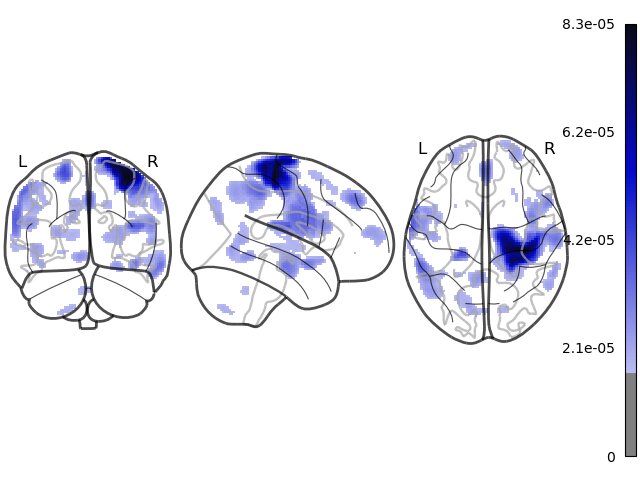

Supplement: S1 Components — (ZIP) [file pcbi.1008795.s002.zip › components/components_files/components_36_glass_brain.jpg]

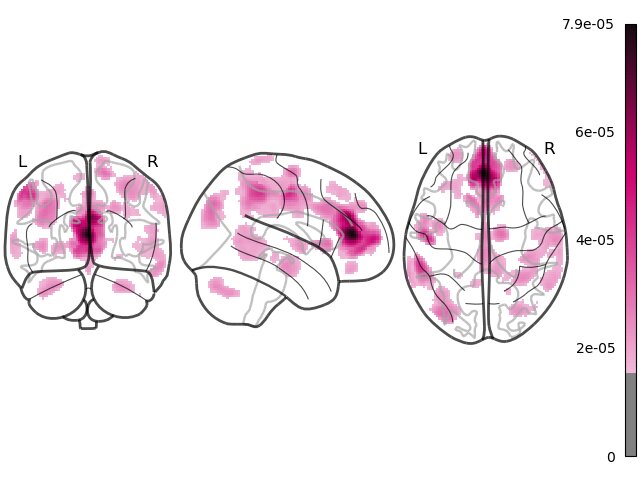

Supplement: S1 Components — (ZIP) [file pcbi.1008795.s002.zip › components/components_files/components_6_glass_brain.jpg]

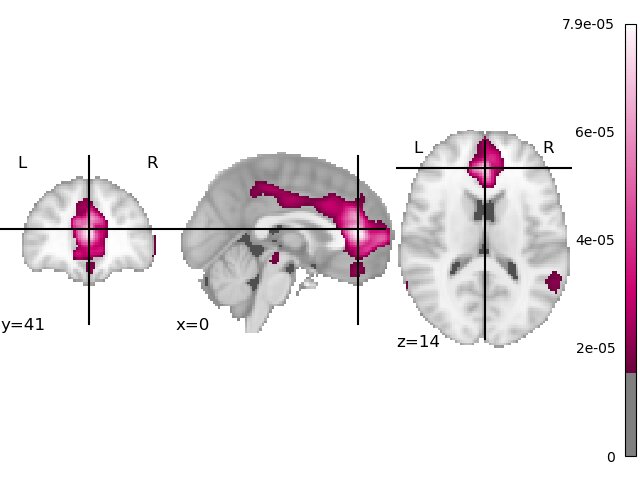

Supplement: S1 Components — (ZIP) [file pcbi.1008795.s002.zip › components/components_files/components_6_stat_map.jpg]

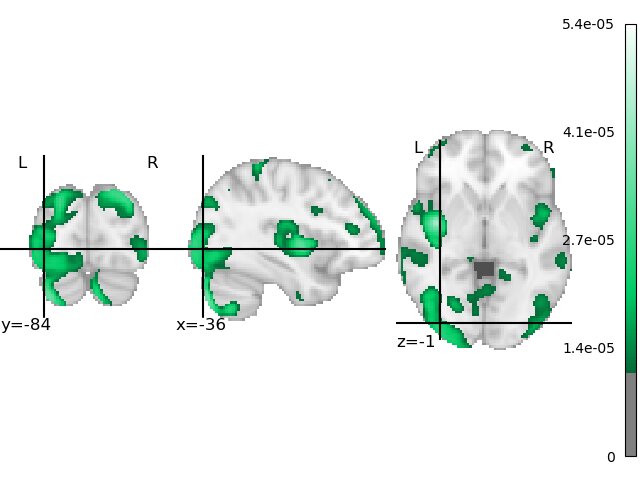

Supplement: S1 Components — (ZIP) [file pcbi.1008795.s002.zip › components/components_files/components_109_stat_map.jpg]

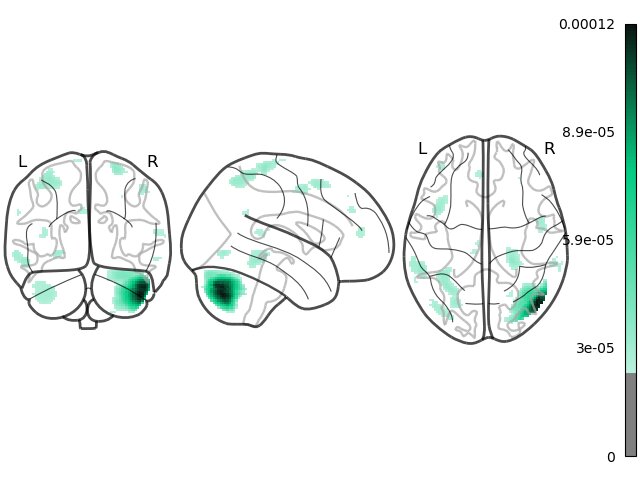

Supplement: S1 Components — (ZIP) [file pcbi.1008795.s002.zip › components/components_files/components_71_glass_brain.jpg]

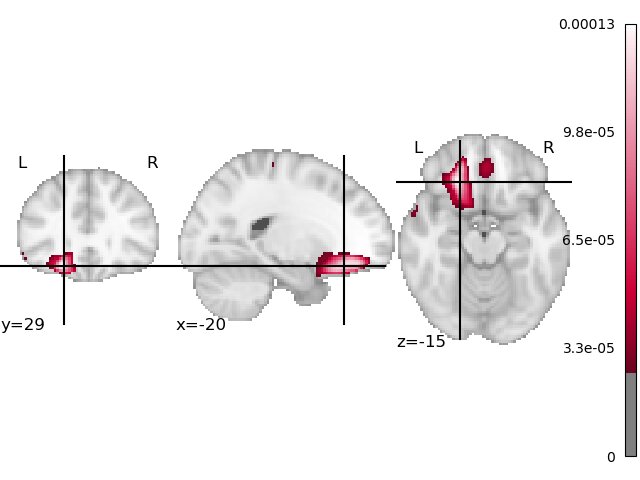

Supplement: S1 Components — (ZIP) [file pcbi.1008795.s002.zip › components/components_files/components_1_stat_map.jpg]

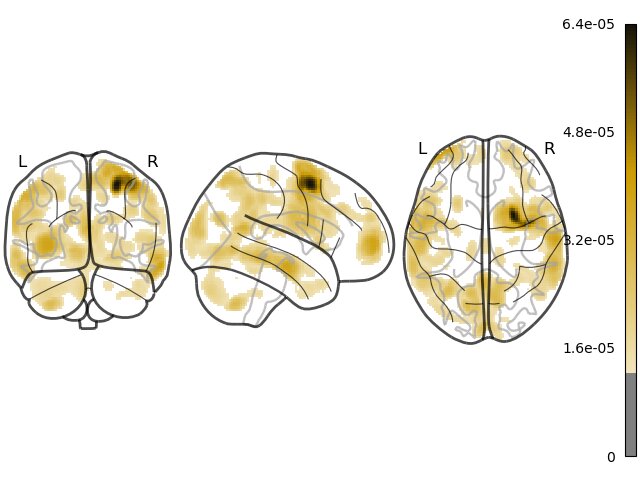

Supplement: S1 Components — (ZIP) [file pcbi.1008795.s002.zip › components/components_files/components_55_glass_brain.jpg]

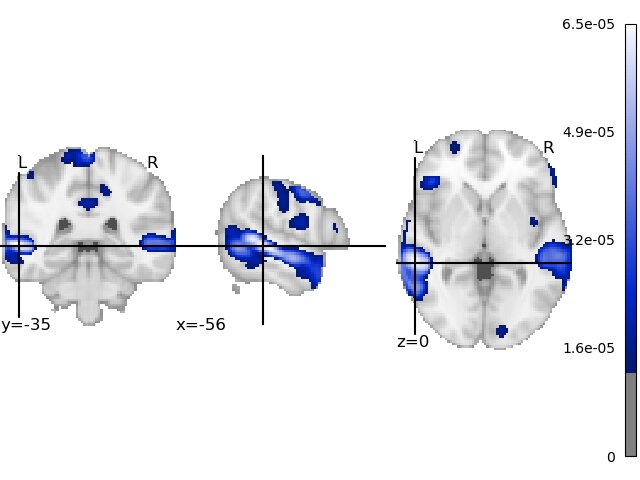

Supplement: S1 Components — (ZIP) [file pcbi.1008795.s002.zip › components/components_files/components_16_stat_map.jpg]

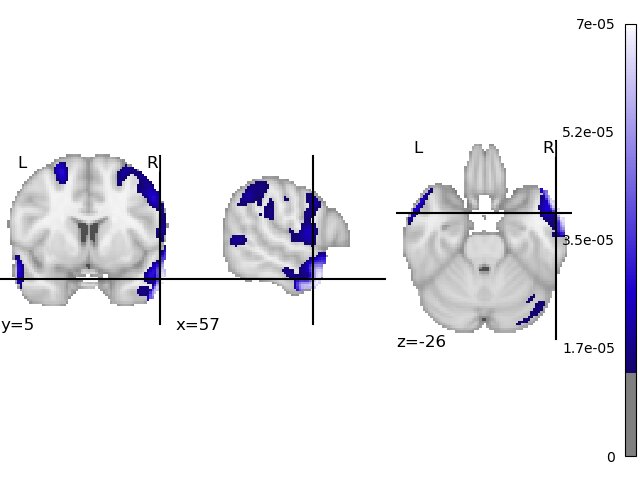

Supplement: S1 Components — (ZIP) [file pcbi.1008795.s002.zip › components/components_files/components_43_stat_map.jpg]

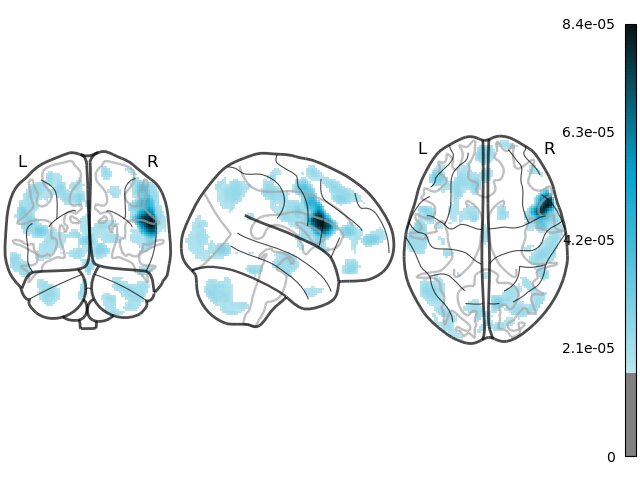

Supplement: S1 Components — (ZIP) [file pcbi.1008795.s002.zip › components/components_files/components_68_glass_brain.jpg]

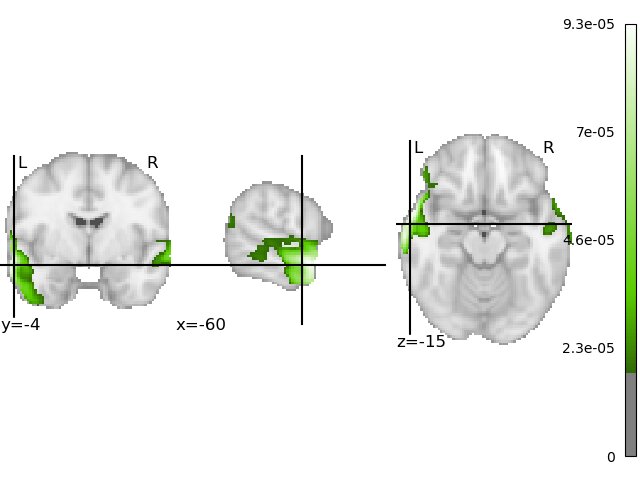

Supplement: S1 Components — (ZIP) [file pcbi.1008795.s002.zip › components/components_files/components_11_stat_map.jpg]

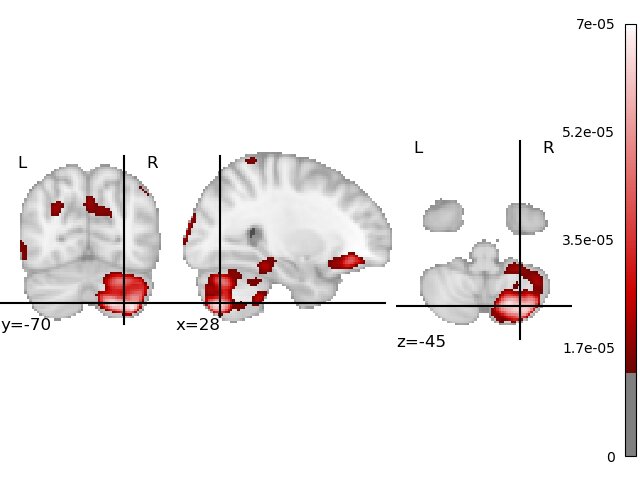

Supplement: S1 Components — (ZIP) [file pcbi.1008795.s002.zip › components/components_files/components_44_stat_map.jpg]

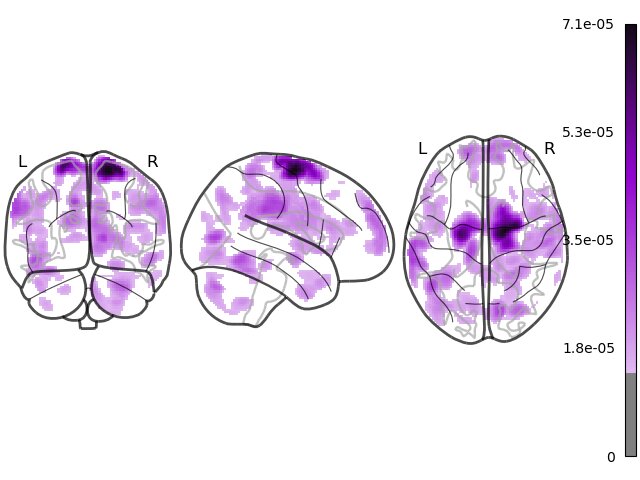

Supplement: S1 Components — (ZIP) [file pcbi.1008795.s002.zip › components/components_files/components_89_glass_brain.jpg]

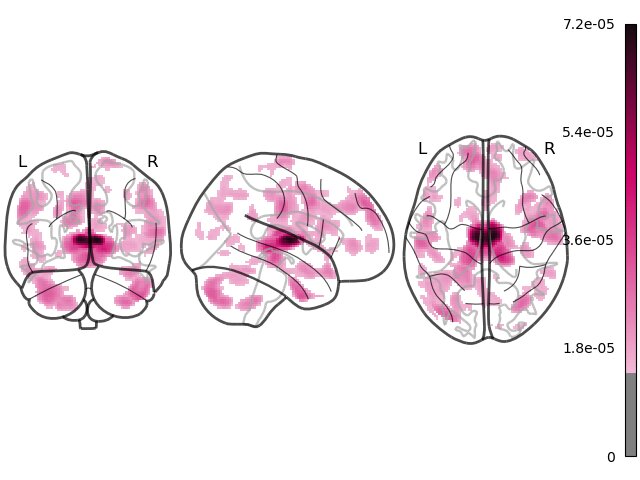

Supplement: S1 Components — (ZIP) [file pcbi.1008795.s002.zip › components/components_files/components_12_glass_brain.jpg]

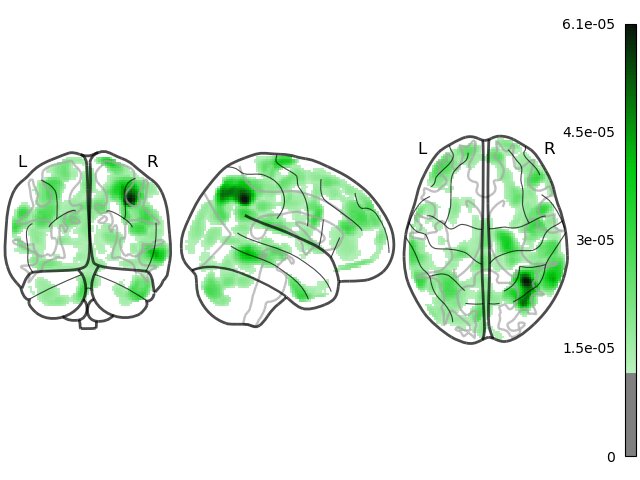

Supplement: S1 Components — (ZIP) [file pcbi.1008795.s002.zip › components/components_files/components_110_glass_brain.jpg]

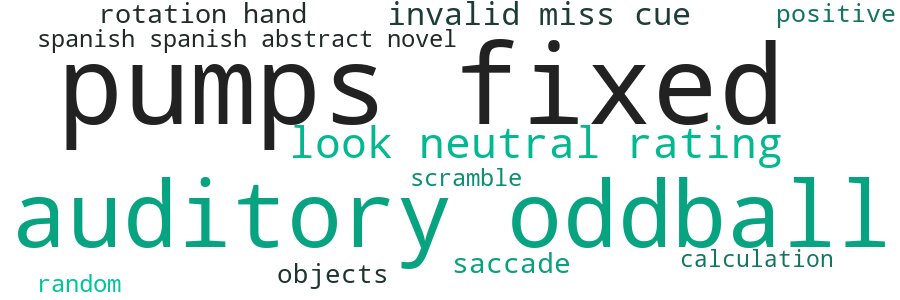

Supplement: S1 Components — (ZIP) [file pcbi.1008795.s002.zip › components/components_files/wc_cat_98.jpg]

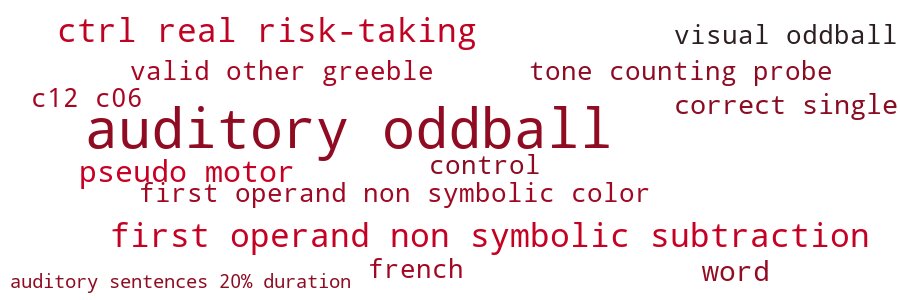

Supplement: S1 Components — (ZIP) [file pcbi.1008795.s002.zip › components/components_files/wc_cat_73.jpg]

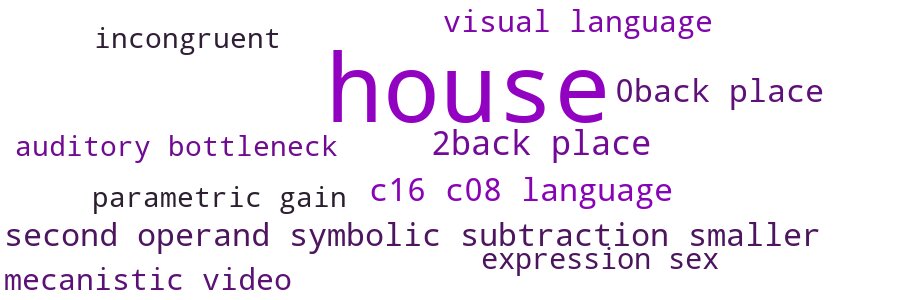

Supplement: S1 Components — (ZIP) [file pcbi.1008795.s002.zip › components/components_files/wc_cat_67.jpg]

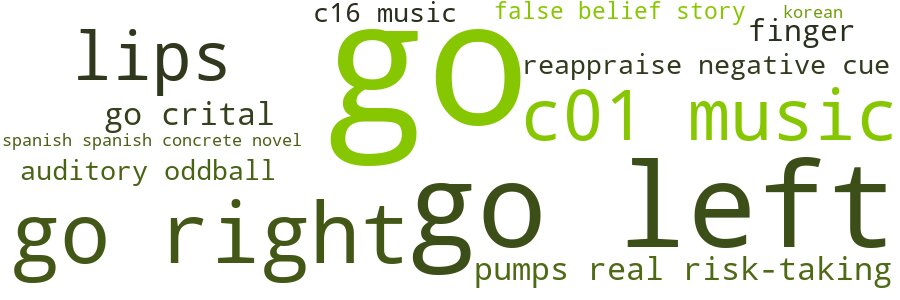

Supplement: S1 Components — (ZIP) [file pcbi.1008795.s002.zip › components/components_files/wc_cat_63.jpg]

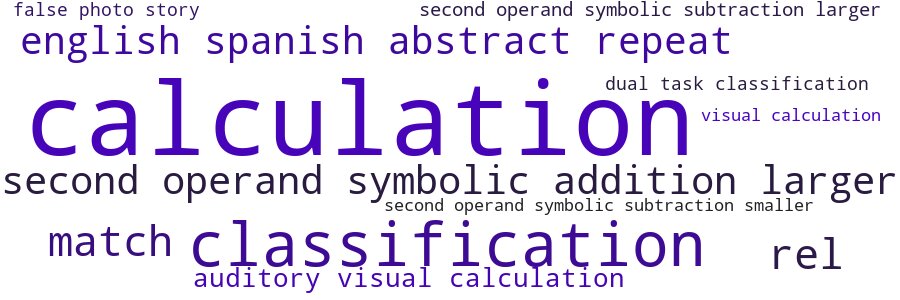

Supplement: S1 Components — (ZIP) [file pcbi.1008795.s002.zip › components/components_files/wc_cat_77.jpg]

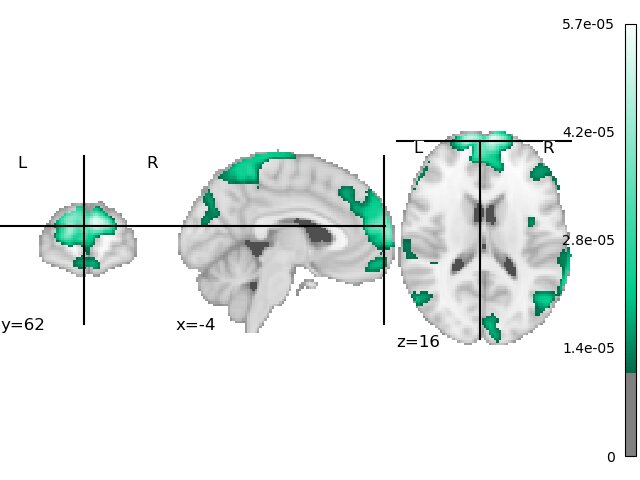

Supplement: S1 Components — (ZIP) [file pcbi.1008795.s002.zip › components/components_files/components_99_stat_map.jpg]

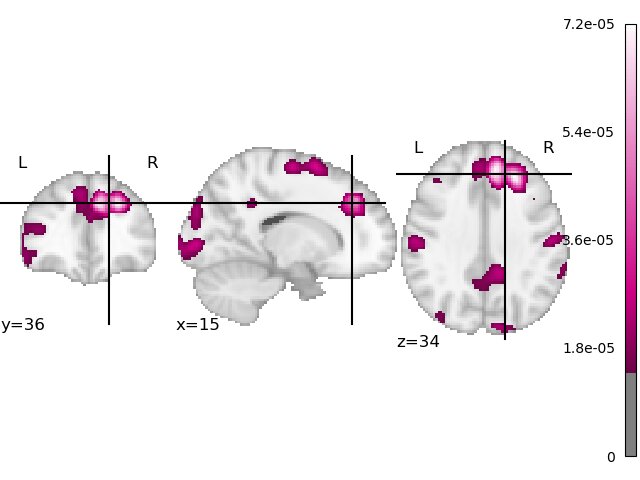

Supplement: S1 Components — (ZIP) [file pcbi.1008795.s002.zip › components/components_files/components_66_stat_map.jpg]

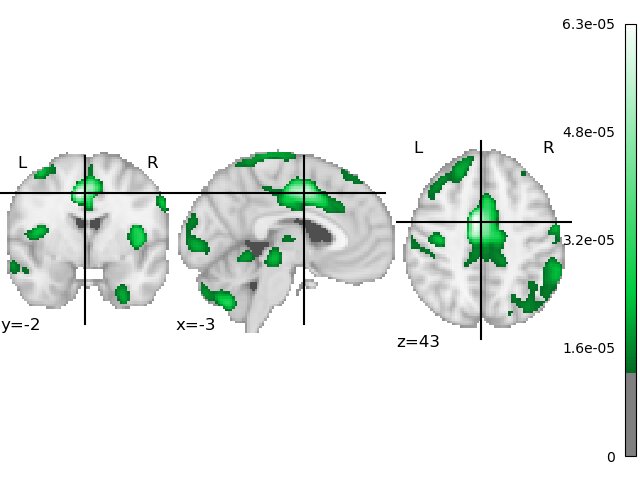

Supplement: S1 Components — (ZIP) [file pcbi.1008795.s002.zip › components/components_files/components_33_stat_map.jpg]

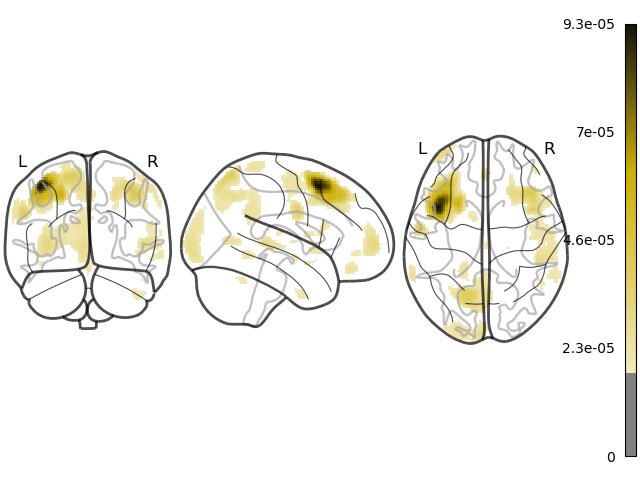

Supplement: S1 Components — (ZIP) [file pcbi.1008795.s002.zip › components/components_files/components_117_glass_brain.jpg]

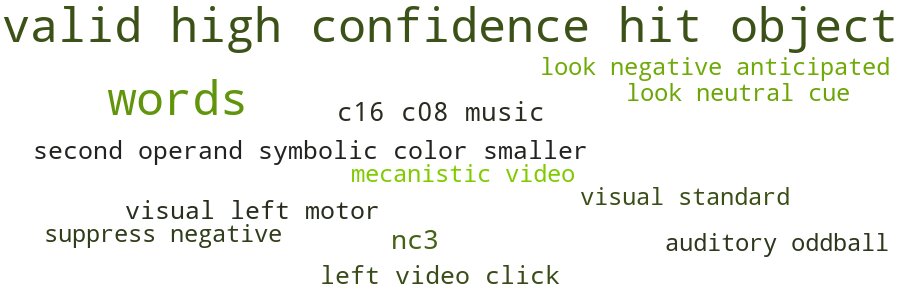

Supplement: S1 Components — (ZIP) [file pcbi.1008795.s002.zip › components/components_files/wc_cat_88.jpg]

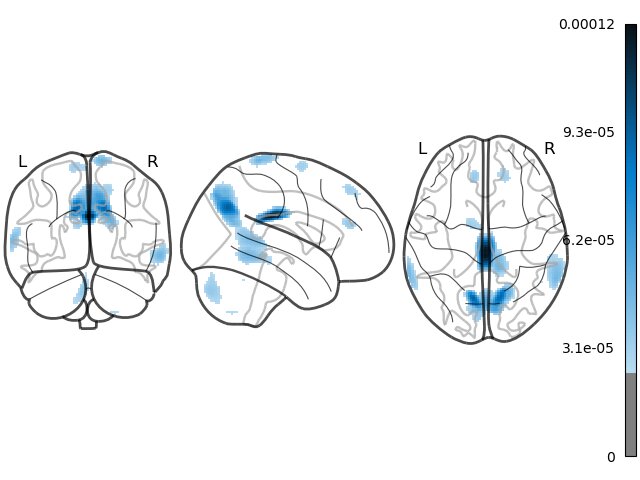

Supplement: S1 Components — (ZIP) [file pcbi.1008795.s002.zip › components/components_files/components_28_glass_brain.jpg]

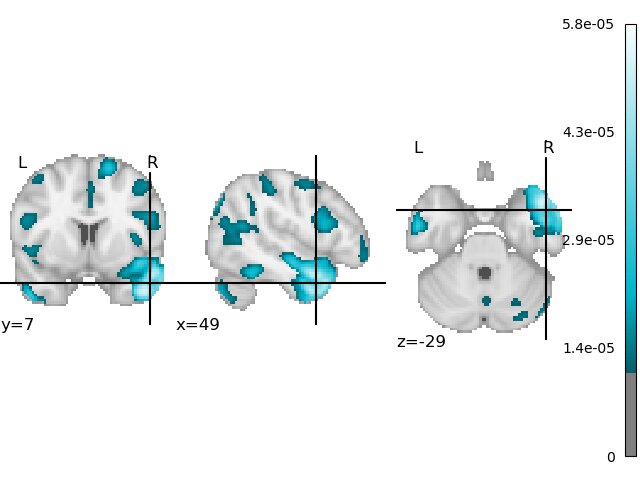

Supplement: S1 Components — (ZIP) [file pcbi.1008795.s002.zip › components/components_files/components_61_stat_map.jpg]

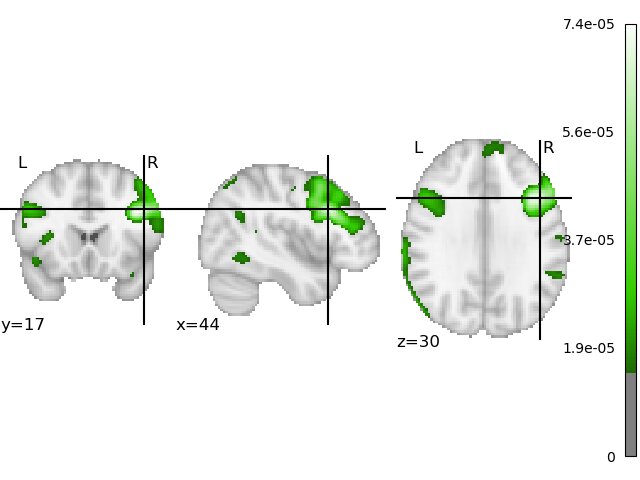

Supplement: S1 Components — (ZIP) [file pcbi.1008795.s002.zip › components/components_files/components_34_stat_map.jpg]

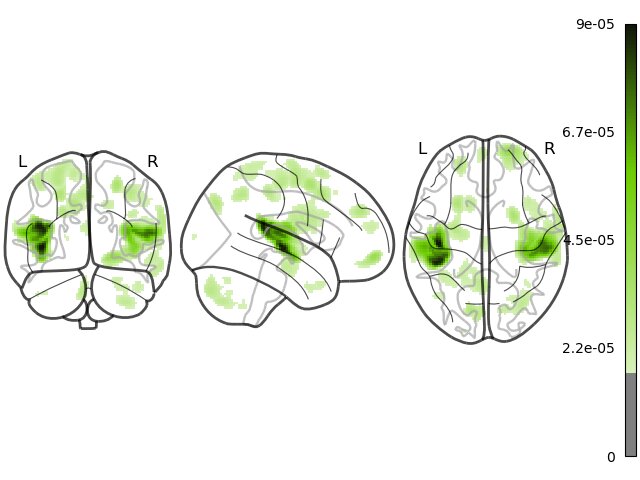

Supplement: S1 Components — (ZIP) [file pcbi.1008795.s002.zip › components/components_files/components_15_glass_brain.jpg]

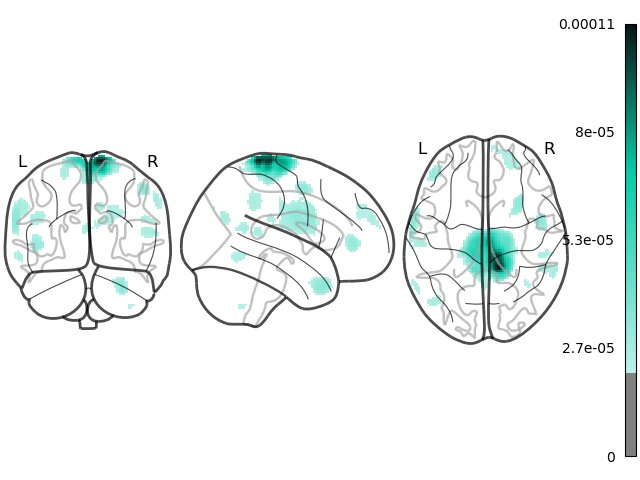

Supplement: S1 Components — (ZIP) [file pcbi.1008795.s002.zip › components/components_files/components_52_glass_brain.jpg]

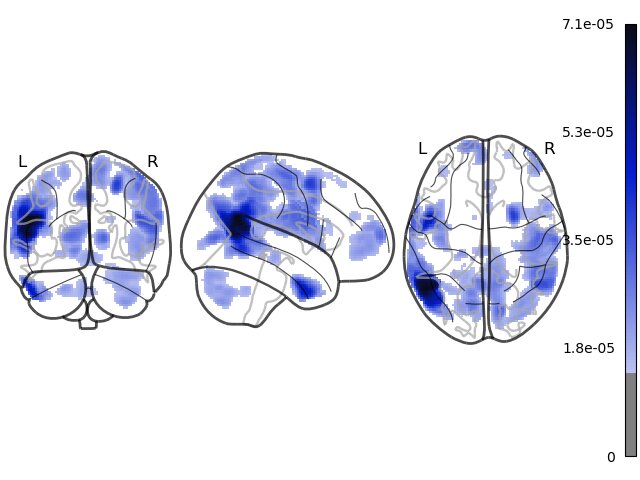

Supplement: S1 Components — (ZIP) [file pcbi.1008795.s002.zip › components/components_files/components_76_glass_brain.jpg]

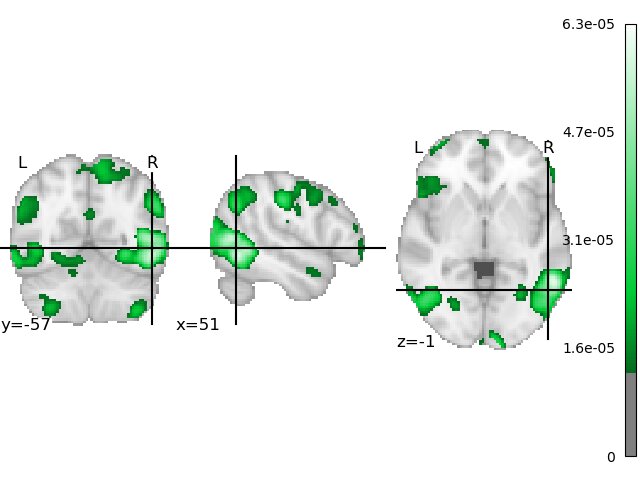

Supplement: S1 Components — (ZIP) [file pcbi.1008795.s002.zip › components/components_files/components_74_stat_map.jpg]

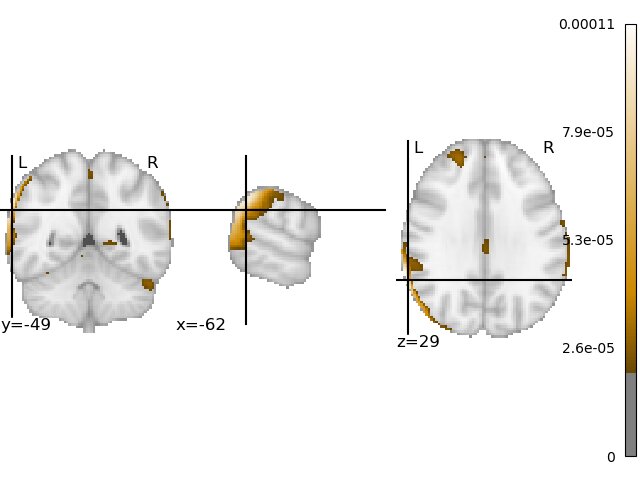

Supplement: S1 Components — (ZIP) [file pcbi.1008795.s002.zip › components/components_files/components_21_stat_map.jpg]

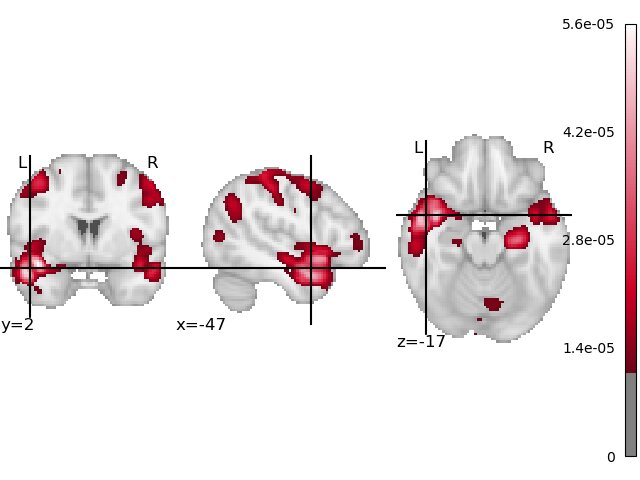

Supplement: S1 Components — (ZIP) [file pcbi.1008795.s002.zip › components/components_files/components_73_stat_map.jpg]

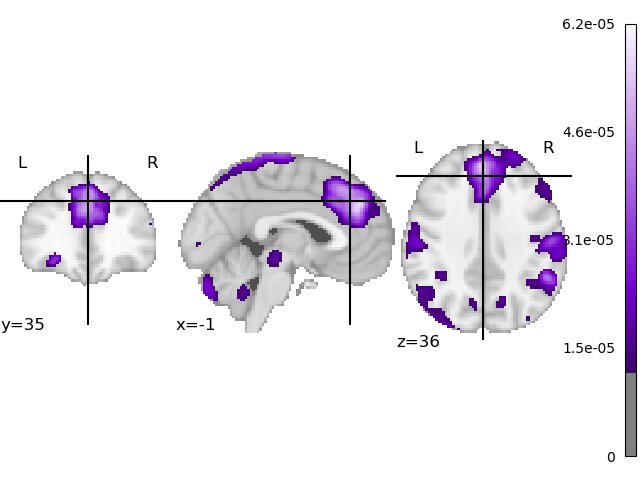

Supplement: S1 Components — (ZIP) [file pcbi.1008795.s002.zip › components/components_files/components_26_stat_map.jpg]

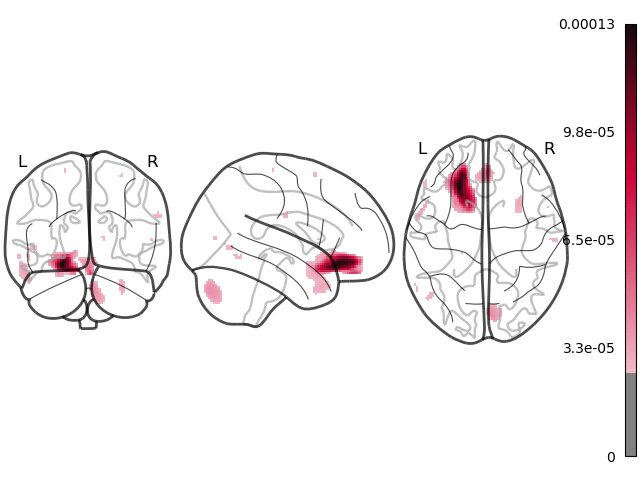

Supplement: S1 Components — (ZIP) [file pcbi.1008795.s002.zip › components/components_files/components_1_glass_brain.jpg]

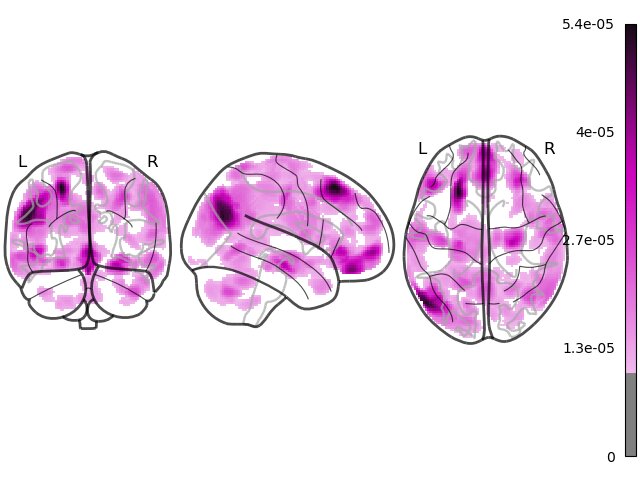

Supplement: S1 Components — (ZIP) [file pcbi.1008795.s002.zip › components/components_files/components_31_glass_brain.jpg]

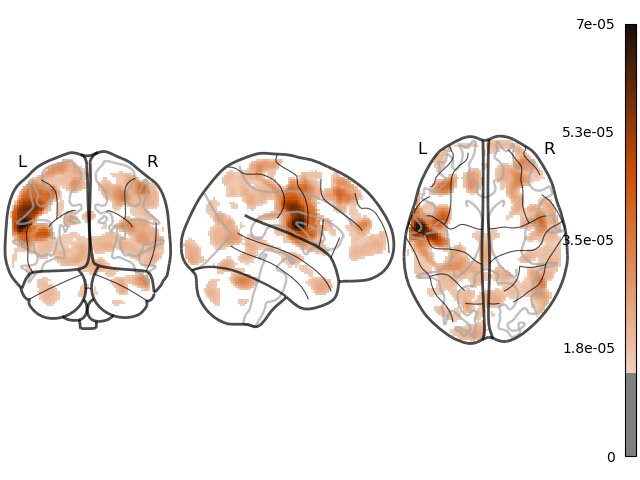

Supplement: S1 Components — (ZIP) [file pcbi.1008795.s002.zip › components/components_files/components_97_glass_brain.jpg]

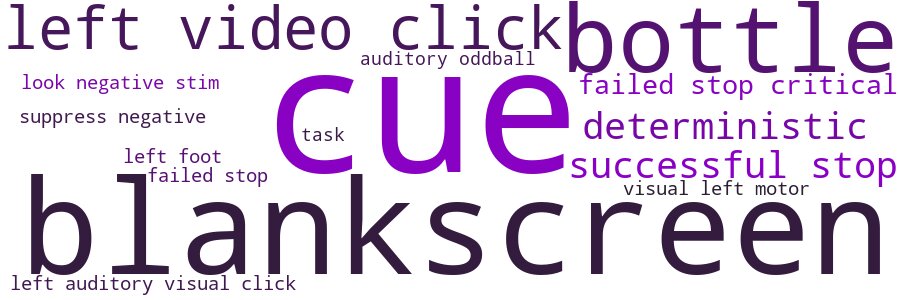

Supplement: S1 Components — (ZIP) [file pcbi.1008795.s002.zip › components/components_files/wc_cat_89.jpg]

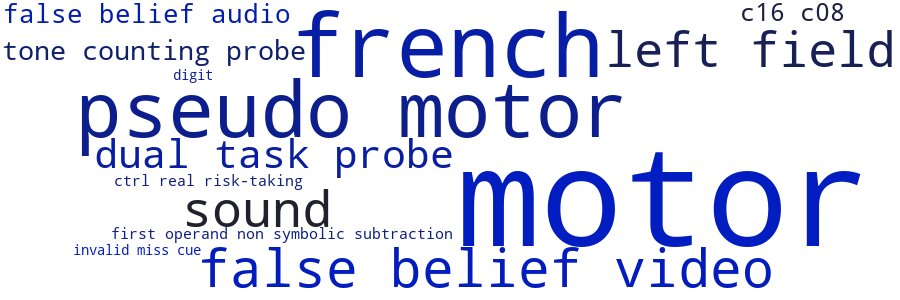

Supplement: S1 Components — (ZIP) [file pcbi.1008795.s002.zip › components/components_files/wc_cat_76.jpg]

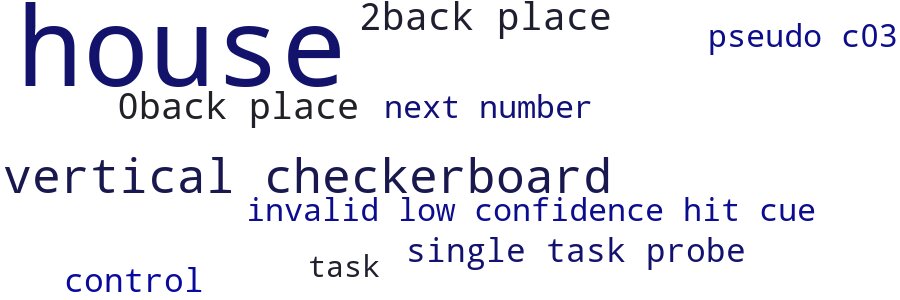

Supplement: S1 Components — (ZIP) [file pcbi.1008795.s002.zip › components/components_files/wc_cat_62.jpg]

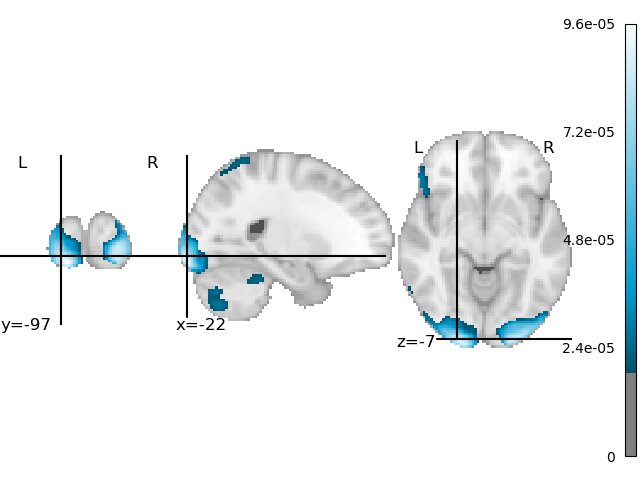

Supplement: S1 Components — (ZIP) [file pcbi.1008795.s002.zip › components/components_files/components_45_stat_map.jpg]

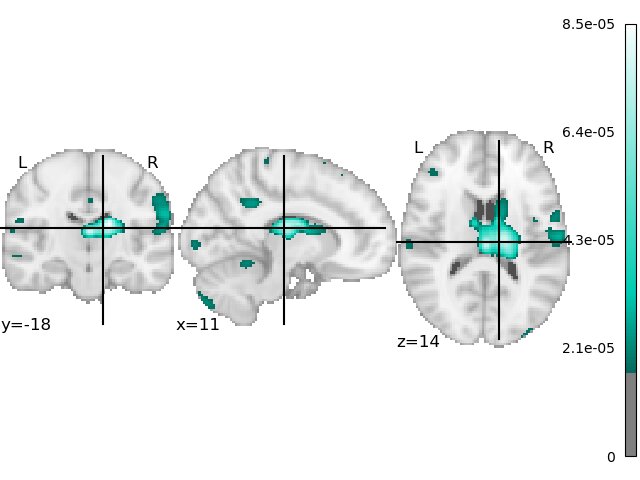

Supplement: S1 Components — (ZIP) [file pcbi.1008795.s002.zip › components/components_files/components_10_stat_map.jpg]

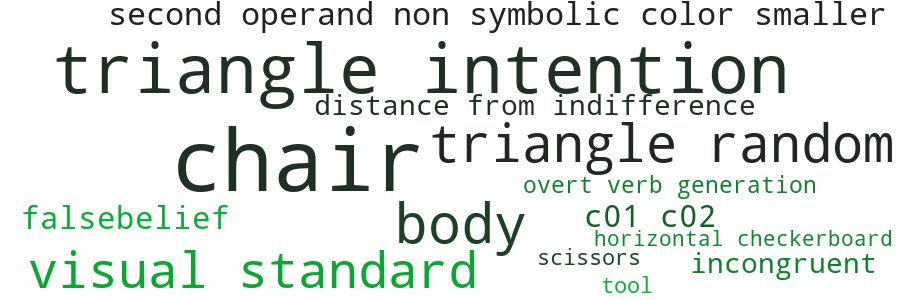

Supplement: S1 Components — (ZIP) [file pcbi.1008795.s002.zip › components/components_files/wc_cat_74.jpg]

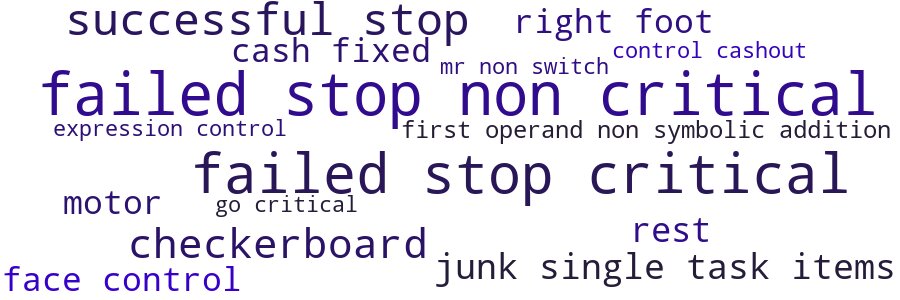

Supplement: S1 Components — (ZIP) [file pcbi.1008795.s002.zip › components/components_files/wc_cat_60.jpg]

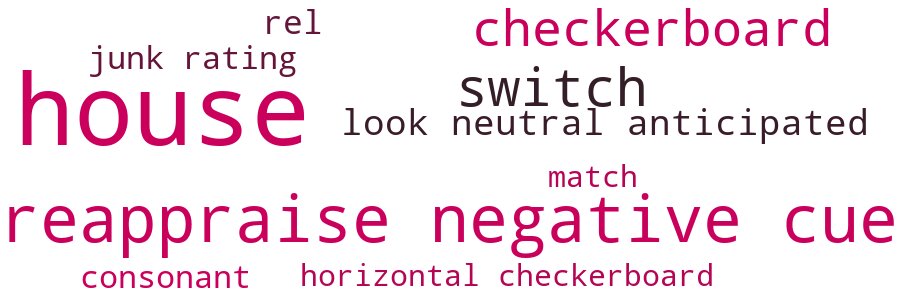

Supplement: S1 Components — (ZIP) [file pcbi.1008795.s002.zip › components/components_files/wc_cat_48.jpg]

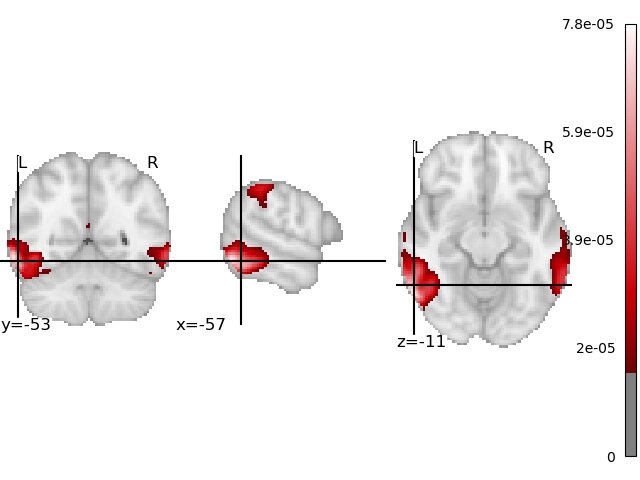

Supplement: S1 Components — (ZIP) [file pcbi.1008795.s002.zip › components/components_files/components_42_stat_map.jpg]

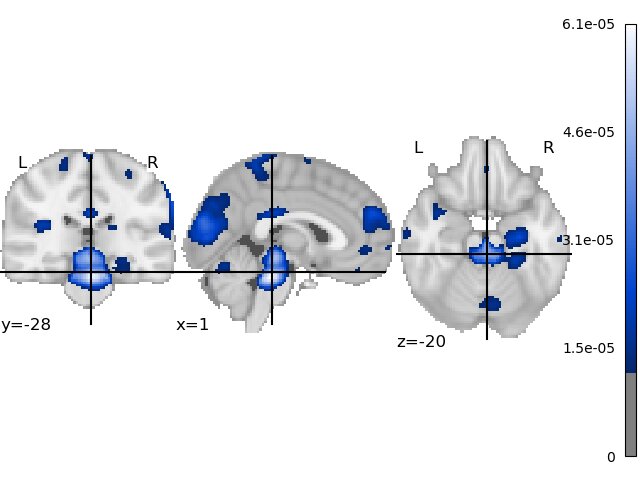

Supplement: S1 Components — (ZIP) [file pcbi.1008795.s002.zip › components/components_files/components_17_stat_map.jpg]

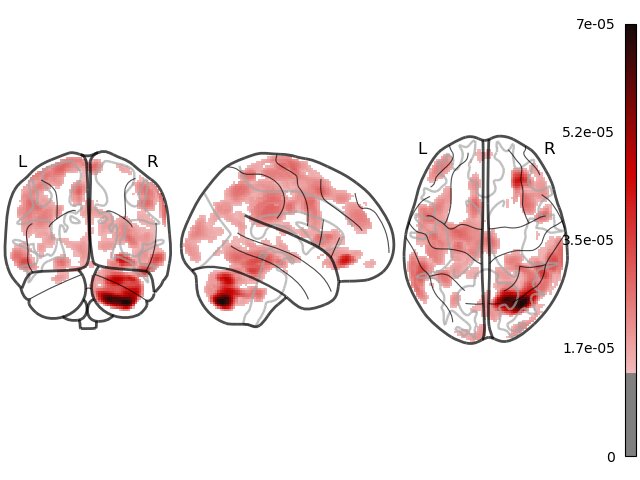

Supplement: S1 Components — (ZIP) [file pcbi.1008795.s002.zip › components/components_files/components_44_glass_brain.jpg]

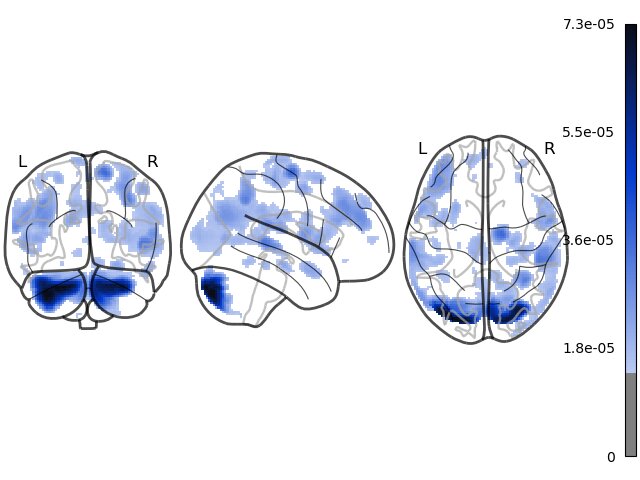

Supplement: S1 Components — (ZIP) [file pcbi.1008795.s002.zip › components/components_files/components_79_glass_brain.jpg]

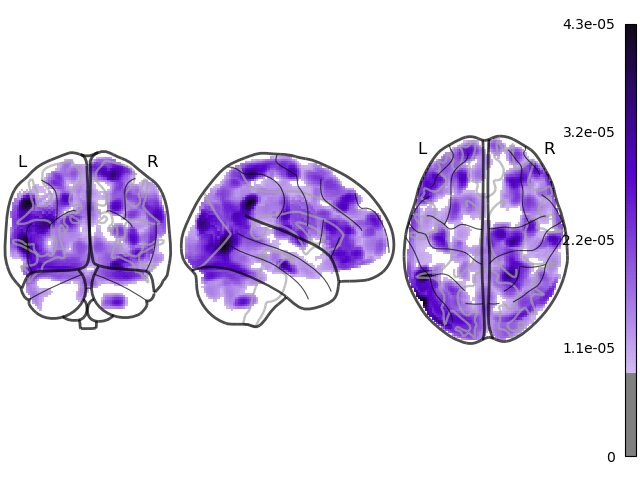

Supplement: S1 Components — (ZIP) [file pcbi.1008795.s002.zip › components/components_files/components_101_glass_brain.jpg]

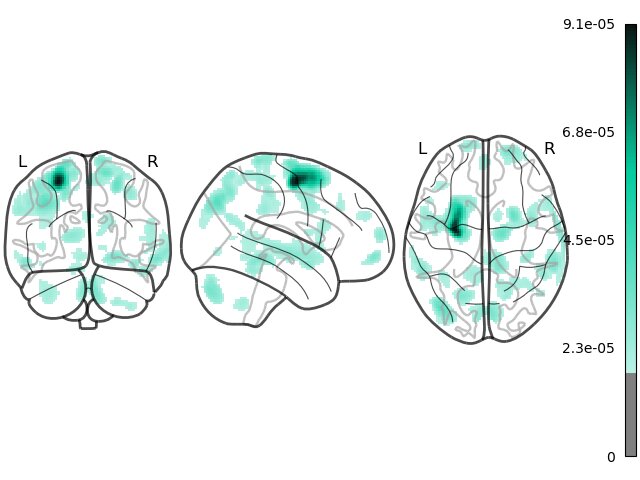

Supplement: S1 Components — (ZIP) [file pcbi.1008795.s002.zip › components/components_files/components_98_glass_brain.jpg]

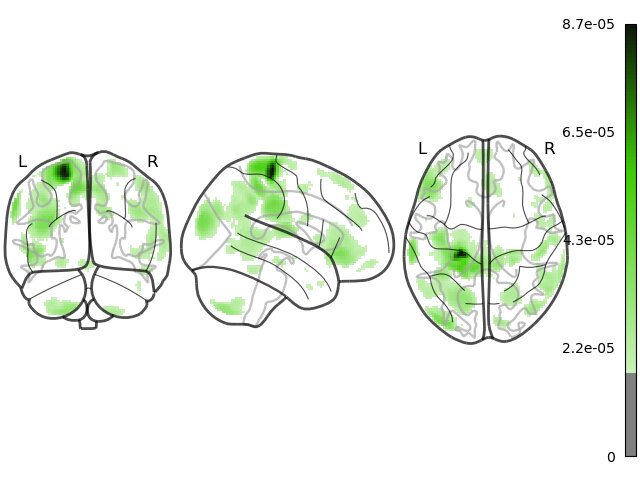

Supplement: S1 Components — (ZIP) [file pcbi.1008795.s002.zip › components/components_files/components_81_glass_brain.jpg]

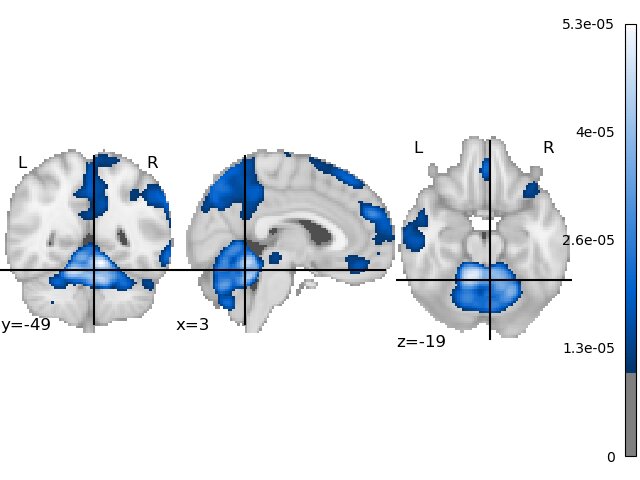

Supplement: S1 Components — (ZIP) [file pcbi.1008795.s002.zip › components/components_files/components_57_stat_map.jpg]

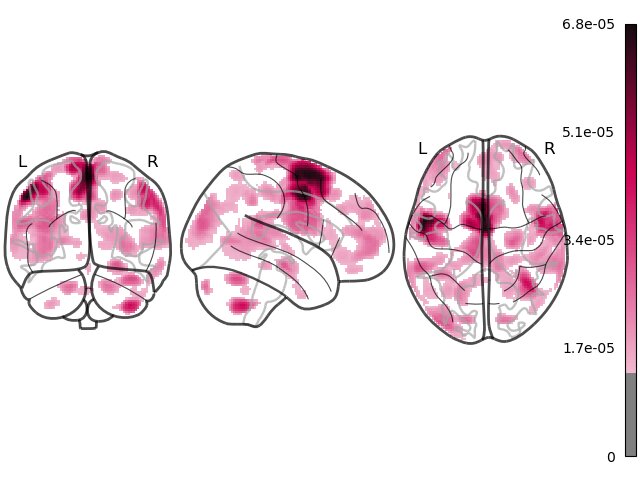

Supplement: S1 Components — (ZIP) [file pcbi.1008795.s002.zip › components/components_files/components_27_glass_brain.jpg]

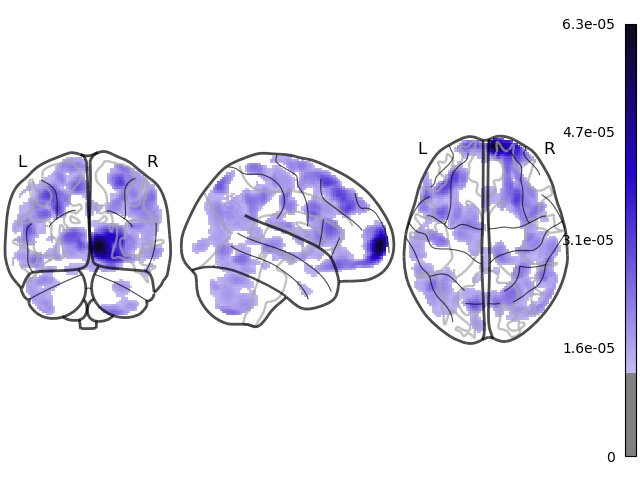

Supplement: S1 Components — (ZIP) [file pcbi.1008795.s002.zip › components/components_files/components_118_glass_brain.jpg]

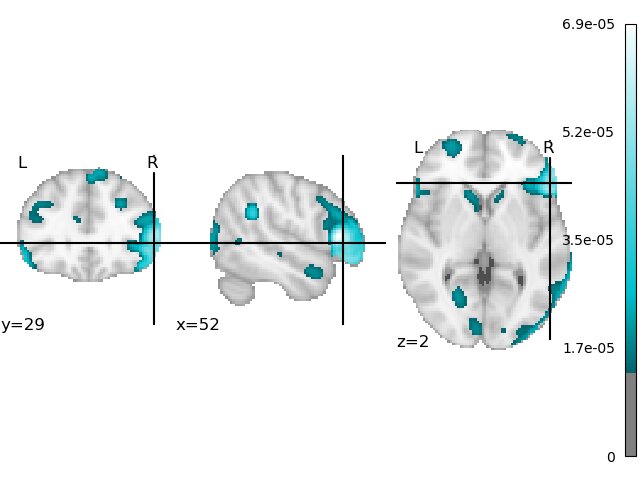

Supplement: S1 Components — (ZIP) [file pcbi.1008795.s002.zip › components/components_files/components_50_stat_map.jpg]

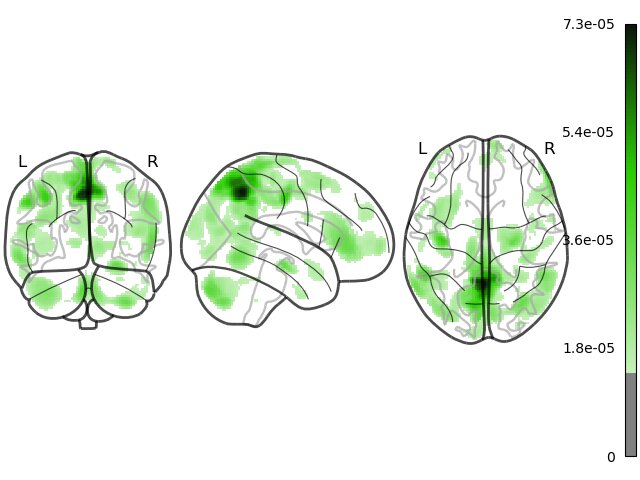

Supplement: S1 Components — (ZIP) [file pcbi.1008795.s002.zip › components/components_files/components_125_glass_brain.jpg]

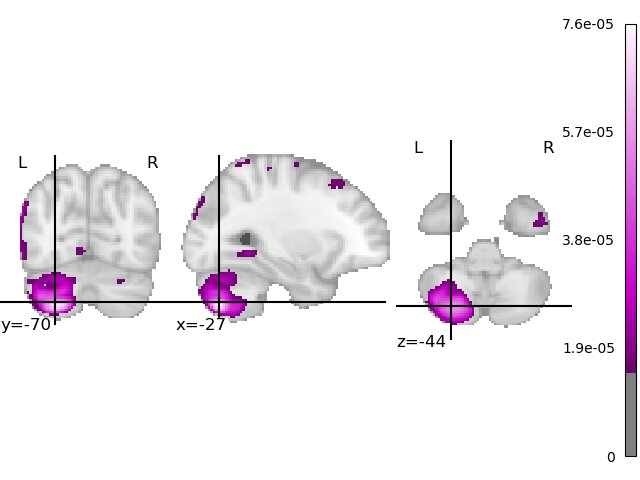

Supplement: S1 Components — (ZIP) [file pcbi.1008795.s002.zip › components/components_files/components_108_stat_map.jpg]

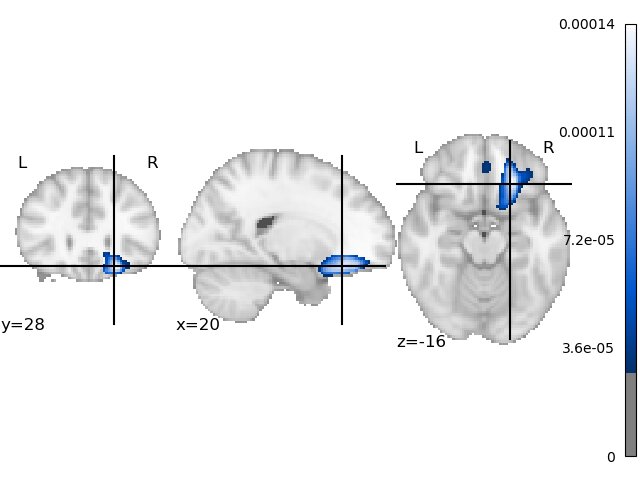

Supplement: S1 Components — (ZIP) [file pcbi.1008795.s002.zip › components/components_files/components_0_stat_map.jpg]

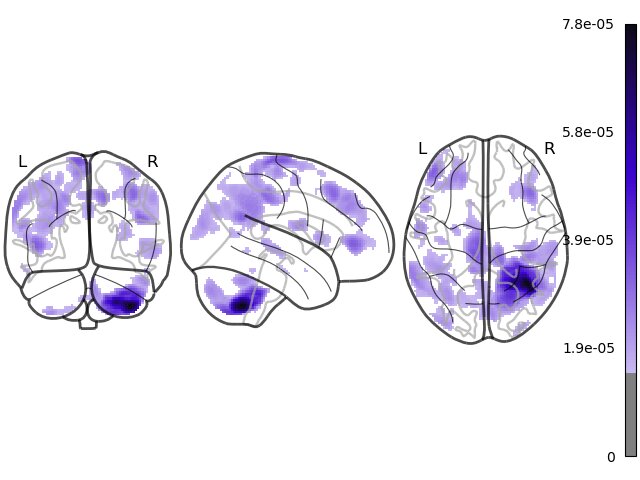

Supplement: S1 Components — (ZIP) [file pcbi.1008795.s002.zip › components/components_files/components_60_glass_brain.jpg]

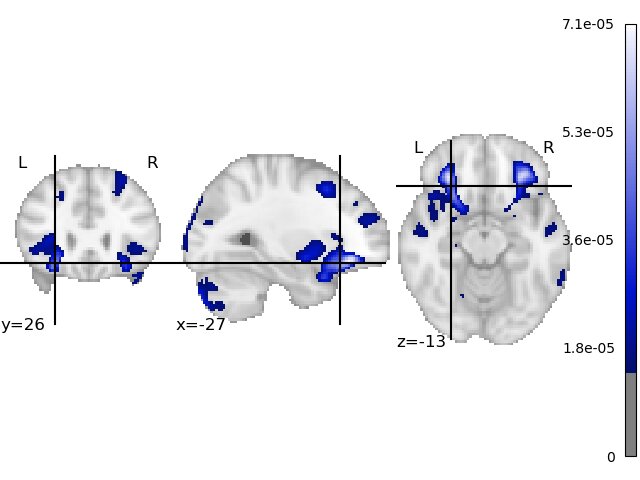

Supplement: S1 Components — (ZIP) [file pcbi.1008795.s002.zip › components/components_files/components_7_stat_map.jpg]

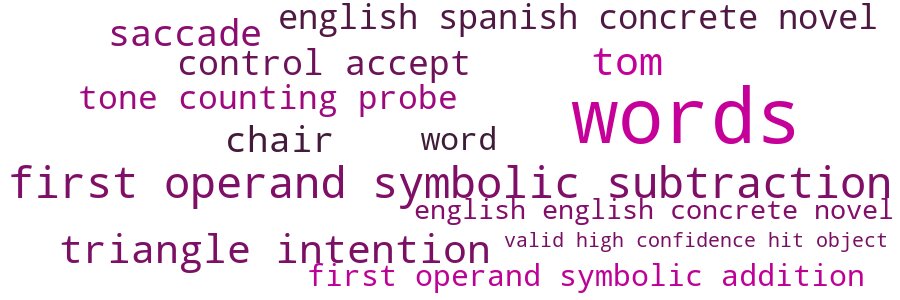

Supplement: S1 Components — (ZIP) [file pcbi.1008795.s002.zip › components/components_files/wc_cat_49.jpg]

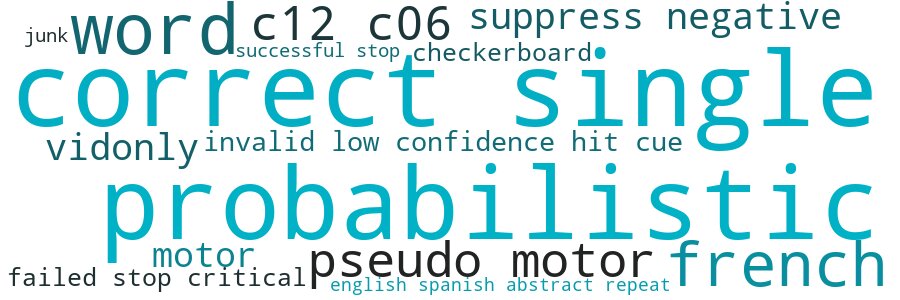

Supplement: S1 Components — (ZIP) [file pcbi.1008795.s002.zip › components/components_files/wc_cat_61.jpg]

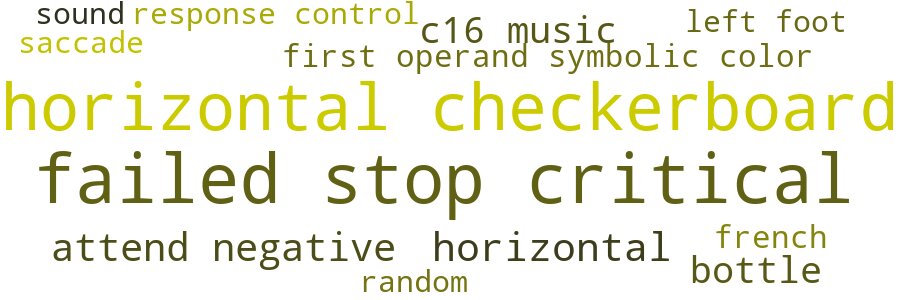

Supplement: S1 Components — (ZIP) [file pcbi.1008795.s002.zip › components/components_files/wc_cat_75.jpg]

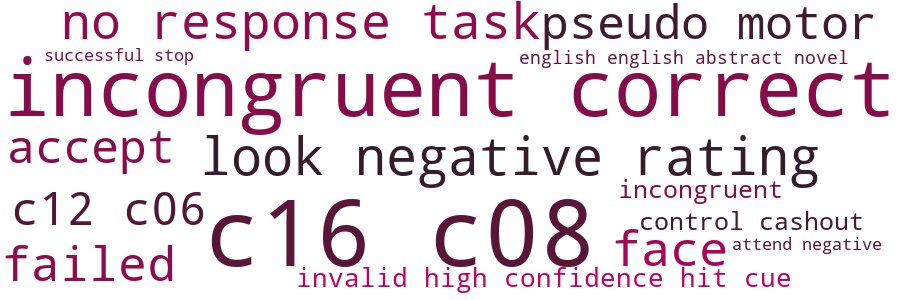

Supplement: S1 Components — (ZIP) [file pcbi.1008795.s002.zip › components/components_files/wc_cat_12.jpg]

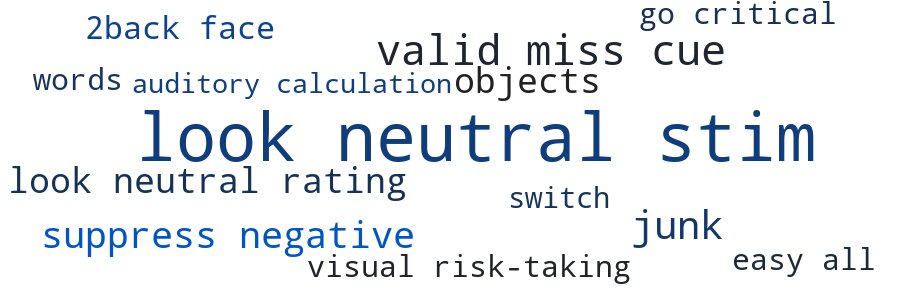

Supplement: S1 Components — (ZIP) [file pcbi.1008795.s002.zip › components/components_files/wc_cat_0.jpg]

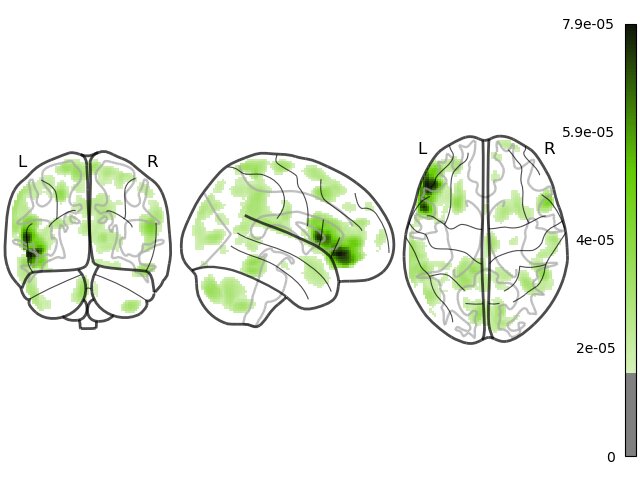

Supplement: S1 Components — (ZIP) [file pcbi.1008795.s002.zip › components/components_files/components_53_glass_brain.jpg]

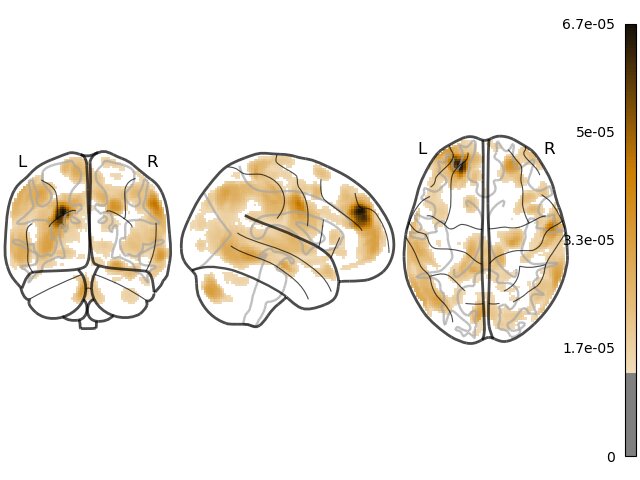

Supplement: S1 Components — (ZIP) [file pcbi.1008795.s002.zip › components/components_files/components_116_glass_brain.jpg]

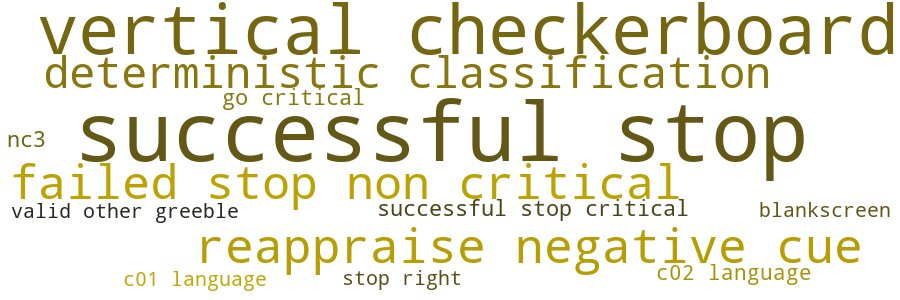

Supplement: S1 Components — (ZIP) [file pcbi.1008795.s002.zip › components/components_files/wc_cat_117.jpg]
