# Supplementary material for: Extracting representations of cognition across neuroimaging studies improves brain decoding
Source: PLoS Comput Biol. 2021 May 3;17(5):e1008795. doi: 10.1371/journal.pcbi.1008795 (PMC8118532; doi:10.1371/journal.pcbi.1008795)
Supplement: Attachment — Submitted filename: response.pdf [file pcbi.1008795.s004.pdf]

Response to reviewers

We thank the reviewers for their thorough reviews, that will greatly improve our submission. Below, we address the concerns and answers the question of each reviewer.

We have updated the title to ”Extracting Representations of Cognition across NeuroImaging Studies Improves Brain Decoding” (instead of ”Brain Mapping”).

We note that the width of the first layer is actually 465 and not 512, as we filter out resting-state components learned on the full-brain that do not intersect with grey-matter. We use a grey-matter mask for decoding. A comparison between using grey-matter and full-brain mask was present in the original submission, but we forgot to update the number 512 to 465 in the main text. This has been corrected.

1 Reviewer 1

The following two answers also address Reviewer's 2 concerns.

1. The choice of the dimensions in the first (i.e., 512 resting-state networks) as well of second layer (i.e., 128 task networks) in their model seems arbitrary.

a. How robust are resulting task-optimized networks for different decisions for k (i.e., 512) and l (i.e., 128)?

Choosing an initial dimension reduction is a difficult problem in its own. It involves finding the ideal size of Image Derived Phenotypes (IDP) for predictive analysis. We have studied this question thoroughly in our recent work (Dadi et al., 2020), where we introduce multi-scale dictionaries learned on very large datasets. Those dictionaries are obtained using matrix factorization, similar to what we do in the first layer of the model presented here. In particular, we show that high-order dictionaries ($k = 512, 1024$) outperform low-order dictionaries for decoding. When we submitted the present manuscript, the $k = 512$ dictionary (used to select 465 components) was the largest that we had estimated and we therefore chose to use it. We have added a reference to this work in Appendix S1.5.

To answer the reviewer's question, we have added a new figure (Fig. 12) displaying the performance of multi-study decoding varying the size of the latent space l. Choosing a low l leads to worse performances but more interpretable components. The performance reaches a plateau at $l \approx 128$. Setting a high l results in more scattered networks, so that different but similar MSTONs may be recruited to decode the same psychological condition (as the reviewer notes below). We therefore use $l = 128$, as if represents a good performance/interpretability trade-off.

b. My main criticism is that the large number of resulting task networks makes interpretation of the networks actually not straightforward. What would be the effect of lowering l? Also, when inspecting all networks on their website, I noticed that many components feature the same contrast as being the one that increases its likelihood the most (e.g. “house” for components 3/22/48/54/62/67 or “saccades” for components 43/68/78/124). Please discuss this.

The “saccade” task (in Papadopoulos Orfanos et al., 2017) triggers a very distributed response of the brain, which is the reason why it appears often in the word-clouds. The base condition “House” is part of the HCP (Van Essen et al., 2012) protocol. Decoding it versus the other HCP tasks gives a classification

map for which the whole visual cortex is positively activated, hence the appearance of the word House in MSTONs comprising a fraction of the visual cortex. We have added this discussion in Appendix S2.1.1, along with a new figure (Fig. 8).

c. What would be the effect of enforcing more/less overlap between the networks?

In the first layer, learned by dictionary learning, more or less overlap can be enforced by selecting a different regularization parameter λ. There is a trade-off between having overlaps but covering the brain entirely, and having no overlap but missing some part of the brain, as discussed in Dadi et al. (2020). As stated in the text, we chose λ to have full brain coverage but minimal overlap. Larger overlap would lead to more correlated activations before the second layer, and therefore a harder learning problem. With lower coverage, we would miss important information for some of the predicted psychological conditions. We refer to Dadi et al. (2020) for further discussion on selecting sparsity level when using dictionary learning in fMRI analysis.

Using the proposed post-hoc analysis, we obtain sparse and non-negative MSTON networks **LD**. Sparsity of the components and therefore overlap is regulated by another hyperparameter λ. As above, components with little overlap are easier to interpret but provides worse performance than components with larger overlap. We have added a comment on this aspect in Appendix S1.6. We tried 4 different values of λ and selected the MSTONs that were the most interpretable (see grids in Appendix S3.1). Note that the networks obtained using different λ show strong resemblance.

d. Please discuss if resulting networks can be interpreted straightforward or if they have a similar problem as weight maps for LDA/SVM have; where non-zero weights could also reflect noise suppression in that brain region (Haufe et al. 2014).

The non-negativity constrain limits the problems were non-zero weights are present to compensate for signal in another brain regions (also related to question 3.b). Indeed, such effect typically occurs with weights of different signs. Some of the regions extracted by dictionary learning (i.e. the first-layer) may capture noise patterns as they optimize a reconstruction objective. On the other hand, our supervised approach should favor the extraction of components that capture a signal useful for decoding; a priori, MSTONs do not correspond to noise patterns, although we have not verified it precisely. We have added a sentence on this aspect at the end of the discussion.

2. With regards to methods, I feel some parts need some clarification/additional information.

a. Each study is treated as a classification task and this implies that each study differs in the number of contrasts that needs to be classified; i.e., differs in its difficulty for decoding and its respective chance level. I would suggest making this more explicit in the manuscript (it was not immediately clear to me) and to highlight this in Fig. 2A by aligning studies according to their chance level and not absolute decoding accuracy.

Following the reviewer's comment, we have made more explicit the fact that tasks have variable difficulties, and added a supplementary figure, that is Fig. 2A with study sorted by chance level (Fig. 19). We observe a slight tendency of higher improvement for easy tasks, although no strong conclusion may be drawn.

b. Further with respect to my point in 2a, it was not clear to me if the classification objective in equation (4) (p.21) somehow takes into account the number of contrast that needs to be decoded on the study-level and does not skew optimization towards improving "easier" studies with fewer contrasts to decode (e.g., rhyme judgement with only 3 contrasts)?

Each "classification head" contributes a multinomial loss ℓ_j to the overall loss $\ell = \sum_j \ell_j$. The importance of the study j to find the latent parameter $\boldsymbol{L}$ depends on the amplitude of the gradient $\frac{\partial \ell_j}{\partial \boldsymbol{L}}$, which does not depend on the number of tasks c^j: in particular, for each study j, contrast $1 \leqslant k \leqslant c^j$ and subject $1 \leqslant i \leqslant n^j$, the susceptibility of the loss to the logits $l^j_{i,k}$ is such that $\frac{\partial \ell_j}{\partial l^j_{i,k}} \in [-1, 1]$ is independent from the number of tasks.

c. It would be also informative to see the decoding accuracy for contrasts separately and not just study-level and cluster them in a meaningful way (contrast loading on the same task-optimized network?). This could provide important insights into which contrasts are harder/easier to decode in the context of multi-study decoding.

Computing correlation between the loadings on task-optimized networks contrasts yields a figure similar to Fig. 6 (where correlation are computed between reconstructed classification maps). We have mentioned it in Fig. 6 caption. We have added a supplementary table (Table 4), giving the balanced-accuracy of different methods for each of the 545 contrasts in use.

3. In the Discussion section authors could elaborate on a few more points

a. Why do the authors think that contrasts that are moderately difficult to decode benefit from multi-study decoding? Is there a link with the number of subjects for those kind of contrasts (i.e., are moderate difficult to decode contrasts more prominent for studies with small N)?

We impute this effect to two causes: easy-to-decode studies do not benefit from the extra signal provided by other studies, while some studies in our corpus are simply too hard to decode due to a low signal-to-noise ratio. We have added this sentence to the result section.

b. What is the effect of non-negative matrix factorization? Is this a valid assumption?

Non-negative matrix factorization yields networks that are more interpretable as they do not contrast positive against negative regions. In particular, this limits the occurence of non-zero weights that reflect noise suppression (Haufe et al. 2014, see also answer to question 1.d). It is a valid way of segmenting the brain signal (akin to soft-assignment). Note that we also tried to use sparsity penalties only in the post-hoc analysis, with similar quantitative results but less interpretable components. We have added a sentence on this question in the description of the methods and in the discussion. Dadi et al. (2020) demonstrate the power of using non-negative functional networks (i.e. the first layer of this work) for many fMRI analysis tasks.

Minor:

1. The list of contrast per study and the number of actual contrasts investigated (i.e., Supplementary Table 1) should be moved to a more prominent part in the manuscript.

We have moved Table 1 to the main section.

2. That networks can be downloaded and visually inspected on their website should be mentioned in the Abstract and manuscript.

We now mention it in the ”Reusable tools and resources paragraph” and in the abstract

3. Ensemble model: In the results, this is referred to as consensus model and I would advise to mention this terminology in the main method section to avoid confusion.

We now use the terminology consensus everywhere.

4. Please mention how it was determined how many subjects were left out per study given difference in N (p.19)?

We use population half-split for all studies. We have clarified this in the main text and in the appendix.

5. The authors should consider moving important methodological details (e.g., the three-layer model description, on dropout and low rank constraints) to the main method section.

We now mention dropout and low-rank constraints in the main text. We have left mathematical description of the three-layer model in the appendix in the interest of readability, as we believe that the

equation p. 6 is sufficient to convey the general three-layer model definition.

6. The figures need clarification:

a. Fig. 3: Does standard decoder refer to voxel-level or resting-state? Please label more precisely.

Standard decoder refer to voxel-level decoding. We have clarified.

b. Fig. 4: Word clouds contain labels of contrasts, e.g. C01, C02 that are meaningless for a reader.

The names of these contrasts corresponds to sentence complexities in Devauchelle et al. (2009) (see Table 4 for a list of all contrasts), and are indeed meaningless as such. We endeavored to rename existing contrasts with clearer names in Fig. 4, but we have missed some. This has been corrected.

c. Fig. 5: What do blue and red colors refer to (positive and negative weights?)? How can the accuracy gain be +6.9% when baseline accuracy is 99.4% for Face vs House?

B stands for "balanced" and not baseline. We have clarified this in the caption.

We have corrected spelling mistakes pointed out by the reviewer.

2 Reviewer 2

1. The Materials and Methods section of the main text should describe the analytical methods used to produce the various results used to verify and explore the model, not just the methods of the decoder itself. Additionally, I would like to see the following methods issues clarified in the main text and in more detail:

We have added a validation paragraph to the method section, moving up contents from the result section.

a. The validation metrics are slightly more complex than simple classifier accuracy due to the multi-task nature of the model; the authors should present not only how they calculated their metrics, but also how they are interpreting these metrics.

Quantitative comparison of multi-study is indeed non-standard. We have measured variation in accuracy in each study, as a simple number is impossible to interpret. This is the reason why we show all the improvements/worsening in accuracy for each study in Fig 2B. We also report mean accuracy gain across studies (which is arguably hard to interpret), and the number of positive gain across studies. We have clarified this in the validation paragraph of the method section.

b. On page 7/Figure 2b, the authors reference that projecting data onto a set of resting state "functional networks" does not significantly improve decoding. From my understanding of the various supplement descriptions, these functional networks are the set of 512 and 128 components derived from resting state data using a three-layer model, described in supplements S1.4 and S1.5. The authors should make this clear, as readers may interpret these as being more akin to the canonical resting state networks usually referred to by this term in the literature. (see point 2 for additional discussion on interpretation of the components produced by the model)

We have been unclear on this aspect, but your understanding is correct. We have edited the legend into "Decoding from 512 fine-grain functional units".

2. The authors describe their layers 1 and 2 as representing a set of 512 "resting state networks" and 128 task-general "multi-study task-optimized networks" (MSTONs). Per the supplement S1.5, the resting state networks are produced in a way that minimizes overlap; the second layer does not appear to have this constraint. The authors should expand their discussion of how these layers relate to brain structure and function at different scales, and how this might practically contribute to the improvements in performance conferred by the task-optimized network decoder.

In the post-processing consensus phase, we extract a second layer that is sparse and non-negative to minimize overlap, yet provides sufficient brain coverage. We have clarified this aspect in the appendix. We have expanded our discussion about the impact of choosing the number of components, and added a figure showing the performance for various sizes of the second layer (Fig. 12). An in-depth discussion on the impact of the size of the first-layer is provided in (Dadi et al., 2020). We also refer the reviewer to the first answers to Reviewer 1.

I would also caution the authors to consider caveating or removing their use of the term "networks" for these components, which are rather far from the literature's intuition of brain networks as a concept. Specific discussion points I think would be worth discussing and some context for their inclusion here are listed below.

We agree that the term network can be misleading given its common understanding in the literature. On the other hand, the components that we learn can be understood as "supervised" functional networks, where typical "functional networks" are obtained in an unsupervised way. We have kept to the old terminology, but added a warning sentence to caveat our use. To prevent misunderstanding, we have replaced the term "functional networks" by "functional units" when dealing with the components of the first layer.

a. In the case of the resting state-derived Layer 1, the level of granularity is more similar to the region scale of brain parcellations, and in fact is even a higher resolution than many regional atlases. Thus, it might be more accurate and indeed fruitful to interpret this layer in a regional context; layer 1 of the model defines the boundaries of regions of the brain that show consistent activity within themselves at rest. Also, it is not mentioned in the methods nor the supplement that any spatial constraints were placed on the component weighting; if so, it seems in and of itself validating that the model naturally segregates voxels into small contiguous cortical areas.

No spatial constraint was used beyond sparsity and non-negativity. Using sparse matrix factorization to obtain such components is known to segregate contiguous regions (Varoquaux et al., 2011; Mensch, Mairal, Thirion, et al., 2016), although we have indeed used a larger dictionary than usual. At $k = 512$, the regions obtained are most often connected, which is not the case of e.g $k = 200$. They may therefore be interpreted as a "soft-parcellation", instead of a set of "functional networks" (as they are not distributed). We have clarified this aspect in the method section, and used the terminology "functional units".

b. In the case of the MSTONs from Layer 2, these appear to have more network-like coverage, but are not whole brain networks in the canonical sense. Based on my understanding of the model, the layer 1 weightings go into training the weightings for layer 2, so the task data used for these weightings is effectively constrained by predefined regions. The model then pulls out 128 components that represent consistencies in the data across tasks. It would indeed seem accurate to say that these components represent sets of regions that tend to have coordinated activity across tasks, i.e., functional networks. However, typical resting-state network parcellations have markedly fewer components, generally fewer than 20. These two types of components are certainly not mutually exclusive; while brain regions may naturally show preference for a particular network structure at rest, network allegiances likely shift as task demands require coordination across different sets of brain regions. The authors should discuss how their MSTONs relate to canonical whole-brain networks, and what they might contribute to understanding how regions coordinate their functions. They might also address how the overlap between MSTONs may relate to the various functions they support.

A systematic comparison between MSTONs and classical functional networks is an promising avenue for future work, but we may already make the following observations. A fraction of classical functional networks support non-Gaussian noise patterns in the BOLD time-series, and permits noise suppression. In contrast, MSTONs optimize a supervised objective and have therefore no reason to support noise patterns. Our qualitative analysis (Fig. 4) also reveals that MSTONs are skewed towards known coordinated brain networks, and differ from known resting-state networks. We have added this paragraph at the end of the discussion.

c. Related to the previous point, as the MSTONs appear to represent diffuse networks of regions, it

seems reductive to then label individual MSTONs with the name of an individual brain region. This detracts from the interpretation of the role of the MSTON as a whole, even if a single region or small set of subregions may be particularly strongly associated with that network. This is especially clear in the case of the "Left DLPFC" network in figure 1, which even in the manuscript is acknowledged as being equally associated with left parietal cortex. At the same time, it seems clear that the MSTONs tend to preferentially represent one or a few contiguous regions or their bilateral pairs, which would be expected as neighboring and hemispherically homologous regions tend to be more correlated with one another than distant regions. The authors should discuss the interpretation of their MSTONs from both a network perspective and a region-level perspective, as it seems like they may serve as a stepping stone between the two. They also might consider removing the explicit region-name labels from figure 4 and instead let the word clouds serve to highlight the functional contributions of the MSTONs.

We agree with the reviewer that seeings MSTONs as "regionalized" misses a part of their interest. They form a network of scattered regions, that are jointly used to classify tasks based on bain images. We have adopted the point of view of the reviewer in introducing MSTONs (p. 4). We have added a discussion on the interpretation of MSTONs from both perspectives. The region names from Fig. 4 label salient parts of the distributed networks in display. We have changed them to stress that they are only parts of the networks in display.

3. While the improvements to classification accuracy are relatively clear, it is less obvious to me how to interpret the differences in the classification maps between the voxelwise and task-optimized network decoders. The authors seem to suggest that the classification maps from the task-optimized network decoder are inherently better than those of the voxelwise decoder outside of a small set of localizers, appearing smoother and better capturing the boundaries of relevant functional regions. This is likely being driven by the data reduction steps in the first layer of the model, which associates groups of voxels with contiguous brain regions (see point 4 below for additional discussion). This would appear to bias any classification map to smooth, well-bounded regions, regardless of ground truth. The authors should address this bias in their discussion of these maps. I would also suggest that the authors include a map of the univariate contrast maps from the studies presented in Figure 5 for comparison, as this would show which regions are explicitly more active in one condition versus the other. Notably, a similar bias from the layer 2 networks would likewise explain why the task-optimized network decoder maps tend to produce networks of regions even in contrasts that should show fairly focal results, in line with the authors' maps from the vertical checkerboard study.

Using MSTONs introduces two biases: smoothness (due to the first "resting-state" layer) and scattering (due to the second layer). These biases improve upon the classification accuracies (as noted above, smoothness is not sufficient for improvement), in particular because the network repartition is actually learned on many images. This can have a negative effect, in particular when the task to decode is associated with very focal patterns. However, the fact that these biases overall improve classification accuracy is evidence to consider them as useful biases in general. We display z-maps and their projections in a supplementary figure (Fig. 10), that we have better referenced in the text.

4. In most cases (if not all), the task-optimized network decoder is trained on more data than the individual study models by definition, which could trivially contribute to its improved performance outside of potential benefits from the additional layers. This would likewise be magnified for comparisons with datasets from smaller sample sizes, as was shown in Figure 2C, and would also explain the relatively small benefits seen for the HCP and Cam-Can data. The authors could train their decoder on a subset of the total available data and assess its performance versus a comparatively sized single study model, or should otherwise find some way to address this potential confound.

We may indeed train a multi-study models on a subset of all studies, capping their size at 15 subjects. We have done so and added supplementary results (Fig. 18). Transfer learning is in this experiment positive for all studies. This includes large studies, for which transfer learning is uneffective when considering all available subjects (e.g. HCP, Fig. 2 and data from the UCLA consortium). The multi-study approach is therefore especially efficient for studies of average size as of 2020.

Note however that the fact that adding data from a different study, with a different paradigm, improves

a give study in general is non-trivial. The different studies ask fundementally different questions, framed in different settings.

Minor Suggestions

1. Given the clear relevance of sample size for the extent of decoder accuracy improvement, it would be helpful for the authors to list the sample sizes from each study as a part of figure 2a.

We have moved Table 1 from the supplementary material to the main text.

2. The use of the subfigure labels for figure 4 seems unnecessary, and I would recommend simply removing them. However, if the authors wish to retain them, the caption for figure 4 should likewise include subcaptions for parts a, b, and c, to make it clear what these parts are referencing.

We have added subcaptions to the caption of Fig. 4.

4. The authors should clarify which template space (MNI, Talairach, other) their task data are registered to (S1.1).

We use the MNI IBC152 grey-matter mask. We now state it in the method section.

3 Reviewer 3

The authors have set up a very ambitious agenda, to determine "universal cognitive representations" and suggest "While extracting a universal basis of cognition is beyond the scope of a single fMRI study, we show that a joint predictive model across multiple studies finds meaningful approximations of atomic cognitive function". However, careful examination of Figure 4 suggests a starkly different reality. Linking motor cortex to motor behaviour is good proof of concept, but is not a cognitive neuroscience discovery. Outside of motor behaviour, other cognitive functions not meaningfully represented. What stands out is how little meaningful information can be extracted from the word clouds aside from rudimentary functional localization, for example faces and fusiform cortex, with conceptual noise (associated labels).

MSTONs make a step towards "universal cognitive representations", yet we agree that we are still far from universality and the road is long. To avoid overstatements, we have toned it down. Our primary purpose is to show that decoding performance can be improved by multi-study training, and that such training also yields networks that are partially interpretable. The results are indeed noisy; it is a consequence of using raw contrast labels and not using an ontology. However, it is also the case of functional networks (e.g. obtained by ICA) used ubiquitously in the literature. Quantitatively, the networks that we obtain form a good low-rank base for projecting the brain signal before decoding.

My primary concern involves the task contrasts that are entered into the model and the use of the cognitive labels. Are the task contrasts used in the current study following the experimental intent of using a tight contrast to determine precise cognitive engagement of brain regions? Or, are the authors contrasting every condition with every other condition for each subject? For example, in the HCP data, social cognition can be "localized" by comparing the mental interaction blocks with the random interaction blocks. However, contrasting geometric shapes moving with intention with tools from the working memory condition will not provide meaningful neural information about mentalizing or tools. This would confound the labels and set up a "garbage in" scenario. I do not know if this contrast was used to train the model. 23 contrasts were used from HCP but no additional detail is provided in supplemental. The tasks of interest in the appendix are listed by site or task. However, many sites have multiple tasks. All tasks, as well as all contrasts, should be listed.

We agree with the reviewer that the list of tasks and contrasts should have been given in the supplementary material, and we have added it (Table 4). In our setting, we use a single multinomial regression per site, for the sake of simplicity. For instance, this amount to classify each of the 23 base conditions

of the HCP dataset against the 22 others, in a ”one versus all” setting, but not opposing every pair of condition. One way of thinking about this approach is that all conditions but one are used to create a baseline for that latter condition.

Some of the studies we consider indeed feature different tasks (see Table 4) We agree with the reviewer that there is a great diversity in the inputs and that only some of the oppositions correspond to psychological manipulations with a natural cognitive interpretation. Yet, when defining a baseline, combining a variety of condition may lead to less crisp results but not invalid ones. The validity of the oppositions is confirmed by the successful decoding: when decoding within contrats from a single site, an activation map is almost never mistaken with classification maps from the same site but from a different task. The hard part of the classification task is deciding between conditions from the same task, even though we use a single multinomial regression model per site. We note that our approach has been used in single-study decoding, e.g. by Bzdok et al. (2015) on HCP data.

On the other hand, we agree with the reviewer that an additional test of our method using one classification objective *per task* provides interesting information. We have added an experiment on this aspect. The performance improvement is similar (see Fig. 13 in the new paragraph Appendix S2.3). We have referenced this experiment in the discussion.

Fig. 4 only shows a selection of networks. The orbitoparietal networks and default mode networks also appears among the MSTONs (see `https://cogspaces.github.io/assets/MSTON/components.html`, for instance components 10 and 24), but are associated with many different contrast names. We therefore deamed them less interesting to be shown in the main text. We have insisted on this point in the text and added a supplementary figure (Fig. 7) with the MSTONs cited above, with this discussion (Appendix S2.1.1).

The authors suggest that fMRI studies of 30 subjects or less “lack statistical power”. It is more accurate to say they have low power. The authors additionally suggest in the introduction that power has been decreasing in cognitive neuroscience. This is not exactly accurate. The effect sizes that cognitive neuroscientists are investigating are smaller, which is true for most maturing fields of science. So, while the effect sizes are smaller, sample size is most certainly increasing. It is possible that the ratio of smaller effect sizes to increases in sample is overall decreasing power. However, this is a matter of debate and likely varies across areas of inquiry. More thoughtful nuance would be appreciated. The authors’ general tone toward the field is somewhat disrespectful, which is surprising considering how reliant the authors are on these data.

We are sorry that our tone was perceived as disrespectful. We are of course thankful for the data provided by many different groups, and glad that the neuro-imaging community has been moving towards more data sharing, as it was a *sine qua non* condition for our work. The point of our paper is to show that it is beneficial to consider many studies at once, as it quantitatively improves decoding, and provide a reasonably interpretable brain decomposition.

It has been pointed out by Button et al. (2013) that the size of some neuro-imaging studies is often not sufficient to reliably investigate small effect sizes. In addition, Poldrack et al. (2017) show that the increase in sample is indeed not enough to compensate for the decrease if effect size, leading to a decrease of power. This raises a problem of reproducibility, that our work hopes to tackle in part. In training a model on many studies, we bring valuable information to studies with few subjects, which results in strong accuracy improvement (see Fig. 2C). It may thus be more reliable to analyse small studies using our approach. In practice, multi-study training injects a prior on classification maps, that become both smooth and supported on meaningful scattered networks (Fig. 5). Following the reviewer comments, we have adapted our introduction to make it more nuanced.

It is unclear how much of the current work is an innovation, or an incremental improvement on the tools and algorithms developed over many years by the research team. This should be spelled out much more clearly in the introduction and discussion.

The current work reuses some components of what the team developed in the previous years. In par-

ticular, the first reduction layer is trained using sparse matrix factorization, using an algorithm from (Mensch, Mairal, Thirion, et al., 2018). The multi-study approach was crafted in a short conference paper (Mensch, Mairal, Bzdok, et al., 2017), with substantial differences and much less ambition in the validation. This was stated in the original submission (Appendix S2.9). We have referenced this paragraph in the method section following the reviewer's comment. Yet, the present manuscript not only contributes a method with important changes –necessary to improve accuracy and bring interpretability– to our previous work, it also gives a much more detailed empirical evaluation of the method and interpretation of its results.

References

Button, Katherine S., John P. A. Ioannidis, Claire Mokrysz, Brian A. Nosek, Jonathan Flint, Emma S. J. Robinson, and Marcus R. Munafò (2013). "Power Failure: Why Small Sample Size Undermines the Reliability of Neuroscience". In: *Nature Reviews Neuroscience* 14.5, pp. 365–376.

Bzdok, Danilo, Michael Eickenberg, Olivier Grisel, Bertrand Thirion, and Gaël Varoquaux (2015). "Semi-Supervised Factored Logistic Regression for High-Dimensional Neuroimaging Data". In: *Advances in Neural Information Processing Systems*, pp. 3348–3356.

Dadi, Kamalakar, Gaël Varoquaux, Antonia Machlouzarides-Shalit, Krzysztof J. Gorgolewski, Demian Wassermann, Bertrand Thirion, and Arthur Mensch (2020). "Fine-grain atlases of functional modes for fMRI analysis". In: *To appear in NeuroImage*. arXiv: 2003.05405.

Devauchelle, A. D., Catherine Oppenheim, Luigi Rizzi, S. Dehaene, and Christophe Pallier (2009). "Sentence Syntax and Content in the Human Temporal Lobe: An fMRI Adaptation Study in Auditory and Visual Modalities". In: *Journal of Cognitive Neuroscience* 21.5, pp. 1000–1012.

Mensch, Arthur, Julien Mairal, Danilo Bzdok, Bertrand Thirion, and Gaël Varoquaux (2017). "Learning Neural Representations of Human Cognition Across Many fMRI Studies". In: *Advances in Neural Information Processing Systems*, pp. 5883–5893.

Mensch, Arthur, Julien Mairal, Bertrand Thirion, and Gaël Varoquaux (2016). "Dictionary Learning for Massive Matrix Factorization". In: *Proceedings of the International Conference on Machine Learning (ICML)*.

— (2018). "Stochastic Subsampling for Factorizing Huge Matrices". In: *IEEE Transactions on Signal Processing* 66.1, pp. 113–128.

Papadopoulos Orfanos, Dimitri, Vincent Michel, Yannick Schwartz, Philippe Pinel, Antonio Moreno, Denis Le Bihan, and Vincent Frouin (2017). "The Brainomics/Localizer Database". In: *NeuroImage* 144, pp. 309–314.

Poldrack, Russell A., Chris I. Baker, Joke Durnez, K. J. Gorgolewski, Paul M. Matthews, Marcus R. Munafò, Thomas E. Nichols, Jean-Baptiste Poline, Edward Vul, and Tal Yarkoni (2017). "Scanning the Horizon: Towards Transparent and Reproducible Neuroimaging Research". In: *Nature Reviews Neuroscience* 18.2, pp. 115–126.

Van Essen, David C., Kamil Ugurbil, E. Auerbach, D. Barch, T. E. J. Behrens, R. Bucholz, Acer Chang, Liyong Chen, Maurizio Corbetta, and Sandra W. Curtiss (2012). "The Human Connectome Project: A Data Acquisition Perspective". In: *NeuroImage* 62.4, pp. 2222–2231.

Varoquaux, G., A. Gramfort, F. Pedregosa, V. Michel, and B. Thirion (2011). "Multi-Subject Dictionary Learning to Segment an Atlas of Brain Spontaneous Activity". In: *Proceedings of the International Conference on Information Processing in Medical Imaging* 22, p. 562.
