# Supplementary material for: Extracting representations of cognition across neuroimaging studies improves brain decoding
Source: PLoS Comput Biol. 2021 May 3;17(5):e1008795. doi: 10.1371/journal.pcbi.1008795 (PMC8118532; doi:10.1371/journal.pcbi.1008795)
Supplement: Attachment — Submitted filename: response.pdf [file pcbi.1008795.s005.pdf]

Response to reviewer 3

We thank reviewer 3 for asking the difficult questions and challenging our statements, in particular given that our last submission had editing errors. We have addressed the substance of the reviewer's comments by modifying the manuscript in multiple places, including reworking completely our discussion on the high-level functional structures present in the MSTONs (fig 5), and better discussions on the interpretations of our results. We answer the review below, providing first a general answer, and then addressing specific concerns more precisely.

We may have been unclear on the nature of the networks that our method extracts. Those networks are tuned to be useful for decoding many different tasks. Some corresponds to low-level functional networks (e.g. motor, visual and audititive tasks), and are associated to word clouds simple to interpret. Other networks are more distributed and corresponds to higher-level brain circuits. Yet a few other MSTONs do not seem to relate to known functional structures, and may be adjusting to peculiarities of the fMRI signal.

The more distributed networks can often be related to known patterns of activation (for instance parts of the default mode network), but the word-clouds generated in association are harder to interpret as they include a broader set of tasks, e.g. those relying on memory and executive tasks. Some groups of words may still be identified that corresponding to well-known associations between cognitive functions and brain regions, as we discuss in a newly-added Figure 5. It is important to keep in mind that the words displayed in the figures are the raw terms used in the contrast names by the investigators; they come with lack of contract and are not always easy to interpret.

We have explain these aspects better in the paragraph of the manuscript around line 331, as well as in the caption of Figure 4 and in Figure 5.

As we now clearly acknowledge in the main text, our interpretation effort is more successful for lower-level than for higher-level brain functions. Better interpretability may be favored by reducing the number of MSTONs (at the cost of a certain loss in decoding performance). We expect this interpretability to improve by using a larger corpus of fMRI studies, as characterizing well a high-level brain region typically requires multiple tasks. We stress that the word-clouds presented in Fig. 4 and Fig. 5 mostly serve for illustration purposes, and, as we noted in the caption of Fig. 4, their interpretation may be difficult without good knowledge of the original studies.

Conversely, an important part of the evaluation of our approach is quantitative (Figure 2 and 3), and a part of the interpretation that we provide goes through the analysis of classification maps (Figure 6). We have insisted on that point in the main text.

To reply specifically to the points raised by the reviewer, the reviewer notes that supplemental figure 7 in the previous was not convincing. We agree.

There does seem to be a partial reply appended beneath the previous one stating: "Fig. 4 only shows a selection of networks. The orbitoparietal networks and default mode networks also appears among the MSTONs (see `https://cogspaces.github.io/assets/MSTON/components.html`, for instance components 10 and 24), but are associated with many different contrast names. We therefore deamed them less interesting to be shown in the main text. We have insisted on this point in the text and added a supplementary figure (Fig. 7) with the MSTONs cited above, with this discussion (Appendix S2.1.1)."

However, this statement is not responsive. On examination of Supplemental Figure 7, it is stated "Fig. 7. Examples of MSTONs that recruits well-known patterns of brain activity.: A. Default Mode Networks and B. Right orbitofrontal cortex". However, the maps are inconsistent with these anatomical terms, and the corresponding word clouds inconsistent with the functions of the named networks. A. Default Mode Networks depicts middle cingulate with the executive function "Switch and dual task probe" (NOTE: Core regions of the default network are posterior cingulate and medial prefrontal cortex and the inferior parietal lobule; Buckner et al., 2008, ANYAS)

The component that we captioned "default mode network" comprises a part of the posterior cingulate (e.g. MNI coordinate $(0, -27, 37)$), which typically visible in the default-mode network, extending forward from the precuneus along the cingulate gyrus. Yet, the reviewer is right in noting that this component only corresponds to a small part of the DMN, and is not representative. Other MSTONs are more representative of nodes of the default mode network, among the 128 components that we extracted. The new Fig. 5 displays networks more clearly associated with the default mode network.

...while the Right orbitofrontal cortex image depicts not orbitofrontal cortex but a subcortical region with the term "Bottle". Were these figures generated in error? Or, are the authors asserting that these "well-known patterns of brain activity" are consistent with the cognitive neuroscience literature? If the latter is the case, then this needs to be supported by references to the literature. The structure function associations suggested in this figure are not well-known to this reviewer.

We agree with the review that this component (component 10 of `https://cogspaces.github.io/assets/MSTON/components.html`) is not very meaningful. It indeed does not correspond to a grey matter region identifiable to a functional unit. In addition, this component is indeed not related to the orbitofrontal cortex. A component related to part of the orbitofrontal cortex is component 18, and it is associated with terms relating to tasks comprising complex decision processes. The use of component 10 was probably a communication mistake between the authors of the manuscript and we apologize for this mistake.

Again, Figure 7 is not what the authors suggest they are depicting, and there is no depiction of the frontoparietal cortex at all. I would like to see the default mode network and the frontoparietal control networks' topography and functions accurately depicted.

We have removed this network, and rather discuss meaningful yet higher-level networks in a new Fig. 5, with units of the default mode network and fronto-parietal networks.

In appendix 2.1.1, the author write: "Well-known patterns of brain activity. Some MSTONs are also composed of brain regions that are activated in many different tasks, such as the default mode networks or the orbitofrontal cortex. We show two examples of such networks in Fig 7, along with word-clouds of associated contrasts. As expected, the associated contrasts do not correspond to identifiable brain functions." There appears to be a significant contradiction between the notion that there are "Examples of MSTONs that recruits well-known patterns of brain activity" AND "As expected, the associated contrasts do not correspond to identifiable brain functions.

These components were bad choices that did not illustrate known functional structures associated with high-level functions. As discussed above, some MSTONs corresponding to low-level cognitive functions, are related to easily interpretable set of tasks. MSTONs corresponding to higher-level functions are related to a more diffuse set of tasks, harder to interpret. This dichotomy was not expressed clearly enough in the text, and may have been interpreted as a contradiction. We have clarified this in the result section accompanying Fig. 4 and Fig. 5.

I return to my original critique... The method employed here draws in information linking executive function or memory to the brain. These higher order cognitive functions have robust and reliable patterns of brain activity. Conversely, cognitive functions linked to the frontoparietal control or default mode networks are also reliable and robust. Convergence between these cognitive functions and brain regions have been demonstrated from a century of lesion work, 3 decades of neuroimaging, and more recently with intracranial EEG. If the present approach is unable to recapitulate them, there is a problem with the

method.

The new Fig. 5 shows MSTONs that corresponds to high-level cognitive functions. In particular, some components outline part of the DMN and of fronto-parietal networks. Word-clouds associated to these networks are somewhat interpretable, as now discussed in the main text.

However, we find that the reviewer's expectation do not do justice to the work: a single data processing can hardly be faithful to a history of investigations across multiple data modalities. While the default mode network is indeed a very salient general structure of brain activity, it has been broken down in different ways into subnetworks (Andrews-Hanna et al., 2010; Leech et al., 2011). Isolating the fronto-parietal network has taken a couple of decades of functional imaging, with thousands of increasingly sophisticated studies. Our approach was applied to only 35 fMRI studies touching a wide variety of functions. It operates on fMRI, which probes much more indirect structure-function links than lesion studies (albeit with the benefit of being suitable for healthy controls). It is not tailored to tease out a specific brain system or mental function. Rather, it strives to provide a good basis for general-purpose decoding and we have shown quantitatively that it does improve decoding on new studies touching a wide variety of topics. We do believe that the promising results of figure 5 suggest that with more data the method will outline with increasing sharpness high-level networks such as those related to executive and control functions. Yet, it is non a trivial task to start from functional-imaging data without prior information and decompose the brain in networks that predict the mental processes underway, from low-level to high-level.

These cognitive functions and networks should be depicted and in a transparent manner (not spread between the main text, appendices, and supplemental figures).

The supplementary Fig. 7 has been removed, replaced by Fig. 5 in the main text. For the sake of clarity, the HTML page with all networks, corresponding to the page `https://cogspaces.github.io/assets/MSTON/components.html` is now provided in the supplementary material.

If the method cannot associate the two, this is a very significant limitation that needs to be openly acknowledged and discussed in the main text.

We now acknowledge in the main text (around line 331) that our word-cloud generation approach provides more interesting results for lower-level than for higher level MSTONs.

References

Andrews-Hanna, Jessica R, Jay S Reidler, Jorge Sepulcre, Renee Poulin, and Randy L Buckner (2010). "Functional-anatomic fractionation of the brain's default network". In: *Neuron* 65.4, pp. 550–562.

Leech, Robert, Salwa Kamourieh, Christian F Beckmann, and David J Sharp (2011). "Fractionating the default mode network: distinct contributions of the ventral and dorsal posterior cingulate cortex to cognitive control". In: *Journal of Neuroscience* 31.9, pp. 3217–3224.
