# Supplementary material for: Extracting representations of cognition across neuroimaging studies improves brain decoding
Source: PLoS Comput Biol. 2021 May 3;17(5):e1008795. doi: 10.1371/journal.pcbi.1008795 (PMC8118532; doi:10.1371/journal.pcbi.1008795)
Supplement: Attachment — Submitted filename: response.pdf [file pcbi.1008795.s006.pdf]

Response to review

We thank Reviewer 3 for their insightful review. In the previous revision, we did not convey the various points that we discussed with Reviewer 3 sufficiently clearly in the text. This was not intentional, and we have endeavored to correct this. Accordingly, we clarified the scope and limitations of our work in the discussion.

However, in the manuscript, the authors DO NOT convey this limitation as candidly. The only mention of the limitation "around line 331" is in the results, not discussion. The authors state: " The MSTONs each plays a role in decoding a subset of 323 contrasts. They capture both low-level and high-level cognitive signal." "The components related to low-level functions tend to have structure-function links that are easier to interpret, high-level functions are also well captured."

We have corrected the text around both of these sentences to insist on that the lower-level components are more interpretable than their high-level counterparts –around line 323 and 334.

There is no revision to the discussion, which was requested by this reviewer. Instead, the authors make two sweeping claims in the discussion without caveat or clarification: 1) While extracting a universal basis of cognition is beyond the scope of a single fMRI study, we show that a joint predictive model across multiple studies finds meaningful approximations of atomic cognitive functions.

We have modified the corresponding paragraph –around line 444– to acknowledge better that our contribution is only a small step forward to the big-picture goal of aggregating information across studies, insisting on the main limitation –that MSTONs delineation of atomic cognitive functions is inherently coarse–, and pointing out possible improvements.

2) In this regime, aggregating existing studies to a new one using a multi-study model as the one we propose is likely to improve decoding performance.

The reviewer is right in that our multi-study decoding will likely not bring much to analyze the results of a very uncommon fMRI task study. Likewise, it will struggle for very specific and high-level cognitive functions. We now acknowledge this limitation in the discussion l. 408-410.

On the other hand, the manuscript does give clear evidence that for the vast majority of the studies in our corpus aggregating the other studies improves decoding performance (figure 2). Moreover, this improvement is present either when we reuse previously learned MSTONs or when we retrain the full multi-study decoding pipeline, as demonstrated in Fig. 9, to which we now refer in the discussion. Many newly produced fMRI studies probes cognitive tasks that are related to previous studies: our approach grounds the accumulation of knowledge in decoding, as it avoids training new decoding models from scratch.

Also in the discussion, the authors write: "Second, MSTONs appears more skewed towards known coordinated brain networks (Fig 4 and Fig 5), that differs from the networks recruited at rest." This statement is factually inaccurate. First, there is a not more noise in the spatial distribution of the MSTONs, which renders the phrase "known coordinated brain networks" untrue. Second, resting-state networks have been robustly related to task-based coactivation patterns (Smith et al 2009 PNAS, ; Laird et al 2011 JOCN).

MSTONs networks are indeed more noisy than resting state networks—we have adjusted the formulation in the discussion to explicitly state this. However, we have shown and discussed in Fig 4 and 5 how they

relate to known coordinated brain networks. Resting-state networks and task-based activation patterns are indeed intimately related, and we incorporate this knowledge in our model via the first layer of our architecture is learned from resting state. Yet, various high-level task recruit different combinations of basic brain structures, an aspect of brain organization which can be captured by a task-driven approach as the one that we contribute and less-so by unsupervised methods as used on resting-state data. We now recall in the discussion –around line 454– the links between task and resting-state network, and discuss with more nuances the differences between this literature and the MSTON approach.

When the authors wrote in their response letter, "We have addressed the substance of the reviewer's comments by modifying the manuscript in multiple places, including reworking completely our discussion on the high-level functional structures present in the MSTONs (fig 5), and better discussions on the interpretations of our results." I expected revision to the discussion section of the manuscript. This did not happen.

We have added at the end of the discussion a full paragraph that recalls the scope of our method, and its associated limitations (l. 470-484).
