# Supplementary material for: Extracting representations of cognition across neuroimaging studies improves brain decoding
Source: PLoS Comput Biol. 2021 May 3;17(5):e1008795. doi: 10.1371/journal.pcbi.1008795 (PMC8118532; doi:10.1371/journal.pcbi.1008795)
Supplement: Attachment — Submitted filename: Review.docx [file pcbi.1008795.s003.docx]

The authors present a modified algorithm for decoding brain activity across a variety of tasks measured with fMRI. The "task-optimized network decoder" is a supervised three-layer algorithm that models brain activity across multiple tasks using 1) static structural weightings derived from resting state data, 2) task-general network weightings derived from the shared variance across tasks with varying demands (MSTONs), and finally 3) task-specific weightings that relate to demand-specific contrasts. The authors compare this method of decoding to traditional voxelwise single-layer, single study methods, investigating their voxel-level accuracy for different sample sizes and across tasks. They show that the task-optimized network decoder demonstrates increased decoding accuracy across most tasks versus both voxel-level methods and methods that only include layers derived from resting state data. They also demonstrate (supplementary material) that, once generated, applying the two task-general layers can improve decoding in untrained tasks above a single-task decoder. The authors then explore the interpretational utility of the task-general MSTON weightings, which extract systematic continuities present in the brain across tasks. They also investigated the differences between contrast-specific classification maps produced by the task-optimized network decoder and voxelwise models and the relationship between maps from different cognitive demands. Here, they found that the maps from the task-optimized network decoder were smoother than those from voxelwise methods, and hierarchical clustering of the correlations between maps revealed clearer cognitive relationships between different contrasts for the former over the latter.

The task-optimized network decoder presented by the authors is an intriguing new method of decoding that takes advantage of the structure present in the brain both anatomically and functionally. The manuscript is well-written and presents the model in an intuitive way, making it accessible to both modelers and the greater neuroscience community. I would also commend the authors for making their model software and the data and results from this work open access. In general, the presented work is an excellent contribution to science. I would offer the following critiques and suggestions to further characterize the results of the task-optimized network decoder and better explore the interpretations and limitations of the method, more or less in order of importance.

**Major Suggestions**

1. The Materials and Methods section of the main text should describe the analytical methods used to produce the various results used to verify and explore the model, not just the methods of the decoder itself. Additionally, I would like to see the following methods issues clarified in the main text and in more detail:
   a. The validation metrics are slightly more complex than simple classifier accuracy due to the multi-task nature of the model; the authors should present not only how they calculated their metrics, but also how they are interpreting these metrics.
   b. On page 7/Figure 2b, the authors reference that projecting data onto a set of resting state "functional networks" does not significantly improve decoding. From my understanding of the various supplement descriptions, these functional networks are the set of 512 and 128 components derived from resting state data using a three-layer model, described in supplements S1.4 and S1.5. The authors should make this clear, as readers may interpret these as being more akin to the canonical resting state networks usually referred to by this term in the literature. (see point 2 for additional discussion on interpretation of the components produced by the model)
2. The authors describe their layers 1 and 2 as representing a set of 512 "resting state networks" and 128 task-general "multi-study task-optimized networks" (MSTONs). Per the supplement S1.5, the resting state networks are produced in a way that minimizes overlap; the second layer does not appear to have this constraint. The authors should expand their discussion of how these layers relate to brain structure and function at different scales, and how this might practically contribute to the improvements in performance conferred by the task-optimized network decoder. I would also caution the authors to consider caveating or removing their use of the term "networks" for these components, which are rather far from the literature's intuition of brain networks as a concept. Specific discussion points I think would be worth discussing and some context for their inclusion here are listed below.
   a. In the case of the resting state-derived Layer 1, the level of granularity is more similar to the region scale of brain parcellations, and in fact is even a higher resolution than many regional atlases. Thus, it might be more accurate and indeed fruitful to interpret this layer in a regional context; layer 1 of the model defines the boundaries of regions of the brain that show consistent activity within themselves at rest. Also, it is not mentioned in the methods nor the supplement that any spatial constraints were placed on the component weighting; if so, it seems in and of itself validating that the model naturally segregates voxels into small contiguous cortical areas.
   b. In the case of the MSTONs from Layer 2, these appear to have more network-like coverage, but are not whole brain networks in the canonical sense. Based on my understanding of the model, the layer 1 weightings go into training the weightings for layer 2, so the task data used for these weightings is effectively constrained by predefined regions. The model then pulls out 128 components that represent consistencies in the data across tasks. It would indeed seem accurate to say that these components represent sets of regions that tend to have coordinated activity across tasks, i.e., functional networks. However, typical resting-state network parcellations have markedly fewer components, generally fewer than 20. These two types of components are certainly not mutually exclusive; while brain regions may naturally show preference for a particular network structure at rest, network allegiances likely shift as task demands require coordination across different sets of brain regions. The authors should discuss how their MSTONs relate to canonical whole-brain networks, and what they might contribute to understanding how regions coordinate their functions. They might also address how the overlap between MSTONs may relate to the various functions they support.
   c. Related to the previous point, as the MSTONs appear to represent diffuse networks of regions, it seems reductive to then label individual MSTONs with the name of an individual brain region. This detracts from the interpretation of the role of the MSTON as a whole, even if a single region or small set of subregions may be particularly strongly associated with that network. This is especially clear in the case of the "Left DLPFC" network in figure 1, which even in the manuscript is acknowledged as being equally associated with left parietal cortex. At the same time, it seems clear that the MSTONs tend to preferentially represent one or a few contiguous regions or their bilateral pairs, which would be expected as neighboring and hemispherically homologous regions tend to be more correlated with one another than distant regions. The authors should discuss the interpretation of their MSTONs from both a network perspective and a region-level perspective, as it seems like they may serve as a stepping stone between the two. They also might consider removing the explicit region-name labels from figure 4 and instead let the word clouds serve to highlight the functional contributions of the MSTONs.
3. While the improvements to classification accuracy are relatively clear, it is less obvious to me how to interpret the differences in the classification maps between the voxelwise and task-optimized network decoders. The authors seem to suggest that the classification maps from the task-optimized network decoder are inherently better than those of the voxelwise decoder outside of a small set of localizers, appearing smoother and better capturing the boundaries of relevant functional regions. This is likely being driven by the data reduction steps in the first layer of the model, which associates groups of voxels with contiguous brain regions (see point 4 below for additional discussion). This would appear to bias any classification map to smooth, well-bounded regions, regardless of ground truth. The authors should address this bias in their discussion of these maps. I would also suggest that the authors include a map of the univariate contrast maps from the studies presented in Figure 5 for comparison, as this would show which regions are explicitly more active in one condition versus the other. Notably, a similar bias from the layer 2 networks would likewise explain why the task-optimized network decoder maps tend to produce networks of regions even in contrasts that should show fairly focal results, in line with the authors' maps from the vertical checkerboard study.
4. In most cases (if not all), the task-optimized network decoder is trained on more data than the individual study models by definition, which could trivially contribute to its improved performance outside of potential benefits from the additional layers. This would likewise be magnified for comparisons with datasets from smaller sample sizes, as was shown in Figure 2C, and would also explain the relatively small benefits seen for the HCP and Cam-Can data. The authors could train their decoder on a subset of the total available data and assess its performance versus a comparatively sized single study model, or should otherwise find some way to address this potential confound.

**Minor Suggestions**

1. Given the clear relevance of sample size for the extent of decoder accuracy improvement, it would be helpful for the authors to list the sample sizes from each study as a part of figure 2a.
2. The use of the subfigure labels for figure 4 seems unnecessary, and I would recommend simply removing them. However, if the authors wish to retain them, the caption for figure 4 should likewise include subcaptions for parts a, b, and c, to make it clear what these parts are referencing.
3. There is an errant space in the word "The" in the first sentence of the "Architecture, constraints, training" paragraph in supplement S2.6.
4. The authors should clarify which template space (MNI, Talairach, other) their task data are registered to (S1.1).

Savannah L. Cookson

University of California, Berkeley
